# Gender Differences in the Dynamics and Kinematics of Running and Their Dependence on Footwear

**DOI:** 10.3390/bioengineering11121261

**Published:** 2024-12-12

**Authors:** Tizian Scharl, Michael Frisch, Franz Konstantin Fuss

**Affiliations:** 1 Division of Biomechatronics, Fraunhofer Institute for Manufacturing Engineering and Automation IPA, D-95447 Bayreuth, Germany; franz.konstantin.fuss@ipa.fraunhofer.de; 2 Chair of Biomechanics, Faculty of Engineering Science, University of Bayreuth, D-95447 Bayreuth, Germany; michael.frisch@uni-bayreuth.de

**Keywords:** sex, female, male, barefoot, shoe, biomechanics, ground reaction force, motion capture, user experience, non-parametric statistics, Cliff’s Delta

## Abstract

Previous studies on gender differences in running biomechanics have predominantly been limited to joint angles and have not investigated a potential influence of footwear condition. This study shall contribute to closing this gap. Lower body biomechanics of 37 recreational runners (19 f, 18 m) were analysed for eight footwear and two running speed conditions. Presenting the effect size Cliff’s Delta enabled the interpretation of gender differences across a variety of variables and conditions. Known gender differences such as a larger range of hip movement in female runners were confirmed. Further previously undiscovered gender differences in running biomechanics were identified. In women, the knee extensors are less involved in joint work. Instead, compared to men, the supinators contribute more to deceleration and the hip abductors to acceleration. In addition to differences in extent, women also show a temporal delay within certain variables. For the foot, ankle and shank, as well as for the distribution of joint work, gender differences were found to be dependent on footwear condition, while sagittal pelvis and non-sagittal hip and thigh kinematics are rather consistent. On average, smaller gender differences were found for an individual compared to a uniform running speed. Future studies on gender differences should consider the influence of footwear and running speed and should provide an accurate description of the footwear condition used. The findings of this study could be used for the development of gender-specific running shoes and sports and medical products and provide a foundation for the application of smart wearable devices in gender-specific training and rehabilitation.

## 1. Introduction

This Introduction Section provides a comprehensive overview of the current state of the literature on the following:

- Gender differences in running biomechanics (Section 1.1);
- The effect of footwear condition on running biomechanics (Section 1.2);
- Gender differences in running biomechanics across multiple footwear conditions (Section 1.3).

This overview is given before

- synthesising the emerging knowledge gap in the existing literature and outlining the aim and relevance of the present study (Section 1.4).

### 1.1. Gender Differences in the Dynamics and Kinematics of Running

Numerous gender differences in endurance running are well documented with regard to pacing strategy, running biomechanics, energy expenditure, recovery and incidence of running-related injuries [1]. For instance, women appear to be more than twice as likely as men to develop patellofemoral pain syndrome [2]. Regarding ground reaction forces (GRFs), women reach a lower vertical active peak [3,4], but a higher vertical load rate [3,5]. However, no significant differences were observed for peak anteroposterior and mediolateral forces [3,5,6].

Using principal component analyses, gender differences were found in principal movements associated with hip adduction [7,8], pelvic tilt [7] and rotation [8]. When comparing running trials in terms of running speed, gender and shoe conditions, gender was differentiated based on intermediate principal components, which are characterised by thigh and shank movement in the transverse plane [9]. For hip adduction and internal rotation, women exhibit greater peak angles [5,10,11,12,13,14,15], greater angles during specific time points [5,14,15,16] and a larger range of motion (ROM) [7,11,17,18]. In the sagittal plane, one study showed larger hip flexion at foot strike [5], while another study detected less hip flexion at foot strike and larger hip extension at toe-off [6]. The majority [5,12,13] did not find significant differences for peak hip angles in the sagittal plane. Despite the fact that the knee joint, due to its functional anatomy, is constrained in its ability to ad-/abduct, numerous studies concluded that women show a larger ROM in frontal plane [7], greater peak knee abduction [6,12,13,14,19,20] and reduced peak knee adduction angles [15]. In the sagittal plane, the female knee seems to be more flexed during foot strike [3,5], shows a reduced peak flexion [19,20], and is more extended at toe-off [3,14]. The results for joint angles in the transverse plane are contradictory [5,6] or insignificant [10,12,13,16]. The female ankle seems to be slightly more pronated at foot strike [5,21] and toe-off [6,21]. However, results for peak angles are contradictory [6,13,14,21,22] and differences in the ROM are insignificant [6,21,23]. The ROM in the sagittal plane, on the other hand, appears to be larger in female runners [23,24]. Ferber et al. [12] found higher peak angular velocities in all three planes of the hip and in the transverse plane of the knee, but without testing these differences for significance. The peak plantarflexion velocity of females seems to be higher [5], while there is no significant difference for peak pronation velocity [5,22]. Women might run with a more anteriorly tilted pelvis [7] and exhibit a larger pelvis ROM in the frontal and transverse planes [11,17,24]. Female runners seem to strike with a smaller sagittal foot–ground angle but with a higher anterior tilting velocity of the foot segment [5].

According to Isherwood et al. [5], women show smaller peak moments in the sagittal plane of the hip, knee and ankle joints, while there are no significant differences in the other planes. In contrast, Sinclair et al. [25] revealed greater knee extension and abduction moments in female runners. A single study compared joint work between genders, demonstrating that women generate more negative work in the hip frontal and transverse plane, but there are no differences in the other joints [12]. A few studies [10,20,25,26] also investigated gender differences in tendon loads, joint forces and stresses. However, muscle moment arms and cross sections as well as articular surface contact areas were only estimated as a function of the instantaneous joint angle, while factors such as gender, body weight, height and joint dimensions were not considered. It can be assumed that musculoskeletal loads calculated in this way provide no additional value over specifying the underlying joint moments. Despite numerous investigations, many aspects of biomechanics were not considered when analysing gender differences. These include peak joint angular velocity, peak joint power, ankle joint work, body segment orientation and angular velocity. In contrast to investigating parameters at specific time markers of the running cycle, e.g., touch-down, toe-off or force peaks, analysing variables continuously over time provides deeper insight into gender differences. A few studies visualised differences in joint angle [6,10,11,12,13,15,17,19], joint moment [10,12,15,25], joint power [12] and pelvic orientation [11,17,24] time series. However, none of these studies analysed gender differences in these variables over time statistically. The partially contradictory results in the current literature could be explained by the differences in running conditions given in Table 1. Participants ran overground or on a treadmill at different speeds, either a prescribed uniform or a subject-dependent speed within the study. The footwear condition was either standardised, varying from barefoot to stability shoes, was not specified or subjects wore their personal running shoes.

### 1.2. Effect of Footwear Condition on the Dynamics and Kinematics of Running

The characteristics of a running shoe, e.g., weight, heel-to-toe drop (HTTD), longitudinal bending stiffness (LBS), motion control technologies and the overall degree of minimalism, can influence numerous aspects of running such as performance, economy and kinetics [27]. While running speed and gender affect the first and intermediate principal components of the kinematics, differences in shoe condition tend to affect higher principal components [9].

Vertical peak force and impulse may increase with the mass of a shoe. A heel-to-toe drop (HTTD) of below 5 mm seems to reduce the peak knee flexion angle and the peak knee extension moment [28], while peak ankle plantarflexion moment and vertical loading rate increase [29]. The running situation appears to possibly influence effects of footwear conditions, as a low HTTD has opposite effects on vertical GRF during overground vs. treadmill running [30]. The influence on susceptibility to running-related injuries (RRI) appears to be person-dependent, with occasional runners having a reduced risk of injury and regular runners having an increased risk when running with shoes with a low HTTD [31]. The sole hardness influences both the timing of the impact peak force (shock spike) and the peak dorsiflexion angle [32], but not the risk of RRI [33]. An increase in longitudinal bending stiffness (LBS) is usually achieved by inserting a carbon plate into the sole. A larger LBS is associated with longer ground contact times and reduced range of motion of the hip and metatarsophalangeal joint [34] as well as an extension of the ankle joint moment arm while the peak ankle joint moment remains unaffected [35]. Motion control systems are designed to provide support for the medial side of the foot and the longitudinal arch through reinforcing elements. Motion control shoes have been shown to reduce foot pronation [36,37] and tibial rotation [36,38]. However, only runners with strongly pronated feet appear to benefit from this effect regarding a reduction in RRI risk [39]. The categorization of running shoes on a spectrum from minimalism to maximalism takes several factors into account, with minimal shoes being characterised by a small mass, low HTTD, low number of motion control technologies and low LBS, with maximal shoes being on the opposite side of the range. Compared to conventional running shoes, running with minimal shoes results in a larger stride frequency [40], a higher vertical loading rate and a more pronounced peak pronation [41], while maximal shoes exhibit a higher peak pronation and a prolonged time in the pronation position [41] and an increase in impact peak, loading rate and stiffness of the leg complex [42], as well as exhibited a decrease in running economy for females who were not used to running with maximal shoes [43]. Although the results of the shoe comparisons in terms of minimalism are sometimes more difficult to interpret, they are important because they reflect the differences between the shoes available on the market and therefore worn in daily life.

### 1.3. Gender Differences in the Dynamics and Kinematics of Running Across Multiple Footwear Conditions

Few studies have investigated different footwear conditions with both male and female subjects. Two of them only investigated the influence of footwear conditions on running biomechanics and economy, but did not take gender differences into account [41,44]. Two further studies investigated the differences between female and male runners, but only analysed them across all shoes without looking at interactions of gender and footwear conditions [7,45]. One study examined gender differences in plantar pressure separately for a racing flat and a traditional running shoe [46]. While some gender differences were consistent across footwear conditions, e.g., women showing larger contact area at the medial forefoot, others were influenced by the footwear condition; e.g., compared to male runners, the force at the medial midfoot of female runners was smaller with the racing flat condition but higher with the training footwear condition. These results indicate that footwear condition is an influential variable of running-related gender differences, not only in magnitude but possibly also in the direction of the effects. Analysing running trials of female and male participants wearing six different running shoe conditions, Schrödter et al. [47] found a higher footwear-related variability of knee joint kinematics in female runners compared to males. This indicates that women and men react differently to changes in footwear characteristics and therefore gender differences in biomechanics may be affected by footwear condition. However, to the best of the authors’ knowledge, there is no study that has directly compared gender differences in the dynamics and kinematics of running for different footwear conditions.

### 1.4. Knowledge Gap, Aim and Relevance

In summary, while there is a good understanding of gender differences in running regarding joint angles and the effect of certain footwear characteristics on running biomechanics in general, the knowledge gap emerging from this extensive literature review on gender differences in running style is as follows:

- Gender differences in joint angular velocity and power;
- Gender differences in body segment orientation and angular velocity;
- Gender differences in biomechanical variables over time;
- Effect of footwear condition on gender differences.

Therefore, the aim of this study is to gain a deeper insight into gender differences in running biomechanics by (1) investigating a total of 276 biomechanical parameters, (2) statistically analysing differences not only at specific time points but also continuously over the entire stance phase for a total of 61 biomechanical variables and (3) examining potential effects of footwear condition on gender differences in running biomechanics.

These findings may contribute to a better understanding of differing injury patterns between genders, provide insights for the development of gender-specific sports and medical products in general and running shoes in particular, be used to determine gender-specific target and threshold values in the context of biofeedback in training and rehabilitation and help to reconcile the existing literature on gender differences in running biomechanics with the variable of footwear condition.

## 2. Materials and Methods

The Materials and Methods Section provides details about the following:

- Participants (Section 2.1) and footwear conditions (Section 2.2) selected for this study;
- Experimental procedure including force plates and motion analysis system as well as a user experience study (Section 2.3);
- Calculation of biomechanical parameters including lower body model and equations (Section 2.4);
- Statistical calculations for assessment of gender differences using *p*-value and Cliff’s Delta (Section 2.5).

### 2.1. Participants

Subject requirements were freedom from injury, a minimum weekly running distance of 12 km and an age between 20 and 45 years. Forty recreational runners participated in the study after confirming that they had not suffered any injuries or muscular disorders of the lower body in the six months prior to the test session. None of the participants were known to have any gait disturbances. Three participants were excluded due to missing force plate or trajectory data, resulting in thirty-seven participants to be analysed (Table 2). All subjects gave their informed consent for inclusion before they participated in the study. The study was conducted in accordance with the Declaration of Helsinki and was approved by the Ethics Committee of the University of Bayreuth (Approval-No. 23–041).

### 2.2. Footwear Conditions

**Figure 1:**
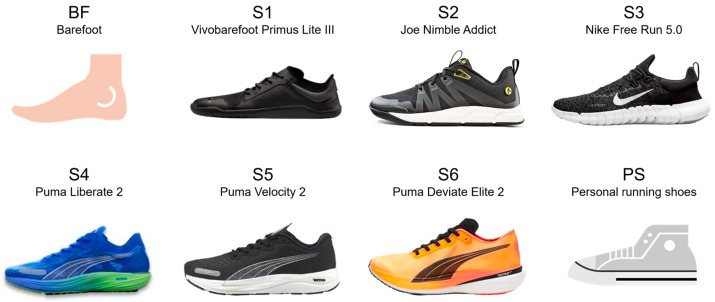
Footwear conditions (S = shoe).

The participants were asked to run with eight different footwear conditions consisting of barefoot (BF), the participants’ personal running shoes (PS), and six predetermined shoe models (S1–S6) within the spectrum of minimalist to maximalist running shoes. To determine the degree of minimalism, a “Minimalist Index” taken from Esculier et al. [48] was calculated from mass, heel-to-toe drop, stack height, amount of motion control technologies and longitudinal as well as torsional flexibility of the shoes. Footwear conditions are presented in Figure 1 and specified in Table 3. To avoid brand-specific size discrepancies, the shoe sizes were selected for a foot length of 25, 27 and 29 cm. For most brands, this corresponds to UK shoe sizes of 6, 8 and 10, respectively. The Vivobarefoot Primus Lite III (S1) is a barefoot shoe with an extremely thin and flexible sole. The Joe Nimble Addict (S2) promises maximum toe freedom thanks to a particularly wide toe box with a slightly higher stack height and a heel-to-toe drop of 0 mm. The Nike Free Run 5.0 (S3) is an extremely lightweight shoe with a highly flexible sole. The Puma Liberate 2 (S4) is a lightweight running shoe without special stability devices. The Puma Velocity 2 (S5) is a standard running shoe with the highest stack height in this study. The Puma Deviate Elite 2 (S6) is a performance running shoe with an integrated carbon plate and a strong rocker shape. The characteristics of the participants’ personal running shoes are given in Table 4 for a reference shoe size of UK8 to enhance comparability. Average mass, heel height and heel-to-toe drop of PS were closest to the standard running shoe tested (S5). Personal shoes of the female subjects had a stack height approx. 4 mm higher than those of the males.

To determine the cushioning properties of the six predetermined running shoes, the heel segments of the shoes in reference size UK8 were mechanically tested in a compression test with a hemispherical indenter (Ø 50 mm) at a deflection rate of 2 mm/min (Z050, ZwickRoell GmbH & Co. KG, Ulm, Germany). Energy absorption in the heel segment was determined for the fifth load cycle at compression forces of 500, 1000, 1500 and 2000 N as the integral of the force–deflection curve. Results of the left and right shoe were averaged.

### 2.3. Experimental Procedure

**Figure 2:**
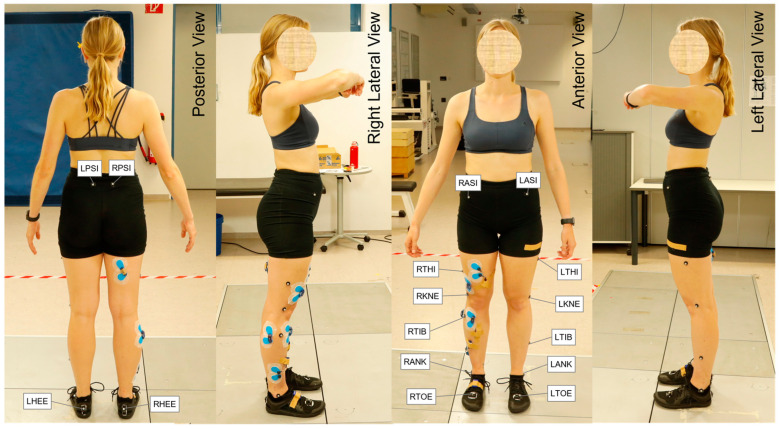
Marker set-up consisting of 16 infrared reflective markers; data recorded by the EMG sensors shown are not part of this paper; L = left; R = right; ASI = spina iliaca anterior superior; PSI = spina iliaca posterior superior; THI = lateral thigh; KNE = epicondylus lateralis femoris; TIB = lateral shank; ANK = malleolus lateralis; HEE = heel; TOE = articulatio metatarsophalangealis II.

All subjects wore skin-tight shorts and short socks (Figure 2). Men performed the test shirtless while women wore a sports bra. The participants were first informed about the procedure and confirmed their informed consent with their signatures. They were then asked to fill in a questionnaire about their running behaviour and experience, information about their own running shoes (brand, model, age) and their running injury history (frequency and location of pain; time, place, and description of running-related injuries). Body mass, body height, foot and leg dimensions and Q-angle were measured. The Q-angle describes the orientation of the quadriceps femoris muscle in relation to the patellar tendon. It was measured as the included angle between the direction of pull of the quadriceps femoris muscle (connection between the anterior superior iliac spine and the patellar centre) and that of the patellar tendon (connection between the patellar centre and the tibial tuberosity) using a goniometer with the subject lying supine.

Lower body movements were recorded with an eight-camera infrared 3D motion capture system (Vicon MX3+, Vicon Motion System Ltd., Oxford, UK) at a frequency of 100 Hz. A total of 16 infrared reflective markers (diameter 9.5 mm) were placed at the anterior superior iliac spine, posterior superior iliac spine, lateral knee, most lateral point of the fibular malleolus, second metatarsophalangeal joint and heel, as well as one point each on the connecting lines between hip, knee, and ankle joints (Figure 2). The lateral knee marker was placed at the point moving the least while the subjects alternately flexed and extended their legs to estimate the position of the axis of rotation of the knee. The markers were attached to the skin with double-sided adhesive tape suitable for this purpose and to the shoes with Velcro tape. An individual marker model was calibrated for each participant in Nexus 2.13 (Vicon Motion System Ltd., Oxford, UK) using a dynamic recording in which the hip, knee and ankle joints were moved to their full extent.

GRF and centre of pressure (COP) were recorded using two 3D force plates (9287, Kistler Instrumente AG, Winterthur, Switzerland). Running speed was measured using two light gates (Speedtrap 2, Brower Timing Systems, Draper, UT, USA) placed 4 m apart with the force plate in between.

After a five-minute warm-up with the subject’s personal running shoes, runs were performed in a randomised order with the eight footwear conditions. For each condition, the participant first ran on a treadmill (stellar med, h/p/cosmos sports & medical GmbH, Nussdorf-Traunstein, Germany) for one minute each at 80% and 100% of their individual training speed as well as at 10 km/h. Six valid overground runs each were then recorded at the individual training speed and at a running speed of 10 km/h. The speed conditions were chosen to examine gender differences both at a uniform running speed and at the speed at which the subjects spent most of their training time. A test was deemed valid if only the whole right foot touched the force plates, and the running speed measured by the light gates was maintained with a 5% tolerance. In a user experience study following each footwear condition, participants rated their perception of shoe characteristics and physical perception using a visual analogue scale (VAS). The limits of the 100 mm long, continuous VAS reflect the extreme values of each parameter, e.g., 0—“extremely uncomfortable” and 100—“extremely comfortable”.

### 2.4. Data Analysis

#### 2.4.1. User Experience Study

The assessed parameters included shoe characteristics (general rating, comfort, support, performance, energy return, cushioning) and physical perception (joint load, joint stability, muscle work). The length in millimetres between the starting point of the VAS and the subject’s marker corresponds to the respective subjective rating of the parameter in a range between 0 and 100.

#### 2.4.2. Biomechanical Parameters

All recording markers were automatically labelled (for labels see Figure 2) using the Nexus 2.13 software (Vicon Motion System Ltd., Oxford, UK). The recording quality was reviewed for every trial. Unlabelled or swapped markers were manually relabelled, short gaps were filled according to Vicon’s recommendations, and recordings with anomalies regarding markers and force plates were discarded. Due to too many discards, the recordings of 3 subjects were completely rejected, resulting in a total of 37 subjects (19 females, 18 males). Data processing was completed in MATLAB R2022a (The MathWorks, Natick, MA, USA). GRF and COP were calculated from the electrical voltage values of the 16 analogue channels of both force plates and their sensor positions. The start (foot strike) and end (toe-off) of ground contact were determined at a threshold of 10 N. The measurement frequency of the marker trajectories was artificially up-sampled from 100 Hz to the force plate measurement frequency of 1000 Hz to not lose any information in the force data during kinetic calculations. For this purpose, the marker data were linearly interpolated and then filtered using a moving average filter with a window width of 11 frames.

**Figure 3:**
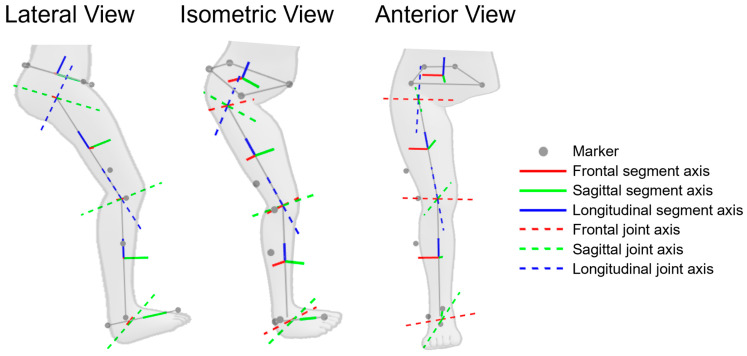
Biomechanical model of pelvis and lower right limb showing segment and joint axes.

For kinematic and kinetic calculations, a biomechanical model of the pelvis and lower right limb was created, incorporating the anatomical joint axes of the hip, knee and ankle (Figure 3).

The position of the hip joint centre (RHJC) in the local pelvis coordinate system with its origin at the midpoint of RASI and LASI (MASI) was calculated from pelvis width (PW) and pelvis depth (PD), where PW is the distance between RASI and LASI and PD is the distance between MASI and the midpoint of RPSI and LPSI (MPSI), using regression functions according to Harrington et al. [49] (Equation (1)) and was subsequently transferred in the global coordinate system.

(1) $${RHJC}_{loc}=\left(\begin{array}{c} 0.33PW+7.3\\ -0.24PD-9.9\\ -0.30PW-10.9 \end{array}\right)$$

$$\begin{array}{ll} where & {RHJC}_{loc}=position\;of\;right\;hip\;joint\;center\;in\;the\;local\;pelvis\;coordinate\;system;\\ & PW=pelvis\;width;\\ & PD=pelvis\;depth. \end{array}$$

Knee and ankle joint offsets (OFFs) are calculated from half the joint width (JW) and the distance between the skin and the centre of an applied marker, where this distance is calculated from the marker height, including the base (MH) and the marker radius (MR) (Equation (2)).

(2) OFF = JW/2 + MH − MR

$$\begin{array}{ll} where & OFF=offset\;between\;marker\;midpoint\;and\;joint\;center;\\ & JW=joint\;width;\\ & MH=marker\;height\;including\;marker\;base;\\ & MR=marker\;radius. \end{array}$$

**Figure 4:**
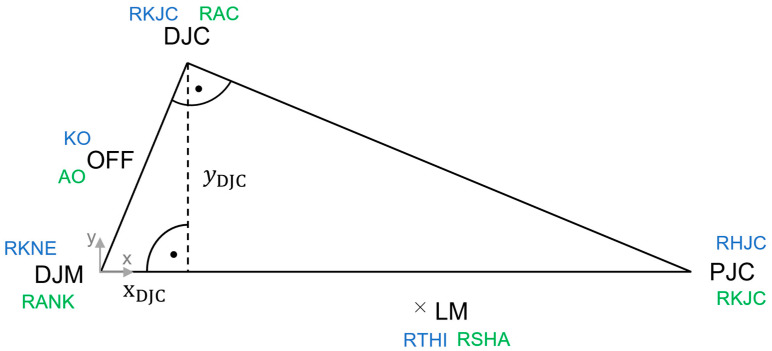
Local position of the distal joint centre (DJC) in the plane spanned by the proximal joint centre (PJC), distal joint marker (DJM) and lateral marker (LM). Corresponding markers for calculating the right knee joint centre (RKJC) are marked in blue and for the right ankle centre (RAC) in green.

To define the centres of knee and ankle joints, the position of the distal joint centre (DJC) is determined in a local two-dimensional coordinate system that lies in a plane spanned by the distal joint marker (DJM), proximal joint centre (PJC) and lateral marker (LM) (Figure 4) and is calculated using the Pythagorean and cathetus theorems (Equation (3)). The local position of the DJC is then transferred to the global coordinate system. The knee joint centre (RKJC) is determined from the position of the RHJC, the markers on the knee (RKNE) and thigh (RTHI) and the knee offset (KO). The ankle centre (RAC) is determined from the positions of the RKJC, the markers on the ankle (RANK) and shank (RSHA) and the ankle offset (AO). Based on the results of the anthropometric studies by Isman et al. [50], the talocrural joint centre (TCRUJC) is assumed to be 12 mm distal and 11 mm anterior to the RAC in the tibial coordinate system, and the talocalcaneal joint centre (TCALJC) is assumed to be 4% of the ankle width medial along the talocrural axis and 5 mm distal from the TCRUJC along the direction vector perpendicular to the talocrural and talocalcaneal axes.

(3) $${DJC}_{loc}=\left(\begin{array}{c} x\\ \sqrt{{OFF}^{2}-x^{2}}\\ 0 \end{array}\right),\;where\;x=\frac{{OFF}^{2}}{{∥({PJC}_{loc}-{DJM}_{loc})∥}_{2}}$$

$$\begin{array}{ll} where & {DJC}_{loc}=position\;of\;the\;distal\;joint\;centre\;in\;the\;local\;coordinate\;system;\\ & OFF=offset\;between\;marker\;midpoint\;and\;joint\;center;\\ & {PJC}_{loc}=position\;of\;the\;proximal\;joint\;centre\;in\;the\;local\;coordinate\;system;\\ & {DJM}_{loc}=position\;of\;the\;distal\;joint\;marker\;in\;the\;local\;coordinate\;system. \end{array}$$

The local pelvis coordinate system originates at MASI with the x-axis pointing from LASI to RASI, the y-axis pointing anteriorly from MPSI to MASI and the z-axis pointing perpendicular to the other axes in the superior direction. The local thigh coordinate system originates at RKJC with the x-axis pointing from RKJC to RKNE, the z-axis pointing superiorly from RKJC to RHJC and the y-axis pointing perpendicular to the other axes in the anterior direction. The local shank coordinate system originates at RAC with the x-axis pointing from RAC to RANK, the z-axis pointing superiorly from RAC to RKJC and the y-axis pointing perpendicular to the other axes in the anterior direction. The longitudinal foot axis points from RHEE to RTOE.

The hip joint is considered a spherical joint with RHJC as a pivot point. The x-, y- and z-axes of the local pelvis coordinate system are used for the angle calculations in the sagittal, frontal and transverse planes, respectively. The knee is assumed to be a bicondylar joint with 2 DOF (extension/flexion and rotation (constrained in extension); some literature sources also mention changes in ab/adduction angle). Knee rotation is assumed to be neutral at the time before the foot strikes the ground. Talocrural and talocalcaneal joints are assumed to be revolute joints. Based on the ankle anthropometrics provided by Isman et al. [50], the talocrural joint axis is obtained by tilting the local x-axis of the shank laterally by 10° and rotating it inwards by 6°, and the talocalcaneal joint axis by tilting the local y-axis of the shank posteriorly by 41° and rotating it inwards by 23°. Movements in the talocrural joint are considered by additionally rotating the talocalcaneal joint axis around the talocrural joint axis by the current talocrural joint angle. Following the clinical convention, joint angles are determined in the order of flexion/extension, adduction/abduction and internal/external rotation. All joint axes in the sagittal, frontal and transverse planes point laterally, anteriorly and superiorly, respectively.

**Figure 5:**
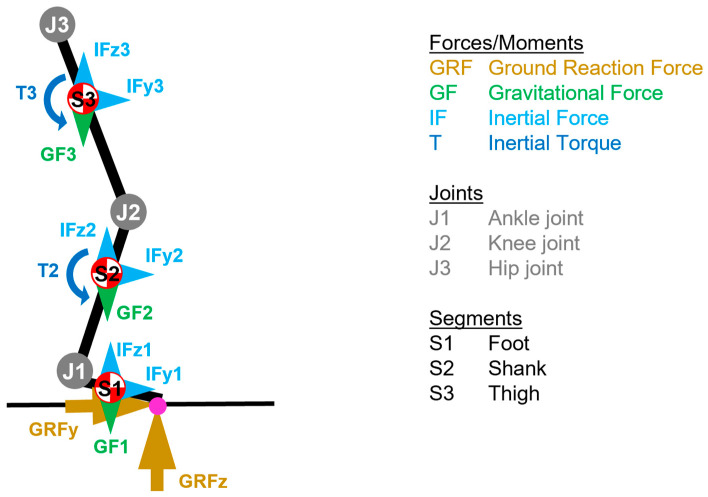
Sagittal representation of the forces acting on the right leg considered for the calculation of external joint moments.

Joint angles and moments in the counterclockwise direction are considered positive and in the clockwise direction negative. The joint angle of joint j about axis h (JA_j,h_) is determined as the angle between the proximal and distal segment axes projected onto the plane perpendicular to the joint axis (JAX_j,h_). Joint angular velocity (JAV_j,h_) is calculated as the average angular change over a period of 2 ms. The calculation of the external joint moments considers the GRFs and the gravitational forces (GFs), the inertial forces (IFs), and the inertial torques (Ts) of the segments except the torque of the foot (Figure 5). The external joint moment (JM_j,h_) of joint j about axis h (JAX_j,h_) is determined based on the COP, the joint centre (JC_j_), as well as the mass m_i_, the centre of mass COM_i_, the translational acceleration of COM_i_ a_i_, the angular acceleration α_i_ and the radius of gyration k_i_ of segment i (Equations (4)–(9)). Mass, centre of mass and radius of gyration of the segments are calculated from body mass and the position of the joint centres using the anthropometric data of Drillis et al. [51]. Joint power (JP_j,h_) is the product of the internal joint moment (−JM_j,h_) and JAV_j,h_ (Equation (10)). Based on visual inspections and strategic testing, GRF is filtered using a moving average filter (window width of 3 ms), COP is filtered using a moving median filter (window width of 11 ms) and angles and angular velocities are filtered using a running average filter (window width of 51 ms).

(4) $$M_{GRF,j}=\left(COP-{JC}_{j}\right)\times GRF$$

$$\begin{array}{ll} where & M_{GRF,j}=external\;moment\;of\;joint\;j\;caused\;by\;ground\;reaction\;forces;\\ & COP=centre\;of\;pressure;\\ & {JC}_{j}=position\;of\;the\;centre\;of\;joint\;j;\\ & GRF=ground\;reaction\;force. \end{array}$$

(5) $$M_{GF,j}=\sum_{i=1}^{j}\left({COM}_{i}-{JC}_{j}\right)\times (g\cdot m_{i})$$

$$\begin{array}{ll} where & M_{GF,j}=external\;moment\;of\;joint\;j\;caused\;by\;gravitational\;forces;\\ & {COM}_{i}=centre\;of\;mass\;of\;segment\;i;\\ & {JC}_{j}=position\;of\;the\;centre\;of\;joint\;j;\\ & g=gravitational\;acceleration;\\ & m_{i}=mass\;of\;segment\;i. \end{array}$$

(6) $$M_{IF,j}=\sum_{i=1}^{j}\left({COM}_{i}-{JC}_{j}\right)\times (-a_{i}\cdot m_{i})$$

$$\begin{array}{ll} where & M_{IF,j}=external\;moment\;of\;joint\;j\;caused\;by\;inertial\;forces;\\ & {COM}_{i}=centre\;of\;mass\;of\;segment\;i;\\ & {JC}_{j}=position\;of\;the\;centre\;of\;joint\;j;\\ & a_{i}=translational\;acceleration\;of\;segment\;i;\\ & m_{i}=mass\;of\;segment\;i. \end{array}$$

(7) $$M_{T,j}=\sum_{i=1}^{j}-\alpha_{i}\cdot m_{i}\cdot {k_{i}}^{2}$$

$$\begin{array}{ll} where & M_{T,j}=external\;moment\;of\;joint\;j\;caused\;by\;inertial\;torques;\\ & \alpha_{i}=angular\;acceleration\;of\;segment\;i;\\ & m_{i}=mass\;of\;segment\;i;\\ & k_{i}=radius\;of\;gyration\;of\;segment\;i. \end{array}$$

(8) $${JM}_{j}=M_{GRF,j}+M_{GF,j}+M_{IF,j}+M_{T,j}$$

$$\begin{array}{ll} where & {JM}_{j}=total\;external\;moment\;of\;joint\;j;\\ & M_{GRF,j}=external\;moment\;of\;joint\;j\;caused\;by\;ground\;reaction\;forces;\\ & M_{GF,j}=external\;moment\;of\;joint\;j\;caused\;by\;gravitational\;forces;\\ & M_{IF,j}=external\;moment\;of\;joint\;j\;caused\;by\;inertial\;forces;\\ & M_{T,j}=external\;moment\;of\;joint\;j\;caused\;by\;inertial\;torques. \end{array}$$

(9) $${JM}_{j,h}={JAX}_{j,h}\cdot {JM}_{j}$$

$$\begin{array}{ll} where & {JM}_{j,h}=external\;moment\;of\;joint\;j\;about\;axis\;h;\\ & {JAX}_{j,h}=joint\;axis\;h\;of\;joint\;j;\\ & {JM}_{j}=three-dimensional\;external\;moment\;of\;joint\;j. \end{array}$$

(10) $${JP}_{j,h}={-JM}_{j,h}\cdot {JAV}_{j,h}$$

$$\begin{array}{ll} where & {JP}_{j,h}=power\;of\;joint\;j\;about\;axis\;h;\\ & {JM}_{j,h}=external\;moment\;of\;joint\;j\;about\;axis\;h;\\ & {JAV}_{j,h}=angular\;velocity\;of\;joint\;j\;about\;axis\;h. \end{array}$$

GRF is normalised to body weight in N, whereas joint moments and hence also joint power, are normalised to body weight in N times body height in m. In this paper, the term “parameter” denotes single values (extrema, integrals or values at specific time markers), while values changing over time are referred to as “variable”.

The parameters presented include joint and segment angles at foot strike (FS), mid-stance (MS) and toe-off (TO), their extrema (MAX, MIN) and their range of motion (ROM), peak GRF, peak joint and segment angular velocities, peak internal joint moments and peak joint power, impulses, angular impulses and joint work, as well as ground contact time, proportion of the braking/deceleration phase and instantaneous and average vertical load rate. The transition from braking to propulsion phase is determined at zero-transition of the anteroposterior GRF. The instantaneous load rate is determined as the maximum and the average load rate in terms of the mean increase in force, both between 20% and 80% of the period from foot strike to impact peak [52].

Ground reaction forces, joint angles, joint angular velocities, external joint moments, joint powers, segment angles and segment angular velocities are further interpolated to 201 time points to compare variables visually and statistically between trials over time. This corresponds to a percentage of the stance phase from 0% to 100% with an increment of 0.5%.

Table 5 describes the orientations of the joint and segment axes for the interpretation of positive and negative values in biomechanical variables as well as joint and segment angles. Peak forces, peak angular velocities, peak moments, peak powers, impulses, angular impulses and joint work are presented in a direction-dependent manner and are therefore always positive.

To determine potential gender differences in time domain, selected variables are analysed for significant differences in the timing of the values. For this purpose, the recordings that have already been normalised in time are also normalised in magnitude, resulting in values between −1 and 1 or 0 and 1. After identifying strictly monotonic regions for the corresponding variables, the percentage of the stance phase is interpolated to values between the boundaries with an increment of 0.01. Thus, the percentage of stance phase at which a specific portion of the value, e.g., 100% of anteroposterior GRF, is reached can be statistically compared between trials.

### 2.5. Statistical Analysis

Statistical analysis was performed using MATLAB R2022a. Outliers in the anteroposterior impulse were identified using Tukey’s fences and excluded. The differences between female and male runners were analysed separately for the individual shoe and running speed conditions. The Shapiro–Wilk test showed that 77.84% of the data sets to be examined were not normally distributed. To ensure comparability, uniformity and clarity, all difference hypotheses were therefore tested using the non-parametric Mann–Whitney-U test. Non-parametric tests are less powerful than parametric ones. It can therefore be assumed that gender differences identified in this study will also appear and may even be more pronounced when carrying out parametric tests. In addition to the significance level, if *p* < 0.05, the effect size Cliff’s Delta (CD) is given for better interpretability of gender differences. Also known as the rank biserial coefficient for the two-sample case, it is a measure of overlap between two groups X and Y and is calculated according to Equation (11) by comparing the values of all f female runners and all m male runners with each other. CD gives values between −1 (all women show lower values than all men) and 1 (all women show higher values than all men). At a value of 0, the two groups are completely overlapping. Table 6 gives a more detailed categorization of CD.

(11) $$CD=\frac{1}{fm}\sum_{i=1}^{f}\sum_{j=1}^{m}\delta_{i,j},\;where\;\delta_{i,j}=\left\{\begin{array}{cc} +1, & x_{i}>y_{j}\\ -1, & x_{i}<y_{j}\\ 0, & x_{i}=y_{j} \end{array}\right.$$

$$\begin{array}{ll} where & CD=Cliff^{\prime }s\;Delta;\\ & x_{i}=value\;of\;female\;i;\\ & y_{j}=value\;of\;male\;j;\\ & f=number\;of\;females;\\ & m=number\;of\;males. \end{array}$$

**Figure 6:**
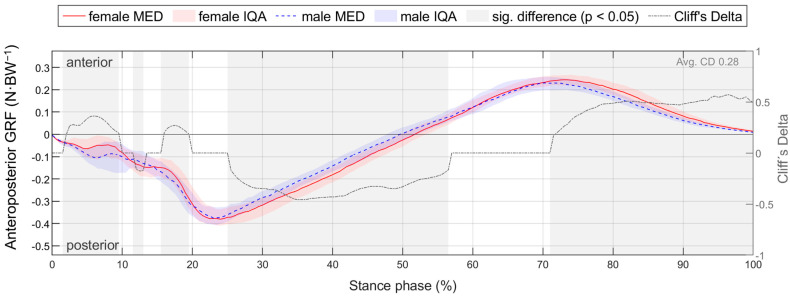
Exemplary graph illustrating gender differences in each variable as a function of time; MED = median; IQA = interquartile area shown as the interquartile range as a function of time.

For better comparability with the literature, gender differences for footwear condition S5, which represents a standard running shoe, are presented in detail in Section 3.2. To compare the time-normalised variables, medians (MEDs) and interquartile ranges (IQRs) of female and male runners as well as the resulting CD are plotted vs. time (Figure 6). This results in median and CD profiles and interquartile areas (IQAs). Periods with a significant gender difference (*p* < 0.05) are shaded in grey. Thus, it can be seen at any point in time whether there is a significant gender difference, how it is directed and how strong its effect is. Gender differences in parameters are shown in tabular form, with the lower quartile (Q1), median (MED) and upper quartile (Q3) of female and male runners; the significance level; and the effect size CD (Table 7). Figures illustrating biomechanical variables vs. time and tables listing biomechanical parameters are given exemplarily for ground reaction force in the corresponding section and can be found in the Appendix A and Appendix B for the other biomechanical quantities (e.g., joint angles). For each section, all parameters showing a significant gender difference with at least a medium effect are summarised in an illustrative figure.

**Figure 7:**
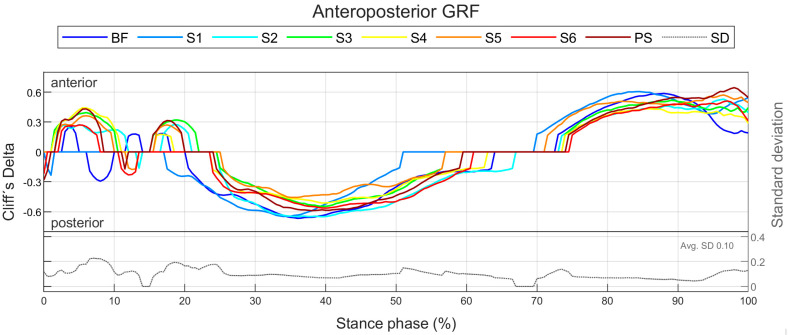
Exemplary graph comparing the effect size Cliff’s Delta of gender differences in each variable as a function of time for all footwear conditions; SD = standard deviation.

The effect of footwear conditions on gender differences is examined in Section 3.3 based on CD. For the variables, the CD curve already shown in Figure 6 for a single footwear condition is compared across all footwear conditions (Figure 7). This allows the effect of the respective footwear condition on direction, size, and duration of gender differences to be examined. The CD is only considered if the difference is significant (*p* < 0.05). For the example of the anteroposterior GRF, a positive CD states that females have a greater value than males. This means that women either have a greater anterior force (positive), a lower posterior force (negative) or an anterior force, while men show a posterior force. The standard deviation is given to illustrate periods in which gender differences in the individual footwear conditions differ most from each other. A comparison of the median curves of female and male runners for each variable, shoe and running speed condition can be found in the Appendix A, Appendix B, Appendix C, Appendix D and Appendix E. The results for the variables are summarised in tabular form by presenting the absolute CD averaged over time for all footwear conditions (Table 8). The averaged absolute CD value is between 0 and 1 and provides a statement about the strength and duration of gender differences, but no longer about the direction. As it is an average value over time, these values should not be classified using the conventional limits of 0.11, 0.28 and 0.43. Rather, they serve as a relative benchmark for comparing footwear conditions, taking into account not only the effect size but also the duration of gender differences. The mean value of CD across all footwear conditions (MV) provides a statement about the extent of gender differences across all footwear conditions, while the standard deviation (SD) averaged over time allows conclusions about the impact of footwear condition on gender differences.

To illustrate the effect of footwear condition on gender differences in biomechanical parameters, the CD already stated for a single footwear condition in Table 7 is compared across all footwear conditions (Table 9). MV and SD are given to quantify the extent of gender differences across all shoes as well as the spread in gender differences between footwear conditions. Figures illustrating gender differences in biomechanical variables vs. time and tables listing gender differences in biomechanical parameters are given exemplarily for ground reaction force in the corresponding section and can be found in the Appendix D and Appendix E for the other biomechanical quantities (e.g., joint angles). For each section, all parameters showing in at least one footwear condition a significant gender difference with a medium or large effect are summarised in an illustrative figure. The results are categorised according to their consistency across the footwear conditions. Parameters with a significant and equidirectional gender difference across all footwear conditions are labelled as *consistent* while parameters showing in at least one footwear condition a not significant or opposite gender difference compared to the other conditions are labelled as *inconsistent*.

## 3. Results

The Results Section is organised to provide insights into the following:

- The energy absorption of the mechanically tested running shoes (Section 3.1);
- Gender differences when running with standard running shoes (Section 3.2);
- Gender differences in running across multiple footwear conditions (Section 3.3);
- Gender differences in user experience for multiple footwear conditions (Section 3.4).

Due to the enormous number of findings on gender differences in running, the results are presented in the form of text, tables, and graphs in the interests of precise scientific documentation and are additionally summarised in figures for a quick overview of each subsection.

### 3.1. Energy Absorption of the Six Predetermined Running Shoe Conditions

**Figure 8:**
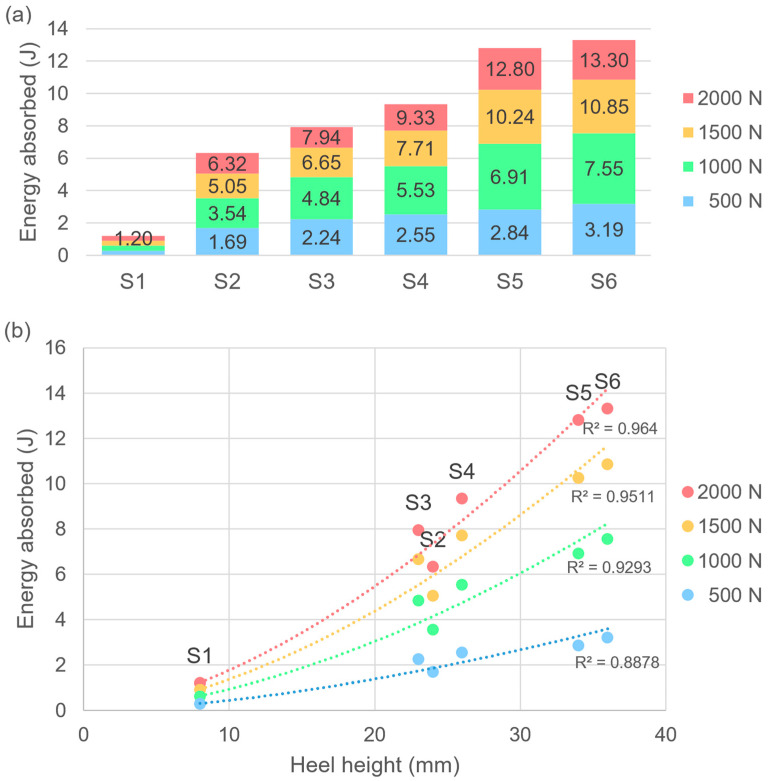
Energy absorbed by the heel segment (**a**) of the six predetermined running shoes at compression forces of 500 to 2000 N; in (**b**), the results are plotted versus heel height including a power function fit for each compression force. S1: Vivobarefoot Primus Lite III; S2: Joe Nimble Addict; S3: Nike Free Run 5.0; S4: Puma Liberate 2; S5: Puma Velocity 2; S6: Puma Deviate Elite 2. For S1 only, the absorbed energy at a force of 2000 N is stated.

At a compression force of 2000 N, the energy absorbed is in a broad range between 1.20 and 13.30 J (Figure 8a). The energy absorbed depends on the heel height, with higher heels absorbing more energy (Figure 8b). Deviations from the fitted power functions, such as the reduced energy absorption of S2, may be due to material differences.

### 3.2. Gender Differences in the Dynamics and Kinematics of Running with Standard Running Shoes

When running with a standard running shoe (S5, Puma Velocity 2; see Figure 1 and Table 3), the rearfoot strike is the dominant foot strike pattern. For women, the proportion of runs with a rearfoot strike is between 94 and 95%; for men, it is between 82 and 85% (Table 10). The average individual running pace was 10.74 (+/−1.05) km/h for females and 12.50 (+/−0.88) km/h for males. Running speed condition (individual pace vs. 10 km/h) does not influence foot strike pattern. 

#### 3.2.1. Gender Differences in Ground Reaction Force with Standard Running Shoes

**Figure 9:**
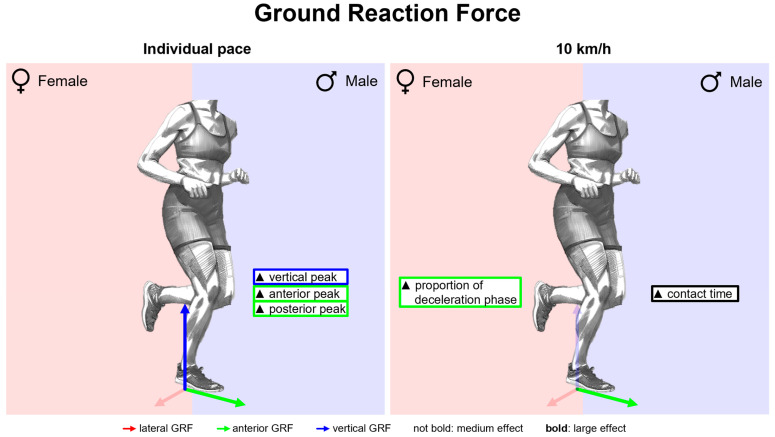
Gender differences in ground reaction force parameters with standard running shoes at the participants’ individual training pace and a running speed of 10 km/h; only significant differences with at least a medium effect size are presented and assigned to the gender with the higher value.

**Figure 10:**
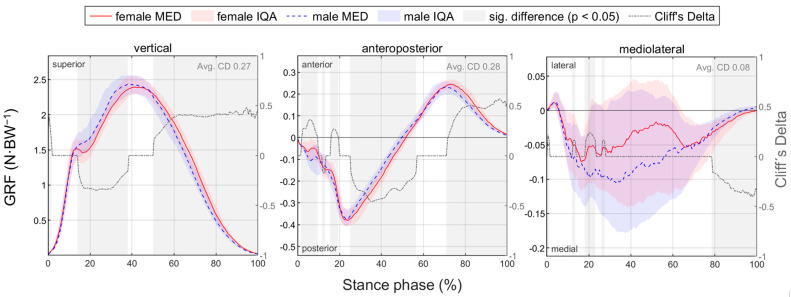
Gender differences in ground reaction force (GRF) when running with a standard running shoe (S5, Puma Velocity 2) at 10 km/h; MED = median; IQA = interquartile area; BW = body weight in N.

Figure 9 summarises notable gender differences in ground reaction force with standard running shoes. Figure 10 depicts the body weight-normalised GRF components vs. time. Women show higher posterior and lower vertical forces at mid-stance and higher anterior and vertical forces at toe-off (comparative expressions refer to significant differences with *p* < 0.05 throughout the paper). Due to high intra-gender variation, a significant difference in the mediolateral force can only be detected at the end of stance phase. Table 11 lists GRF parameters. At 10 km/h, women show a shorter stance phase, while at individual pace, their ground contact time is longer than that of men. The proportion of the deceleration phase in the stance phase is longer for women in both conditions, with a higher effect size at 10 km/h. The delayed transition from posterior to anterior GRF of female runners can be seen for 10 km/h in Figure 10. At individual speed, women show smaller peak forces in the vertical, anterior, posterior and medial directions. At 10 km/h, the peak medial force, the peak posterior force, and the vertical impulse are also smaller than those of male runners, while female runners exhibit a greater peak anterior force. Corresponding to the average population, in the present study, the individual training speed of women was slower than that of men despite a similar training volume (10.74 +/− 1.05 km/h vs. 12.50 +/− 0.88 km/h). The slower running speed of women at individual running speed explains the longer contact time and lower peak GRF and vertical load rate compared to men.

#### 3.2.2. Gender Differences in Joint Angles with Standard Running Shoes

**Figure 11:**
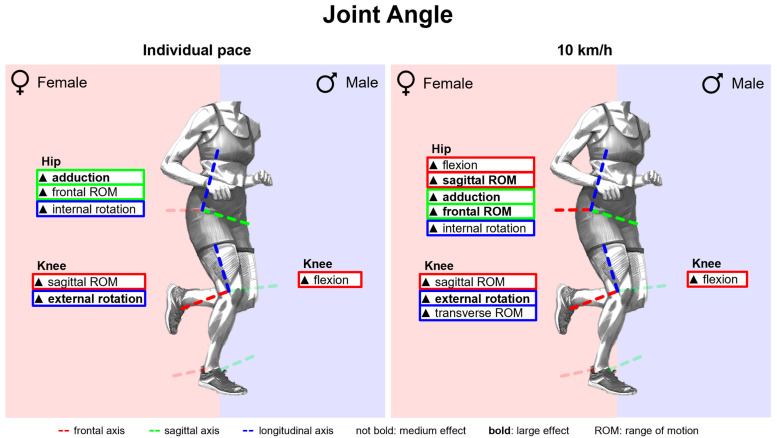
Gender differences in peak joint angles and ranges of motion with standard running shoes at the participants’ individual training pace and a running speed of 10 km/h; only significant differences with at least a medium effect size are presented and assigned to the gender with the higher value.

**Figure A1:**
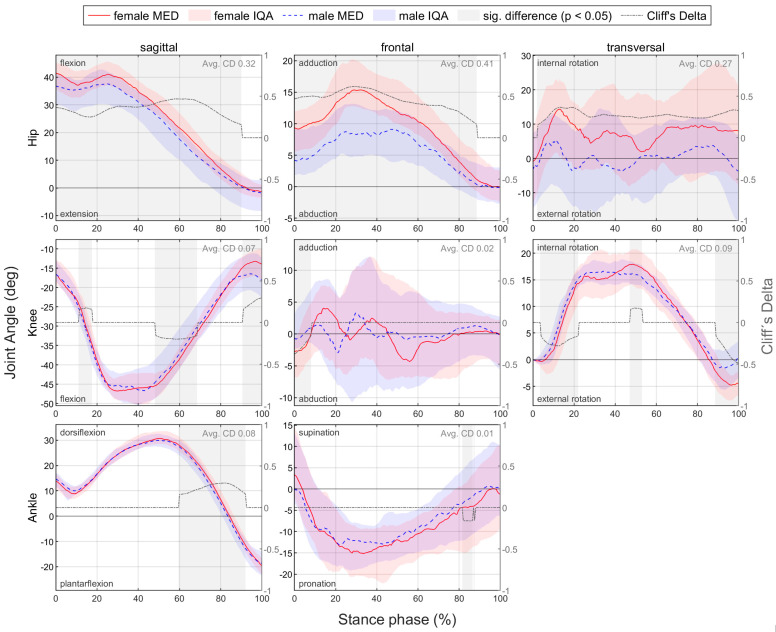
Gender differences in joint angles when running with shoe S5 at 10 km/h; MED = median; IQA = interquartile area.

Figure 11 summarises notable gender differences in joint angles with standard running shoes. Figure A1 depicts joint angles vs. time. Gender differences are most evident in the hip joint data. The female hip joint is more flexed, adducted and internally rotated almost throughout the entire stance phase. The female knee is less flexed and more externally rotated at the end of the stance phase. No gender differences are evident in the ankle joint, particularly in the frontal plane. Joint angle parameters are listed in Table A1. Hip flexion and hip adduction angles at foot strike and midstance as well as the peak angles are greater in women. Except for the sagittal plane at individual speed, women show a larger hip range of motion in all planes. In the sagittal plane, females also show a larger range of motion in the knee and ankle. Only at foot strike do female runners demonstrate a more pronounced knee abduction than male runners.

#### 3.2.3. Gender Differences in Joint Angular Velocities with Standard Running Shoes

**Figure 12:**
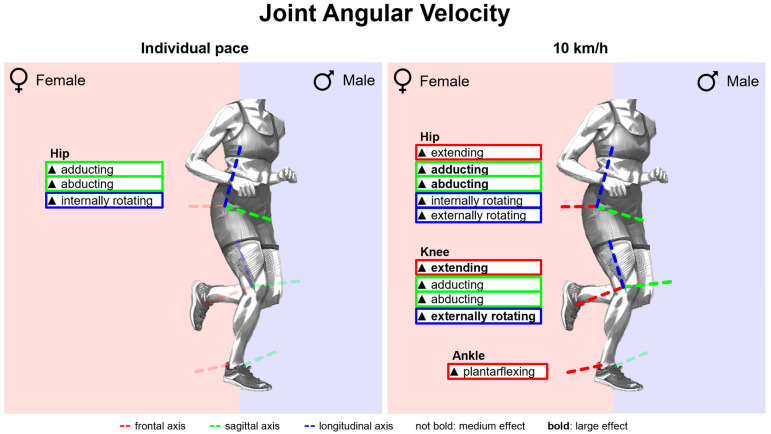
Gender differences in peak joint angular velocities with standard running shoes at the participants’ individual training pace and a running speed of 10 km/h; only significant differences with at least a medium effect size are presented and assigned to the gender with the higher value.

**Figure A2:**
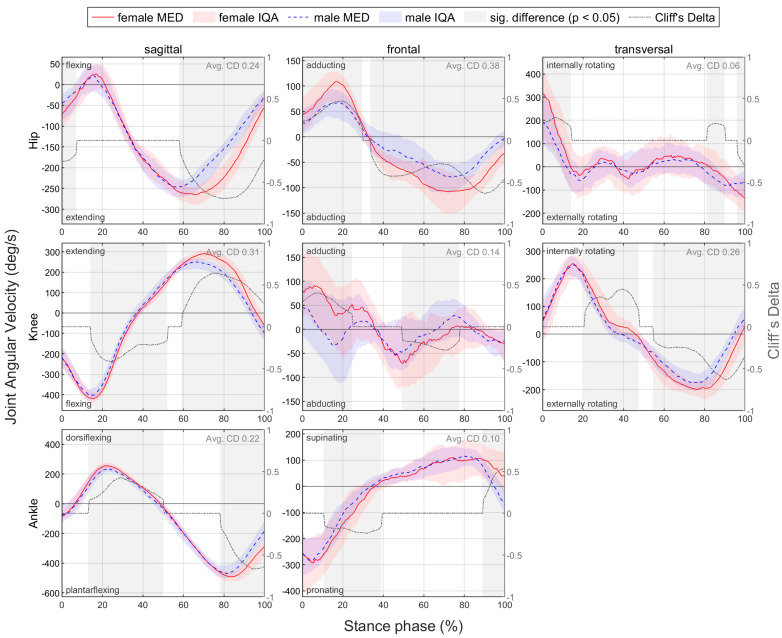
Gender differences in joint angular velocities when running with a standard running shoe (S5, Puma Velocity 2) at 10 km/h; MED = median; IQA = interquartile area.

Figure 12 summarises notable gender differences in joint angular velocities with standard running shoes. Figure A2 shows joint angular velocity vs. time. Women exhibit higher angular velocities in the frontal plane of the hip and sagittal plane of the knee over the entire stance phase and occasionally for the other joints and planes. In particular, shortly after foot strike, female runners show higher joint angular velocities for hip adduction and shortly before toe-off for hip extension, hip abduction, plantar flexion and supination. Peak joint angular velocities are listed in Table A2. Except for hip flexion, knee internal rotation and ankle supination, all peak joint angular velocities are higher in female runners at 10 km/h, with particularly strong differences for hip adduction, hip abduction, knee extension and knee external rotation. At individual speed, the differences are reduced, cancelled out or even reversed. Apart from hip extension, however, female runners also exhibit higher peak hip angular velocities.

#### 3.2.4. Gender Differences in External Joint Moments and Angular Impulses with Standard Running Shoes

**Figure 13:**
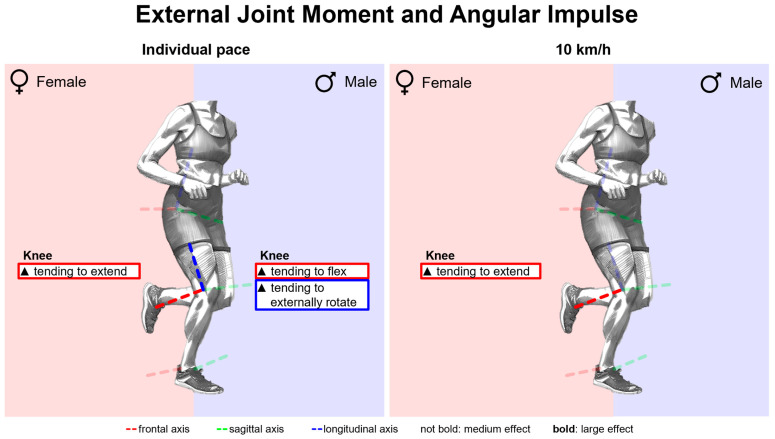
Gender differences in external peak joint moments and angular impulses with standard running shoes at the participants’ individual training pace and a running speed of 10 km/h; only significant differences with at least a medium effect size are presented and assigned to the gender with the higher value; peak moments and angular impulses are combined in a way that only the parameter with the higher effect size was considered.

**Figure A3:**
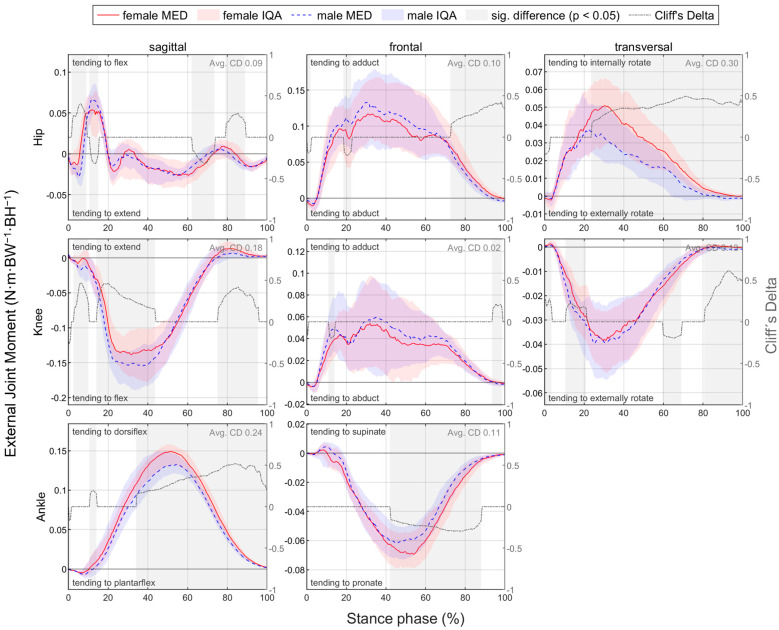
Gender differences in external joint moments when running with a standard running shoe (S5, Puma Velocity 2) at 10 km/h; MED = median; IQA = interquartile area; BW = body weight in N; BH = body height in m.

Figure 13 summarises notable gender differences in external joint moments and angular impulses with standard running shoes. Figure A3 shows external joint moment vs. time. Female runners experience a lower external knee flexion moment in the first 40% of the stance phase and a greater external knee extension moment in the last 20%. In the second half of the stance phase, women exhibit greater external hip rotation, dorsiflexion, and pronation moments. Peak joint moments are listed in Table A3. The greatest gender differences are found in the knee joint. Female runners show a lower external knee flexion moment but a greater external knee extension moment. Joint angular impulses are presented in Table A4. Here as well, female runners exhibit a lower external knee flexion angular impulse and a larger external knee extension angular impulse. They also show a larger external angular impulse for hip and knee internal rotation.

#### 3.2.5. Gender Differences in Joint Power and Work with Standard Running Shoes

**Figure 14:**
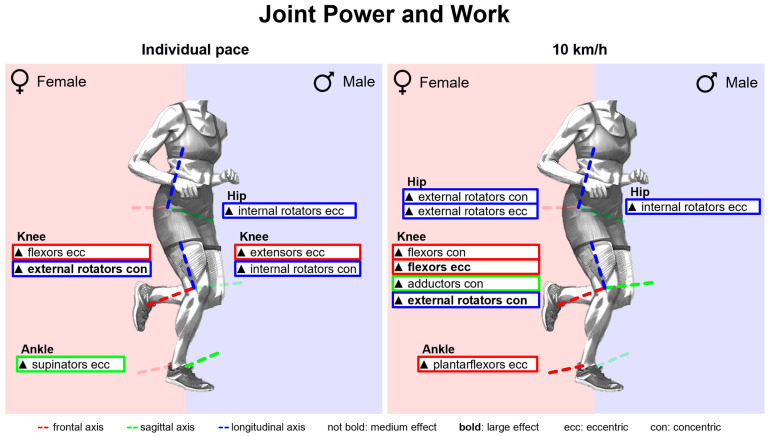
Gender differences in peak joint power and joint work with standard running shoes at the participants’ individual training pace and a running speed of 10 km/h; only significant differences with at least a medium effect size are presented and assigned to the gender with the higher value; peak power and work are combined in a way that only the parameter with the higher effect size was considered.

**Figure A4:**
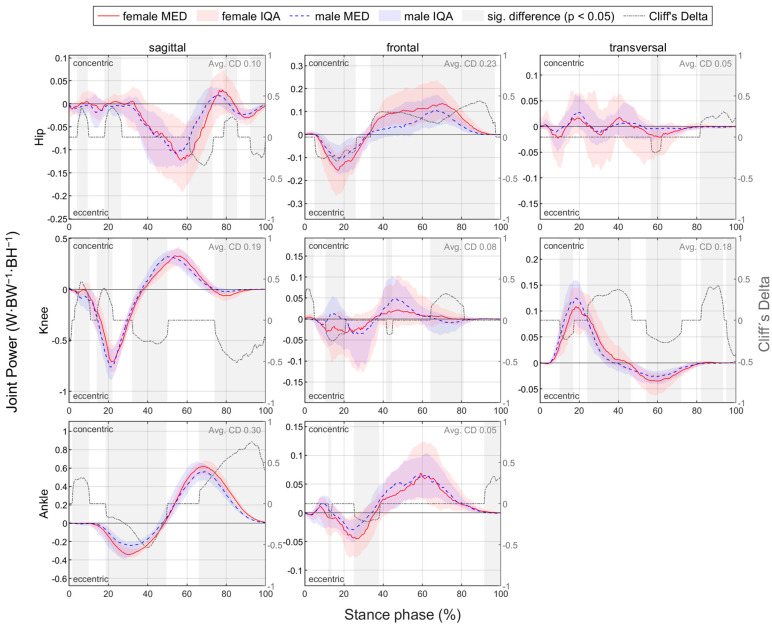
Gender differences in joint powers when running with a standard running shoe (S5, Puma Velocity 2) at 10 km/h; MED = median; IQA = interquartile area; BW = body weight in N; BH = body height in m.

Figure 14 summarises notable gender differences in joint power and work with standard running shoes. Figure A4 depicts joint power vs. time. Almost throughout the entire stance phase, female runners generate higher normalised joint power in the frontal plane of the hip and the sagittal plane of the ankle. In the last 20% of the stance phase, women demonstrate higher eccentric knee joint power in the sagittal plane. Peak joint power is listed in Table A5. Female runners exhibit higher eccentric and concentric peak power for knee flexion and higher eccentric peak power for supination in both speed conditions. At 10 km/h, they also show higher eccentric and concentric peak hip abduction, peak hip external rotation and peak plantarflexion power. Joint work is presented in Table A6. In both speed conditions, women generate more eccentric hip external rotation, knee flexion, plantar flexion and ankle supination work, but less eccentric knee extension work. At a uniform running speed of 10 km/h, women also show greater concentric work for hip extension, hip abduction, hip external rotation and plantar flexion.

**Figure 15:**
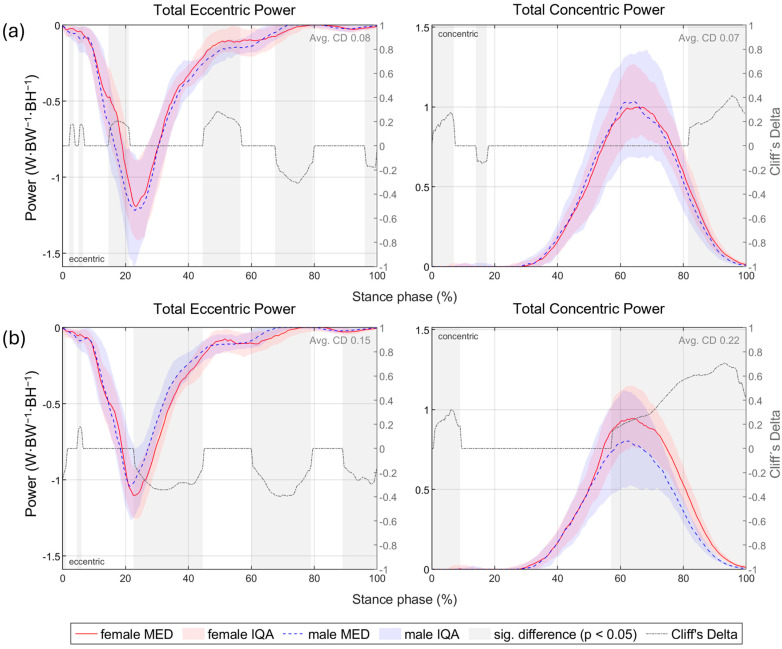
Gender differences in eccentric and concentric power totalled across all relevant muscle groups when running with a standard running shoe (S5, Puma Velocity 2) at (**a**) individual pace and (**b**) 10 km/h; MED = median; IQA = interquartile area; BW = body weight in N; BH = body height in m.

**Figure 16:**
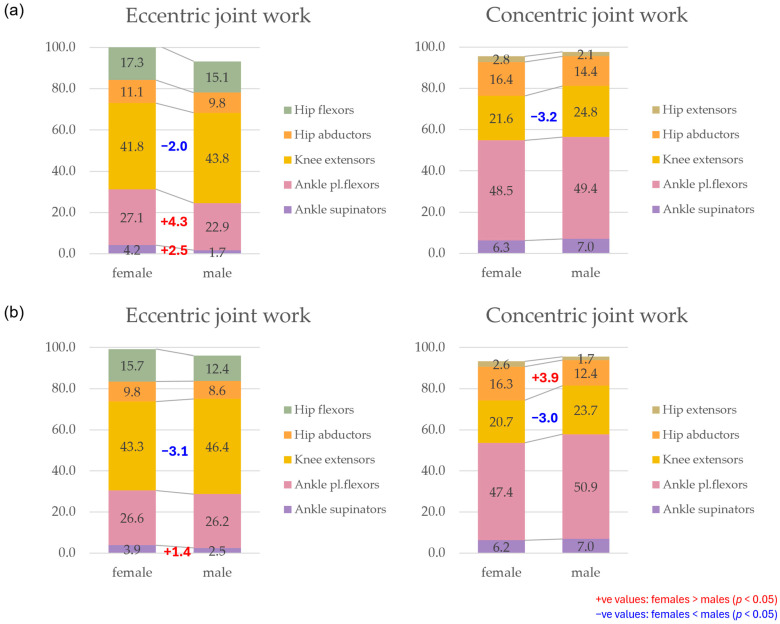
Gender differences in the distribution of total eccentric and concentric joint work when running with a standard running shoe (S5, Puma Velocity 2) at (**a**) individual pace and (**b**) 10 km/h; 100% = sum of eccentric/concentric work across all muscle groups; note that hip flexors are only involved in eccentric work and hip extensors in concentric work; since the medians are given, the percentages do not necessarily add up to 100%.

The total eccentric and concentric joint power at each point in time was determined as the sum across all joints that on average accounted for at least 2% of the total joint work (eccentric: hip flexors, hip abductors, knee extensors, plantar flexors and supinators; concentric: hip extensors, hip abductors, knee extensors, plantar flexors and supinators). At a uniform running speed of 10 km/h, female runners exhibited a higher total eccentric power between 20% and 40% and between 60% and 80% of the stance phase and a higher total concentric power from 60% onwards; however, there was no sustained gender difference at individual running pace (Figure 15). Furthermore, the eccentric and concentric total joint work and the proportion of the individual muscle groups are presented in Table 12 and visualised in Figure 16. Female runners generated more concentric work at 10 km/h and less eccentric work at individual speeds. Despite the significantly slower running speed of female runners, there is no difference in the total concentric joint work at individual running speed. Women and men therefore performed the same amount of propulsive joint work at their individual training speed. Overall, the knee extensors do most of the eccentric work and the plantar flexors most of the concentric work (40–50% each). The larger range of motion of female runners in the non-sagittal planes is also reflected in the distribution of joint work. In women, the knee extensors perform a smaller proportion of the total joint work, while the supinators contribute more to the eccentric work and the hip abductors to the concentric joint work.

**Figure 17:**
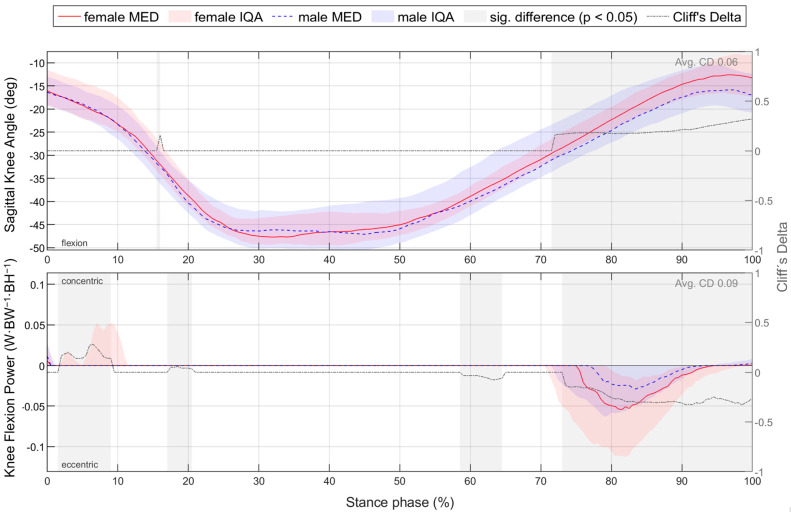
Gender differences in knee flexion angle and power when running with a standard running shoe (S5, Puma Velocity 2) at 10 km/h; MED = median; IQA = interquartile area; BW = body weight in N; BH = body height in m.

At the end and therefore within the propulsive second half of the stance phase, runners exhibit a negative knee flexion power. This eccentric knee flexion work is more pronounced in women, who are associated with a lower knee flexion angle at toe-off compared to men (Figure 17). As a result, female runners lose a larger proportion of the total concentric joint work required for propulsion, both in absolute and relative terms (Table 13). Together with the greater concentric work at uniform running speed, this may indicate a less energetically efficient running style in female runners. Due to the eccentric work of the knee flexors, women lose on average 1.50% and men 0.29% of the propulsive joint work at a running speed of 10 km/h, and 1.68% and 0.71%, respectively, at individual speeds.

#### 3.2.6. Gender Differences in Segment Angles with Standard Running Shoes

**Figure 18:**
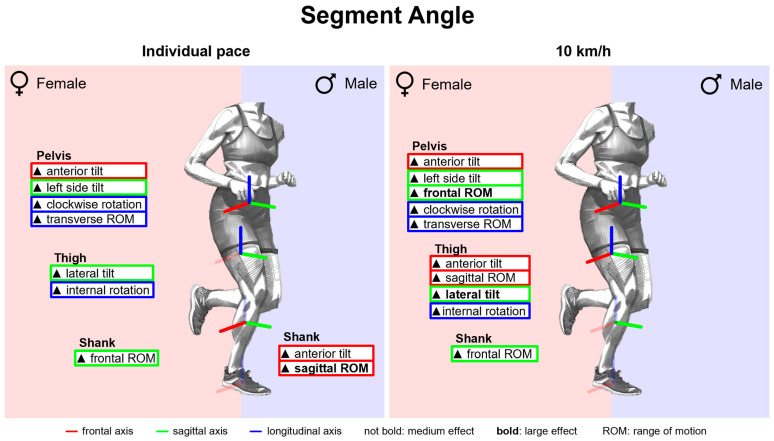
Gender differences in peak segment angles and ranges of motion with standard running shoes at the participants’ individual training pace and a running speed of 10 km/h; only significant differences with at least a medium effect size are presented and assigned to the gender with the higher value.

**Figure 19:**
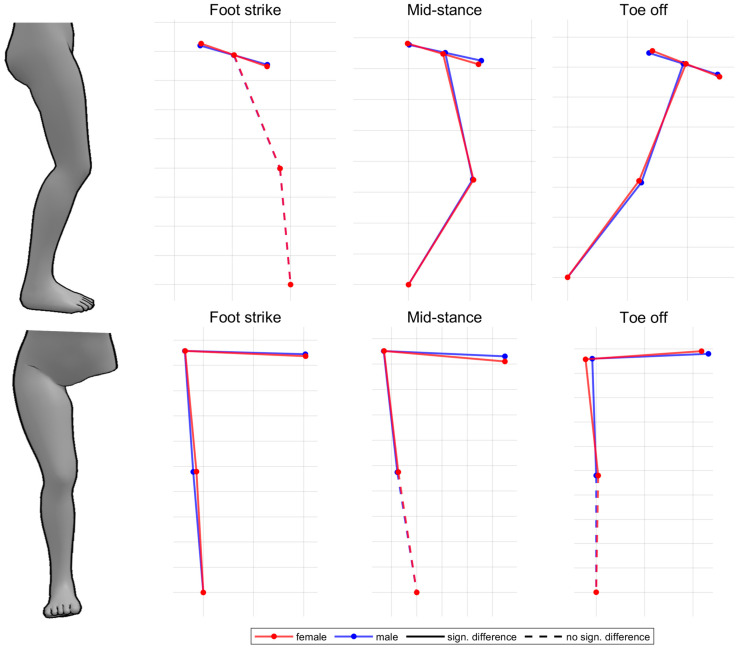
Visualisation of body segment orientations of female and male runners’ pelvis, right thigh and right shank in sagittal (**top**) and frontal (**bottom**) plane when running with standard running shoe (S5, Puma Velocity 2) at 10 km/h; ankles aligned.

**Figure A5:**
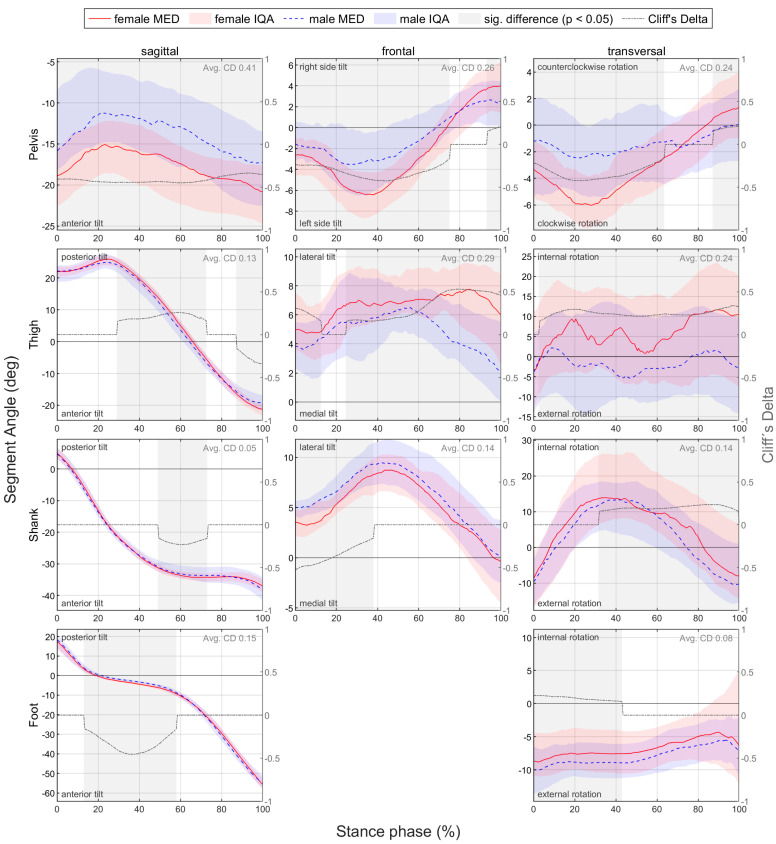
Gender differences in segment angles when running with a standard running shoe (S5, Puma Velocity 2) at 10 km/h; MED = median; IQA = interquartile area.

Figure 18 summarises notable gender differences in segment angles with standard running shoes. Figure A5 shows segment angles vs. time. Women demonstrate a stronger pelvic anterior tilt as well as a more pronounced lateral tilt and internal rotation of the thigh throughout almost the entire stance phase. The female thigh is tilted more anteriorly at toe-off, whereas there is no gender difference at foot strike. Together with the consistently more anteriorly tilted female pelvis, this results in a more flexed hip joint at foot strike and no gender difference at toe-off. In the frontal and transverse plane of the pelvis, on the other hand, the curves intersect at approx. 75% of the stance phase. Compared to males, the female pelvis is initially tilted more to the left and rotated clockwise, while at the end it is tilted more to the right and rotated counterclockwise. Both genders demonstrate a lateral tilting of the thigh in the first half of the stance phase. While men straighten their thigh back up to the initial position (at foot strike), women maintain a more laterally tilted thigh until toe-off. Therefore, the female hip is more adducted at foot strike with the greatest gender difference at 30% of the stance phase, while no gender difference is detected at toe-off. In the first half of the stance phase, the female foot is more anteriorly tilted and less externally rotated. Segment angle parameters are listed in Table A7. Females exhibit a larger range of motion in the frontal and transverse plane of the pelvis, in the transverse plane of the thigh and in the frontal plane of the shank. Only in the sagittal plane of the shank is the range of motion reduced compared to men. While the female shank is only at foot strike less laterally tilted, the thigh is more laterally tilted during the entire stance phase and especially at toe-off. The segment angles of female and male runners in the sagittal and frontal plane are visually compared in Figure 19.

#### 3.2.7. Gender Differences in Segment Angular Velocities with Standard Running Shoes

**Figure 20:**
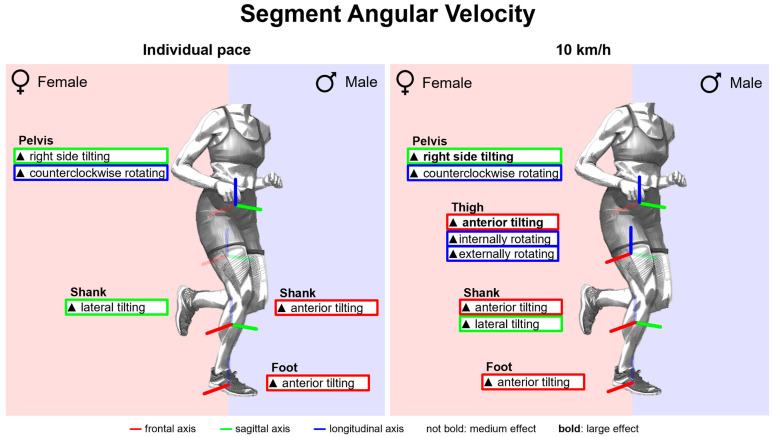
Gender differences in peak segment angular velocities with standard running shoes at the participants’ individual training pace and a running speed of 10 km/h; only significant differences with at least a medium effect size are presented and assigned to the gender with the higher value.

**Figure A6:**
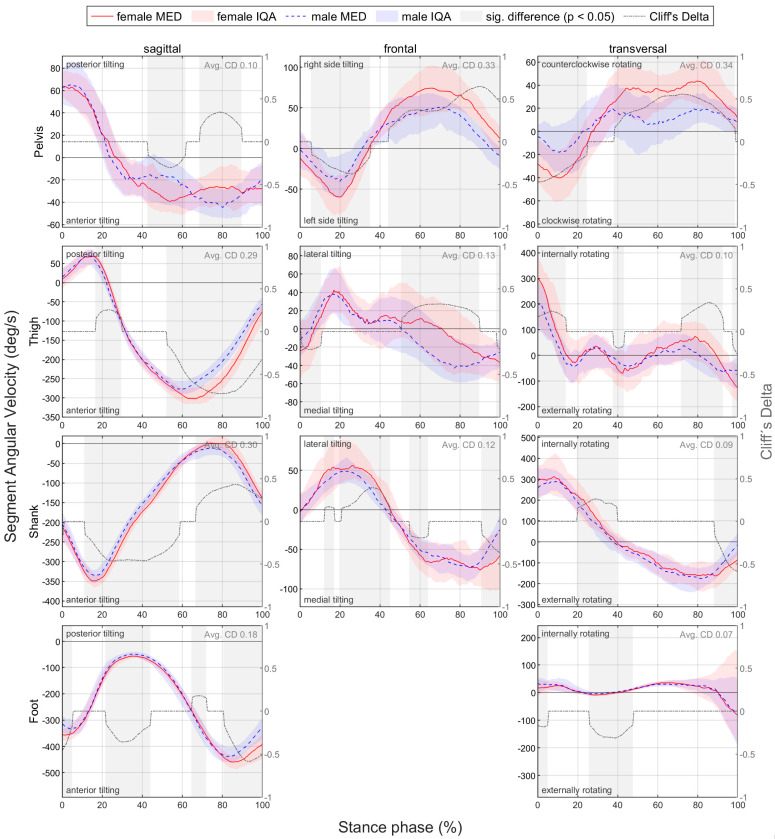
Gender differences in segment angular velocities when running with a standard running shoe (S5, Puma Velocity 2) at 10 km/h; MED = median; IQA = interquartile area.

Figure 20 summarises notable gender differences in segment angular velocities with standard running shoes. Figure A6 depicts segment angular velocity vs. time. The female pelvis exhibits almost continuously higher angular velocities in the frontal and transverse planes. In the sagittal plane, at the end of the stance phase, women show a faster thigh and foot, but a slower shank anterior tilting. In the second half of the stance phase, men demonstrate earlier and faster medial tilting of the thigh, which counteracts the initial lateral tilting during the first half of the stance phase. Peak segment angular velocities are listed in Table A8. At 10 km/h, women exhibit higher peak angular velocities in the frontal and transverse plane of the pelvis, transverse plane of the thigh and sagittal and frontal plane of the shank. In addition, the female foot and thigh tilt faster anteriorly when at their maximum. At individual speed, female runners also exhibit higher angular velocities in the frontal and transverse planes, but a slower anterior tilt of the foot and thigh.

#### 3.2.8. Gender Differences in Groups of Biomechanical Variables over Time with Standard Running Shoes

**Figure 21:**
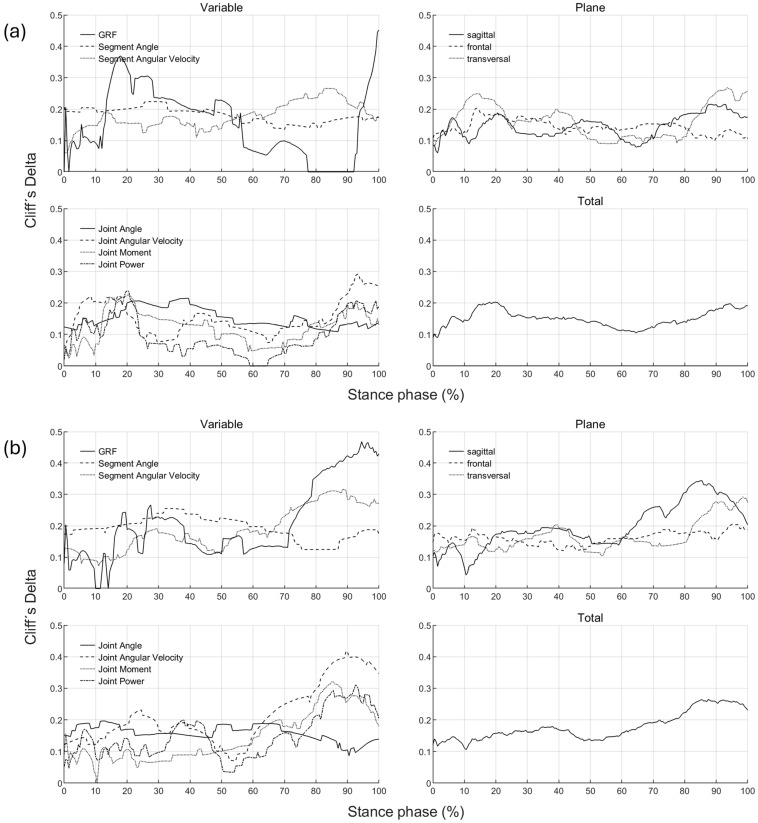
Effect size Cliff’s Delta for gender differences in biomechanical variables rectified and averaged over planes for each variable, over variables for each plane and over all variables and planes (total) when running with a standard running shoe (S5, Puma Velocity 2) at (**a**) individual pace and (**b**) 10 km/h; due to rectification, the direction cannot be determined, but the strength and duration of gender differences can.

Figure 21 shows the average effect size for gender differences over time grouped across variables, planes and in total for a running speed of 10 km/h and the individual running pace, respectively. While at 10 km/h, gender differences in joint and segment angles are uniform and tend to decrease at the end of the stance phase, the largest gender differences in GRF, joint angular velocity, joint moment, and joint power occur in the last 20% of the stance phase. Similarly, the greatest differences in sagittal and transverse planes, as well as averaged across all variables, are found at the end of the stance phase. At individual speed, considerably higher gender differences in the GRF occur between 10% and 50%, but also between 90% and 100% of the stance phase. The greatest gender differences can be seen at 20% and 95% of the stance phase. When comparing speed conditions, gender differences are overall and particularly at the end of the stance phase smaller at individual running speed compared to the uniform running speed of 10 km/h.

#### 3.2.9. Gender Differences in Timing of Ground Reaction Force and Joint Work with Standard Running Shoes

**Figure 22:**
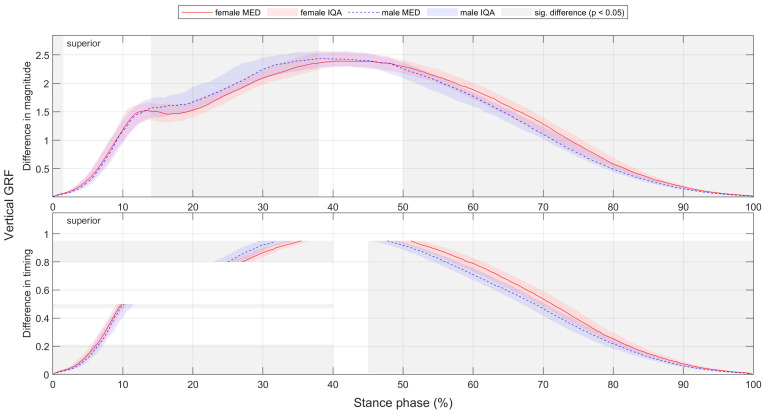
Gender differences in the magnitude of vertical ground reaction force (GRF) normalised to body weight (**top**) and in the timing of the normalised vertical GRF (**bottom**) when running with a standard running shoe (S5, Puma Velocity 2) at 10 km/h; analysis of the differences in timing is limited to the ranges with a monotonous curve; MED = median; IQA = interquartile area.

**Figure A7:**
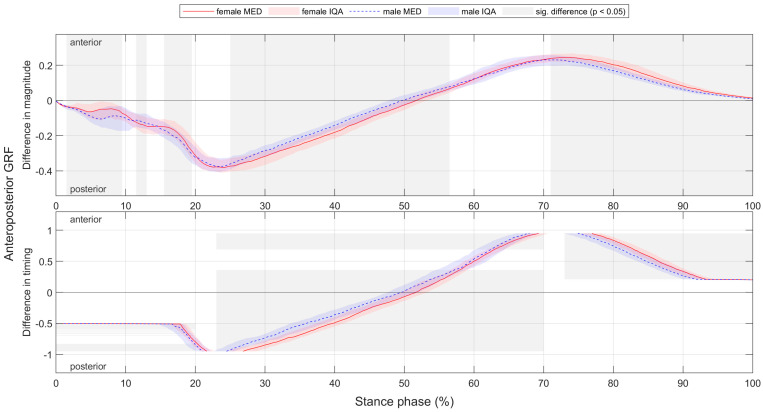
Gender differences in the magnitude of anteroposterior ground reaction force (GRF) normalised to body weight (top) and in the timing of the normalised anteroposterior GRF (bottom) when running with a standard running shoe (S5, Puma Velocity 2) at 10 km/h; analysis of the differences in timing is limited to the ranges with a monotonous curve; MED = median; IQA = interquartile area.

**Figure A8:**
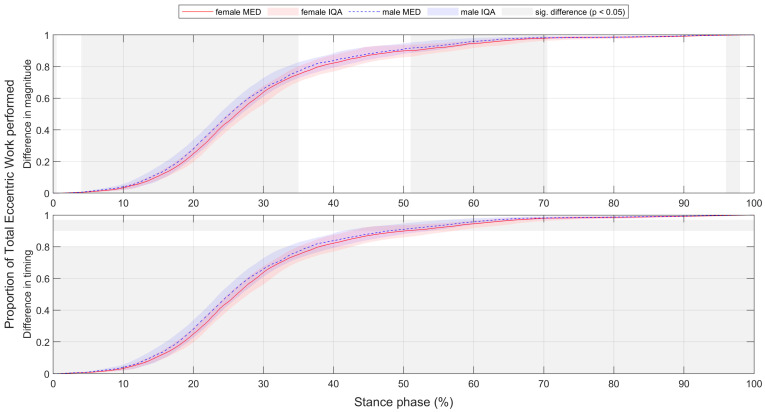
Gender differences in the proportion of total eccentric joint work performed in magnitude (top) and in timing (bottom) when running with a standard running shoe (S5, Puma Velocity 2) at 10 km/h; MED = median; IQA = interquartile area.

**Figure A9:**
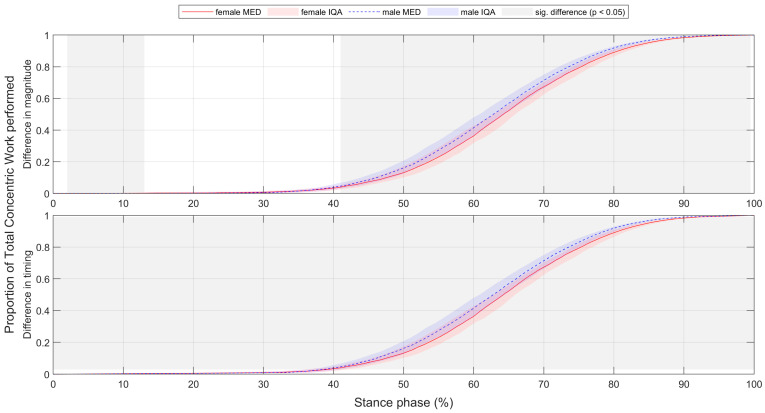
Gender differences in the proportion of total concentric joint work generated in magnitude (top) and in timing (bottom) when running with a standard running shoe (S5, Puma Velocity 2) at 10 km/h; MED = median; IQA = interquartile area.

So far, only the magnitude of the variables at each time point has been compared between female and male runners. In order to statistically analyse the timing in certain variables, the data were first normalised to stance phase duration and then to magnitude. Figure A7 and Figure 22 depict significant gender differences in anteroposterior and vertical GRF in both magnitude (grey vertical bars highlight periods with a significant gender difference in magnitude) and timing (grey horizontal bars highlight value ranges with a significant gender difference in timing). The female curves are shifted to the right compared to those of male runners. This indicates that peak forces occur earlier in men and later in women. Figure A8 and Figure A9 reveal gender differences in the proportion of total eccentric and concentric joint work generated at each point in time, both in terms of magnitude and time. The rightward shift in the female curve can be seen for the majority of eccentric and the entire concentric work. Compared to men, women achieve 80% of their total eccentric joint work (at f 38.0% vs. m 36.5% of the stance phase) and 80% of their total concentric joint work (at f 75.3% vs. m 73.5% of the stance phase) later in time. Compared to each other, male runners generate a larger proportion of their total concentric joint work between 40% and 60% and female runners between 70% and 90% of the stance phase. The time offset is more pronounced at a uniform running speed and is in accordance with the higher proportion of the deceleration phase in female runners. This time offset also potentially exists in other variables and should be considered when comparing gender. Therefore, in addition to analysing variable progressions, the additional calculation and comparison of extrema are mandatory.

### 3.3. Gender Differences in the Dynamics and Kinematics of Running Across Various Footwear Conditions

The distribution of the foot strike pattern among female and male runners is given in Table 14 for all footwear conditions. While women demonstrate a forefoot strike significantly more often than men when running barefoot, this ratio is reversed when running shoes are worn (S3-PS), with women almost always demonstrating a rearfoot strike (92–100%), while 12–18% of men stick to a forefoot or midfoot strike pattern. The average individual running pace was 10.74 (+/−1.05) km/h for females and 12.50 (+/−0.88) km/h for males. Running speed condition (individual pace vs. 10 km/h) does not affect foot strike pattern.

#### 3.3.1. Gender Differences in Ground Reaction Force Across Various Footwear Conditions

**Figure 23:**
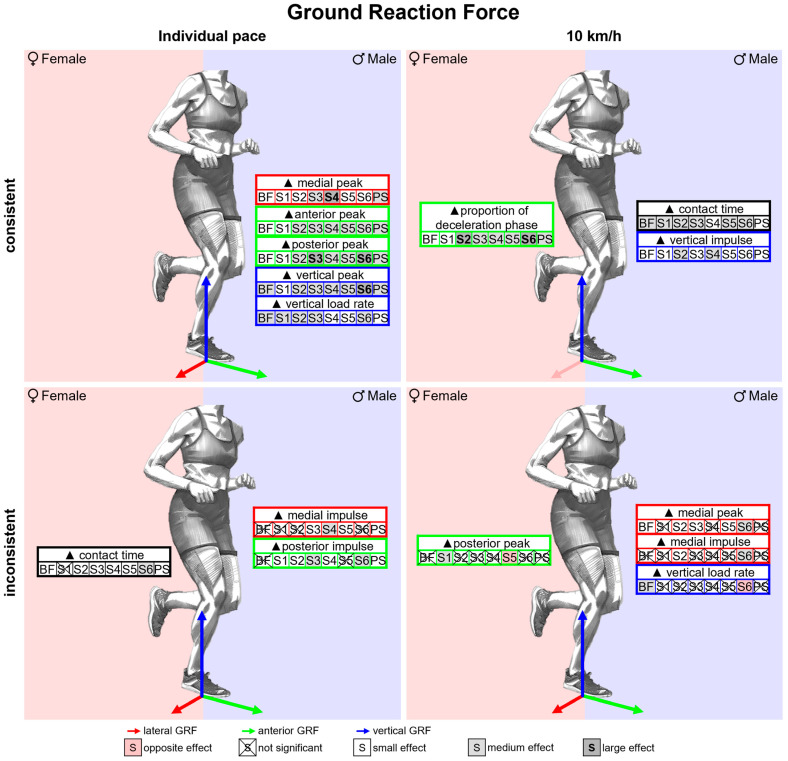
Gender differences in ground reaction force parameters across various footwear conditions at the participants’ individual training pace and a running speed of 10 km/h; only parameters with a significant difference and medium effect size in at least one footwear condition are presented and assigned to the gender with the on average higher value; consistent: significant and equidirectional gender difference across all footwear conditions; inconsistent: not significant or opposite gender difference in at least one footwear condition.

**Figure 24:**
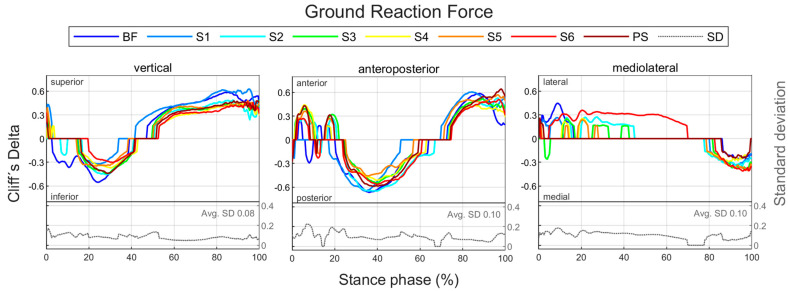
Effect size Cliff’s Delta for gender differences in ground reaction forces when running at 10 km/h across footwear conditions.

**Figure A10:**
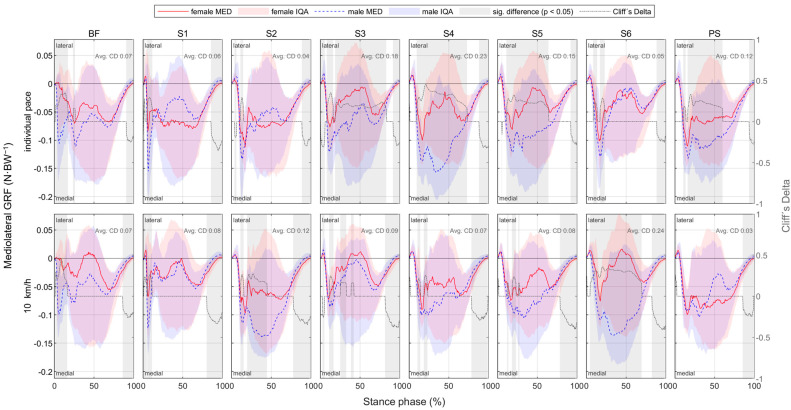
Gender differences in mediolateral ground reaction force (GRF).

**Figure A11:**
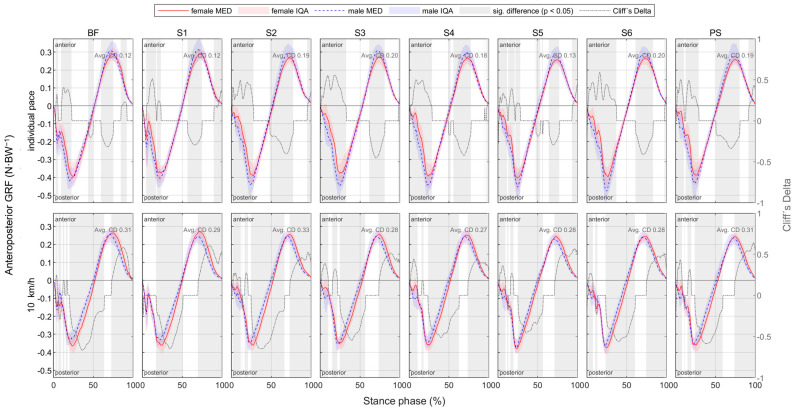
Gender differences in anteroposterior ground reaction force (GRF).

**Figure A12:**
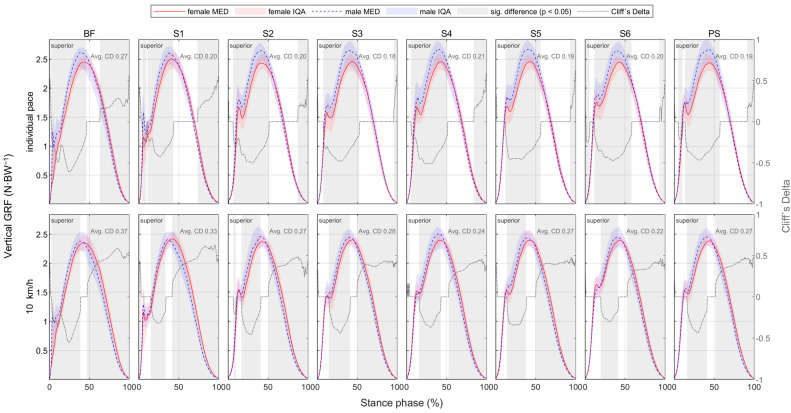
Gender differences in vertical ground reaction force (GRF).

Figure 23 summarises notable gender differences in ground reaction force across various footwear conditions. The curves of the GRF components of female and male runners are juxtaposed in Figure A10, Figure A11 and Figure A12. Figure 24 shows the effect size of gender differences for the single footwear conditions as well as their standard deviation over time. In contrast to the other footwear conditions, when wearing S6 (Puma Deviate Elite 2 with integrated carbon plate), women exhibited a significantly lower medial GRF than men for a large part of the stance phase. In the other two planes, the different footwear conditions produced similar gender differences with females showing higher posterior and lower vertical forces in the first half of the stance phase and higher anterior and vertical forces in the second half of the stance phase. The effect sizes of gender differences in GRF parameters are listed in Table 15. At individual speed, ground contact time for women is higher for almost all footwear conditions, but there is no difference in the proportion of the deceleration phase. In all footwear conditions, females exhibit lower peak forces in the vertical, anterior, posterior and medial directions as well as lower vertical loading rates. At a uniform running speed, women show shorter ground contact times and a larger proportion of the deceleration phase for all footwear conditions. With otherwise small or medium effects, the greatest differences are present in S2 and S6, each with a large effect. This might indicate that women run less efficiently than men, particularly with these footwear conditions. In addition, women have a lower vertical impulse across the board. Only two footwear conditions showed significant gender differences in the vertical loading rate, with women showing smaller values in barefoot running and higher values when running with S6. In addition, for S6, female runners exhibited lower peak medial and lateral forces and a lower medial impulse.

#### 3.3.2. Gender Differences in Joint Angles Across Various Footwear Conditions

**Figure 25:**
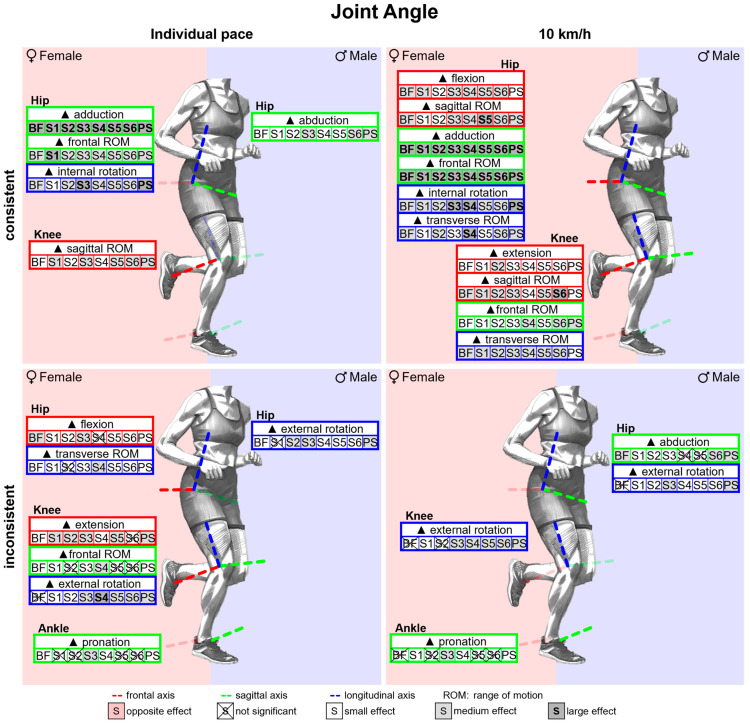
Gender differences in joint angles across various footwear conditions at the participants’ individual training pace and a running speed of 10 km/h; only parameters with a significant difference and medium effect size in at least one footwear condition are presented and assigned to the gender with the on average higher value; consistent: significant and equidirectional gender difference across all footwear conditions; inconsistent: not significant or opposite gender difference in at least one footwear condition.

**Figure A13:**
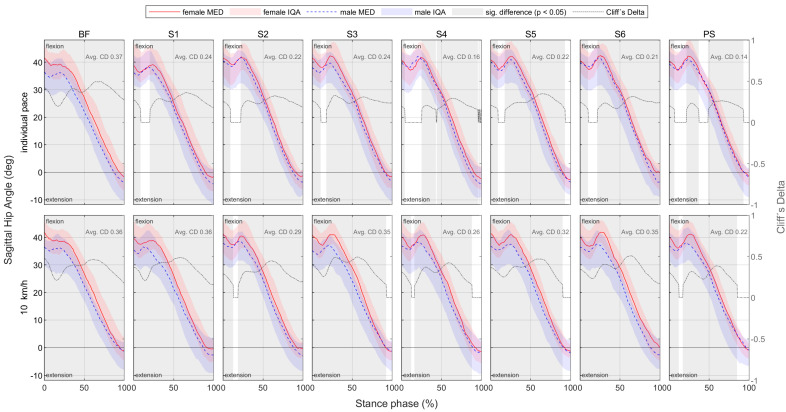
Gender differences in sagittal hip angle.

**Figure A14:**
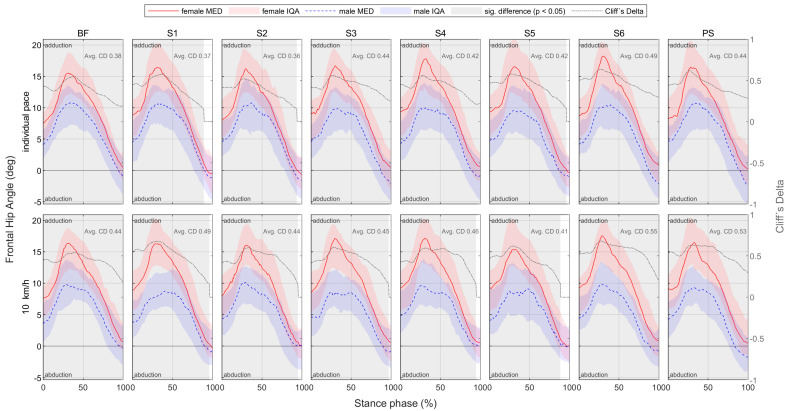
Gender differences in frontal hip angle.

**Figure A15:**
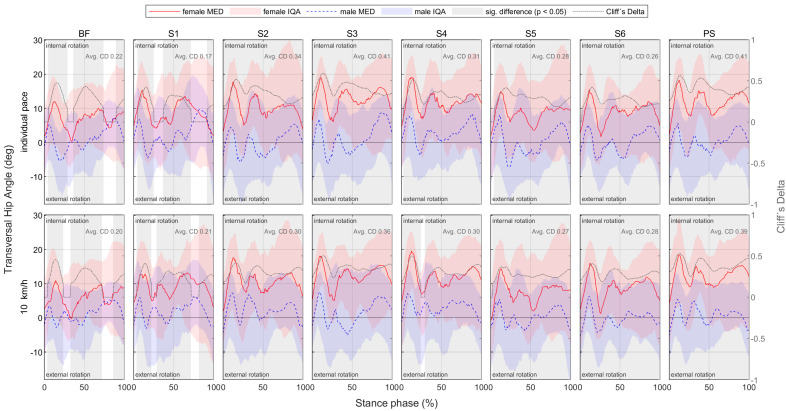
Gender differences in transverse hip angle.

**Figure A16:**
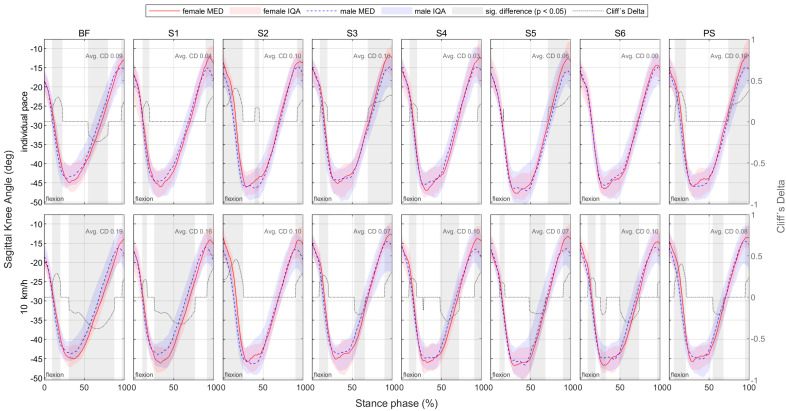
Gender differences in sagittal knee angle.

**Figure A17:**
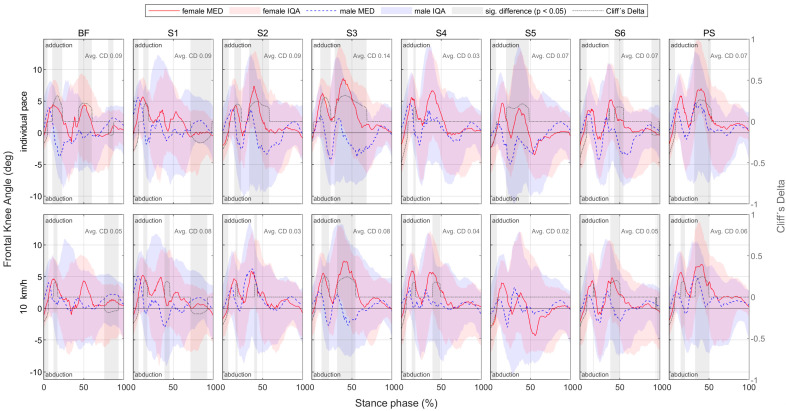
Gender differences in frontal knee angle.

**Figure A18:**
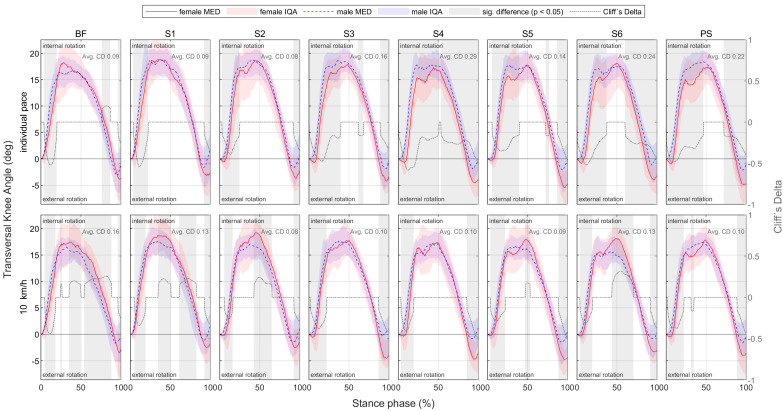
Gender differences in transverse knee angle.

**Figure A19:**
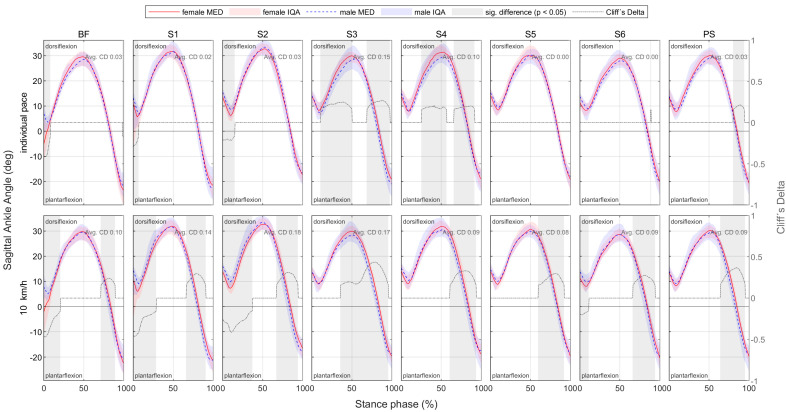
Gender differences in sagittal ankle angle.

**Figure A20:**
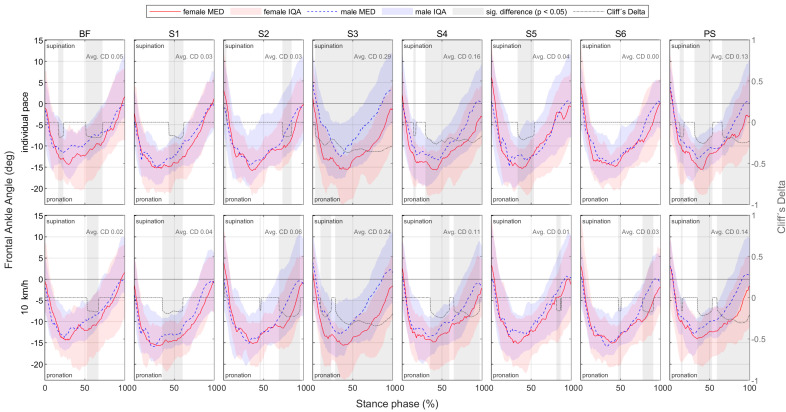
Gender differences in frontal ankle angle.

Figure 25 summarises notable gender differences in joint angles across various footwear conditions. The curves of the joint angles of female and male runners are juxtaposed in Figure A13, Figure A14, Figure A15, Figure A16, Figure A17, Figure A18, Figure A19 and Figure A20. Figure A75 shows the effect size of gender differences for the individual footwear conditions as well as their standard deviation over time. For all footwear conditions, the female hip is more flexed, adducted and internally rotated than the male hip throughout almost the entire ground contact time. For BF (barefoot) and S1 (Vivobarefoot Primus Lite III), the female knee is more flexed and internally rotated for a longer period compared to the other footwear conditions and the female ankle is more plantarflexed than the male ankle at foot strike. For S3 (Nike Free 5.0), women show earlier in stance phase a significantly greater dorsiflexion as well as a more pronounced ankle pronation over a longer period with a medium effect size. For S4 (Puma Liberate 2), PS (personal running shoe) and especially S3, women show significantly larger ankle pronation angles than men in both speed conditions (Figure A20). This appears to result from the contrasting reaction of both genders to each of the three footwear conditions. While females show a slightly higher ankle pronation compared to the other footwear conditions, males show smaller pronation angles for S3, S4 and PS. The effect sizes of gender differences in joint angle parameters are listed in Table A10. Across all footwear conditions, gender differences are greatest in the hip with a more flexed (small to medium effect), adducted and internally rotated (medium to large effect) female hip at the individual time points as well as at the peak values. In addition, women show larger ranges of motion in all planes of the hip and knee. Gender differences are more pronounced at uniform running speed than at individual running speed. The footwear condition has the greatest influence on gender differences in the sagittal knee and ankle angles at foot strike. Only for S2 (Joe Nimble Addict), the female knee is significantly less flexed. For BF, S1 and S2, women exhibited significantly greater plantar flexion angles at foot strike than men, while no gender differences are found for the other footwear conditions. For S3 (medium effect), S1, S4 and PS (small effect), female runners also show a larger peak pronation angle.

#### 3.3.3. Gender Differences in Joint Angular Velocities Across Various Footwear Conditions

**Figure 26:**
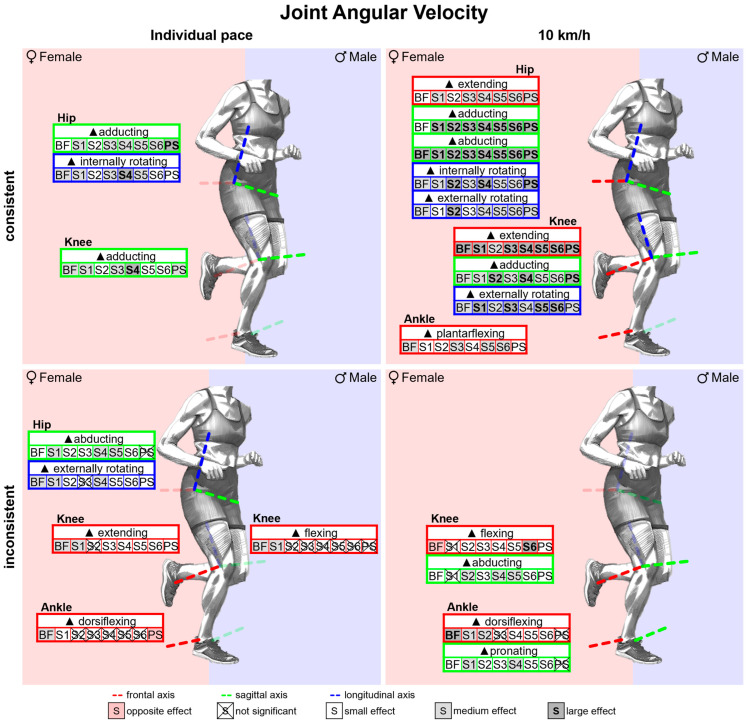
Gender differences in peak joint angular velocities across various footwear conditions at the participants’ individual training pace and a running speed of 10 km/h; only parameters with a significant difference and medium effect size in at least one footwear condition are presented and assigned to the gender with the on average higher value; consistent: significant and equidirectional gender difference across all footwear conditions; inconsistent: not significant or opposite gender difference in at least one footwear condition.

**Figure A21:**
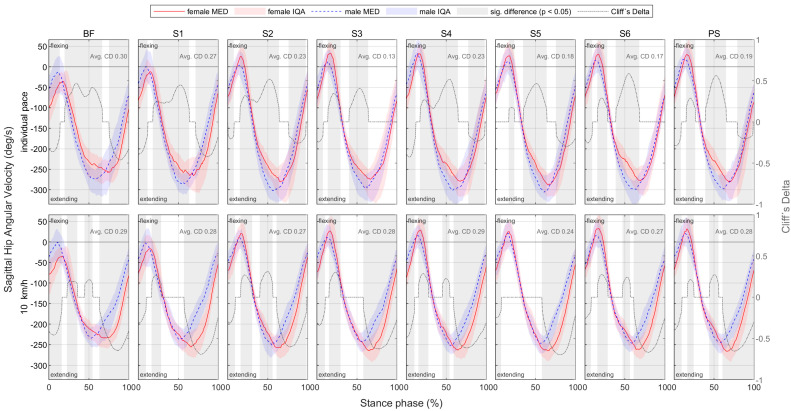
Gender differences in sagittal hip angular velocity.

**Figure A22:**
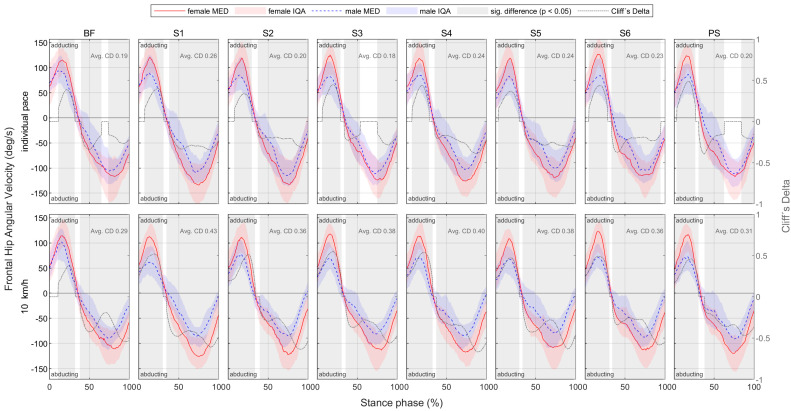
Gender differences in frontal hip angular velocity.

**Figure A23:**
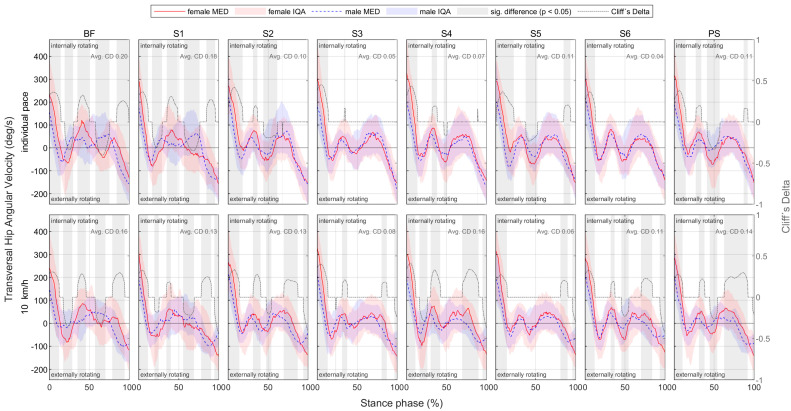
Gender differences in transverse hip angular velocity.

**Figure A24:**
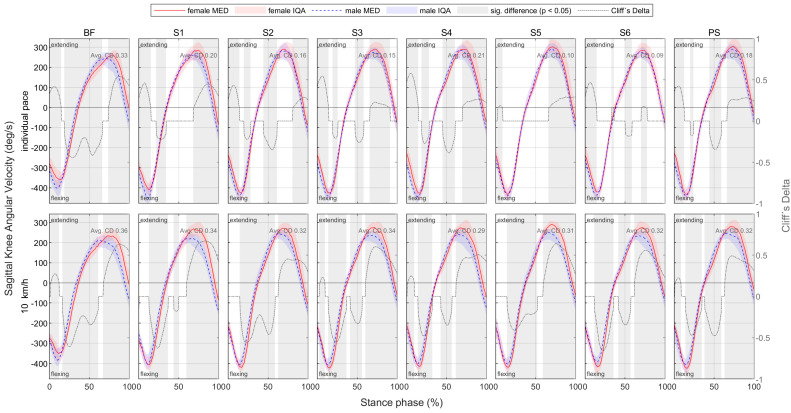
Gender differences in sagittal knee angular velocity.

**Figure A25:**
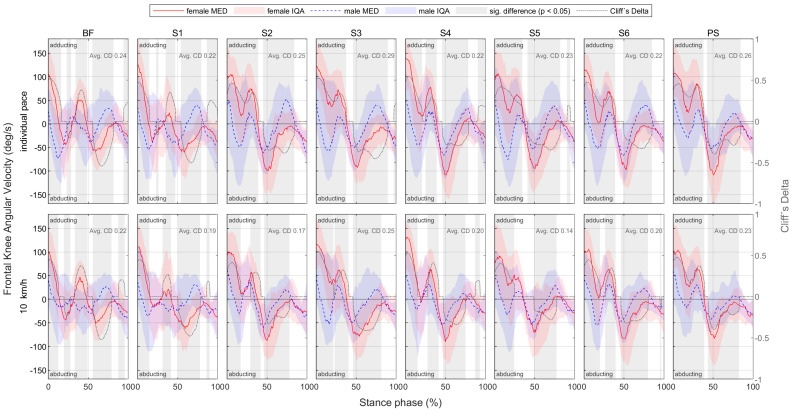
Gender differences in frontal knee angular velocity.

**Figure A26:**
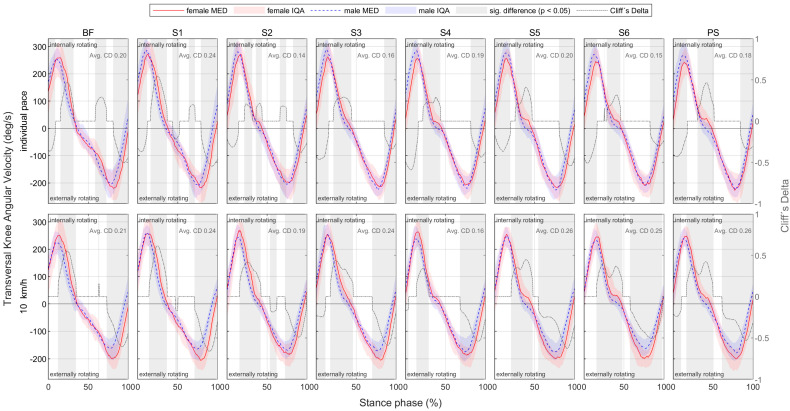
Gender differences in transverse knee angular velocity.

**Figure A27:**
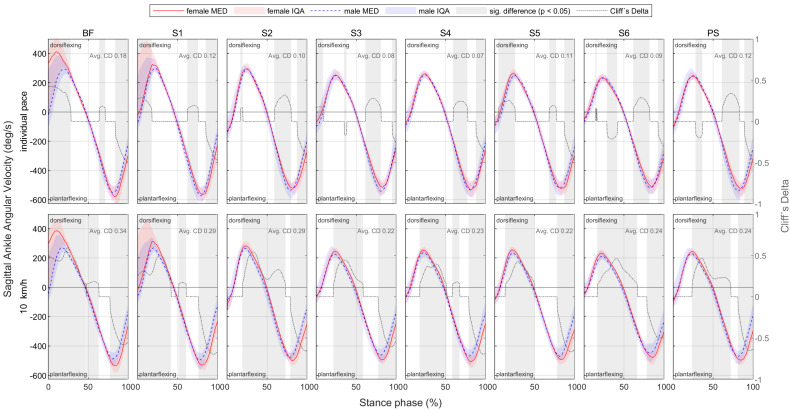
Gender differences in sagittal ankle angular velocity.

**Figure A28:**
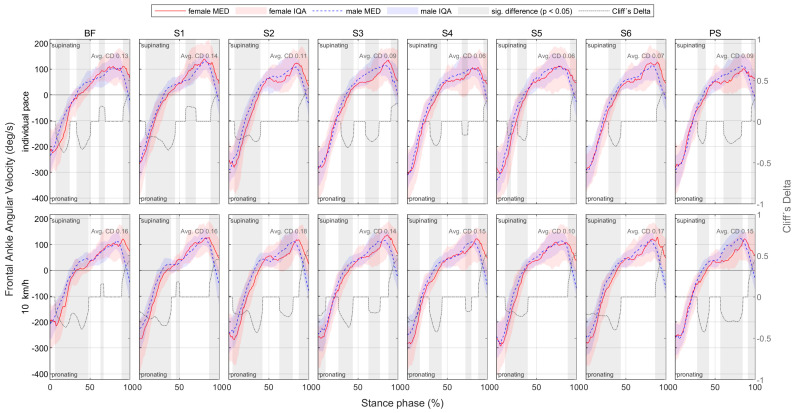
Gender differences in frontal ankle angular velocity.

Figure 26 summarises notable gender differences in joint angular velocities across various footwear conditions. The curves of the joint angular velocities of female and male runners are juxtaposed in Figure A21, Figure A22, Figure A23, Figure A24, Figure A25, Figure A26, Figure A27 and Figure A28. Figure A76 shows the effect size of gender differences for the individual footwear conditions as well as their standard deviation over time. Across all footwear conditions, women demonstrate higher velocities in hip and knee adduction at the beginning of the stance phase and in hip extension, hip abduction, knee extension, plantar flexion and supination at the end of the stance phase. The effect sizes of gender differences in peak joint angular velocities are listed in Table A11. At 10 km/h, women exhibit higher angular velocities than men in almost all footwear conditions (medium to large effect). With standard deviations between 0.15 and 0.18, hip and knee flexion velocity are the variables with the strongest influence of footwear condition on gender differences. For BF (barefoot), women showed slower knee flexion (small effect), while for S6 (Puma Deviate Elite 2), they showed faster knee flexion (large effect). For peak pronation velocity, there is no significant gender difference for PS (personal running shoe), while women showed larger values in all other footwear conditions with the largest effect found for S4 (Puma Liberate 2). Gender differences in the peak angular velocities are attenuated for individual velocity. In hip extension, knee flexion and knee internal rotation, female runners showed significantly lower values in part.

#### 3.3.4. Gender Differences in External Joint Moments and Angular Impulses Across Various Footwear Conditions

**Figure 27:**
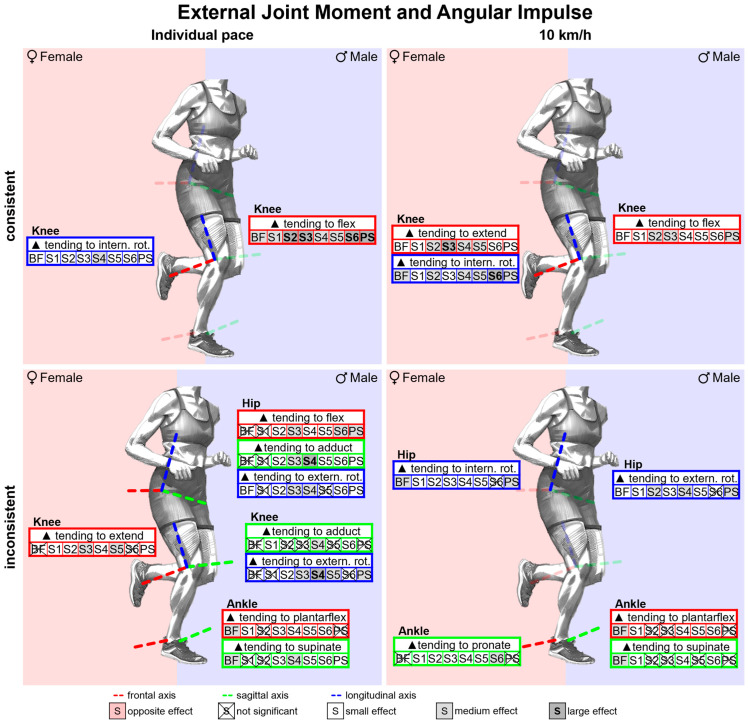
Gender differences in external peak joint moments and angular impulses across various footwear conditions at the participants’ individual training pace and a running speed of 10 km/h; only parameters with a significant difference and medium effect size in at least one footwear condition are presented and assigned to the gender with the on average higher value; consistent: significant and equidirectional gender difference across all footwear conditions; inconsistent: not significant or opposite gender difference in at least one footwear condition; peak moments and angular impulses are combined in a way that only the parameter with the higher effect size was considered.

**Figure A29:**
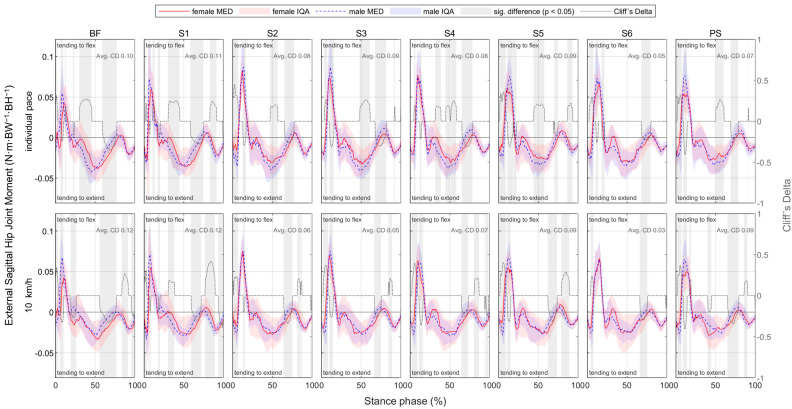
Gender differences in external sagittal hip joint moment.

**Figure A30:**
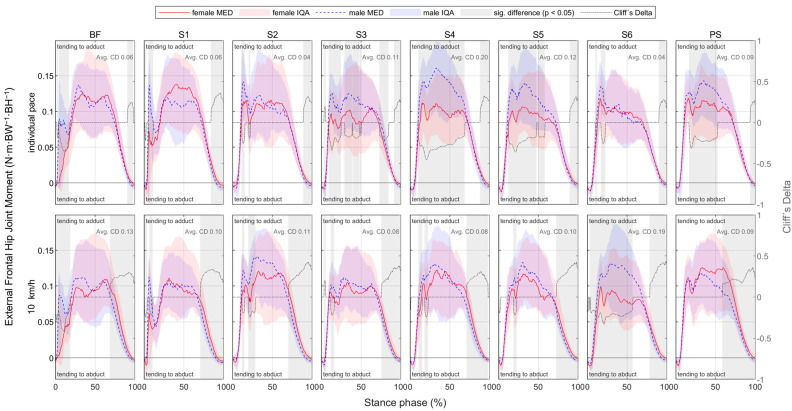
Gender differences in external frontal hip joint moment.

**Figure A31:**
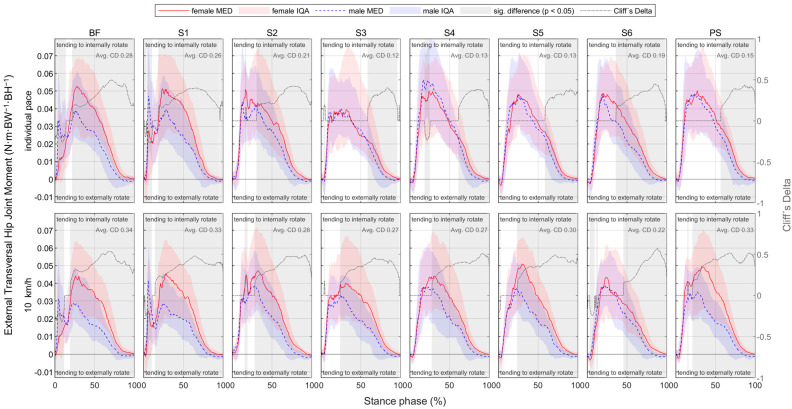
Gender differences in external transverse hip joint moment.

**Figure A32:**
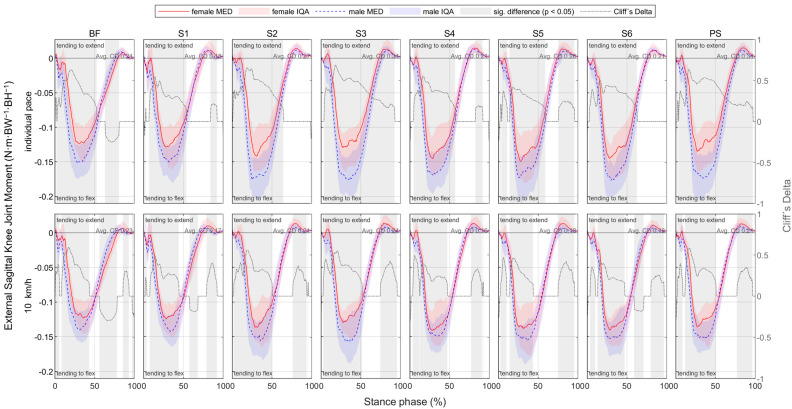
Gender differences in external sagittal knee joint moment.

**Figure A33:**
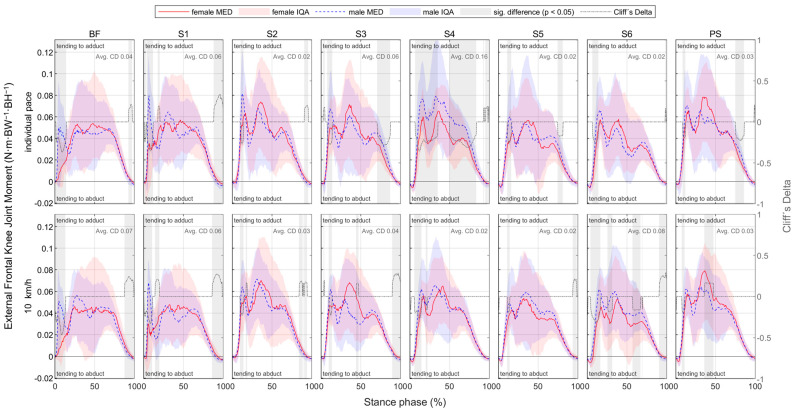
Gender differences in external frontal knee joint moment.

**Figure A34:**
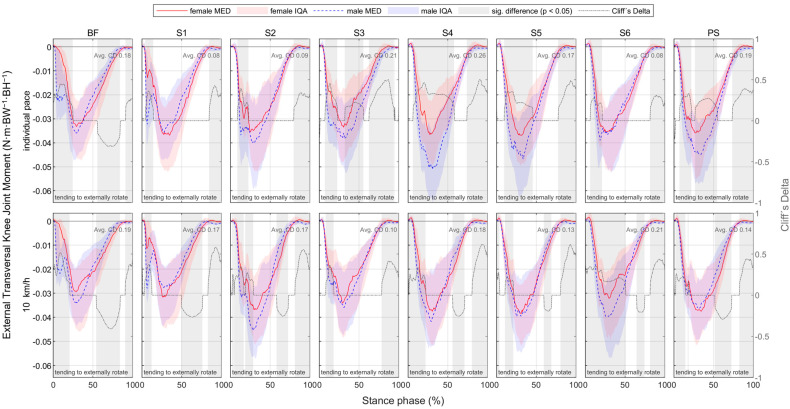
Gender differences in external transverse knee joint moment.

**Figure A35:**
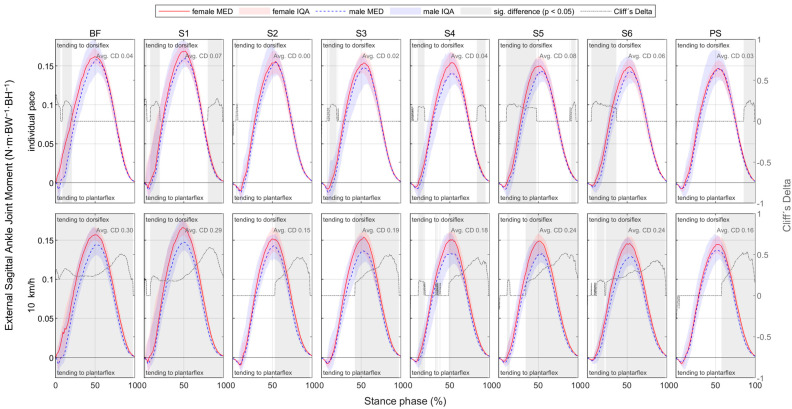
Gender differences in external sagittal ankle joint moment.

**Figure A36:**
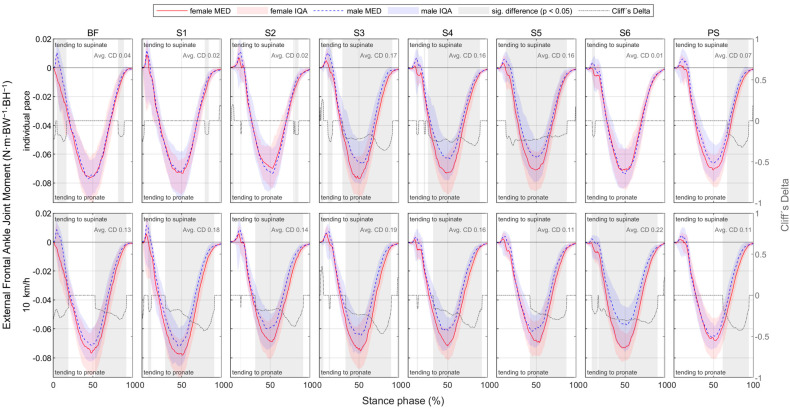
Gender differences in external frontal ankle joint moment.

Figure 27 summarises notable gender differences in external joint moments and angular impulses across various footwear conditions. The curves of the joint moments of female and male runners are juxtaposed in Figure A29, Figure A30, Figure A31, Figure A32, Figure A33, Figure A34, Figure A35 and Figure A36. Figure A77 shows the effect size of gender differences for the individual footwear conditions as well as their standard deviation over time. Female runners show a lower external knee flexion moment at the beginning of the stance phase across all footwear conditions, a greater external hip internal rotation moment throughout almost the entire stance phase and a greater external hip adduction, knee extension, dorsiflexion and pronation moment at the end of the stance phase. There are no gender differences in the frontal plane of the knee. S6 (Puma Deviate Elite 2) is the only condition in which women exhibit a lower hip adduction moment in the middle of the stance phase. In addition, compared to the other footwear conditions, female runners exhibit significantly higher hip external rotation moments than their male counterparts much later. Even though women exhibit greater external pronation moments in all footwear conditions, the duration for which this is the case differs significantly between footwear conditions. While the significant difference in S6 is present throughout the entire duration of ground contact, it is significantly reduced for S5 (Puma Velocity 2) and BF (barefoot) and shortest for PS (personal running shoe). The effect sizes of gender differences in peak joint moments and angular impulses are listed in Table A12 and Table A13. The greatest gender differences are found in the sagittal plane of the knee. Women show greater external knee extension and lower knee flexion moments. Female runners show greater external peak pronation moments for S1–S6, while the differences for BF and PS are not significant. In the frontal plane of the hip and knee, however, there are no constant gender differences in peak internal joint moments and joint angular impulses. In contrast to the other footwear conditions, there is no gender difference for the external hip internal rotation impulse for S6, while women show the greatest knee internal rotation impulse, and this is the only condition where female runners demonstrate a larger external ankle pronation impulse. A negative shift in the effect size is evident for individual running speed. Nevertheless, female runners still show larger angular impulses for knee internal rotation in all footwear conditions and for hip internal rotation, knee extension, dorsiflexion, and pronation in at least 50% of the footwear conditions.

#### 3.3.5. Gender Differences in Joint Power and Work Across Various Footwear Conditions

**Figure 28:**
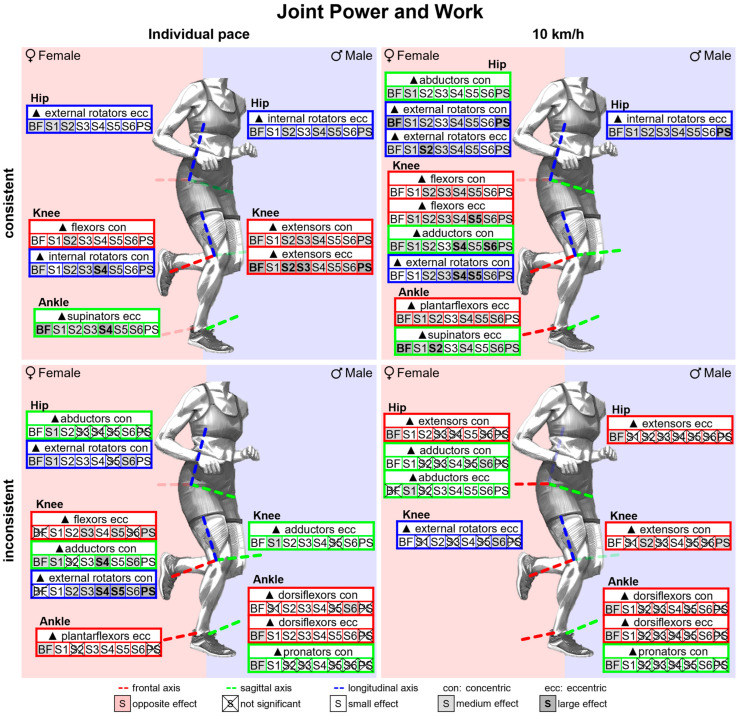
Gender differences in peak joint power and work across various footwear conditions at the participants’ individual training pace and a running speed of 10 km/h; only parameters with a significant difference and medium effect size in at least one footwear condition are presented and assigned to the gender with the on average higher value; consistent: significant and equidirectional gender difference across all footwear conditions; inconsistent: not significant or opposite gender difference in at least one footwear condition; peak power and work are combined in a way that only the parameter with the higher effect size was considered.

**Figure A37:**
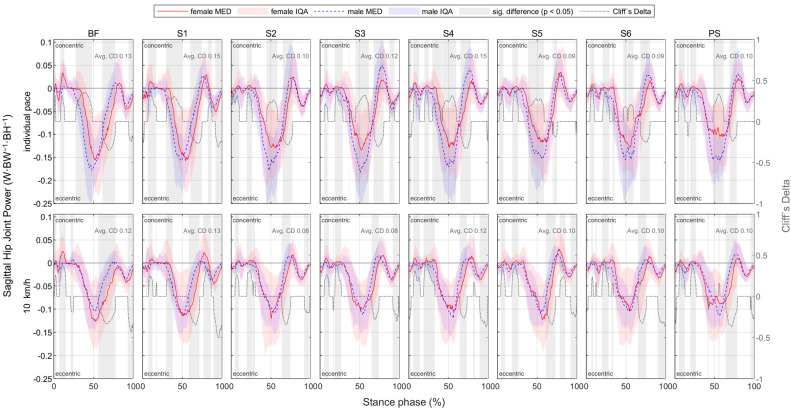
Gender differences in sagittal hip joint power.

**Figure A38:**
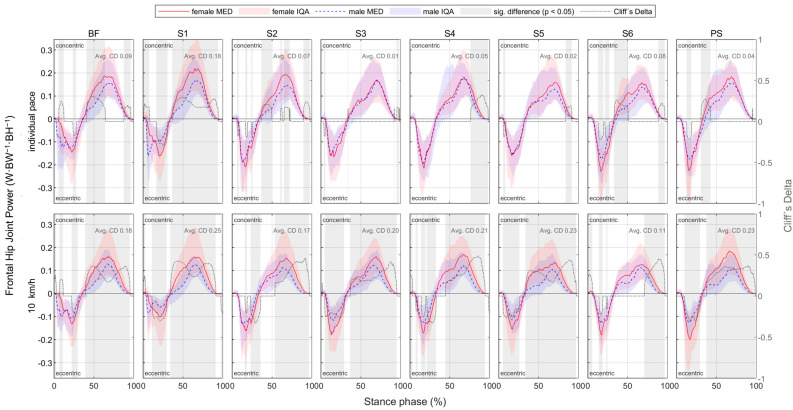
Gender differences in frontal hip joint power.

**Figure A39:**
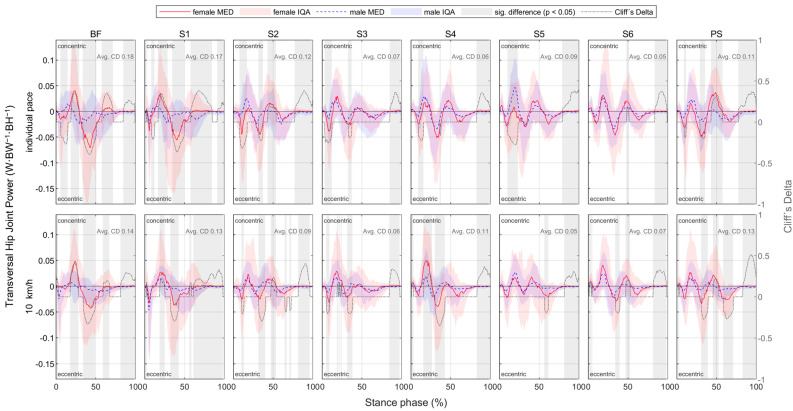
Gender differences in transverse hip joint power.

**Figure A40:**
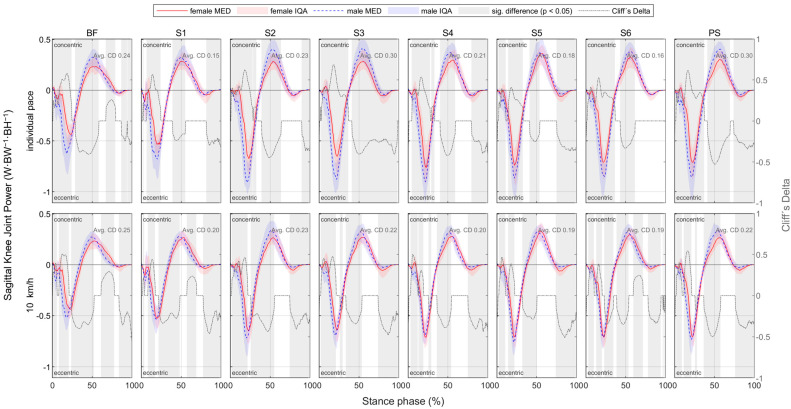
Gender differences in sagittal knee joint power.

**Figure A41:**
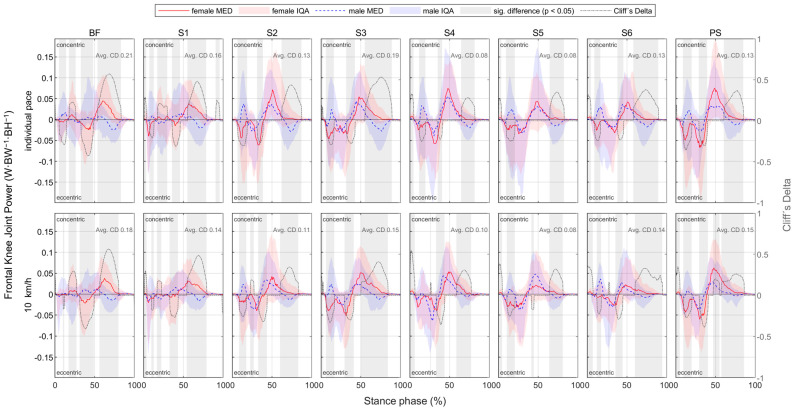
Gender differences in frontal knee joint power.

**Figure A42:**
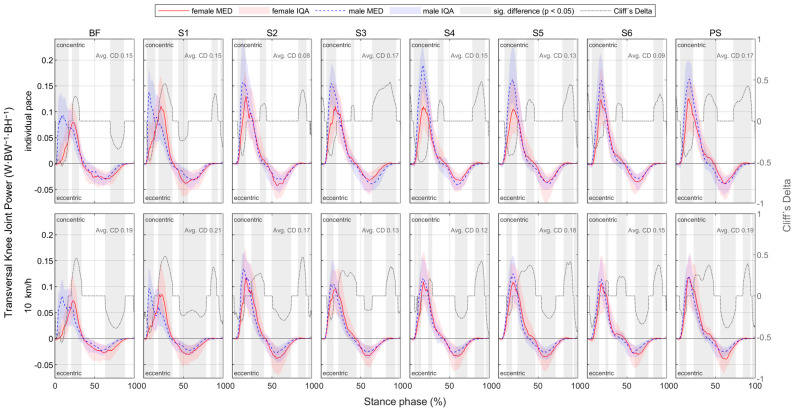
Gender differences in transverse knee joint power.

**Figure A43:**
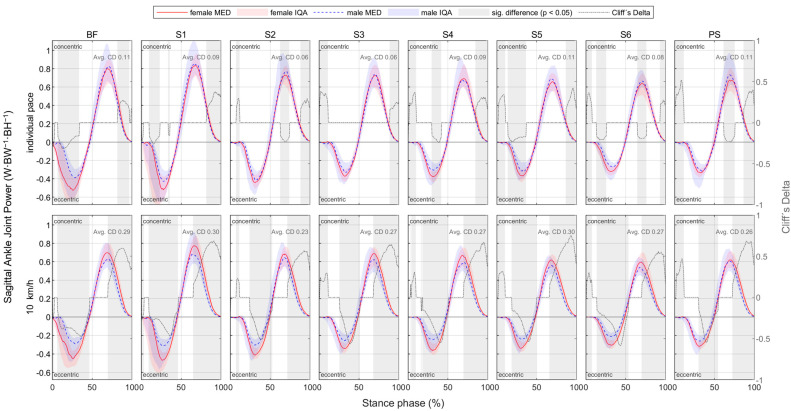
Gender differences in sagittal ankle joint power.

**Figure A44:**
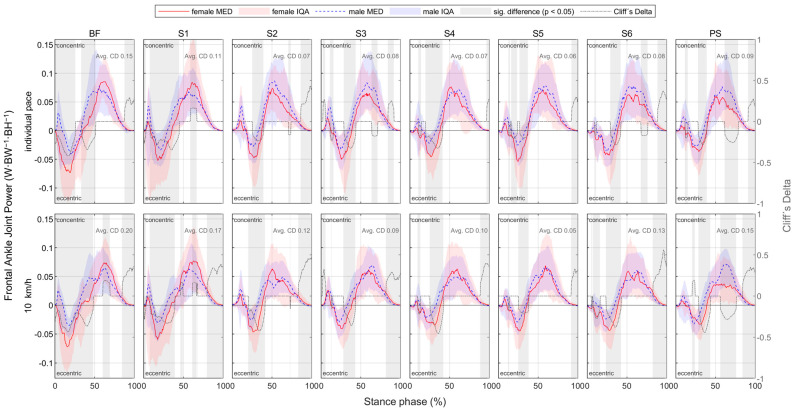
Gender differences in frontal ankle joint power.

Figure 28 summarises notable gender differences in joint power and work across various footwear conditions. The curves of the joint powers of female and male runners are juxtaposed in Figure A37, Figure A38, Figure A39, Figure A40, Figure A41, Figure A42, Figure A43 and Figure A44. Figure A78 shows the effect size of gender differences for the individual footwear conditions as well as their standard deviation over time. In all footwear conditions, women show a higher eccentric power at the beginning of the stance phase and a higher concentric power at the end of the stance phase in the hip abductors, plantar flexors and supinators. The differences in the eccentric power of the plantar flexors and supinators last longest for BF (barefoot) and S1 (Vivobarefoot Primus Lite III). While women show a higher concentric hip abduction power during the last 60% of the stance phase in most conditions, this difference only exists during the last 30% of the stance phase in S6 (Puma Deviate Elite 2). The effect sizes of gender differences in peak joint powers and joint work are listed in Table A14 and Table A15. At 10 km/h, female runners across all footwear conditions showed greater values in eccentric hip external rotation, knee flexion, plantar flexion and supination as well as in concentric hip abduction, hip external rotation and knee flexion. The effect of gender differences is smallest for the eccentric plantar flexion and supination work in S3 (Nike Free 5.0), S5 (Puma Velocity 2) and PS (personal running shoe) (small effects) and largest for BF (large effect). In contrast to the shoe conditions, female runners also perform less eccentric and more concentric hip extension work than men when running barefoot.

Figure A67 and Figure A68 show the total eccentric and concentric power totalled over all relevant muscle groups (muscle groups that on average accounted for at least 2% of the total joint work; eccentric: hip flexors, hip abductors, knee extensors, plantar flexors and supinators; concentric: hip extensors, hip abductors, knee extensors, plantar flexors and supinators) vs. time and Figure A69 and Figure A70 the cumulated eccentric and concentric total work vs. time. Table 16 presents gender differences in the amount and distribution of the total eccentric and concentric joint work. At 10 km/h, women tend to perform more concentric work and less eccentric work at individual speed. At a uniform running speed, women perform more eccentric and/or concentric joint work for BF (barefoot), S1 (Vivobarefoot Primus Lite III), S4 (Puma Liberate 2), S5 (Puma Velocity 2) and S6 (Puma Deviate Elite 2). For PS (personal running shoe), there are no gender differences in total joint work at 10 km/h, while women perform significantly less work at individual speed. During deceleration, female runners in both speed conditions utilise the knee extensors less and the plantar flexors and supinators more compared to men. At 10 km/h, hip abductors generally play a greater role in propulsion while knee extensors are used less. The only exception to this is S6, where no gender differences were observed in the distribution of concentric joint work. At individual speed, the hip abductors performed a larger proportion of the concentric work for BF, S1, S2 (Joe Nimble Addict), S6 and PS in women, while no gender differences were given for S3 (Nike Free 5.0), S4 and S5.

Women show a higher knee extension velocity at the end of the stance phase with a simultaneously higher external knee extension moment. This results in a more extended female knee at toe-off and more eccentric knee flexion work generated by female runners. Gender differences in the loss of propulsive work caused by the eccentric work of the knee flexors at the end of the stance phase are given in Table 17. For footwear conditions S3, S4, S5, S6 and PS, women thereby lose a higher proportion of their propulsive work than men. One potential explanation is that women are unable to counteract knee extension due to weaker ischiocrural muscles. However, BF (barefoot), S1 (Vivobarefoot Primus Lite III) and S2 (Joe Nimble Addict) do not show any gender differences in the loss of propulsive work, which is why a different movement pattern seems to be the more likely underlying cause.

#### 3.3.6. Gender Differences in Segment Angles Across Various Footwear Conditions

**Figure 29:**
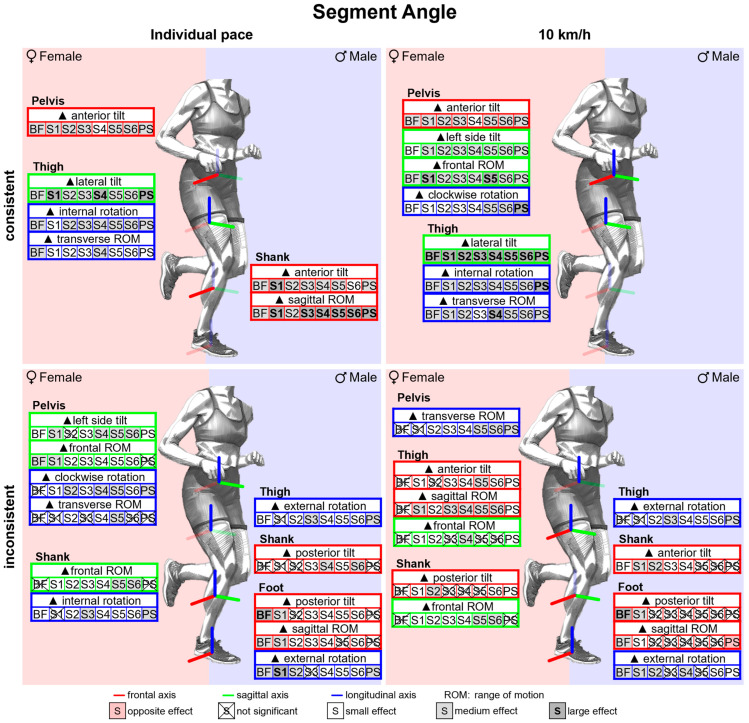
Gender differences in peak segment angles and ranges of motion across various footwear conditions at the participants’ individual training pace and a running speed of 10 km/h; only parameters with a significant difference and medium effect size in at least one footwear condition are presented and assigned to the gender with the on average higher value; consistent: significant and equidirectional gender difference across all footwear conditions; inconsistent: not significant or opposite gender difference in at least one footwear condition.

**Figure A45:**
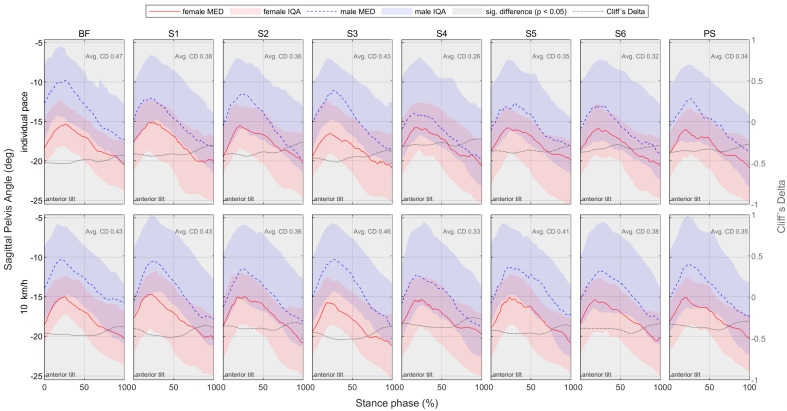
Gender differences in sagittal pelvis angle.

**Figure A46:**
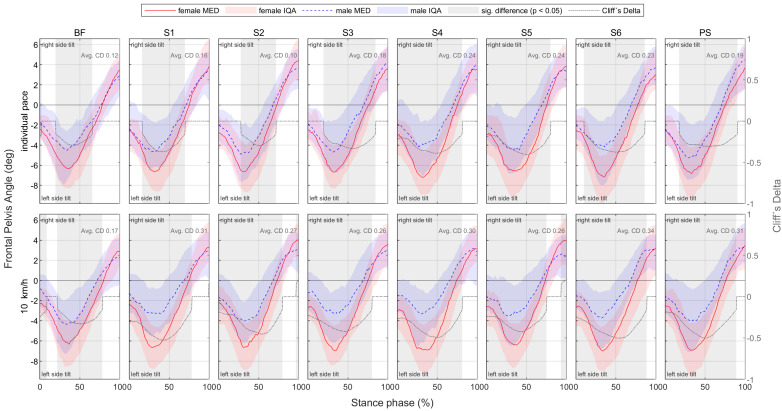
Gender differences in frontal pelvis angle.

**Figure A47:**
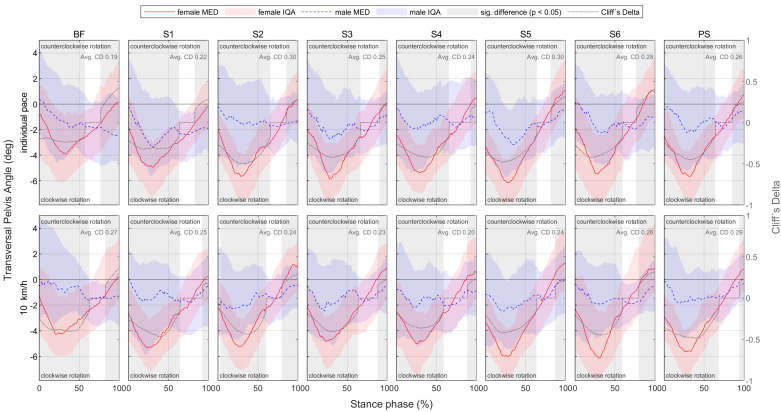
Gender differences in transverse pelvis angle.

**Figure A48:**
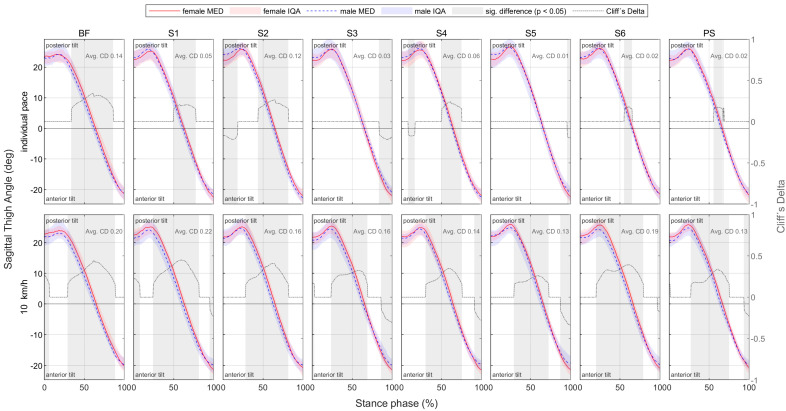
Gender differences in sagittal thigh angle.

**Figure A49:**
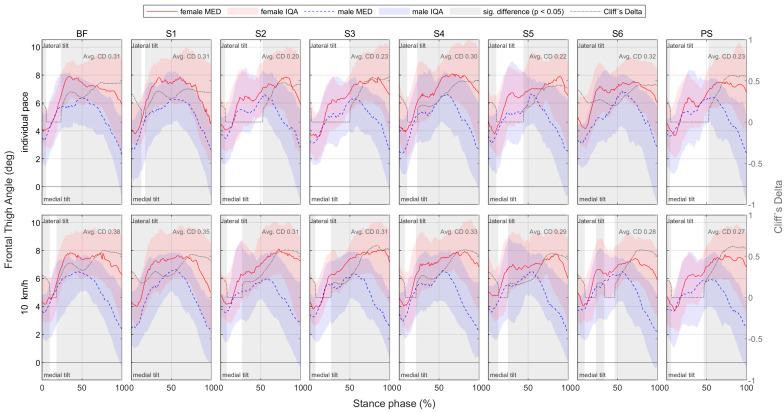
Gender differences in frontal thigh angle.

**Figure A50:**
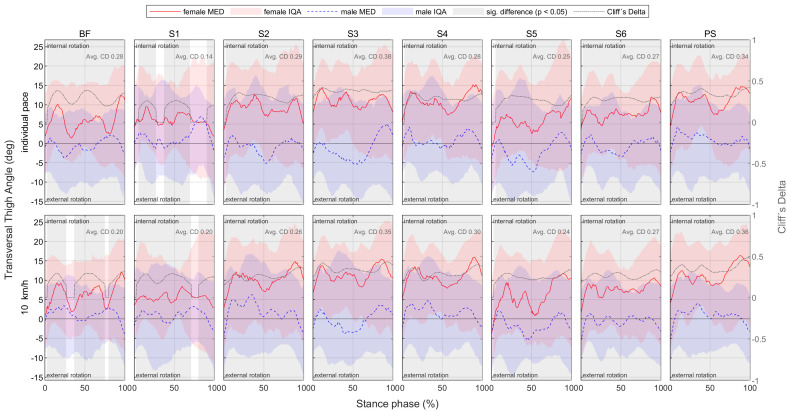
Gender differences in transverse thigh angle.

**Figure A51:**
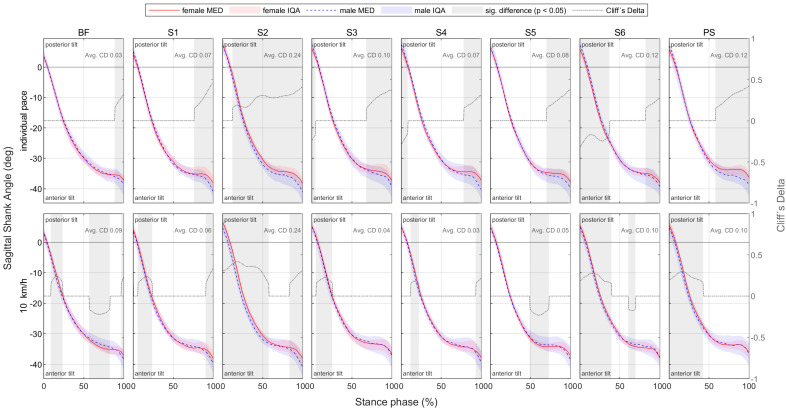
Gender differences in sagittal shank angle.

**Figure A52:**
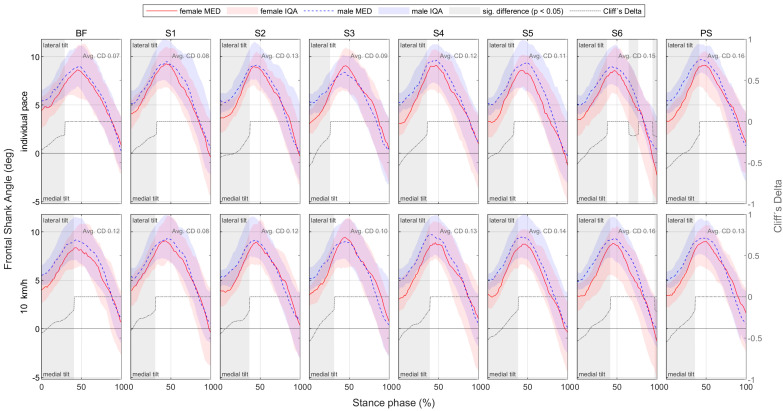
Gender differences in frontal shank angle.

**Figure A53:**
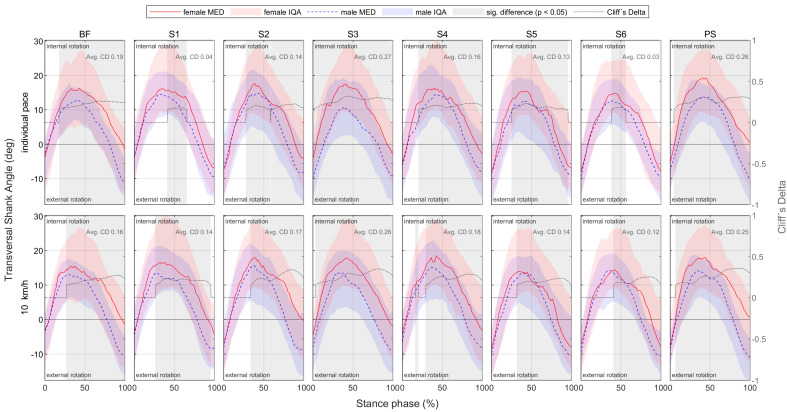
Gender differences in transverse shank angle.

**Figure A54:**
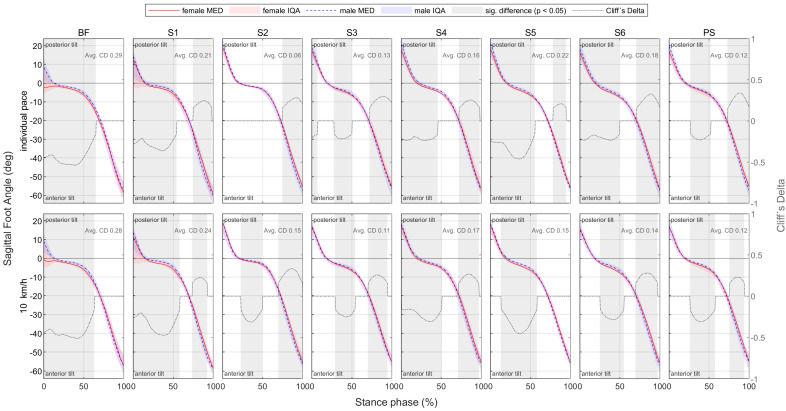
Gender differences in sagittal foot angle.

**Figure A55:**
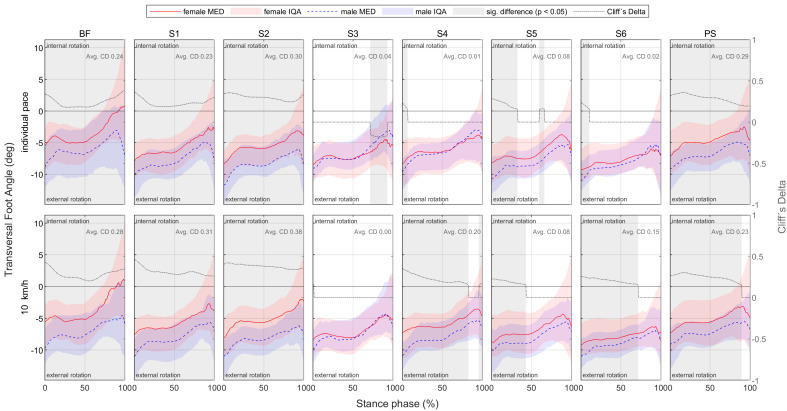
Gender differences in transverse foot angle.

Figure 29 summarises notable gender differences in segment angles across various footwear conditions. The curves of the segment angles of female and male runners are juxtaposed in Figure A45, Figure A46, Figure A47, Figure A48, Figure A49, Figure A50, Figure A51, Figure A52, Figure A53, Figure A54 and Figure A55. Figure A79 shows the effect size of gender differences for the individual footwear conditions as well as their standard deviation over time. Across all footwear conditions, the female pelvis is consistently tilted more anteriorly and to the left and rotated more clockwise until shortly before toe-off, the female thigh is consistently rotated more internally and tilted more laterally at the end of the stance phase and the female shank is rotated more internally and tilted less laterally at foot strike. With average standard deviations between 0.11 and 0.13, the gender differences in the orientation of the shank in the sagittal plane and of the foot in the sagittal and transverse planes are particularly affected by footwear condition. In contrast to the other footwear conditions, women strike at S6 (Puma Deviate Elite 2), PS (personal running shoe) and especially at S2 (Joe Nimble Addict) with a more posteriorly tilted shank compared to men and at BF (barefoot), S1 (Vivobarefoot Primus Lite III) and S4 (Puma Liberate 2) with a less posteriorly or more anteriorly tilted foot. Except for S3 (Nike Free 5.0), the female foot is less externally rotated for all footwear conditions (see Figure A55). In addition, gender differences with S4, S5 (Puma Velocity 2), S6 and PS only last until 40–90% of the stance phase, whereas with BF (barefoot), S1 and S2, they last throughout the entire stance phase. While S3 shows no gender differences in the transverse orientation of the foot, a stronger internal rotation of the female shank is observed earlier in the stance phase. Despite the impaired internal rotation of the foot, the female shank appears to follow its habitual movement path. This may be a reason for the more pronounced pronation in female runners found for this footwear condition. Potentially, S3 hinders the internal rotation of the foot at foot strike, which is more pronounced in women for the other footwear conditions. Due to the soft material and segmentation of the sole, a low torsional stiffness of the heel can be assumed, which may absorb the internal rotation by elastic torsion, whereas the harder and therefore presumably torsionally stiffer sole of the standard running shoe (S5), for example, is more likely to cause the sole to rotate relative to the ground.

The effect sizes of gender differences in segment angle parameters are listed in Table A16. Female runners show a greater peak forward tilt, left side tilt and clockwise rotation angle of the pelvis and a greater peak lateral tilt and internal rotation angle of the thigh across all footwear conditions. Women demonstrate a larger range of motion in the frontal plane of the pelvis and in the transverse plane of the thigh. With standard deviations between 0.13 and 0.20, footwear condition has the greatest effect on gender differences in the orientation of the foot. Female runners show a smaller range of motion in the sagittal plane of the foot for BF and S1 and a larger range of motion for PS than men. For BF, S1 and S2, gender differences in the transverse orientation of the foot are particularly pronounced, whereas they are not present at all in S3.

#### 3.3.7. Gender Differences in Segment Angular Velocities Across Various Footwear Conditions

**Figure 30:**
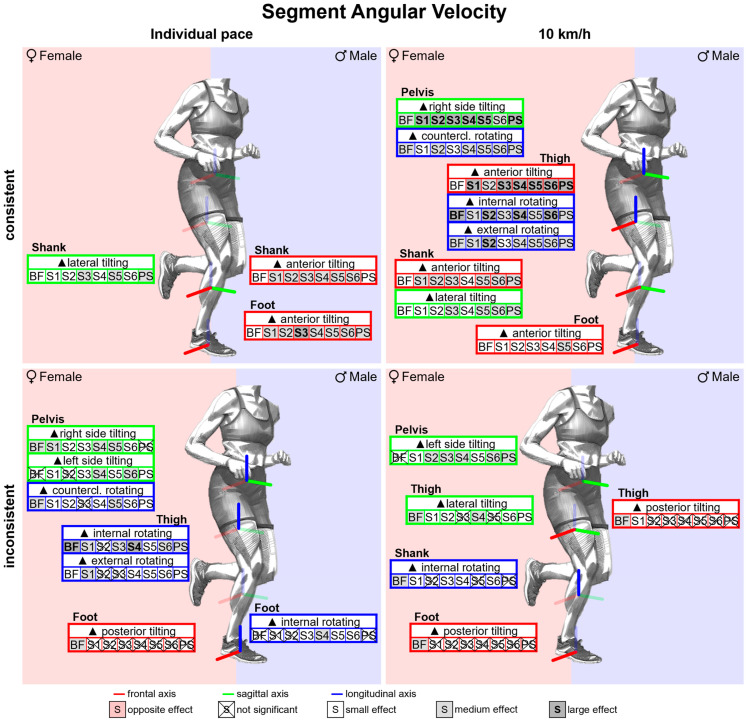
Gender differences in peak segment angular velocities across various footwear conditions at the participants’ individual training pace and a running speed of 10 km/h; only parameters with a significant difference and medium effect size in at least one footwear condition are presented and assigned to the gender with the on average higher value; consistent: significant and equidirectional gender difference across all footwear conditions; inconsistent: not significant or opposite gender difference in at least one footwear condition.

**Figure 31:**
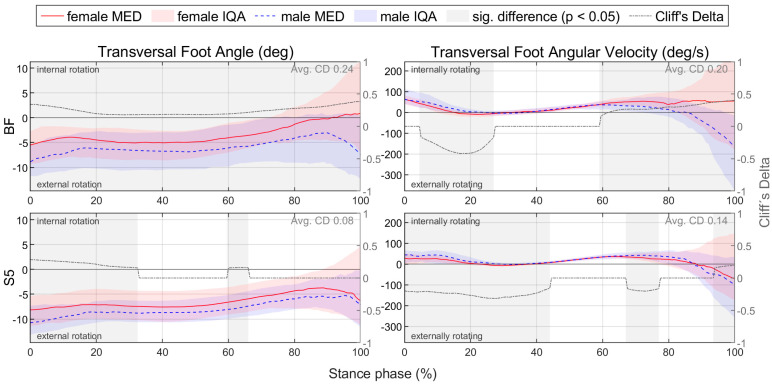
Gender differences in transverse foot angle and angular velocity when running barefoot (BF) and with standard running shoe (S5, Puma Velocity 2) at 10 km/h.

**Figure A56:**
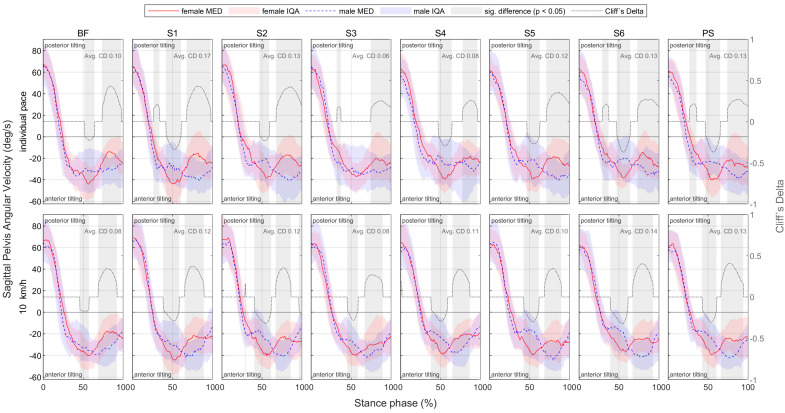
Gender differences in sagittal pelvis angular velocity.

**Figure A57:**
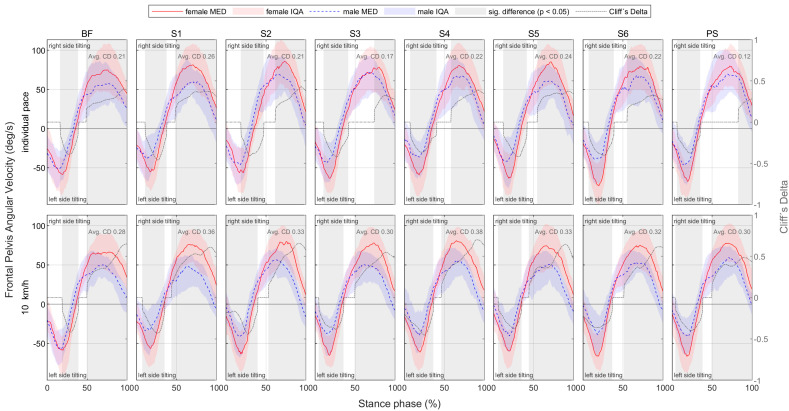
Gender differences in frontal pelvis angular velocity.

**Figure A58:**
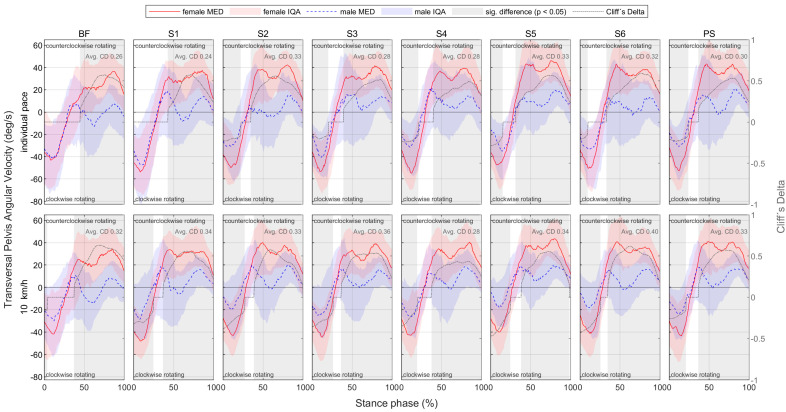
Gender differences in transverse pelvis angular velocity.

**Figure A59:**
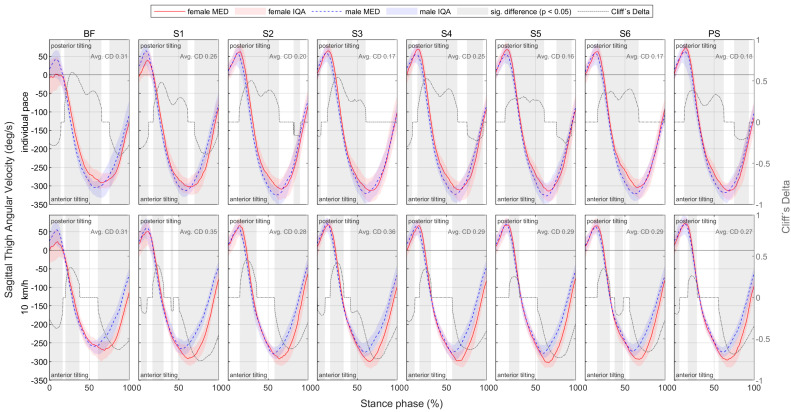
Gender differences in sagittal thigh angular velocity.

**Figure A60:**
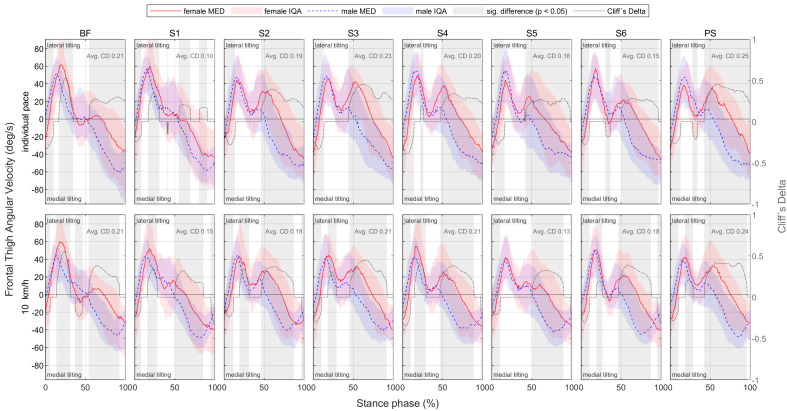
Gender differences in frontal thigh angular velocity.

**Figure A61:**
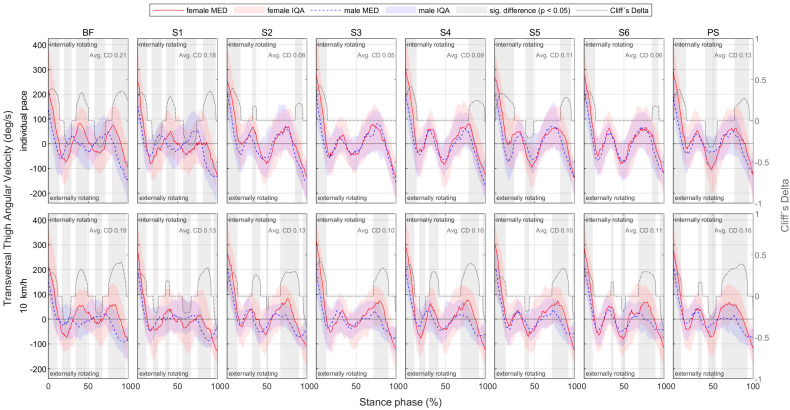
Gender differences in transverse thigh angular velocity.

**Figure A62:**
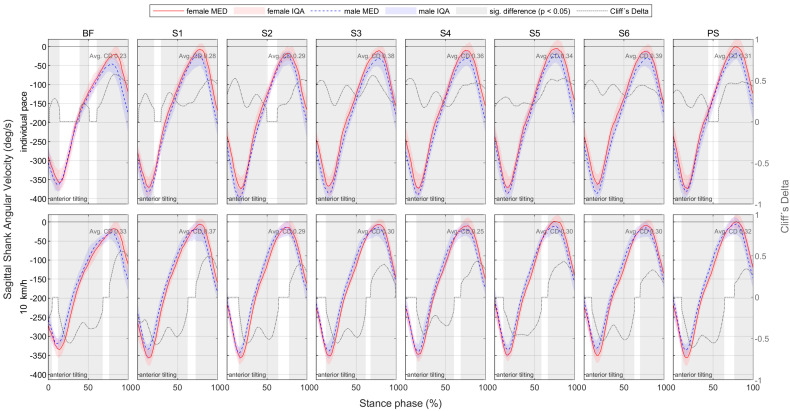
Gender differences in sagittal shank angular velocity.

**Figure A63:**
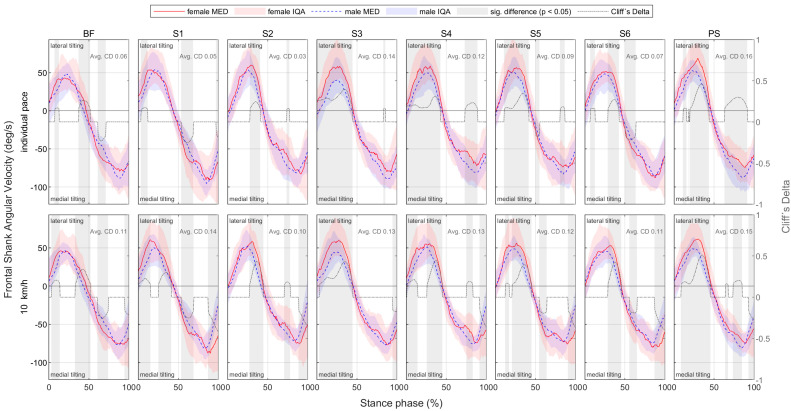
Gender differences in frontal shank angular velocity.

**Figure A64:**
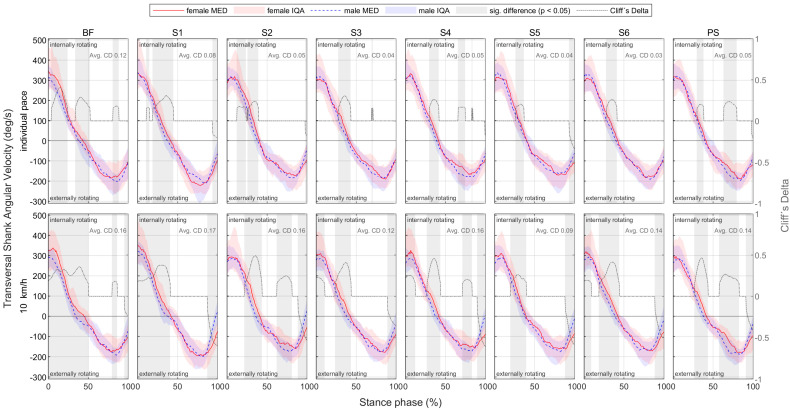
Gender differences in transverse shank angular velocity.

**Figure A65:**
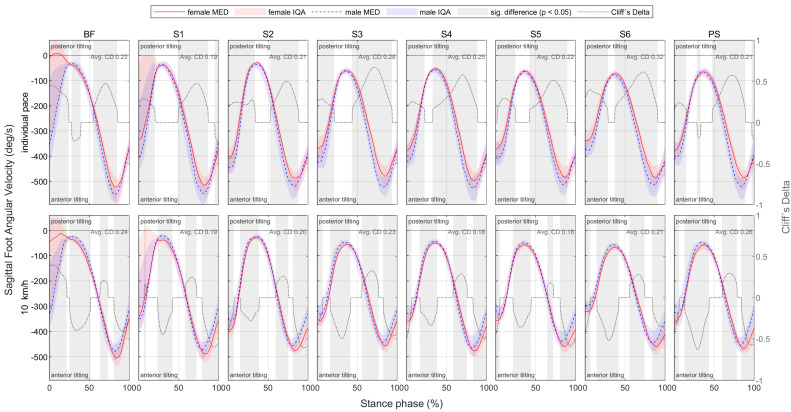
Gender differences in sagittal foot angular velocity.

**Figure A66:**
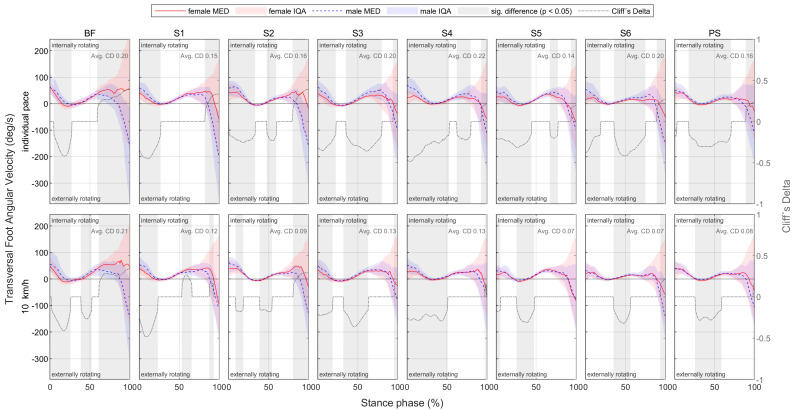
Gender differences in transverse foot angular velocity.

**Figure A67:**
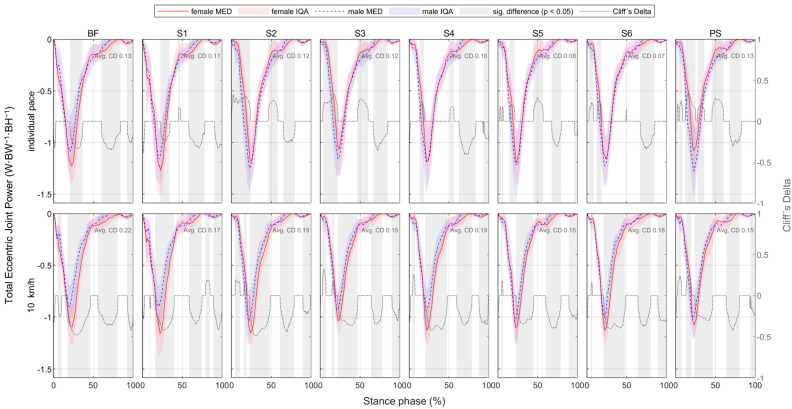
Gender differences in total eccentric joint power.

**Figure A68:**
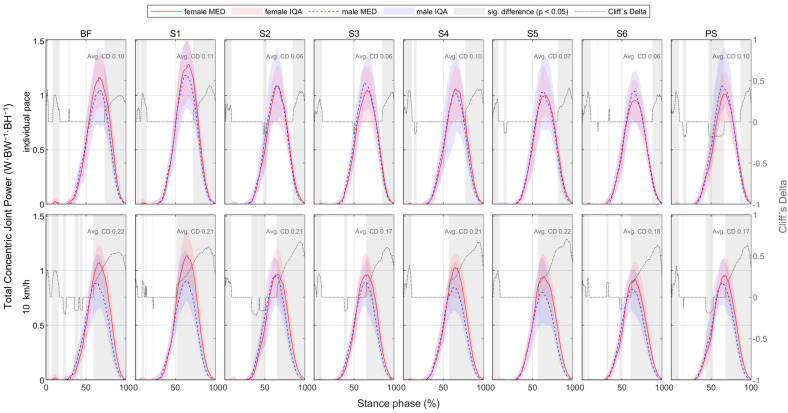
Gender differences in total concentric joint power.

**Figure A69:**
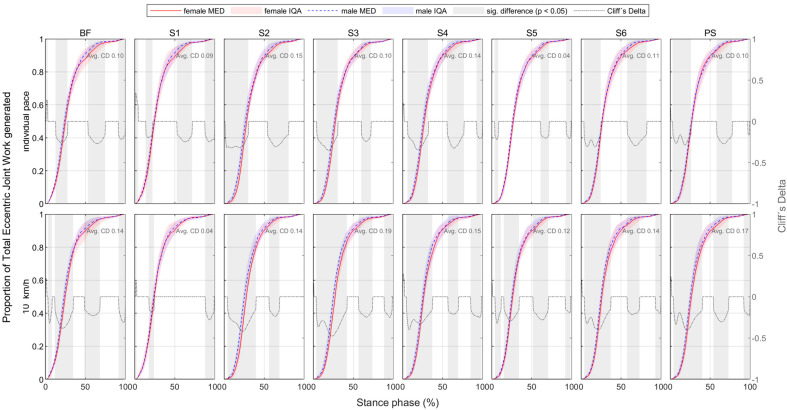
Gender differences in the proportion of total eccentric joint work generated.

**Figure A70:**
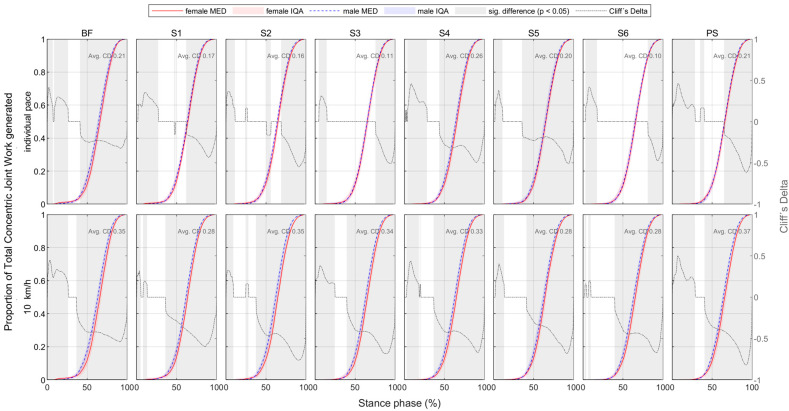
Gender differences in the proportion of total concentric joint work generated.

Figure 30 summarises notable gender differences in segment angular velocities across various footwear conditions. The curves of the segment angular velocities of female and male runners are juxtaposed in Figure A56, Figure A57, Figure A58, Figure A59, Figure A60, Figure A61, Figure A62, Figure A63, Figure A64, Figure A65 and Figure A66. Figure A80 shows the effect size of gender differences for the individual footwear conditions as well as their standard deviation over time. Across all footwear conditions, females exhibit a faster left side tilt and a faster clockwise rotation at the beginning and a faster right side tilt and a faster counterclockwise rotation of the pelvis at the end of the stance phase. At the end of the stance phase, the female thigh and foot tilt anteriorly faster, whereas the female shank tilts more slowly. In addition, the female thigh tilts medially more slowly in the second half of the stance phase. There are clear differences in foot rotation between minimum and maximum footwear conditions. In Figure 31, gender differences in the transverse orientation of the foot are compared representatively for BF (barefoot) and S5 (Puma Velocity 2). While the female foot for BF is less externally rotated than the male foot, gender differences for S5 are mostly not significant and less pronounced. The female runner appears to adapt her foot orientation for S5 compared to BF, while the male runner does not adapt his movement pattern. This can also be observed for angular velocity. At toe-off, men tend to externally rotate their foot in both footwear conditions, while women also tend to externally rotate their foot for S5, but internally rotate their foot for BF. The effect sizes of gender differences in peak segment angular velocities are listed in Table A17. Female runners exhibit higher peak segment angular velocities in the frontal and transverse planes of the pelvis, thigh and shank as well as in the sagittal plane of the thigh and shank. Shoe condition has the greatest influence on the anterior tilting velocity of thigh and shank. For BF, the female thigh tilts anteriorly more slowly while the foot tilts posteriorly more quickly.

#### 3.3.8. Gender Differences in Groups of Biomechanical Parameters and Variables over Time Across Various Footwear Conditions

**Figure 32:**
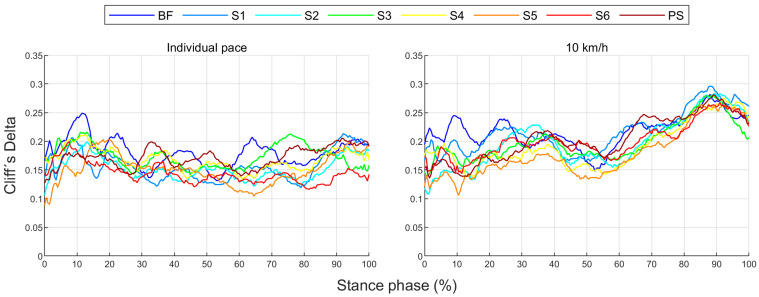
Effect size Cliff’s Delta for gender differences averaged over all biomechanical variables.

Finally, biomechanical gender differences in the individual variables are grouped to identify at which point in time, in which plane and in which groups of variables the greatest gender differences and the greatest effect of footwear condition on gender differences are present. Figure 32 shows the course of the effect size of gender difference for the individual footwear conditions averaged over all variables. While gender differences at individual speed are almost constant over time, gender differences at 10 km/h are more pronounced in the last 20% of the stance phase. Especially at foot-strike, BF (barefoot) seems to provoke the largest and S5 (Puma Velocity 2) the smallest gender differences. Figure A81 illustrates the progression of the standard deviation of the gender differences between footwear conditions for 10 km/h and individual pace, indicating the point in time at which the variance of gender differences is greatest. When all variables are considered, no major difference can be recognised over time for either speed condition. The greatest influence of footwear condition on gender differences in the GRF, joint moments, joint power and sagittal variables are found at the beginning of stance phase. The effect on the differences in angular velocities tends to be greater than that on joint and segment angles.

Table A9 summarises the time-averaged effect sizes of gender differences for the individual footwear conditions across all biomechanical variables. The largest gender differences with an MV > 0.5 can be found for the hip joint angle, the sagittal and frontal hip joint angular velocity, the sagittal knee joint angular velocity, the sagittal pelvic orientation, the frontal thigh orientation and the segmental angular velocity of the pelvis in the frontal and transverse plane and of the thigh and shank in the sagittal plane. Footwear condition has the greatest influence on gender differences, with an SD > 0.11 for knee joint power and for foot angle and angular velocity in the sagittal and transverse plane. For uniform running speed, the gender differences tended to be greater, while the effect of footwear condition on the gender differences was greater for the individual pace.

Gender differences in biomechanical variables are grouped by category in Table 18. The greatest gender differences are found in the segment angles and segment angular velocities and, for 10 km/h, additionally for the GRF and the joint angular velocities. Grouped by joint and segment, the greatest gender differences are found in the hips and pelvis. Footwear condition has the greatest effect on gender differences in the knee and foot. Averaged over all variables, there are greater gender differences at uniform running speed (MV 0.201 vs. 0.162), while the effect of footwear condition on gender differences is greater for individual running pace (SD 0.097 vs. 0.088). For both speed conditions, BF showed the largest gender differences (0.180 and 0.218). At 10 km/h, S5 (Puma Velocity 2) with a total effect size of 0.178 and S6 (Puma Deviate Elite 2) at individual speed with a total effect size of 0.144 produced the smallest gender differences.

Gender differences in biomechanical parameters are grouped by category and summarised in Table 19. At 10 km/h, the greatest gender differences occur in the joint angle velocities, in the hip, in the pelvis and in the frontal plane, and at individual speed in the joint angles, in the knee, in the pelvis and in the transverse plane. Footwear condition has the greatest effect on gender differences in ankle, shank and in the sagittal plane. Averaged across all parameters, there were greater gender differences at uniform running speed (MV 0.186 vs. 0.178), while the effect of footwear condition on gender differences is greater for individual running pace (SD 0.082 vs. 0.077). At individual pace, S5 and S6 show the smallest gender differences; at 10 km/h, S3 (Nike Free 5.0), S5 and PS (personal running shoe) show the smallest gender differences. For the barefoot condition, the greatest gender differences were found with women showing a lower load rate, a lower foot strike ankle angle and a slower peak knee flexion velocity. This could be due to the difference in foot strike pattern, with female runners being more likely than men to use a forefoot strike in barefoot running. Footwear condition and speed condition appear to interact with each other. While for 10 km/h, S3 provokes small and S6 large gender differences, the opposite is true for individual running speed.

#### 3.3.9. Gender Differences in the Timing of Ground Reaction Force and Joint Work Across Various Footwear Conditions

**Figure A71:**
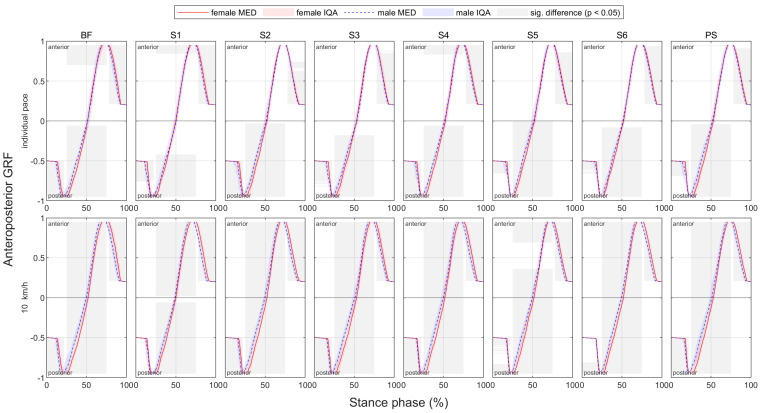
Gender differences in timing of normalised anteroposterior ground reaction force (GRF) for defined monotonic intervals.

**Figure A72:**
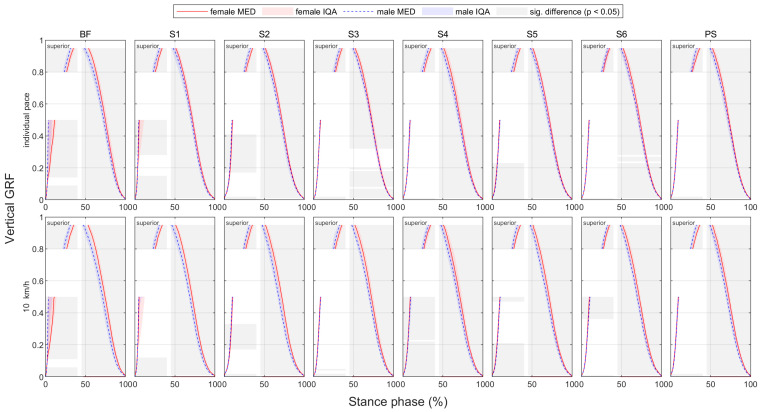
Gender differences in timing of normalised vertical ground reaction force (GRF) for defined monotonic intervals.

**Figure A73:**
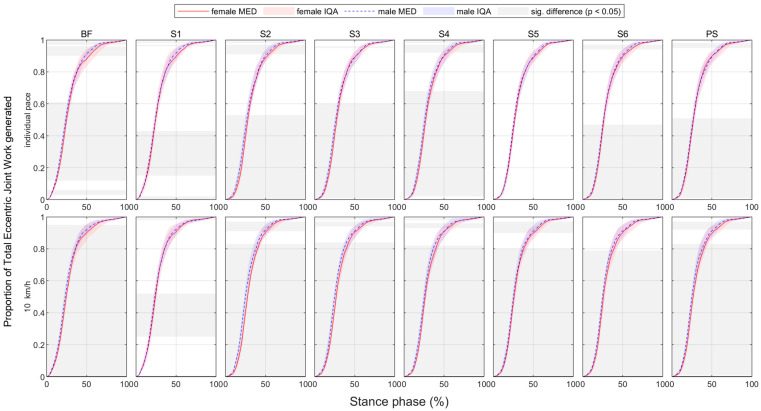
Gender differences in timing of proportion of total eccentric joint work generated.

**Figure A74:**
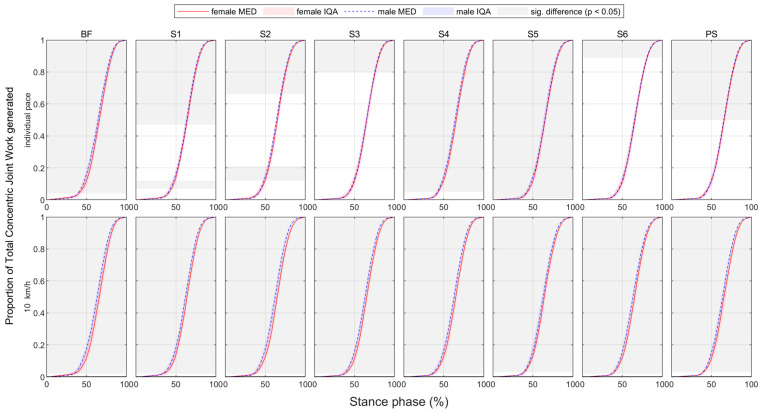
Gender differences in timing of proportion of total concentric joint work generated.

**Figure A75:**
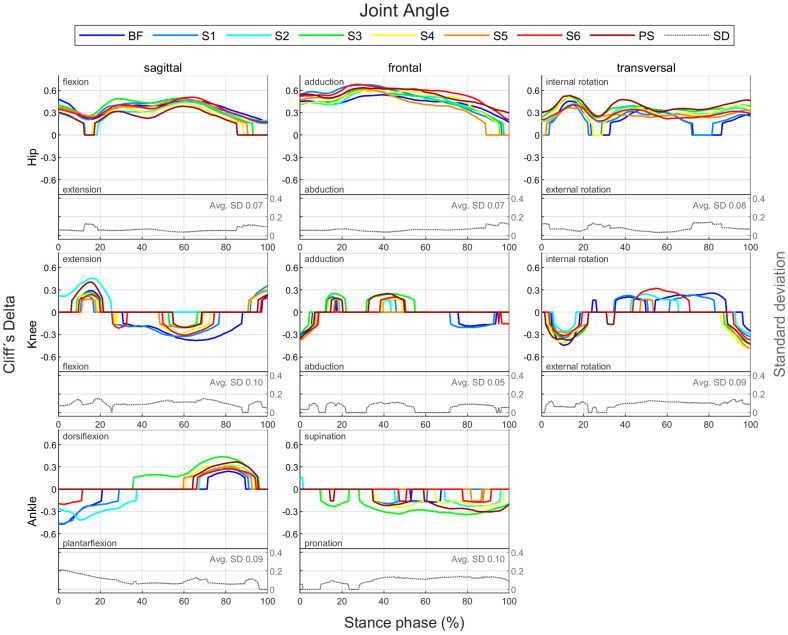
Effect size Cliff’s Delta for gender differences in joint angles when running at 10 km/h across footwear conditions.

**Figure A76:**
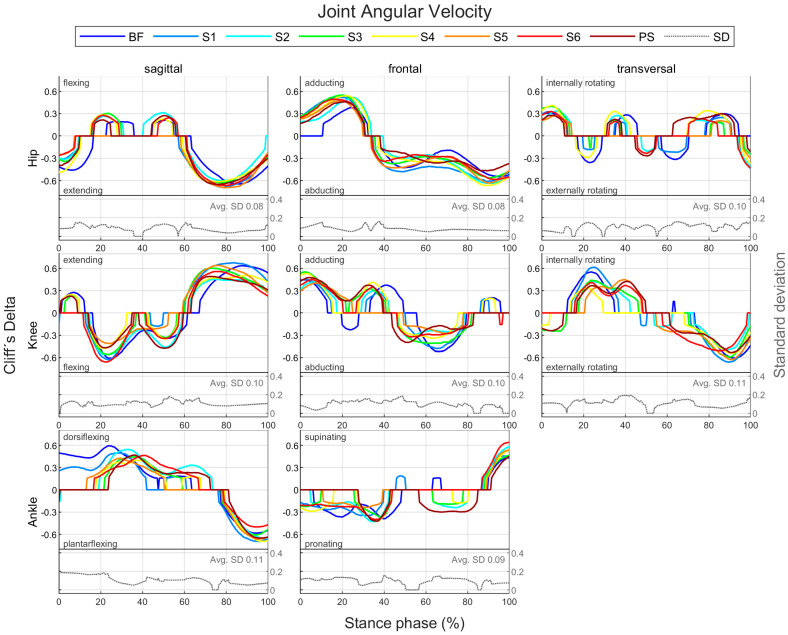
Effect size Cliff’s Delta for gender differences in joint angular velocities when running at 10 km/h across footwear conditions.

**Figure A77:**
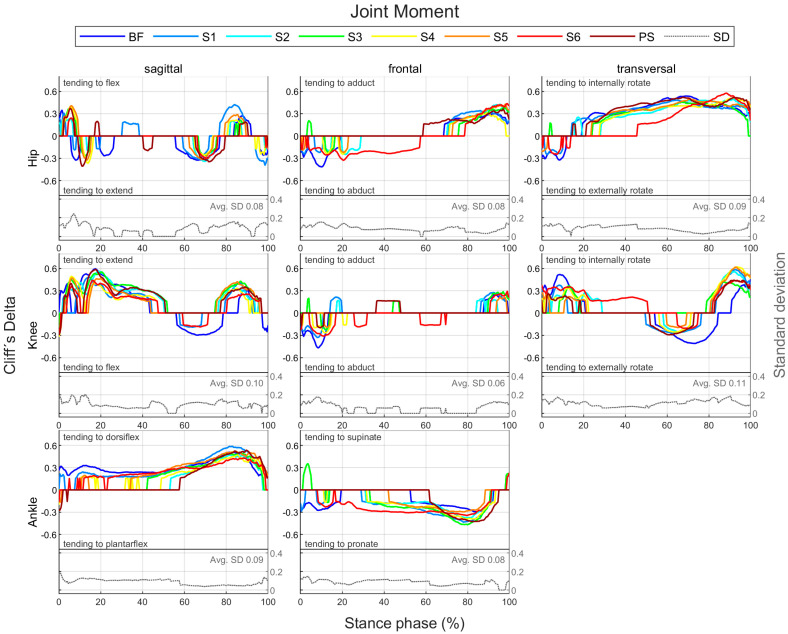
Effect size Cliff’s Delta for gender differences in external joint moments when running at 10 km/h across footwear conditions.

**Figure A78:**
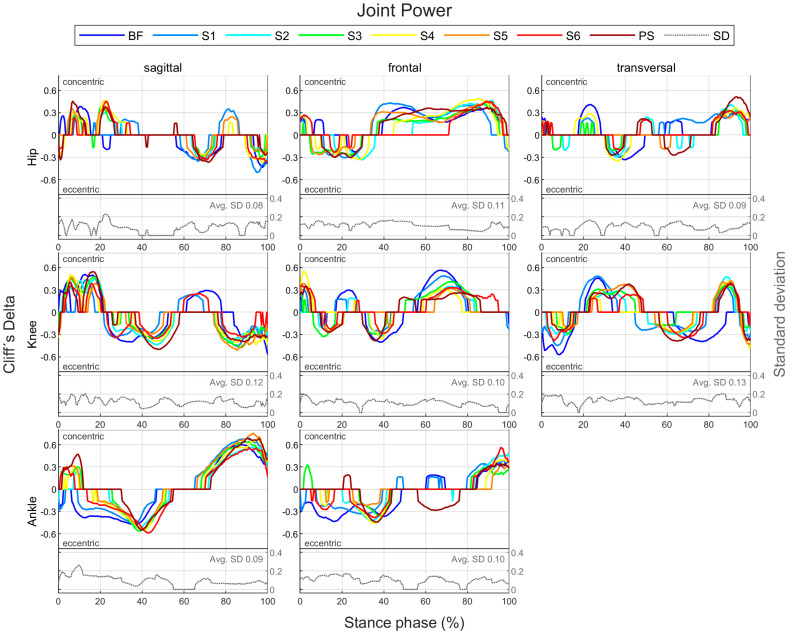
Effect size Cliff’s Delta for gender differences in joint powers when running at 10 km/h across footwear conditions.

**Figure A79:**
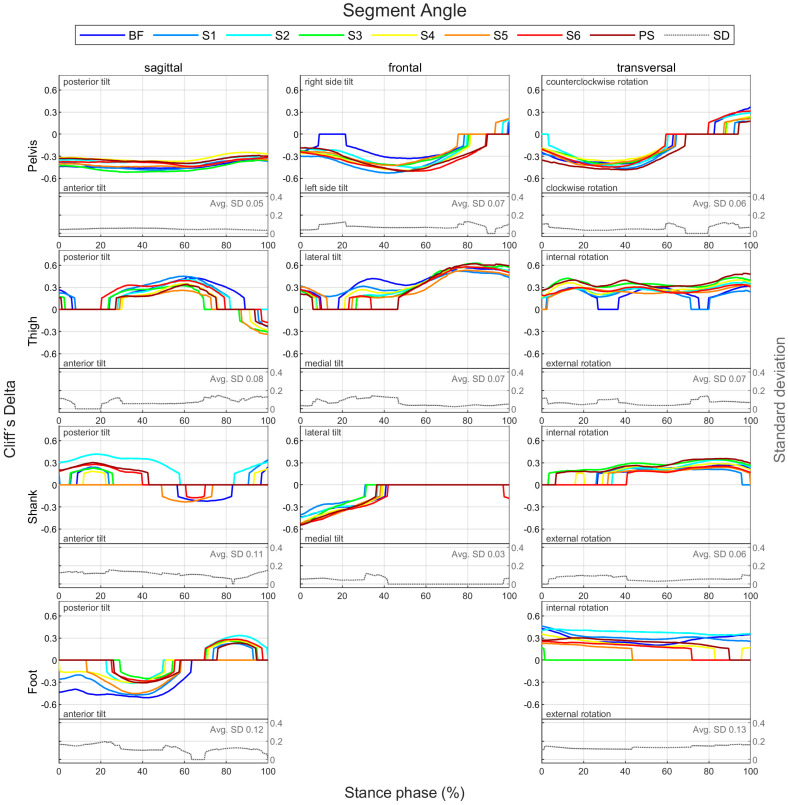
Effect size Cliff’s Delta for gender differences in segment angles when running at 10 km/h across footwear conditions.

**Figure A80:**
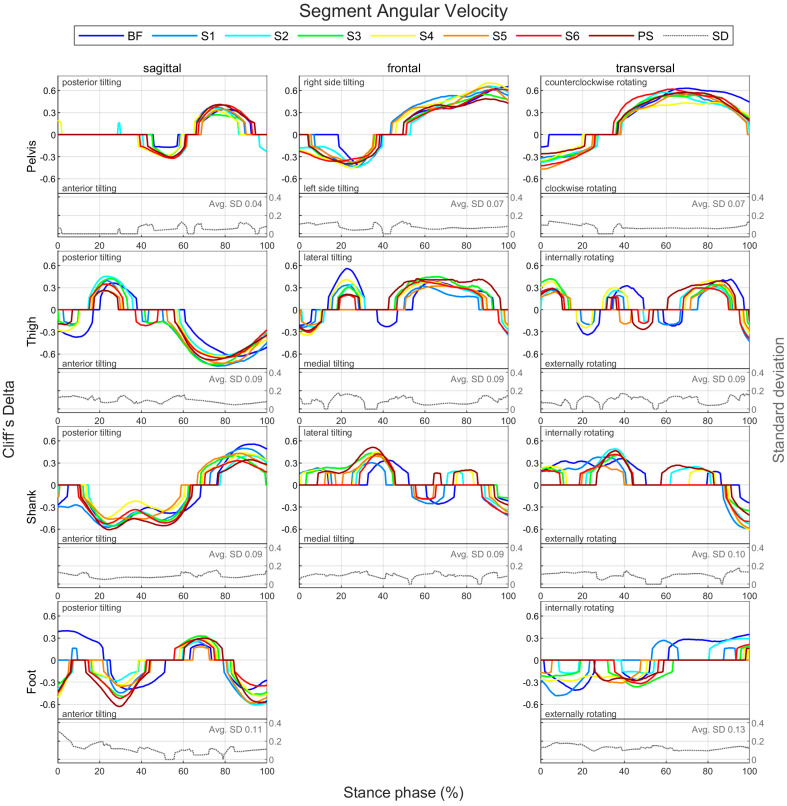
Effect size Cliff’s Delta for gender differences in segment angular velocities when running at 10 km/h across footwear conditions.

**Figure A81:**
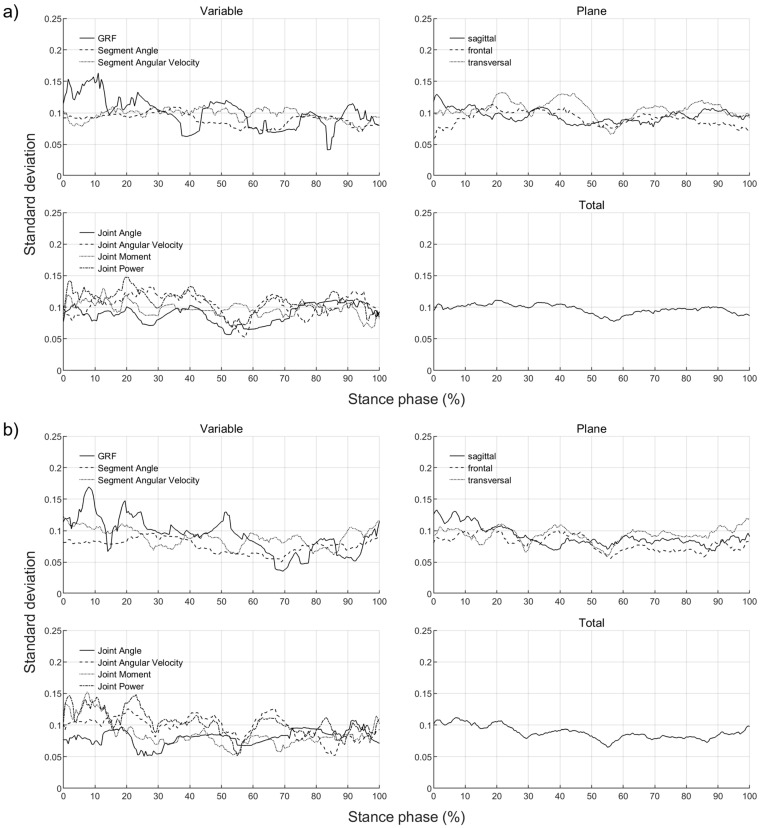
Standard deviation of gender differences between footwear conditions in variables, directions and in total over time when running at (**a**) individual pace and (**b**) 10 km/h.

Attention should be paid to the fact that gender differences in the time domain of individual biomechanical variables already described for the standard running shoe (S5) in Section 3.2.9 can be observed across all footwear conditions. The progressions of anteroposterior (Figure A71) and vertical GRF (Figure A72) as well as the proportion of eccentric (Figure A73) and concentric total joint work (Figure A74) generated at the respective point in time are shifted to the right in female runners compared to male runners, meaning that peak values occur earlier in male runners and later in female runners. Gender differences in the timing of ground reaction force and joint work are much more pronounced at a uniform running speed of 10 km/h than at the participants’ individual training pace.

### 3.4. Gender Differences in User Experience of Various Footwear Conditions During Running

Gender differences in the perception of shoe characteristics and the physical perception during running are presented in Table 20 and Table 21. On average, both genders rated their own shoes the best and the minimalist shoes the worst. There are greater differences in ratings between the shoes for women than for men. In general, female runners rated the test shoes worse and their personal running shoes better compared to male runners. The standard running shoe (S5) on the other hand, was rated similarly by both genders. The biggest gender differences are in the general rating, the performance, the joint load and the work of the foot muscles. Women rated S2 (Joe Nimble Addict) and S6 (Puma Deviate Elite 2) in general and the performance of S4 (Puma Liberate 2) and S6 in particular worse than men. The results of the user experience study indicate that, compared to men, women were more critical of the footwear conditions provided and more favourable towards their personal footwear. One possible reason is that the shoes provided are more in line with the requirements of male runners. However, this is contradicted by the great similarity between the personal running shoe characteristics of male and female runners (see Table 4). An alternative explanation is that women are more sensitive to differences in shoe characteristics and therefore accept a smaller range of properties. Women report larger joint and foot muscle loads in the minimal footwear conditions S1-S3, which confirms a higher sensitivity of female runners. Within the running shoe conditions, women rated the joint stability for S4 in the knee and ankle and for S6 in the ankle significantly lower than men. In accordance with their ratings, S4 and S6 are the only footwear conditions that demonstrate large gender differences in the range of motion of the knee and ankle in the frontal plane. In addition, S4 and S6 show the greatest differences in knee abduction velocity and S4 also in ankle pronation velocity. Of the shoes provided, S5 shows the smallest gender differences in the subjective rating. This footwear condition also shows the smallest gender differences in running biomechanics.

## 4. Discussion

The present study reveals numerous gender differences in the dynamics and kinematics of running. In particular, female runners show larger peak angles in hip adduction and hip internal rotation as well as larger ranges of motion and angular velocities in the non-sagittal planes of the hip and knee joints as well as the pelvis and thigh segments. Analysing the orientation of the segments in the global coordinate system made it possible to understand the emergence of gender differences in the joint angles. These gender differences are much more pronounced at a uniform running speed of 10 km/h. At the participants‘ individual pace, however, women show lower peak forces. In women, the knee extensors perform a smaller proportion of the total eccentric work. This deficit is compensated for by the increased work of the plantar flexors and supinators in female runners. Many of the gender differences are consistent across footwear conditions, while others are dependent on footwear condition. For example, S3 is the only footwear condition where women exhibit a higher peak pronation with a medium effect size in both running speed conditions. On the other hand, S3 is also the only footwear condition without a gender difference in the orientation of the foot in both running speed conditions, while men show a greater external peak rotation of the foot in the other footwear conditions. The results of the present study are subsequently discussed via the following:

- Comparison with the literature (Section 4.1);
- Highlighting the limitations of the present study (Section 4.2).

### 4.1. Comparison with the Literature

As most studies on gender differences in running biomechanics were conducted with standard running shoes at a uniform speed, the results for the standard running shoe (S5) at a running speed of 10 km/h are used for comparison with the literature. In accordance with the literature, female runners exhibited a shorter ground contact time [5,15,17,40,54,55], a slightly lower vertical active peak [3,4,22] and a slightly higher vertical loading rate [3,4,5,6]. However, in contrast to half of the literature, no significant difference was found for vertical active peak and vertical loading rate in this study, which could be due to the use of a less powerful non-parametric test. While the literature showed no significant gender differences in peak horizontal forces, female runners showed lower posterior and medial force peaks, but a higher anterior force peak, each with a small effect size. In line with the literature, females showed greater peak values in hip adduction and hip internal rotation [5,10,11,12,13,14,15], a larger hip and knee range of motion in the frontal plane [7,17], a larger ankle range of motion in the sagittal plane [23,24] and a more extended knee at toe-off [3,14]. In addition, the present study demonstrated greater peak hip flexion angles and larger sagittal hip and knee ranges of motion in female runners, whereas the literature to date has assumed no or a differently orientated gender difference in the sagittal plane [5,6,12,13,17]. On the other hand, numerous studies with standard and stability shoes showed greater peak knee abduction angles in female runners [6,13,14,19,20], whereas no significant differences were found in the present study. The non-existent gender difference in peak ankle pronation confirms the results of Takabayashi et al. [23] and Isherwood et al. [5], although the literature is inconsistent on this point and other sources have shown larger [6,22] or smaller [13,14] peak pronation angles in female runners.

Apart from the joint angles, there are only a few reference values for other biomechanical parameters. The present study demonstrated higher joint angle velocities in all joints and planes in female runners, while the literature found no significant differences [5,22] or differences were not tested for significance [12]. Gender differences in joint moments are non-existent or minor, which is consistent with the literature [5,12,15]. In addition to the lower external knee flexion moment in female runners already shown in the literature [5,10,12], the present study also revealed a larger external knee extension moment. The present study confirms the results of Ferber et al. [12], whereby women perform more hip joint work in the transverse plane. Based on the present study, it can be concretised that this applies to the concentric and eccentric work of the hip external rotators and, at 10 km/h, to the concentric hip abduction work. By considering the direction and type of joint work, the present study also showed for the first time that women perform more concentric hip extension work and more eccentric knee flexion, plantar flexion and supination work compared to men, but less eccentric knee extension work [12].

The results of the present study confirm a more pronounced pelvic anterior tilt and a larger range of motion of the female pelvis in the frontal and transverse planes as reported in the literature [7,11,17,24], but contradict the results of Schache et al. [17] regarding a larger range of motion in the sagittal plane. Furthermore, no reference values for gender differences in segment angles, segment angular velocities, joint power and joint work are given in the literature.

The results of the present study show that footwear condition affects the extent and, in some cases, even the existence of differences between female and male runners in a number of biomechanical parameters. Potential effects of different shoe characteristics on biomechanics are well documented [27,28,29,30,34,35,36,37,41,42]. The fact that some biomechanical values, e.g., ankle kinematics, are influenced by the footwear condition, while others, e.g., pelvis kinematics, are not, could be due to the fact that only a certain part of the principal movement components is influenced by the footwear condition [9]. The effect of footwear condition on gender differences indicates that female and male runners not only exhibit differences in running biomechanics, but also react differently to changes with shoe characteristics. Schrödter et al. [47] found a higher footwear-related variability in knee joint kinematics in women compared to men and hypothesised that differences in anthropometrics and strength capacity might make women more susceptible to footwear-induced changes in running biomechanics. These differences may also explain different reactions to footwear conditions in the present study, for example, for S3 compared to the other footwear conditions, with women showing a larger and men a smaller peak pronation. Although there is no other study that has investigated the influence of footwear condition on gender differences, the results of Nigg et al. [56] support the hypothesis of footwear-dependent gender differences, as a gender difference in ankle moment was found when walking with specific minimal shoes, whereas no such difference was observed in any previous study for barefoot or normal shoe conditions.

### 4.2. Limitations

Only recreational runners took part in the study. As this is by far the largest group of runners, the results are relevant for a wide population. However, the results cannot be applied to inexperienced runners or professional athletes.

The trials were conducted with eight shoe and two speed conditions. As the shoes did not differ in individual characteristics under otherwise identical conditions, it is not possible to draw any conclusions about running biomechanics and gender differences from the manipulation of individual shoe characteristics. The wide range of shoe models, from minimal to maximal shoes, made it possible to investigate their influence on gender differences in running biomechanics. In addition, the running shoe models are widely available on the market, which is why the results are relevant for a wide section of the running population. The runs were carried out both at a uniform running speed of 10 km/h and at the individual training speed of the participants. A running speed of 10 km/h is common among recreational runners and can be achieved by all participants. By examining both conditions, gender differences could be analysed at uniform speed, where similar ground reaction forces are to be expected, as well as at individual speed, which was predominantly used by the participants and thus best reflected their actual training loads. The use of further shoe and speed conditions was rejected due to the additional load on the test subjects.

The subjects warmed up in their own shoe models and were able to familiarise themselves with the current footwear condition in three-minute runs. During the runs, only the acute reactions of the runners to the altered footwear characteristics were recorded and analysed. It cannot be precluded that fatigue or long-term familiarisation may result in different adaptions.

A marker set-up with a total of 16 markers was used to record lower body movements. This model has been validated through its frequent citation in peer-reviewed publications [57,58]. In addition, the markers were placed at the anatomical landmarks with the utmost care. Although only two markers were attached to the right foot of the participants, the ankle joint in both the sagittal and frontal planes could be analysed by considering the anatomical axes of the talocrural and talocalcaneal joints.

The movements were recorded at a frequency of 100 Hz. As running at these speeds does not involve highly dynamic movements, it can be assumed that the selected recording frequency is sufficient and does not result in any loss of information.

The data were not recorded continuously during a single run. Instead, one single contact on the force plate was analysed for each of multiple runs. This discontinuous analysis might have a certain error impact on the experimental results; however, this issue was mitigated by executing each run related to a specific experimental condition six times. Subsequent studies could use multiple force plates or instrumented walkways [59], instrumented treadmills [60], instrumented insoles [61] or wearable devices [62] to continuously analyse running biomechanics.

Only the biomechanics of the supporting leg were analysed during the stance phase. The greatest forces in running occur in the supporting leg. This is also where most of the muscle work is generated. It can be assumed that the movements of the upper body correspond to the movements of the lower body and that, in general, the same gender differences exist for both legs and potential exceptions will not be significant given the large number of participants. Even if the analysis of the upper body and swing phase could provide additional information, it can be assumed that the analysis of the supporting leg will reveal the most relevant gender differences regarding running biomechanics.

Only the forces and movements of the right supporting leg were measured. In a symmetrical walking or running pattern (phase shift of 50%), we can assume that the right and left legs show similar force and movement patterns when averaged over the participants. Deviations from a 50% phase shift affecting force and movement patterns usually occur in gait impairments and fatigue. Gait disturbances were one of the exclusion criteria. The probability of fatigue was estimated to be small due to the low running speed and distance and was additionally counteracted by taking sufficient recovery breaks. Although precautions were taken so that a 50% phase shift between right and left leg was not affected, we could not entirely exclude the fact that force and movement patterns were not equally distributed between both legs.

Although the foot strike pattern has a significant influence on lower body biomechanics, it was not prescribed for the participants. As shown in the results, the foot strike pattern of the runners changed depending on the footwear condition. Therefore, the plantar touchdown habit of an individual is not consistent. Rather, the adaptation of the foot strike pattern is part of the runner’s response to the footwear condition. The foot strike pattern was not prescribed in this study in order not to influence the runner’s natural response to the different footwear conditions.

The same anthropometric data were used to determine the mass, centre of mass and radius of gyration of the segments for both genders. In the general population, men and women show differences in body mass distribution. As the participants are ambitious recreational runners, it can be assumed that gender differences in anthropometry are smaller compared to the general population. Due to the high variation within the genders and the lack of availability of gender- and group-specific anthropometric models, it is questionable whether the use of general gender-specific anthropometric data provides any added value. The individual determination of anthropometric data for each subject was rejected due to the enormous amount of time and effort involved.

The joint power was determined on the assumption that agonists and antagonists are not simultaneously activated. Even though this does not correspond to the reality of natural movements, this is a widespread assumption for calculating joint power and nevertheless allows an estimation of the joint work generated by the respective muscle groups.

## 5. Conclusions

The movements of 37 recreational runners (19 f, 18 m) were recorded for two speed conditions (uniform running speed of 10 km/h and participants’ individual training speed) and eight footwear conditions (ranging from barefoot to carbon-plated shoes) using a motion capturing system and force plates. Kinematics and dynamics of the right leg were analysed during the stance phase and differences between genders were investigated using non-parametric statistics.

Reporting the effect size, Cliff’s Delta ensured a simple, standardised and quantitative interpretation of the direction and strength of gender differences. This allowed for a comparison of gender differences across several footwear conditions for individual parameters as well as for variables as a function of time. The presented approach using Cliff’s Delta has proven to be suitable whenever differences between two groups are to be compared for a large number of parameters, for variables vs. time and for multiple conditions. Furthermore, by normalising variables to both, stance phase and magnitude, differences in timing between the two groups could be analysed for monotonous sections.

Numerous gender differences reported in the literature were confirmed, such as greater peak values and greater ranges of motion in the frontal and transverse planes of the hip. This study identified further previously undiscovered gender differences in running biomechanics. The female pelvis is initially more left side tilted and rotated clockwise, whereas at toe-off, it is more right side tilted and rotated counterclockwise. The greater range of motion in the non-sagittal planes is also reflected in the distribution of joint work. In women, the knee extensors generate a smaller proportion of the total joint work, while ankle supinators generate a greater proportion of the eccentric joint work, and the hip abductors tend to generate a greater proportion of the concentric joint work. Both genders tilt the thigh laterally from foot strike to mid-stance. While the male thigh returns to the initial position at toe off, the female thigh remains in a laterally tilted position. Both genders have a hip adduction of 0° at toe off. Therefore, the female pelvis and thigh are, as a complete frame, more laterally tilted than those of the male. Some findings indicate a reduced running efficiency of female runners. The proportion of the deceleration phase in the stance phase is greater for women. At a uniform running speed of 10 km/h, women exhibit greater total concentric joint work when normalised to height and body weight. As a result of more pronounced eccentric knee flexion work shortly before toe-off, female runners lose a greater proportion of their propulsive energy in absolute and relative terms. By statistically analysing gender differences in timing, a time shift in GRF and the total joint work generated by female runners was discovered. Female runners show force peaks and produce the total joint work later relative to the stance phase. Despite the on average 14% lower individual training speed of the female participants, in general, there were smaller gender differences for individual running speed than for uniform running speed. This is particularly the case for joint and segment angular velocities. The more pronounced differences for the uniform speed may be due to the anthropometric differences between female and male runners. The running speed of the target group and its potential dependence on gender should be taken into account when developing running shoes.

To the best of the authors’ knowledge, for the first time, gender differences were analysed and compared across several footwear conditions. Overall, gender differences were smallest for the standard running shoe. Gender differences in sagittal pelvis orientation and non-sagittal hip joint angles are rather consistent, while, for example, angular displacement and velocity of the foot orientation and ankle joint as well as the joint work distribution were affected by footwear condition. While in the remaining footwear conditions, compared to male runners, females utilised the knee extensors less and hip abductors more for propulsion, these differences were not evident in the carbon-plated running shoe (S6). The carbon-plated shoe was also the only footwear condition where women demonstrated lower external hip and knee adduction moments and a larger pronation impulse than men. Unlike the other footwear conditions, where the gender difference was not significant or had a small effect, women showed a substantially more pronounced peak ankle pronation for the highly flexible minimal shoe characterized by a soft midsole (S3). While women demonstrated higher peak pronation velocity and a greater external peak pronation moment at uniform running speed in all other footwear conditions, these differences were not evident when running with their personal running shoes (PS). Furthermore, in contrast to some other footwear conditions, women running with their personal running shoes did not perform more total joint work for uniform running speed and, of all footwear conditions, most evidently showed less total joint work than men for individual running speed. Thus, female runners may run more efficiently when running with their familiar personal running shoes. The influence of footwear condition on gender differences in running efficiency is not consistent. Especially for barefoot and barefoot shoes, female runners showed greater eccentric and concentric total joint work at a uniform running speed. In contrast, in these footwear conditions, women did not exhibit greater eccentric knee flexion work shortly before toe-off, which reduced propulsion in the other shoe conditions. At participants’ individual training speed, there were on average smaller gender differences than at a uniform running speed of 10 km/h. However, this phenomenon is not consistent across all footwear conditions. For the carbon-plated running shoe, gender differences are more pronounced for uniform running speed, whereas for the highly flexible minimal shoe, greater differences occur for individual speed, which could be related to differences between these footwear conditions regarding their freedom of movement and longitudinal stiffness. The user experience study suggests that female recreational runners are more sensitive to joint and muscle loads as well as to changes in shoe characteristics, and therefore have a more restricted range of acceptable shoe characteristics compared to men and are less open to running in minimal shoes. Future studies should be aware that gender differences in certain biomechanical variables depend on footwear and running speed condition and that there is an interaction between these conditions. Findings from one shoe model cannot necessarily be generalised. Future studies on gender differences should therefore at least provide as accurate a description as possible of the footwear condition used.

The findings of this study may serve as a foundation for advances in bioengineering in several areas. The user experience study revealed a smaller range of acceptable product characteristics for women. The knowledge of gender differences in running biomechanics and the potential of influencing certain parameters by adapting shoe characteristics can be used in general for the development of sports and medical products, such as knee and ankle braces, and in particular for the development of running shoes. For example, the ankle kinematics can be influenced by the shoe design, while the movement of the pelvis remains unaffected. In addition, the data can be used to select shoe materials depending on body weight, gender and running speed. For female recreational runners, a sole material should be selected that has sufficient cushioning properties but is not as soft as to al-low extreme pronation angles. Innovative technologies such as smart wearable devices can be used in running training or within sports medicine during rehabilitation to obtain (real-time) biofeedback. The data collected demonstrate that the underlying threshold values for biofeedback should be chosen based on gender differences. Based on the findings, relevant biomechanical parameters and thus also advantageous positions of the wearable devices can be determined. For example, instrumentation of the pelvis appears to be particularly suitable for identifying profound changes in the movement pattern of runners, while instrumentation of the foot may be more indicative of a variation in shoe characteristics.

## Figures and Tables

**Table 1:** Running conditions of relevant publications on gender differences in running biomechanics.

Publication	Running Condition	Running Speed (m/s)	Footwear Specifications
Bazuelo-Ruiz et al. [3]	Overground	3.3	Kelme Gravity MC
Keller et al. [4]	Overground	1.5–6.0	“Nike Aircraft” (not further specified)
Isherwood et al. [5]	Overground	3.3	Not specified
Sinclair et al. [6]	Overground	4.0	Saucony ProGrid Guide 2
Nigg et al. [7]	Overground	3.3	Shoes with var. sole hardness (Asker C-40 − C-65)
Mohr et al. [8]	Treadmill	Self-selected	Personal
Almonroeder et al. [10]	Overground	4	New Balance NBA-801
Chumanov et al. [11]	Treadmill	1.8–3.6	Not specified
Ferber et al. [12]	Overground	3.7	Not specified
Sakaguchi et al. [13]	Overground	3.5	Adidas Adizero Boston
Phinyomark et al. [14]	Treadmill	Self-selected	Not specified
Gehring et al. [15]	Overground	3.0–5.0	Adidas Supernova Glide
Willson et al. [16]	Overground	3.7	New Balance NBA-629
Schache et al. [17]	Treadmill	4.0	Personal
Hannigan et al. [18]	Overground	Self-selected	Personal
Malinzak et al. [19]	Overground	5.1	Personal
Esculier et al. [20]	Treadmill	1.3–3.0	Saucony ProGrid Ride
Sinclair et al. [21]	Overground	4.0	Asics GT 2160
Hennig [22]	Overground	3.3	Five running shoes (not specified)
Takabayashi et al. [23]	Treadmill	Individual	Barefoot
Bruening et al. [24]	Treadmill	Leg length norm.	Personal
Sinclair et al. [25]	Overground	4.0	Asics 2160
Greenhalgh et al. [26]	Overground	4.0	Saucony ProGrid Guide 2

**Table 2:** Mean (SD) of participant characteristics.

Variable	Female	Male	Total
n	19	18	37
Age (years)	23.05 (1.99)	26.50 (7.23)	24.73 (5.45)
Height (m)	1.62 (0.36)	1.78 (0.06)	1.70 (0.27)
Mass (kg)	62.22 (8.62)	73.76 (8.26)	67.83 (10.18)
BMI (kg/m^2^)	21.57 (2.71)	23.41 (2.35)	22.46 (2.67)
Leg length (m)	0.85 (0.05)	0.89 (0.05)	0.87 (0.05)
Pelvic width (cm)	25.18 (2.08)	24.99 (1.74)	25.09 (1.90)
Pelvic depth (cm)	18.13 (1.67)	18.30 (1.13)	18.21 (1.42)
Q-angle (°)	14.11 (4.77)	9.94 (3.52)	12.08 (4.66)
Running volume (km/week)	21.17 (10.82)	25.06 (20.56)	23.06 (16.17)
Individual training pace (km/h)	10.74 (1.05)	12.50 (0.88)	11.59 (1.31)

**Table 3:** Characteristics of eight footwear conditions tested in reference size UK8; ID: identification code according to Figure 1.

Footwear Condition	ID	Mass (g)	Heel Height (mm)	Heel-to-Toe Drop (mm)	Motion Control Technologies	Flexibility ^1^	Minimalist Index ^2,3^
Barefoot ^4^	BF	0	0	0	0	5	100
Vivobarefoot Primus Lite III	S1	217	8	0	0	5	88
Joe Nimble Addict	S2	291	24	0	1	3.5	62
Nike Free Run 5.0	S3	172	23	9	2	5	64
Puma Liberate 2	S4	205	26	8	3	4	48
Puma Velocity 2	S5	264	34	10	4	3	28
Puma Deviate Elite 2	S6	214	36	8	5	1.5	26
Personal shoe, MV (SD)	PS	261 (30)	32.0 (4.7)	8.7 (2.2)	−	−	26.7 (8.7)

^1^ Flexibility is based on manual testing of longitudinal and torsional flexibility according to Esculier et al. [48]. The rating scale ranges from 0 (=minimum flexibility) to 5 (=maximum flexibility). ^2^ The Minimalist Index is calculated from mass, heel height, heel-to-toe drop, the number of motion control technologies and the flexibility according to Esculier et al. [48]. The scale ranges from 0 (=maximum shoe) to 100 (=barefoot). ^3^ The Minimalist Index of participant’s personal running shoes was adapted from Esculier et al. [48] and is calculated solely based on mass, heel height and heel-to-toe drop. ^4^ As the barefoot condition did not include shoes, the values representing the highest level of minimalism were selected for the individual criteria.

**Table 4:** Characteristics of participants’ personal running shoe models in reference size UK8.

Subject	Gender	Brand	Model	Mass (g)	Heel Height (mm)	Heel-to-Toe Drop (mm)	Minimalist Index ^1^
1	m	Nike	Epic React Flyknit	221	28	10	33
2	m	Adidas	Two Parley	283	29	6	33
3	m	Asics	Noosa Tri 14	198	26	5	47
4	m	Adidas	Adizero Boston II	255	40	9	27
5	m	Adidas	Adizero Boston 9	221	22	10	40
6	f	Adidas	Adizero SL	238	35	8	27
7	m	Nike	Revolution 6	245	34	11	20
8	m	TrueMotion	Solo	203	22	8	47
9	f	Asics	Gel Kayano 29	268	33	8	27
10	f	Nike	React Infinity Run	299	39	9	20
11	f	Brooks	Ghost 13	269	32	12	20
12	f	Hoka	Mach 5	215	29	5	47
13	m	Nike	React Miler 3	238	31	10	27
14	m	Nike	Pegasus Trail 4 React	251	36	10	20
15	m	Nike	Pegasus 39	244	33	10	20
16	f	New Balance	880	255	34	10	20
17	m	Asics	Gel Nimbus 22	294	31	10	20
18	m	New Balance	Fresh Foam 860	284	27	10	20
19	f	ON	Cloud Runner	281	28	9	27
20	f	New Balance	680	284	33	5	27
21	f	Asics	GT1000	252	30	8	33
22	m	Adidas	Ultra Boost DNA 5	311	22	10	27
23	f	Brooks	Aurora BL x17	227	37	6	33
24	m	Adidas	Solar Glide	278	37	10	13
25	f	On	Cloud Monster	255	35	7	27
26	f	Hoka	Clifton 9	231	33	6	33
27	f	Asics	Gel-Kayano 27	306	30	12	20
28	m	Adidas	Ultraboost 21	314	31	10	20
29	f	Nike	Pegasus 40	249	33	10	20
30	m	Brooks	Adrenaline GTS	246	35	12	20
31	f	Adidas	Solar Boost 4	288	32	10	13
32	f	Adidas	Solar Boost 3	301	31	10	20
33	m	Nike	Pegasus 38	274	32	9	27
34	f	Hoka	Bondy 6	292	37	4	27
35	m	Hoka	Mach 5	249	29	4	40
36	f	Asics	Nimbus 25	272	41	8	27
37	f	Brooks	Glycerin GTS 20	249	38	10	20
Female MV (SD)				265 (27)	33.7 (3.5)	8.26 (2.31)	25.6 (7.5)
Male MV (SD)				256 (34)	30.3 (5.2)	9.11 (2.08)	27.8 (10)
Total MV (SD)				261 (30)	32.0 (4.7)	8.68 (2.21)	26.7 (8.7)

^1^ The Minimalist Index is calculated from mass, heel height and heel-to-toe drop, as adapted from Esculier et al. [48]. The scale ranges from 0 (=maximum shoe) to 100 (=barefoot).

**Table 5:** Interpretation of variables (forces, angles, angular velocities, moments and powers) displayed as a function of time and joint and segment angle parameters.

Variable		Direction/Plane	Positive Value (>0)	Negative Value (<0)
GRF		anteroposterior	anterior	posterior
		mediolateral	lateral	medial
		vertical	proximal	−
Joint	Hip	sagittal	flexion	extension
		frontal	adduction	abduction
		transverse	internal rotation	external rotation
	Knee	sagittal	extension	flexion
		frontal	adduction	abduction
		transverse	internal rotation	external rotation
	Ankle	sagittal	dorsiflexion	plantarflexion
		frontal	supination/inversion	pronation/eversion
Segment	Pelvis	sagittal	posterior tilt	anterior tilt
		frontal	right side tilt	left side tilt
		transverse	counterclockwise rotation	clockwise rotation
	Thigh	sagittal	posterior tilt	anterior tilt
		frontal	lateral tilt	medial tilt
		transverse	internal rotation	external rotation
	Shank	sagittal	posterior tilt	anterior tilt
		frontal	lateral tilt	medial tilt
		transverse	internal rotation	external rotation
	Foot	sagittal	posterior tilt	anterior tilt
		transverse	internal rotation	external rotation

**Table 6:** Interpretation of Cliff’s Delta in terms of direction and effect size of gender differences. The colouring reflects the specified areas of Cliff’s Delta.

Cliff’s Delta	<−0.43	<−0.28	<−0.11	−0.11 < CD < 0.11	>0.11	>0.28	>0.43
Direction	female < male			female ≈ male	female > male		
Effect size	large	medium	small	−	small	medium	large

**Table 7:** Exemplary table for determining gender differences in each parameter for a specific footwear condition; Q1 = lower quartile; MED = median; Q3 = upper quartile; CD = Cliff’s Delta.

Parameters	Individual Pace								10 km/h							
	Female			Male			*p*	CD	Female			Male			*p*	CD
	Q1	MED	Q3	Q1	MED	Q3			Q1	MED	Q3	Q1	MED	Q3		
Peak posterior force (N·BW^−1^)	0.358	**0.413**	0.491	0.439	**0.491**	0.538	**0.00**	**−0.37**	0.345	**0.390**	0.423	0.368	**0.398**	0.447	**0.03**	**−0.17**
Peak anterior force (N·BW^−1^)	0.238	**0.265**	0.290	0.245	**0.302**	0.346	**0.00**	**−0.29**	0.226	**0.251**	0.273	0.202	**0.236**	0.269	**0.03**	**0.17**

CD in bold—significant difference (*p* < 0.05); red cell—female > male (CD > 0); blue cell—female < male (CD < 0); light colour—small effect (|CD| > 0.11); medium colour—medium effect (|CD| > 0.28); dark colour—large effect (|CD| > 0.43).

**Table 8:** Exemplary table comparing the effect size Cliff’s Delta (CD; shaded grey; the higher the value, the darker the colour) of gender differences in each variable rectified and averaged over time for each footwear condition; MV = mean of averaged absolute CD across footwear conditions (shaded pink; the higher the value, the darker the colour); SD = standard deviation between CD of all footwear conditions averaged over time (shaded green; the higher the value, the darker the colour).

Variable	Individual Pace										10 km/h									
	BF	S1	S2	S3	S4	S5	S6	PS	MV	SD	BF	S1	S2	S3	S4	S5	S6	PS	MV	SD
Anteroposterior GRF	0.12	0.12	0.19	0.20	0.18	0.13	0.20	0.19	0.165	0.099	0.31	0.29	0.33	0.28	0.27	0.28	0.28	0.31	0.295	0.098

**Table 9:** Exemplary table comparing the effect size Cliff’s Delta (CD; shaded red if the female value is higher and blue if the female value is lower) of gender differences in parameters for each footwear condition; MV = mean of CD across footwear conditions (shaded pink); SD = standard deviation between CD of all footwear conditions (shaded green).

Parameter	Individual Pace										10 km/h									
	BF	S1	S2	S3	S4	S5	S6	PS	MV	SD	BF	S1	S2	S3	S4	S5	S6	PS	MV	SD
Peak posterior force	**−0.26**	**−0.22**	**−0.35**	**−0.52**	**−0.37**	**−0.37**	**−0.55**	**−0.43**	0.38	0.11	0.15	**0.29**	0.13	−0.12	0.01	**−0.17**	0.04	−0.03	0.12	0.15
Peak anterior force	**−0.21**	**−0.24**	**−0.37**	**−0.39**	**−0.33**	**−0.29**	**−0.41**	**−0.31**	0.32	0.07	0.14	**0.18**	−0.01	0.09	0.04	**0.17**	0.00	0.08	0.09	0.07

CD in bold—significant difference (*p* < 0.05); red cell—female > male (CD > 0); blue cell—female < male (CD < 0); light colour—small effect (|CD| > 0.11); medium colour—medium effect (|CD| > 0.28); dark colour—large effect (|CD| > 0.43); pink cell—mean CD (the higher the value, the darker the colour); green cell—standard deviation (the higher the value, the darker the colour).

**Table 10:** Foot strike pattern distribution of female and male runners when running with a standard running shoe (S5, Puma Velocity 2).

Foot Strike Pattern ^1^	Individual Pace		10 km/h	
	Female	Male	Female	Male
Rearfoot strike	94%	85%	95%	82%
Midfoot strike	0%	10%	0%	11%
Forefoot strike	6%	5%	5%	7%

^1^ Calculated from foot strike angle based on Altman et al. [53].

**Table 11:** Gender differences in ground reaction force parameters when running with a standard running shoe (S5, Puma Velocity 2); BW = body weight in N.

	Ground Reaction Force Parameters															
Parameters	Individual Pace								10 km/h							
	Female			Male			*p*	CD	Female			Male			*p*	CD
	Q1	MED	Q3	Q1	MED	Q3			Q1	MED	Q3	Q1	MED	Q3		
Ground contact time (s)	0.260	**0.273**	0.286	0.242	**0.257**	0.280	**0.00**	**0.27**	0.274	**0.289**	0.300	0.282	**0.300**	0.316	**0.00**	**−0.29**
Deceleration phase proportion (−)	0.504	**0.523**	0.544	0.484	**0.518**	0.543	**0.05**	**0.16**	0.498	**0.518**	0.534	0.479	**0.496**	0.521	**0.00**	**0.30**
Vertical active peak (N·BW^−1^)	2.34	**2.48**	2.61	2.46	**2.69**	2.83	**0.00**	**−0.38**	2.31	**2.40**	2.56	2.31	**2.47**	2.58	0.20	−0.10
Vertical instantaneous load rate (N·s^−1^·BW^−1^)	60.0	**82.7**	100.3	75.8	**90.6**	113.6	**0.00**	**−0.25**	63.3	**71.5**	81.3	57.7	**67.8**	84.3	0.55	0.05
Vertical average load rate (N·s^−1^·BW^−1^)	41.7	**54.6**	71.2	48.9	**60.9**	75.1	**0.03**	**−0.17**	40.2	**48.2**	58.1	40.5	**46.3**	53.7	0.25	0.09
Vertical impulse (Ns·BW^−1^)	0.374	**0.388**	0.401	0.377	**0.389**	0.401	0.75	−0.03	0.381	**0.393**	0.405	0.389	**0.398**	0.412	**0.01**	**−0.21**
Peak posterior force (N·BW^−1^)	0.358	**0.413**	0.491	0.439	**0.491**	0.538	**0.00**	**−0.37**	0.345	**0.390**	0.423	0.368	**0.398**	0.447	**0.03**	**−0.17**
Peak anterior force (N·BW^−1^)	0.238	**0.265**	0.290	0.245	**0.302**	0.346	**0.00**	**−0.29**	0.226	**0.251**	0.273	0.202	**0.236**	0.269	**0.03**	**0.17**
Posterior impulse (N·s·BW^−1^)	0.025	**0.029**	0.033	0.027	**0.029**	0.034	0.18	−0.11	0.024	**0.027**	0.030	0.024	**0.027**	0.030	0.86	−0.01
Anterior impulse (N·s·BW^−1^)	0.018	**0.020**	0.022	0.017	**0.020**	0.023	0.11	−0.13	0.017	**0.020**	0.021	0.017	**0.018**	0.021	**0.03**	**0.17**
Peak medial force (N·BW^−1^)	0.078	**0.129**	0.179	0.101	**0.153**	0.216	**0.00**	**−0.26**	0.077	**0.114**	0.171	0.082	**0.150**	0.193	**0.02**	**−0.18**
Peak lateral force (N·BW^−1^)	0.015	**0.047**	0.082	0.015	**0.033**	0.072	0.39	0.07	0.014	**0.029**	0.073	0.012	**0.027**	0.064	0.59	0.04
Medial impulse (N·s·BW^−1^)	3.0 × 10^−^^3^	**9.1 × 10^−^^3^**	2.4 × 10^−^^2^	4.8 × 10^−^^3^	**1.6 × 10^−^^2^**	2.7 × 10^−^^2^	**0.01**	**−0.21**	0.00	**1.1 × 10^−^^2^**	2.4 × 10^−^^2^	3.8 × 10^−^^3^	**1.9 × 10^−^^2^**	2.8 × 10^−^^2^	0.10	−0.13
Lateral impulse (N·s·BW^−1^)	2.0 × 10^−^^4^	**1.4 × 10^−^^3^**	5.8 × 10^−^^3^	1.8 × 10^−^^4^	**5.6 × 10^−^^4^**	3.7 × 10^−^^3^	0.12	0.12	0.00	**7.4 × 10^−^^4^**	4.6 × 10^−^^3^	1.6 × 10^−^^4^	**7.1 × 10^−^^4^**	3.7 × 10^−^^3^	0.79	0.02

CD in bold—significant difference (*p* < 0.05); red cell—female > male (CD > 0); blue cell—female < male (CD < 0); light colour—small effect (|CD| > 0.11); medium colour—medium effect (|CD| > 0.28); dark colour—large effect (|CD| > 0.43).

**Table 12:** Gender differences in total eccentric and concentric joint work and their distribution between muscle groups when running with a standard running shoe (S5, Puma Velocity 2); BW = body weight in N; BH = body height in m.

Joint Work	Individual Pace								10 km/h							
	Female			Male			*p*	CD	Female			Male			*p*	CD
	Q1	MED	Q3	Q1	MED	Q3			Q1	MED	Q3	Q1	MED	Q3		
Total eccentric J·BW^−1^·BH^−1^)	0.061	**0.083**	0.098	0.076	**0.085**	0.104	**0.05**	**−0.16**	0.059	**0.077**	0.092	0.065	**0.074**	0.086	0.76	0.02
% Hip flexors	8.8	**17.3**	20.8	11.3	**15.1**	20.0	0.79	−0.02	8.2	**15.7**	21.0	9.8	**12.4**	16.6	0.16	0.11
% Hip abductors	6.0	**11.1**	15.7	5.4	**9.8**	16.8	0.48	0.06	5.7	**9.8**	16.7	5.2	**8.6**	16.1	0.32	0.08
% Knee extensors	33.8	**41.8**	46.6	38.3	**43.8**	52.1	**0.00**	**−0.23**	32.9	**43.3**	48.5	38.3	**46.4**	54.7	**0.00**	**−0.26**
% Ankle plantar flexors	22.3	**27.1**	37.2	17.6	**22.9**	29.6	**0.00**	**0.25**	21.9	**26.6**	38.0	18.5	**26.2**	33.5	0.06	0.15
% Ankle supinators	2.2	**4.2**	6.3	0.6	**1.7**	3.6	**0.00**	**0.41**	2.2	**3.9**	5.8	1.1	**2.5**	4.5	**0.00**	**0.26**
Total concentric J·BW^−1^·BH^−1^)	0.084	**0.100**	0.111	0.082	**0.097**	0.122	0.83	0.02	0.082	**0.095**	0.109	0.077	**0.087**	0.108	**0.02**	**0.18**
% Hip extensors	1.0	**2.8**	5.2	1.0	**2.1**	3.6	0.34	0.08	0.9	**2.6**	5.4	0.7	**1.7**	3.3	0.09	0.13
% Hip abductors	8.1	**16.4**	24.3	7.6	**14.4**	22.4	0.41	0.07	9.6	**16.3**	25.0	5.7	**12.4**	18.9	**0.00**	**0.24**
% Knee extensors	14.4	**21.6**	27.4	17.6	**24.8**	29.6	**0.01**	**−0.22**	14.9	**20.7**	27.2	19.0	**23.7**	30.8	**0.00**	**−0.24**
% Ankle plantar flexors	44.4	**48.5**	54.2	44.0	**49.4**	56.6	0.86	−0.01	44.3	**47.4**	53.6	43.3	**50.9**	56.5	0.08	−0.14
% Ankle supinators	4.4	**6.3**	11.0	4.9	**7.0**	9.1	0.84	0.02	4.1	**6.2**	9.5	5.7	**7.0**	9.8	0.20	−0.10

CD in bold—significant difference (*p* < 0.05); red cell—female > male (CD > 0); blue cell—female < male (CD < 0); light colour—small effect (|CD| > 0.11); medium colour—medium effect (|CD| > 0.28); dark colour—large effect (|CD| > 0.43).

**Table 13:** Gender differences in the absolute and relative loss of propulsive work caused by eccentric knee flexor work when running with a standard running shoe (S5, Puma Velocity 2).

Loss of Propulsive Work	Individual Pace								10 km/h							
	Female			Male			*p*	CD	Female			Male			*p*	CD
	Q1	MED	Q3	Q1	MED	Q3			Q1	MED	Q3	Q1	MED	Q3		
Absolute (J·BW^−1^·BH^−1^)	0.04	**1.66**	3.77	−0.04	**0.54**	1.52	**0.01**	**0.23**	−0.04	**1.58**	3.96	−0.13	**0.27**	1.21	**0.00**	**0.30**
Relative (%)	0.05	**1.68**	3.51	−0.03	**0.71**	1.77	**0.01**	**0.21**	−0.03	**1.50**	3.83	−0.14	**0.29**	1.34	**0.00**	**0.27**

CD in bold—significant difference (*p* < 0.05); red cell—female > male (CD > 0); blue cell—female < male (CD < 0); light colour—small effect (|CD| > 0.11); medium colour—medium effect (|CD| > 0.28); dark colour—large effect (|CD| > 0.43).

**Table 14:** Foot strike pattern distribution of female and male runners.

Gender	Foot Strike Pattern ^1^	Individual Pace								10 km/h							
		BF	S1	S2	S3	S4	S5	S6	PS	BF	S1	S2	S3	S4	S5	S6	PS
	Rearfoot strike	30%	57%	86%	93%	95%	94%	98%	100%	34%	56%	87%	92%	96%	95%	99%	100%
Female	Midfoot strike	9%	3%	9%	4%	1%	0%	2%	0%	13%	6%	9%	6%	1%	0%	1%	0%
	Forefoot strike	61%	41%	5%	4%	4%	6%	0%	0%	53%	38%	4%	2%	4%	5%	0%	0%
	Rearfoot strike	56%	68%	85%	83%	89%	85%	88%	82%	53%	71%	83%	83%	88%	82%	87%	84%
Male	Midfoot strike	24%	12%	4%	6%	8%	10%	0%	10%	29%	11%	9%	12%	5%	11%	3%	10%
	Forefoot strike	19%	20%	11%	11%	3%	5%	12%	8%	18%	18%	9%	6%	7%	7%	11%	7%

Background colour reflects foot strike pattern to facilitate comparison between genders; ^1^ Estimated from foot strike angle based on Altman et al. [53].

**Table 15:** Effect size Cliff’s Delta (CD) for gender differences in ground reaction force parameters for all footwear conditions; MV = mean of CD across footwear conditions; SD = standard deviation between CD of all footwear conditions.

Parameter	Individual Pace										10 km/h									
	BF	S1	S2	S3	S4	S5	S6	PS	MV	SD	BF	S1	S2	S3	S4	S5	S6	PS	MV	SD
Ground contact time	**0.18**	0.08	**0.26**	**0.27**	**0.25**	**0.27**	**0.31**	**0.23**	0.23	0.07	**−0.32**	**−0.41**	**−0.28**	**−0.33**	**−0.27**	**−0.29**	**−0.34**	**−0.26**	0.31	0.05
Proportion of deceleration	0.05	−0.11	0.12	−0.01	0.12	**0.16**	0.09	0.13	0.10	0.09	**0.23**	**0.16**	**0.46**	**0.30**	**0.35**	**0.30**	**0.46**	**0.32**	0.32	0.10
Vertical active peak	**−0.29**	**−0.24**	**−0.36**	**−0.33**	**−0.37**	**−0.38**	**−0.44**	**−0.38**	0.35	0.06	−0.09	0.04	**−0.15**	**−0.17**	**−0.20**	−0.10	−0.14	−0.13	0.13	0.07
Vert. instantaneous load rate	**−0.40**	**−0.31**	**−0.21**	**−0.38**	**−0.27**	**−0.25**	**−0.27**	**−0.20**	0.29	0.07	**−0.29**	−0.08	0.14	0.02	0.10	0.05	**0.26**	0.02	0.12	0.16
Vert. average load rate	**−0.37**	**−0.28**	**−0.36**	**−0.30**	**−0.24**	**−0.17**	**−0.32**	−0.14	0.27	0.08	**−0.24**	−0.03	−0.05	0.11	0.13	0.09	**0.17**	0.08	0.11	0.13
Vertical impulse	0.01	−0.05	−0.08	−0.07	−0.03	−0.03	−0.05	−0.13	0.06	0.04	**−0.26**	**−0.24**	**−0.33**	**−0.26**	**−0.33**	**−0.21**	**−0.24**	**−0.27**	0.27	0.04
Peak posterior force	**−0.26**	**−0.22**	**−0.35**	**−0.52**	**−0.37**	**−0.37**	**−0.55**	**−0.43**	0.38	0.11	0.15	**0.29**	0.13	−0.12	0.01	**−0.17**	0.04	−0.03	0.12	0.15
Peak anterior force	**−0.21**	**−0.24**	**−0.37**	**−0.39**	**−0.33**	**−0.29**	**−0.41**	**−0.31**	0.32	0.07	0.14	**0.18**	−0.01	0.09	0.04	**0.17**	0.00	0.08	0.09	0.07
Posterior impulse	−0.07	**−0.19**	**−0.22**	**−0.39**	**−0.25**	−0.11	**−0.34**	**−0.24**	0.22	0.11	**0.28**	**0.22**	**0.16**	0.05	0.12	−0.01	**0.21**	0.13	0.15	0.10
Anterior impulse	−0.08	−0.14	**−0.19**	**−0.17**	**−0.18**	−0.13	−0.13	**−0.16**	0.15	0.04	0.09	0.12	−0.06	0.06	−0.01	**0.17**	−0.06	0.06	0.08	0.09
Peak medial force	**−0.23**	**−0.26**	**−0.23**	**−0.37**	**−0.50**	**−0.26**	**−0.23**	**−0.34**	0.30	0.10	**−0.23**	−0.14	**−0.17**	**−0.23**	−0.15	**−0.18**	**−0.36**	−0.13	0.20	0.07
Peak lateral force	−0.03	−0.03	−0.14	−0.08	0.06	0.07	−0.04	−0.09	0.07	0.07	0.10	0.13	0.09	0.01	0.06	0.04	**0.21**	−0.09	0.09	0.09
Medial impulse	−0.13	−0.03	0.04	**−0.20**	**−0.34**	**−0.21**	−0.03	**−0.17**	0.14	0.12	−0.15	−0.11	**−0.16**	−0.13	−0.11	−0.13	**−0.32**	−0.02	0.14	0.08
Lateral impulse	0.00	−0.07	−0.11	0.03	0.13	0.12	−0.03	0.00	0.06	0.08	0.10	0.12	0.07	0.05	0.02	0.02	**0.20**	−0.07	0.08	0.08

CD in bold—significant difference (*p* < 0.05); red cell—female > male (CD > 0); blue cell—female < male (CD < 0); light colour—small effect (|CD| > 0.11); medium colour—medium effect (|CD| > 0.28); dark colour—large effect (|CD| > 0.43); pink cell—mean CD (the higher the value, the darker the colour); green cell—standard deviation (the higher the value, the darker the colour).

**Table 16:** Effect size Cliff’s Delta (CD) for gender differences in total eccentric and concentric joint work and their distribution between muscle groups; MV = mean of CD across footwear conditions; SD = standard deviation between CD of all footwear conditions.

Joint Work	Individual Pace										10 km/h									
	BF	S1	S2	S3	S4	S5	S6	PS	MV	SD	BF	S1	S2	S3	S4	S5	S6	PS	MV	SD
Total eccentric	0.11	0.04	**−0.28**	**−0.26**	−0.15	**−0.16**	−0.15	**−0.34**	0.19	0.15	**0.29**	**0.25**	0.04	0.05	0.11	0.02	**0.17**	−0.04	0.12	0.12
% Hip flexors	0.00	0.00	0.04	0.00	0.00	−0.02	0.02	0.06	0.02	0.03	0.10	−0.05	0.06	0.15	0.00	0.11	−0.01	0.13	0.08	0.07
% Hip abductors	−0.13	0.04	**0.17**	0.07	0.03	0.06	**0.19**	**0.19**	0.11	0.11	**−0.16**	0.00	0.08	0.08	0.14	0.08	0.03	**0.16**	0.09	0.10
% Knee extensors	**−0.46**	**−0.42**	**−0.35**	**−0.40**	**−0.31**	**−0.23**	**−0.38**	**−0.36**	0.36	0.07	**−0.46**	**−0.38**	**−0.32**	**−0.34**	**−0.29**	**−0.26**	**−0.23**	**−0.32**	0.33	0.07
% Ankle plantar flexors	**0.33**	**0.23**	**0.28**	**0.34**	**0.32**	**0.25**	**0.19**	**0.17**	0.26	0.07	**0.29**	**0.20**	**0.16**	**0.18**	**0.19**	0.15	0.14	0.11	0.18	0.06
% Ankle supinators	**0.58**	**0.45**	**0.36**	**0.40**	**0.55**	**0.41**	**0.33**	**0.38**	0.43	0.09	**0.51**	**0.42**	**0.46**	**0.30**	**0.37**	**0.26**	**0.37**	**0.30**	0.37	0.09
Total concentric	0.09	0.10	−0.06	−0.07	−0.02	0.02	0.01	−0.12	0.06	0.08	**0.20**	**0.31**	−0.03	0.16	**0.17**	**0.18**	0.11	0.05	0.15	0.10
% Hip extensors	0.14	**0.18**	0.00	0.04	0.03	0.08	−0.06	0.03	0.07	0.08	**0.21**	**0.16**	0.15	0.13	0.09	0.13	0.03	0.02	0.12	0.07
% Hip abductors	**0.23**	**0.29**	**0.29**	0.08	0.10	0.07	**0.22**	**0.19**	0.18	0.09	**0.26**	**0.28**	**0.25**	**0.21**	**0.24**	**0.24**	0.10	**0.37**	0.25	0.08
% Knee extensors	**−0.23**	**−0.22**	**−0.30**	**−0.33**	**−0.18**	**−0.22**	**−0.16**	**−0.30**	0.24	0.06	**−0.26**	**−0.23**	**−0.28**	**−0.26**	**−0.28**	**−0.24**	−0.13	**−0.36**	0.26	0.06
% Ankle plantar flexors	−0.15	**−0.24**	−0.09	0.11	−0.01	−0.01	−0.12	0.02	0.09	0.11	−0.15	**−0.31**	−0.08	−0.04	**−0.16**	−0.14	−0.10	−0.07	0.13	0.08
% Ankle supinators	0.03	0.01	0.02	−0.05	0.02	0.02	−0.02	−0.01	0.02	0.03	−0.06	0.01	0.00	−0.09	−0.03	−0.10	0.01	**−0.27**	0.07	0.10

CD in bold—significant difference (*p* < 0.05); red cell—female > male (CD > 0); blue cell—female < male (CD < 0); light colour—small effect (|CD| > 0.11); medium colour—medium effect (|CD| > 0.28); dark colour—large effect (|CD| > 0.43); pink cell—mean CD (the higher the value, the darker the colour); green cell—standard deviation (the higher the value, the darker the colour).

**Table 17:** Effect size Cliff’s Delta (CD) for gender differences in the absolute and relative loss of propulsive joint work caused by eccentric knee flexor work; MV = mean of CD across footwear conditions; SD = standard deviation between CD of all footwear conditions; the higher the value, the darker the colour.

Loss of Propulsive Work	Individual Pace										10 km/h									
	BF	S1	S2	S3	S4	S5	S6	PS	MV	SD	BF	S1	S2	S3	S4	S5	S6	PS	MV	SD
Absolute	−0.10	−0.05	0.05	**0.17**	**0.29**	**0.23**	0.08	**0.30**	0.16	0.15	0.07	0.07	0.05	**0.22**	**0.24**	**0.30**	**0.20**	**0.32**	0.18	0.11
Relative	−0.12	−0.05	0.04	**0.17**	**0.28**	**0.21**	0.07	**0.33**	0.16	0.16	0.04	0.06	0.03	**0.20**	**0.20**	**0.27**	**0.17**	**0.31**	0.16	0.11

CD in bold—significant difference (*p* < 0.05); red cell—female > male (CD > 0); blue cell—female < male (CD < 0); light colour—small effect (|CD| > 0.11); medium colour—medium effect (|CD| > 0.28); dark colour—large effect (|CD| > 0.43); pink cell—mean CD (the higher the value, the darker the colour); green cell—standard deviation (the higher the value, the darker the colour).

**Table 18:** Effect size Cliff’s Delta (CD; shaded grey; the higher the value, the darker the colour) for gender differences in biomechanical variables averaged over time and categories; GRF = ground reaction force; JA = joint angle; JAV = joint angular velocity; JM = joint moment; JP = joint power; SA = segment angle; SAV = segment angular velocity; MV = mean of CD across footwear conditions (shaded pink; the higher the value, the darker the colour); SD = standard deviation between CD of all footwear conditions (shaded green; the higher the value, the darker the colour); higher values for the individual footwear conditions and for MV indicate greater gender differences; higher SD indicates larger variations in the gender differences across the footwear conditions.

Category	Individual Pace										10 km/h									
	BF	S1	S2	S3	S4	S5	S6	PS	MV	SD	BF	S1	S2	S3	S4	S5	S6	PS	MV	SD
**Variable**																				
GRF	0.152	0.127	0.142	0.188	0.208	0.154	0.150	0.164	0.161	0.100	0.253	0.230	0.240	0.220	0.195	0.209	0.248	0.203	0.225	0.093
JA	0.167	0.133	0.155	0.241	0.189	0.153	0.157	0.194	0.174	0.089	0.191	0.201	0.186	0.226	0.183	0.158	0.198	0.203	0.193	0.081
JAV	0.221	0.203	0.161	0.143	0.162	0.152	0.133	0.165	0.167	0.104	0.254	0.257	0.238	0.240	0.237	0.215	0.240	0.241	0.240	0.097
JM	0.123	0.104	0.091	0.141	0.155	0.123	0.082	0.122	0.117	0.098	0.188	0.177	0.142	0.144	0.141	0.145	0.169	0.145	0.156	0.086
JP	0.158	0.144	0.108	0.125	0.108	0.095	0.096	0.132	0.121	0.109	0.195	0.190	0.150	0.151	0.154	0.148	0.145	0.177	0.164	0.103
SA	0.212	0.172	0.203	0.192	0.175	0.181	0.176	0.213	0.191	0.089	0.235	0.236	0.241	0.208	0.209	0.193	0.218	0.230	0.221	0.076
SAV	0.194	0.177	0.170	0.181	0.192	0.178	0.186	0.183	0.183	0.097	0.222	0.222	0.202	0.211	0.209	0.187	0.206	0.218	0.210	0.089
**Joint**																				
Hip	0.210	0.201	0.172	0.165	0.176	0.165	0.158	0.172	0.177	0.095	0.231	0.245	0.214	0.219	0.230	0.212	0.220	0.237	0.226	0.084
Knee	0.175	0.138	0.137	0.189	0.171	0.132	0.121	0.181	0.155	0.111	0.192	0.173	0.152	0.163	0.139	0.140	0.166	0.165	0.161	0.097
Ankle	0.092	0.075	0.053	0.120	0.094	0.076	0.050	0.083	0.080	0.091	0.194	0.197	0.168	0.188	0.161	0.138	0.174	0.163	0.173	0.095
**Segment**																				
Pelvis	0.225	0.236	0.239	0.226	0.222	0.262	0.249	0.226	0.236	0.072	0.258	0.300	0.275	0.282	0.268	0.280	0.307	0.285	0.282	0.060
Thigh	0.245	0.174	0.177	0.182	0.198	0.154	0.164	0.193	0.186	0.093	0.248	0.233	0.222	0.250	0.238	0.197	0.221	0.239	0.231	0.081
Shank	0.115	0.101	0.146	0.169	0.146	0.131	0.132	0.177	0.140	0.090	0.164	0.161	0.179	0.158	0.146	0.139	0.155	0.182	0.161	0.080
Foot	0.237	0.195	0.181	0.161	0.160	0.166	0.180	0.197	0.185	0.130	0.251	0.217	0.205	0.118	0.171	0.121	0.141	0.172	0.175	0.123
**Plane**																				
Sagittal	0.199	0.161	0.159	0.169	0.154	0.146	0.143	0.158	0.161	0.096	0.245	0.237	0.210	0.211	0.188	0.198	0.205	0.197	0.211	0.090
Frontal	0.148	0.148	0.124	0.171	0.170	0.144	0.143	0.153	0.150	0.091	0.186	0.205	0.180	0.200	0.196	0.162	0.202	0.200	0.191	0.080
Transverse	0.199	0.164	0.175	0.178	0.175	0.164	0.145	0.210	0.176	0.106	0.212	0.201	0.199	0.180	0.189	0.166	0.184	0.219	0.194	0.094
**Total**																				
	0.180	0.156	0.152	0.173	0.168	0.151	0.144	0.171	0.162	0.097	0.218	0.216	0.199	0.199	0.191	0.178	0.201	0.205	0.201	0.088

**Table 20:** Gender differences in the perception of shoe characteristics; upper quartile (Q3), median (MED) and lower quartile (Q1) of female (f) and male (m) runners based on a visual analogue scale from 0 to 100 and the effect size Cliff’s Delta (CD) for gender differences.

Shoe Characteristics		BF		S1		S2		S3		S4		S5		S6		PS	
		f	m	f	m	f	m	f	m	f	m	f	m	f	m	f	m
General rating	Q3	-	-	31	46	21	50	63	70	71	73	86	80	72	81	98	89
	**MED**	**-**	**-**	**12**	**28**	**15**	**30**	**41**	**55**	**57**	**69**	**69**	**73**	**65**	**75**	**92**	**79**
	Q1	-	-	6	15	6	20	26	38	42	51	61	64	48	61	86	70
	CD	-		−0.29		**−0.51**		−0.22		−0.18		0.01		**−0.46**		**0.57**	
Comfort	Q3	-	-	26	35	28	53	74	77	76	75	86	85	70	79	100	90
	**MED**	**-**	**-**	**12**	**25**	**13**	**33**	**57**	**56**	**58**	**66**	**76**	**78**	**60**	**66**	**91**	**83**
	Q1	-	-	4	4	6	15	36	42	50	56	73	73	39	52	81	67
	CD	-		−0.20		**−0.40**		−0.09		−0.12		0.01		−0.29		0.33	
Support	Q3	-	-	40	38	51	59	31	65	64	66	73	73	58	66	93	84
	**MED**	**-**	**-**	**26**	**27**	**23**	**37**	**23**	**38**	**47**	**55**	**61**	**57**	**33**	**52**	**85**	**71**
	Q1	-	-	9	6	8	15	8	5	34	43	50	47	21	36	60	57
	CD	-		0.05		−0.22		−0.17		−0.23		0.06		−0.31		0.17	
Performance	Q3	-	-	26	35	23	38	48	69	62	78	87	79	72	84	99	89
	**MED**	**-**	**-**	**9**	**15**	**12**	**28**	**31**	**39**	**56**	**71**	**69**	**67**	**59**	**77**	**87**	**75**
	Q1	-	-	5	7	6	11	23	25	46	53	52	55	40	65	83	69
	CD	-		−0.15		−0.24		−0.15		**−0.44**		0.04		**−0.38**		0.32	
Energy return	Q3	-	-	8	18	22	43	40	46	63	74	82	73	85	81	88	78
	**MED**	**-**	**-**	**3**	**7**	**9**	**24**	**23**	**28**	**50**	**52**	**59**	**68**	**75**	**74**	**74**	**61**
	Q1	-	-	1	1	5	8	9	5	32	38	34	41	61	57	64	45
	CD	-		−0.16		−0.27		−0.06		−0.18		0.09		0.05		0.32	
Rearfoot cushioning	Q3	-	-	4	10	48	40	64	46	66	75	84	81	90	91	92	83
	**MED**	**-**	**-**	**1**	**3**	**24**	**21**	**47**	**31**	**60**	**64**	**72**	**67**	**77**	**77**	**83**	**76**
	Q1	-	-	0	1	5	7	33	13	43	57	59	50	62	68	70	60
	CD	-		−0.21		0.01		0.30		−0.26		0.18		−0.07		0.28	
Forefoot cushioning	Q3	-	-	4	10	34	48	58	43	55	75	77	75	87	82	80	72
	**MED**	**-**	**-**	**2**	**3**	**15**	**31**	**33**	**27**	**39**	**53**	**66**	**63**	**71**	**74**	**68**	**62**
	Q1	-	-	0	2	4	6	13	12	29	40	54	44	59	61	61	51
	CD	-		−0.35		−0.23		0.09		**−0.38**		0.10		−0.03		0.25	

CD in bold—significant difference (*p* < 0.05); red cell—female > male (CD > 0); blue cell—female < male (CD < 0); light colour—small effect (|CD| > 0.11); medium colour—medium effect (|CD| > 0.28); dark colour—large effect (|CD| > 0.43).

**Table 21:** Gender differences in the physical perception during running; upper quartile (Q3), median (MED) and lower quartile (Q1) of female (f) and male (m) runners based on a visual analogue scale from 0 to 100 and the effect size Cliff’s Delta (CD) for gender differences.

Physical Perception		BF		S1		S2		S3		S4		S5		S6		PS	
		f	m	f	m	f	m	f	m	f	m	f	m	f	m	f	m
Knee Load	Q3	68	63	78	54	73	57	68	58	42	45	45	35	32	41	41	36
	**MED**	**55**	**52**	**67**	**50**	**43**	**48**	**58**	**41**	**31**	**28**	**24**	**24**	**18**	**31**	**21**	**30**
	Q1	32	38	59	43	30	26	51	26	17	16	19	14	8	16	13	13
	CD	0.11		**0.56**		0.16		**0.40**		0.07		0.09		−0.15		−0.07	
Ankle Load	Q3	81	79	78	64	70	56	74	62	52	47	40	39	51	48	28	33
	**MED**	**63**	**62**	**70**	**57**	**51**	**49**	**57**	**48**	**36**	**27**	**29**	**25**	**34**	**32**	**18**	**23**
	Q1	49	48	56	47	34	32	30	28	27	23	15	15	16	15	9	16
	CD	0.05		**0.43**		0.16		0.20		0.20		0.05		0.10		−0.22	
Foot Load	Q3	97	80	95	75	78	62	75	64	57	50	48	44	50	48	44	39
	**MED**	**92**	**72**	**83**	**67**	**68**	**55**	**54**	**47**	**47**	**39**	**33**	**24**	**38**	**22**	**31**	**28**
	Q1	64	52	67	49	54	42	42	34	37	18	16	16	16	13	19	17
	CD	0.31		**0.50**		**0.37**		0.21		0.23		0.11		0.09		0.09	
Knee Stability	Q3	85	79	62	64	63	69	55	62	56	71	76	78	58	72	87	78
	**MED**	**73**	**59**	**39**	**52**	**44**	**45**	**41**	**47**	**51**	**61**	**70**	**67**	**48**	**59**	**77**	**67**
	Q1	53	45	24	33	26	30	28	34	41	53	46	49	37	35	54	61
	CD	0.17		−0.19		−0.04		−0.11		**−0.46**		−0.02		−0.30		0.14	
Ankle Stability	Q3	77	68	64	60	58	52	53	57	52	71	75	77	46	70	83	79
	**MED**	**44**	**48**	**39**	**52**	**30**	**35**	**34**	**41**	**47**	**62**	**66**	**64**	**39**	**44**	**75**	**66**
	Q1	24	34	18	33	11	16	20	18	34	51	40	48	15	37	64	56
	CD	−0.11		−0.11		−0.11		−0.12		**−0.46**		−0.07		**−0.38**		0.11	
Foot Stability	Q3	85	73	70	61	60	61	50	64	61	72	77	79	64	73	82	74
	**MED**	**61**	**52**	**41**	**49**	**42**	**35**	**36**	**45**	**52**	**60**	**68**	**70**	**53**	**60**	**72**	**65**
	Q1	22	38	15	30	10	17	14	17	44	46	44	57	26	45	54	56
	CD	−0.02		−0.08		−0.06		−0.21		−0.21		−0.14		−0.32		0.09	
Thigh Muscle Work	Q3	66	57	72	72	72	62	61	70	52	48	53	47	49	44	52	50
	**MED**	**50**	**49**	**54**	**57**	**49**	**50**	**53**	**48**	**47**	**43**	**45**	**37**	**29**	**34**	**37**	**41**
	Q1	24	35	43	46	42	41	39	37	33	33	23	29	19	22	16	31
	CD	0.01		−0.02		0.08		0.08		0.11		0.07		−0.04		−0.12	
Shank Muscle Work	Q3	91	75	90	76	78	74	68	61	66	58	50	53	51	62	60	59
	**MED**	**72**	**62**	**80**	**63**	**70**	**60**	**56**	**53**	**50**	**49**	**42**	**38**	**47**	**47**	**46**	**46**
	Q1	64	47	64	50	50	51	41	44	39	38	25	28	21	25	21	30
	CD	0.36		0.33		0.16		0.13		0.08		−0.06		−0.06		−0.01	
Foot Muscle Work	Q3	97	86	96	78	85	68	73	70	61	67	47	56	58	58	52	61
	**MED**	**91**	**73**	**92**	**71**	**79**	**62**	**64**	**58**	**51**	**51**	**38**	**36**	**46**	**41**	**40**	**46**
	Q1	85	48	80	60	55	49	45	39	41	36	20	25	23	19	22	32
	CD	**0.51**		**0.52**		**0.38**		0.18		−0.02		−0.12		0.08		−0.17	

CD in bold—significant difference (*p* < 0.05); red cell—female > male (CD > 0); blue cell—female < male (CD < 0); light colour—small effect (|CD| > 0.11); medium colour—medium effect (|CD| > 0.28); dark colour—large effect (|CD| > 0.43).

**Table 19:** Effect size Cliff’s Delta (CD; shaded grey; the higher the value, the darker the colour) for gender differences in biomechanical parameters averaged for categories; GRF = ground reaction force; JA = joint angle; JAV = joint angular velocity; JM = joint moment; JP = joint power; JAI = joint angular impulse; JW = joint work; SA = segment angle; SAV = segment angular velocity; MV = mean of CD across footwear conditions (shaded pink; the higher the value, the darker the colour); SD = standard deviation between CD of all footwear conditions (shaded green; the higher the value, the darker the colour); higher values for the individual footwear conditions and for MV indicate greater gender differences; higher SD indicates larger variations in the gender differences across the footwear conditions.

Category	Individual Pace										10 km/h									
	BF	S1	S2	S3	S4	S5	S6	PS	MV	SD	BF	S1	S2	S3	S4	S5	S6	PS	MV	SD
**Variable**																				
GRF	0.174	0.132	0.152	0.171	0.163	0.144	0.173	0.140	0.156	0.077	0.178	0.121	0.121	0.123	0.127	0.121	0.149	0.093	0.129	0.077
JA	0.197	0.194	0.188	0.224	0.205	0.191	0.191	0.202	0.199	0.075	0.207	0.217	0.206	0.222	0.217	0.208	0.230	0.220	0.216	0.068
JAV	0.234	0.217	0.150	0.171	0.217	0.185	0.185	0.177	0.192	0.087	0.307	0.301	0.310	0.284	0.319	0.308	0.331	0.283	0.305	0.084
JM	0.131	0.113	0.181	0.215	0.222	0.151	0.164	0.179	0.170	0.093	0.172	0.156	0.152	0.170	0.144	0.137	0.188	0.118	0.155	0.085
JP	0.178	0.162	0.163	0.176	0.174	0.138	0.158	0.151	0.163	0.090	0.214	0.186	0.184	0.151	0.185	0.156	0.175	0.156	0.176	0.084
JAI	0.150	0.125	0.160	0.177	0.189	0.162	0.133	0.164	0.157	0.077	0.171	0.135	0.157	0.160	0.153	0.157	0.159	0.127	0.152	0.069
JW	0.192	0.177	0.161	0.171	0.180	0.159	0.149	0.159	0.168	0.086	0.226	0.186	0.167	0.149	0.189	0.167	0.170	0.178	0.179	0.082
SA	0.196	0.193	0.186	0.214	0.193	0.194	0.191	0.196	0.195	0.069	0.206	0.217	0.196	0.211	0.211	0.205	0.207	0.210	0.208	0.065
SAV	0.222	0.200	0.186	0.127	0.170	0.179	0.158	0.185	0.178	0.092	0.243	0.243	0.262	0.171	0.229	0.208	0.215	0.232	0.225	0.085
**Joint**																				
Hip	0.175	0.181	0.184	0.198	0.179	0.165	0.187	0.182	0.181	0.080	0.229	0.228	0.203	0.211	0.212	0.199	0.218	0.216	0.214	0.074
Knee	0.187	0.188	0.198	0.209	0.232	0.186	0.174	0.213	0.198	0.085	0.194	0.182	0.220	0.196	0.219	0.203	0.204	0.211	0.204	0.075
Ankle	0.195	0.130	0.108	0.161	0.164	0.137	0.127	0.106	0.141	0.087	0.228	0.180	0.141	0.140	0.160	0.145	0.188	0.100	0.160	0.086
**Segment**																				
Pelvis	0.254	0.241	0.244	0.199	0.176	0.218	0.194	0.214	0.217	0.081	0.281	0.292	0.270	0.217	0.236	0.235	0.239	0.245	0.252	0.068
Thigh	0.198	0.177	0.177	0.211	0.207	0.198	0.184	0.210	0.195	0.069	0.214	0.233	0.229	0.265	0.260	0.245	0.253	0.273	0.247	0.064
Shank	0.173	0.197	0.176	0.201	0.207	0.180	0.195	0.202	0.192	0.086	0.182	0.187	0.202	0.185	0.203	0.182	0.190	0.198	0.191	0.079
Foot	0.176	0.147	0.129	0.138	0.144	0.153	0.143	0.124	0.144	0.059	0.169	0.158	0.116	0.105	0.136	0.139	0.127	0.112	0.133	0.068
**Plane**																				
Sagittal	0.191	0.177	0.177	0.191	0.166	0.179	0.154	0.165	0.175	0.083	0.215	0.195	0.195	0.181	0.177	0.190	0.184	0.147	0.186	0.079
Frontal	0.197	0.191	0.168	0.186	0.211	0.165	0.189	0.174	0.185	0.079	0.227	0.230	0.200	0.196	0.228	0.201	0.235	0.209	0.216	0.074
Transverse	0.184	0.168	0.183	0.201	0.205	0.181	0.176	0.210	0.188	0.080	0.202	0.197	0.208	0.202	0.219	0.190	0.201	0.243	0.208	0.070
**Total**																				
	0.187	0.172	0.174	0.188	0.189	0.168	0.169	0.175	0.178	0.082	0.208	0.196	0.183	0.175	0.187	0.177	0.187	0.174	0.186	0.077

## Data Availability

The data presented in this study are available on request from the first author to any qualified researcher who has obtained ethics approval for secondary use of existing data through a consent waiver.

## Appendix A. Biomechanical Variables for Standard Running Shoes

This section contains graphs of biomechanical variables vs. time during the right stance phase of running at 10 km/h with a standard running shoe (S5, Puma Velocity 2). Median curves and interquartile areas of the two groups of women and men are shown, as well as the course of the effect size Cliff’s Delta, as a measure of the direction and extent of the gender difference.

## Appendix B. Biomechanical Parameters for Standard Running Shoes

This section contains tables of biomechanical parameters during the right stance phase of running at individual pace and 10 km/h with a standard running shoe (S5, Puma Velocity 2). The median and quartiles of the two groups of women and men are given, as well as the effect size Cliff’s Delta, as a measure of the direction and extent of the gender difference.

**Table A1:** Gender differences in joint angle parameters when running with a standard running shoe (S5, Puma Velocity 2); sag = sagittal plane; fron = frontal plane; tran = transverse plane; FS = at foot strike; MS = at mid-stance; TO = at toe-off; MAX = maximum; MIN = minimum; ROM = range of motion.

			Joint Angle (deg)															
Joint	Plane	Par.	Individual Pace								10 km/h							
			Female			Male			*p*	CD	Female			Male			*p*	CD
			Q1	MED	Q3	Q1	MED	Q3			Q1	MED	Q3	Q1	MED	Q3		
Hip	sag	FS	36.13	**40.97**	46.82	32.80	**40.23**	44.05	**0.01**	**0.21**	35.85	**41.49**	45.26	30.41	**36.80**	41.36	**0.00**	**0.37**
		MS	24.69	**27.56**	32.72	21.28	**26.79**	30.08	**0.01**	**0.21**	24.36	**27.20**	32.27	19.70	**25.43**	28.36	**0.00**	**0.30**
+ flexion		TO	−5.92	**−2.56**	1.11	−7.92	**−3.63**	1.62	0.12	0.12	−3.36	**−1.18**	1.12	−8.35	**−1.98**	2.71	0.20	0.10
− extension		MAX	39.85	**43.11**	48.27	34.27	**41.73**	46.24	**0.01**	**0.21**	38.85	**42.14**	46.71	31.97	**38.41**	43.44	**0.00**	**0.35**
		MIN	−5.96	**−2.79**	1.11	−8.20	**−4.01**	0.85	0.10	0.13	−3.51	**−1.44**	1.10	−8.36	**−1.98**	2.69	0.23	0.09
		ROM	41.63	**45.43**	48.82	39.86	**44.34**	48.23	0.06	0.15	40.57	**43.76**	46.05	37.28	**39.82**	43.04	**0.00**	**0.43**
	fron	FS	5.24	**9.36**	11.67	1.95	**4.76**	7.81	**0.00**	**0.46**	5.61	**9.35**	11.94	1.83	**4.21**	9.25	**0.00**	**0.47**
		MS	9.69	**12.71**	16.94	5.04	**8.45**	12.34	**0.00**	**0.49**	9.91	**11.73**	16.53	4.28	**8.74**	11.67	**0.00**	**0.44**
+ adduction		TO	−2.58	**−0.20**	2.35	−3.92	**−0.85**	2.09	0.07	0.14	−2.34	**−0.11**	2.53	−2.74	**−0.17**	3.12	0.76	0.02
− abduction		MAX	12.96	**16.73**	21.10	7.53	**10.92**	14.92	**0.00**	**0.52**	13.23	**15.85**	20.58	7.47	**9.91**	14.33	**0.00**	**0.57**
		MIN	−3.09	**−0.41**	2.01	−4.59	**−1.57**	0.52	**0.00**	**0.23**	−2.46	**−0.24**	2.28	−2.86	**−0.86**	1.26	0.10	0.13
		ROM	13.42	**16.07**	20.81	10.21	**13.50**	16.25	**0.00**	**0.38**	13.40	**15.98**	19.70	9.39	**11.08**	14.50	**0.00**	**0.52**
	tran	FS	−11.95	**1.80**	9.36	−12.10	**−1.23**	6.61	0.14	0.12	−13.05	**−0.81**	9.12	−14.55	**−3.28**	7.33	0.08	0.14
		MS	−4.89	**4.57**	18.50	−10.44	**−3.73**	8.17	**0.00**	**0.31**	−5.45	**3.88**	19.09	−11.96	**−0.89**	7.34	**0.00**	**0.25**
+ internal rot.		TO	−7.61	**9.52**	22.81	−15.44	**−1.31**	9.51	**0.00**	**0.30**	−7.82	**8.05**	20.78	−18.13	**−3.82**	6.80	**0.00**	**0.33**
− external rot.		MAX	9.53	**20.23**	31.93	1.62	**11.08**	22.93	**0.00**	**0.35**	7.60	**18.62**	30.49	−1.49	**9.77**	18.60	**0.00**	**0.37**
		MIN	−16.62	**−7.70**	8.04	−20.62	**−11.13**	2.27	**0.02**	**0.19**	−16.93	**−8.52**	7.70	−21.60	**−11.05**	2.96	**0.02**	**0.19**
		ROM	18.82	**22.79**	29.58	15.40	**20.42**	26.03	**0.02**	**0.19**	18.55	**22.78**	29.13	14.43	**18.96**	23.97	**0.00**	**0.28**
Knee	sag	FS	−19.25	**−16.07**	−11.62	−19.19	**−16.39**	−12.98	0.71	0.03	−18.99	**−16.46**	−12.74	−18.21	**−16.46**	−12.96	0.73	−0.03
		MS	−45.91	**−44.03**	−41.37	−47.81	**−44.99**	−38.85	0.50	0.05	−46.69	**−44.15**	−40.78	−47.33	**−43.49**	−37.76	0.29	−0.08
+ extension		TO	−17.39	**−13.36**	−8.41	−20.76	**−16.91**	−12.39	**0.00**	**0.32**	−18.46	**−14.01**	−10.09	−22.81	**−18.28**	−12.27	**0.00**	**0.30**
− flexion		MAX	−14.73	**−11.33**	−7.89	−18.06	**−14.02**	−10.10	**0.00**	**0.30**	−15.55	**−12.56**	−9.44	−17.88	**−13.90**	−10.63	**0.00**	**0.23**
		MIN	−50.53	**−48.20**	−44.64	−50.76	**−47.25**	−42.65	0.73	−0.03	−51.04	**−47.32**	−44.13	−50.56	**−46.79**	−42.41	0.12	−0.12
		ROM	32.36	**35.09**	39.30	30.89	**32.83**	35.73	**0.00**	**0.33**	32.11	**35.79**	38.20	30.02	**31.52**	34.83	**0.00**	**0.41**
	fron	FS	−6.48	**−2.42**	0.22	−2.51	**−0.50**	2.87	**0.00**	**−0.32**	−6.78	**−2.80**	−0.60	−2.38	**−0.64**	3.08	**0.00**	**−0.38**
		MS	−6.32	**−1.70**	8.26	−9.40	**−2.42**	5.69	0.10	0.13	−6.50	**−3.48**	8.36	−8.27	**0.58**	6.48	0.37	0.07
+ adduction		TO	−4.53	**−0.51**	2.22	−5.42	**0.43**	3.16	0.62	−0.04	−5.10	**−0.22**	2.21	−5.59	**−0.19**	2.74	0.94	0.01
− abduction		MAX	2.13	**7.48**	14.12	0.26	**5.69**	14.16	0.19	0.10	0.41	**7.17**	12.74	−0.18	**5.29**	12.61	0.28	0.09
		MIN	−10.75	**−7.09**	−0.94	−13.48	**−5.89**	0.92	0.79	0.02	−11.07	**−8.00**	−0.86	−11.27	**−4.54**	1.40	0.36	−0.07
		ROM	10.90	**13.74**	17.51	9.87	**13.90**	16.28	0.18	0.11	11.28	**13.58**	16.81	8.52	**11.55**	14.79	**0.00**	**0.26**
	tran	MS	14.32	**17.38**	20.10	15.17	**16.96**	20.01	0.67	−0.03	13.12	**17.36**	20.46	14.03	**15.85**	17.88	0.08	0.14
		TO	−7.78	**−4.64**	−1.73	−3.82	**0.14**	2.94	**0.00**	**−0.48**	−6.75	**−4.31**	−2.15	−3.43	**0.47**	3.75	**0.00**	**−0.49**
+ internal rot.		MAX	15.24	**19.04**	22.12	17.70	**19.53**	22.76	**0.04**	**−0.17**	14.83	**18.76**	22.62	16.32	**18.03**	19.82	0.68	0.03
− external rot.		MIN	−8.91	**−5.77**	−3.22	−6.42	**−2.34**	−0.58	**0.00**	**−0.39**	−8.14	**−5.16**	−3.17	−5.79	**−2.64**	−0.58	**0.00**	**−0.38**
		ROM	21.43	**25.50**	29.27	21.41	**23.81**	26.43	0.10	0.13	21.42	**24.28**	28.91	19.56	**21.24**	23.98	**0.00**	**0.35**
Ankle	sag	FS	10.74	**14.22**	16.91	12.21	**15.25**	17.21	0.33	−0.08	10.83	**13.90**	16.56	11.53	**14.68**	17.36	0.36	−0.07
		MS	27.95	**29.76**	33.64	26.78	**29.99**	32.56	0.20	0.10	28.10	**30.27**	33.37	27.39	**29.80**	32.68	0.19	0.10
+ dorsiflexion		TO	−23.90	**−19.27**	−16.27	−22.75	**−18.15**	−14.52	0.13	−0.12	−23.27	**−19.66**	−16.82	−23.45	**−18.72**	−14.08	0.19	−0.10
− plantarflexion		MAX	28.57	**30.53**	34.37	27.27	**30.69**	33.31	0.25	0.09	28.81	**30.77**	33.70	27.77	**30.64**	32.82	0.14	0.12
		MIN	−23.90	**−19.27**	−16.27	−22.75	**−18.45**	−14.52	0.14	−0.12	−23.27	**−19.66**	−16.82	−23.45	**−18.72**	−14.08	0.19	−0.10
		ROM	47.98	**52.28**	55.83	45.11	**49.38**	53.26	**0.01**	**0.22**	48.73	**52.23**	55.31	44.84	**49.78**	53.83	**0.01**	**0.20**
	fron	FS	−2.51	**6.30**	15.12	−6.40	**0.71**	12.71	0.17	0.11	−2.12	**3.29**	14.76	−3.39	**−0.14**	13.05	0.42	0.06
		MS	−18.24	**−12.00**	−8.18	−15.97	**−12.18**	−4.21	**0.02**	**−0.18**	−19.23	**−11.85**	−8.33	−16.15	**−11.74**	−6.54	0.08	−0.14
+ supination		TO	−7.66	**−0.47**	7.87	−7.86	**0.16**	11.49	0.24	−0.09	−9.24	**−1.38**	10.42	−6.24	**0.02**	10.13	0.41	−0.07
− pronation		MAX	−2.00	**6.69**	16.52	−2.68	**3.18**	16.85	0.83	0.02	−1.04	**6.27**	15.84	−1.68	**2.23**	14.24	0.64	0.04
		MIN	−24.75	**−17.28**	−12.05	−20.75	**−17.60**	−9.13	0.06	−0.15	−22.70	**−16.98**	−11.37	−20.18	**−14.32**	−9.37	0.10	−0.13
		ROM	16.04	**22.25**	30.39	15.73	**21.67**	24.80	0.16	0.11	16.95	**21.62**	30.02	16.58	**21.47**	25.08	0.21	0.10

CD in bold—significant difference (*p* < 0.05); red cell—female > male (CD > 0); blue cell—female < male (CD < 0); light colour—small effect (|CD| > 0.11); medium colour—medium effect (|CD| > 0.28); dark colour—large effect (|CD| > 0.43).

**Table A2:** Gender differences in peak joint angular velocities when running with a standard running shoe (S5, Puma Velocity 2); sag = sagittal plane; fron = frontal plane; tran = transverse plane; FLEX = flexing; EXT = extending; ADD = adducting; ABD = abducting; IR = internally rotating; ER = externally rotating; DF = dorsiflexing; PF = plantarflexing; SUP = supinating; PRO = pronating.

			Peak Joint Angular Velocity (Deg/s)															
Joint	Plane	Dir.	Individual Pace								10 km/h							
			Female			Male			*p*	CD	Female			Male			*p*	CD
			Q1	MED	Q3	Q1	MED	Q3			Q1	MED	Q3	Q1	MED	Q3		
Hip	sag	FLEX	7.1	**40.3**	68.1	0.0	**22.6**	51.7	**0.03**	**0.17**	0.0	**36.6**	65.2	0.0	**27.4**	57.5	0.31	0.08
		EXT	270.1	**292.7**	322.8	276.9	**315.9**	333.8	0.07	−0.14	256.0	**277.9**	300.1	236.0	**253.9**	279.5	**0.00**	**0.37**
	fron	ADD	82.3	**125.8**	149.6	59.3	**89.6**	122.9	**0.00**	**0.35**	80.7	**110.8**	133.8	60.8	**79.9**	104.8	**0.00**	**0.43**
		ABD	104.4	**126.5**	163.6	81.8	**107.3**	135.2	**0.00**	**0.35**	99.7	**120.4**	158.5	70.3	**90.4**	118.7	**0.00**	**0.48**
	tran	IR	198.8	**284.3**	354.6	153.5	**216.6**	287.2	**0.00**	**0.30**	198.4	**300.3**	389.4	129.9	**172.8**	261.2	**0.00**	**0.41**
		ER	127.2	**208.5**	272.3	108.0	**152.9**	200.7	**0.00**	**0.27**	135.1	**188.9**	252.4	97.3	**122.8**	183.4	**0.00**	**0.36**
Knee	sag	EXT	283.6	**307.7**	346.6	254.3	**294.1**	325.2	**0.00**	**0.25**	267.0	**293.9**	321.0	220.3	**256.5**	272.0	**0.00**	**0.56**
		FLEX	406.9	**438.5**	469.1	413.3	**446.6**	474.5	0.42	−0.06	399.5	**432.0**	450.4	384.2	**407.7**	436.7	**0.00**	**0.23**
	fron	ADD	105.7	**155.7**	192.6	92.6	**129.9**	173.8	**0.01**	**0.21**	101.1	**144.1**	197.1	74.9	**105.8**	130.9	**0.00**	**0.39**
		ABD	107.6	**142.8**	184.1	96.8	**127.0**	164.5	**0.05**	**0.16**	107.1	**138.2**	181.4	77.5	**104.7**	141.6	**0.00**	**0.29**
	tran	IR	226.6	**271.0**	306.0	264.2	**282.8**	326.7	**0.00**	**−0.26**	221.3	**265.1**	294.2	225.3	**252.1**	286.5	0.65	0.04
		ER	199.9	**238.2**	279.5	187.2	**232.1**	266.4	0.17	0.11	191.9	**219.8**	262.6	150.6	**188.5**	211.7	**0.00**	**0.46**
Ankle	sag	DF	239.0	**267.7**	298.6	230.9	**251.6**	281.7	0.09	0.13	238.3	**258.5**	281.7	214.7	**236.7**	263.9	**0.00**	**0.28**
		PF	472.1	**528.3**	571.3	484.8	**522.8**	574.6	0.98	0.00	462.6	**495.4**	526.7	417.3	**472.0**	502.8	**0.00**	**0.32**
	fron	SUP	97.7	**152.4**	222.9	100.8	**131.2**	185.3	0.29	0.08	105.6	**156.9**	206.1	97.3	**136.3**	172.0	0.08	0.14
		PRO	202.5	**322.7**	439.2	233.3	**302.7**	371.1	0.36	0.07	203.8	**310.7**	405.9	208.9	**282.5**	326.4	**0.03**	**0.17**

CD in bold—significant difference (*p* < 0.05); red cell—female > male (CD > 0); blue cell—female < male (CD < 0); light colour—small effect (|CD| > 0.11); medium colour—medium effect (|CD| > 0.28); dark colour—large effect (|CD| > 0.43).

**Table A3:** Gender differences in peak external joint moments when running with a standard running shoe (S5, Puma Velocity 2); sag = sagittal plane; fron = frontal plane; tran = transverse plane; FLEX = tending to flex; EXT = tending to extend; ADD = tending to adduct; ABD = tending to abduct; IR = tending to internally rotate; ER = tending to externally rotate; DF = tending to dorsiflex; PF = tending to plantarflex; SUP = tending to supinate; PRO = tending to pronate; BW = body weight in N; BH = body height in m.

			External Joint Moment (N·m·BW^−1^·BH^−1^)															
Joint	Plane	Dir.	Individual Pace								10 km/h							
			Female			Male			*p*	CD	Female			Male			*p*	CD
			Q1	MED	Q3	Q1	MED	Q3			Q1	MED	Q3	Q1	MED	Q3		
Hip	sag	FLEX	6.5 × 10^−^^2^	**8.1 × 10^−^^2^**	1.1 × 10^−^^1^	7.7 × 10^−^^2^	**9.1 × 10^−^^2^**	1.2 × 10^−^^1^	**0.00**	**−0.23**	6.1 × 10^−^^2^	**7.7 × 10^−^^2^**	9.3 × 10^−^^2^	6.8 × 10^−^^2^	**7.9 × 10^−^^2^**	9.4 × 10^−^^2^	0.17	−0.11
		EXT	3.7 × 10^−^^2^	**5.4 × 10^−^^2^**	6.8 × 10^−^^2^	3.5 × 10^−^^2^	**4.9 × 10^−^^2^**	7.6 × 10^−^^2^	0.88	0.01	3.8 × 10^−^^2^	**4.9 × 10^−^^2^**	6.2 × 10^−^^2^	3.2 × 10^−^^2^	**3.9 × 10^−^^2^**	7.8 × 10^−^^2^	0.13	0.12
	fron	ADD	1.0 × 10^−^^1^	**1.5 × 10^−^^1^**	1.8 × 10^−^^1^	1.4 × 10^−^^1^	**1.7 × 10^−^^1^**	2.0 × 10^−^^1^	**0.00**	**−0.25**	1.0 × 10^−^^1^	**1.4 × 10^−^^1^**	1.8 × 10^−^^1^	1.1 × 10^−^^1^	**1.5 × 10^−^^1^**	1.9 × 10^−^^1^	0.16	−0.11
		ABD	0.0 × 10^0^	**1.2 × 10^−^^2^**	2.6 × 10^−^^2^	0.0 × 10^0^	**1.1 × 10^−^^2^**	2.8 × 10^−^^2^	0.66	−0.03	0.0 × 10^0^	**3.9 × 10^−^^3^**	2.0 × 10^−^^2^	0.0 × 10^0^	**5.0 × 10^−^^3^**	1.8 × 10^−^^2^	0.89	−0.01
	tran	IR	4.0 × 10^−^^2^	**5.9 × 10^−^^2^**	7.8 × 10^−^^2^	3.8 × 10^−^^2^	**5.9 × 10^−^^2^**	8.3 × 10^−^^2^	0.61	−0.04	3.7 × 10^−^^2^	**5.7 × 10^−^^2^**	7.1 × 10^−^^2^	3.4 × 10^−^^2^	**4.4 × 10^−^^2^**	6.5 × 10^−^^2^	**0.04**	**0.16**
		ER	0.0 × 10^0^	**3.5 × 10^−^^3^**	1.2 × 10^−^^2^	8.5 × 10^−^⁵	**6.0 × 10^−^^3^**	1.3 × 10^−^^2^	0.56	−0.05	0.0 × 10^0^	**1.8 × 10^−^^3^**	8.3 × 10^−^^3^	2.8 × 10^−^^4^	**2.3 × 10^−^^3^**	7.9 × 10^−^^3^	0.13	−0.12
Knee	sag	EXT	1.4 × 10^−^^2^	**2.3 × 10^−^^2^**	3.3 × 10^−^^2^	6.9 × 10^−^^3^	**1.4 × 10^−^^2^**	2.3 × 10^−^^2^	**0.00**	**0.36**	1.1 × 10^−^^2^	**2.1 × 10^−^^2^**	3.2 × 10^−^^2^	4.2 × 10^−^^3^	**9.5 × 10^−^^3^**	1.8 × 10^−^^2^	**0.00**	**0.39**
		FLEX	1.2 × 10^−^^1^	**1.5 × 10^−^^1^**	1.9 × 10^−^^1^	1.6 × 10^−^^1^	**1.8 × 10^−^^1^**	2.2 × 10^−^^1^	**0.00**	**−0.37**	1.2 × 10^−^^1^	**1.5 × 10^−^^1^**	1.8 × 10^−^^1^	1.4 × 10^−^^1^	**1.6 × 10^−^^1^**	2.0 × 10^−^^1^	**0.00**	**−0.23**
	fron	ADD	4.7 × 10^−^^2^	**7.7 × 10^−^^2^**	1.1 × 10^−^^1^	4.9 × 10^−^^2^	**7.3 × 10^−^^2^**	1.3 × 10^−^^1^	0.26	−0.09	4.5 × 10^−^^2^	**6.9 × 10^−^^2^**	1.1 × 10^−^^1^	4.8 × 10^−^^2^	**7.5 × 10^−^^2^**	1.1 × 10^−^^1^	0.65	−0.04
		ABD	1.4 × 10^−^⁷	**1.1 × 10^−^^2^**	3.4 × 10^−^^2^	0.0 × 10^0^	**9.4 × 10^−^^3^**	3.4 × 10^−^^2^	0.88	−0.01	0.0 × 10^0^	**4.3 × 10^−^^3^**	2.0 × 10^−^^2^	7.3 × 10^−^⁵	**4.5 × 10^−^^3^**	2.6 × 10^−^^2^	0.73	−0.03
	tran	IR	5.2 × 10^−^^4^	**1.9 × 10^−^^3^**	4.5 × 10^−^^3^	3.1 × 10^−^^4^	**1.6 × 10^−^^3^**	3.7 × 10^−^^3^	0.15	0.11	3.1 × 10^−^^4^	**1.7 × 10^−^^3^**	4.1 × 10^−^^3^	7.1 × 10^−^⁵	**7.8 × 10^−^^4^**	2.6 × 10^−^^3^	**0.04**	**0.16**
		ER	3.2 × 10^−^^2^	**4.3 × 10^−^^2^**	5.5 × 10^−^^2^	4.0 × 10^−^^2^	**5.1 × 10^−^^2^**	6.6 × 10^−^^2^	**0.00**	**−0.31**	3.0 × 10^−^^2^	**4.1 × 10^−^^2^**	5.3 × 10^−^^2^	3.1 × 10^−^^2^	**4.6 × 10^−^^2^**	5.8 × 10^−^^2^	0.20	−0.10
Ankle	sag	DF	1.4 × 10^−^^1^	**1.5 × 10^−^^1^**	1.6 × 10^−^^1^	1.3 × 10^−^^1^	**1.5 × 10^−^^1^**	1.6 × 10^−^^1^	0.11	0.13	1.3 × 10^−^^1^	**1.5 × 10^−^^1^**	1.6 × 10^−^^1^	1.2 × 10^−^^1^	**1.3 × 10^−^^1^**	1.5 × 10^−^^1^	**0.00**	**0.25**
		PF	4.0 × 10^−^^3^	**6.3 × 10^−^^3^**	1.1 × 10^−^^2^	4.6 × 10^−^^3^	**9.0 × 10^−^^3^**	2.0 × 10^−^^2^	**0.02**	**−0.19**	3.1 × 10^−^^3^	**6.7 × 10^−^^3^**	1.1 × 10^−^^2^	2.8 × 10^−^^3^	**7.8 × 10^−^^3^**	1.3 × 10^−^^2^	0.17	−0.11
	fron	SUP	3.6 × 10^−^⁵	**3.9 × 10^−^^3^**	1.0 × 10^−^^2^	1.5 × 10^−^^3^	**7.8 × 10^−^^3^**	1.3 × 10^−^^2^	**0.03**	**−0.17**	1.2 × 10^−^^4^	**3.5 × 10^−^^3^**	8.4 × 10^−^^3^	3.0 × 10^−^^4^	**5.7 × 10^−^^3^**	9.4 × 10^−^^3^	0.25	−0.09
		PRO	6.1 × 10^−^^2^	**7.3 × 10^−^^2^**	8.8 × 10^−^^2^	5.1 × 10^−^^2^	**6.5 × 10^−^^2^**	8.1 × 10^−^^2^	**0.00**	**0.23**	5.8 × 10^−^^2^	**7.1 × 10^−^^2^**	8.2 × 10^−^^2^	5.3 × 10^−^^2^	**6.4 × 10^−^^2^**	7.4 × 10^−^^2^	**0.02**	**0.19**

CD in bold—significant difference (*p* < 0.05); red cell—female > male (CD > 0); blue cell—female < male (CD < 0); light colour—small effect (|CD| > 0.11); medium colour—medium effect (|CD| > 0.28); dark colour—large effect (|CD| > 0.43).

**Table A4:** Gender differences in external joint angular impulses when running with a standard running shoe (S5, Puma Velocity 2); sag = sagittal plane; fron = frontal plane; tran = transverse plane; FLEX = tending to flex; EXT = tending to extend; ADD = tending to adduct; ABD = tending to abduct; IR = tending to internally rotate; ER = tending to externally rotate; DF = tending to dorsiflex; PF = tending to plantarflex; SUP = tending to supinate; PRO = tending to pronate; BW = body weight in N; BH = body height in m.

			External Joint Angular Impulse (N·m·s·BW^−1^·BH^−1^)															
Joint	Plane	Dir.	Individual Pace								10 km/h							
			Female			Male			*p*	CD	Female			Male			*p*	CD
			Q1	MED	Q3	Q1	MED	Q3			Q1	MED	Q3	Q1	MED	Q3		
Hip	sag	FLEX	1.8 × 10^−^^3^	**2.9 × 10^−^^3^**	3.9 × 10^−^^3^	1.8 × 10^−^^3^	**2.5 × 10^−^^3^**	3.1 × 10^−^^3^	0.05	0.16	1.9 × 10^−^^3^	**2.7 × 10^−^^3^**	4.0 × 10^−^^3^	1.7 × 10^−^^3^	**2.4 × 10^−^^3^**	2.9 × 10^−^^3^	0.06	0.15
		EXT	1.8 × 10^−^^3^	**4.0 × 10^−^^3^**	6.0 × 10^−^^3^	2.9 × 10^−^^3^	**4.2 × 10^−^^3^**	5.2 × 10^−^^3^	0.52	−0.05	1.8 × 10^−^^3^	**3.6 × 10^−^^3^**	6.3 × 10^−^^3^	2.7 × 10^−^^3^	**3.9 × 10^−^^3^**	5.4 × 10^−^^3^	1.00	0.00
	fron	ADD	1.3 × 10^−^^2^	**2.0 × 10^−^^2^**	2.7 × 10^−^^2^	1.5 × 10^−^^2^	**2.2 × 10^−^^2^**	2.8 × 10^−^^2^	0.09	−0.14	1.4 × 10^−^^2^	**1.9 × 10^−^^2^**	3.0 × 10^−^^2^	1.3 × 10^−^^2^	**2.1 × 10^−^^2^**	2.9 × 10^−^^2^	0.46	−0.06
		ABD	7.0 × 10^−^^5^	**2.1 × 10^−^^4^**	4.4 × 10^−^^4^	1.3 × 10^−^^4^	**2.5 × 10^−^^4^**	4.9 × 10^−^^4^	0.23	−0.10	4.0 × 10^−^^5^	**1.9 × 10^−^^4^**	4.2 × 10^−^^4^	8.4 × 10^−^^5^	**2.2 × 10^−^^4^**	4.5 × 10^−^^4^	0.23	−0.10
	tran	IR	3.9 × 10^−^^3^	**6.7 × 10^−^^3^**	9.2 × 10^−^^3^	3.3 × 10^−^^3^	**5.6 × 10^−^^3^**	8.2 × 10^−^^3^	0.15	0.12	4.5 × 10^−^^3^	**6.6 × 10^−^^3^**	8.8 × 10^−^^3^	3.0 × 10^−^^3^	**4.6 × 10^−^^3^**	7.3 × 10^−^^3^	**0.00**	**0.28**
		ER	2.1 × 10^−^^5^	**9.5 × 10^−^^5^**	2.1 × 10^−^^4^	4.0 × 10^−^^5^	**1.1 × 10^−^^4^**	3.6 × 10^−^^4^	0.08	−0.14	1.2 × 10^−^^5^	**7.1 × 10^−^^5^**	2.1 × 10^−^^4^	4.0 × 10^−^^5^	**1.2 × 10^−^^4^**	4.8 × 10^−^^4^	**0.01**	**−0.22**
Knee	sag	EXT	5.0 × 10^−^^4^	**8.6 × 10^−^^4^**	1.2 × 10^−^^3^	2.1 × 10^−^^4^	**4.5 × 10^−^^4^**	1.0 × 10^−^^3^	**0.00**	**0.32**	4.0 × 10^−^^4^	**8.1 × 10^−^^4^**	1.4 × 10^−^^3^	1.0 × 10^−^^4^	**4.1 × 10^−^^4^**	8.6 × 10^−^^4^	**0.00**	**0.33**
		FLEX	1.2 × 10^−^^2^	**1.7 × 10^−^^2^**	2.1 × 10^−^^2^	1.4 × 10^−^^2^	**1.9 × 10^−^^2^**	2.5 × 10^−^^2^	**0.00**	**−0.25**	1.2 × 10^−^^2^	**1.6 × 10^−^^2^**	2.2 × 10^−^^2^	1.5 × 10^−^^2^	**1.9 × 10^−^^2^**	2.5 × 10^−^^2^	**0.00**	**−0.25**
	fron	ADD	4.0 × 10^−^^3^	**9.8 × 10^−^^3^**	1.4 × 10^−^^2^	4.7 × 10^−^^3^	**8.9 × 10^−^^3^**	1.6 × 10^−^^2^	0.39	−0.07	4.2 × 10^−^^3^	**8.8 × 10^−^^3^**	1.4 × 10^−^^2^	5.1 × 10^−^^3^	**9.4 × 10^−^^3^**	1.5 × 10^−^^2^	0.44	−0.06
		ABD	7.2 × 10^−^^5^	**2.1 × 10^−^^4^**	7.7 × 10^−^^4^	8.2 × 10^−^^5^	**2.0 × 10^−^^4^**	7.2 × 10^−^^4^	0.87	0.01	6.6 × 10^−^^5^	**1.6 × 10^−^^4^**	5.6 × 10^−^^4^	8.0 × 10^−^^5^	**2.0 × 10^−^^4^**	6.8 × 10^−^^4^	0.38	−0.07
	tran	IR	2.6 × 10^−^^5^	**5.7 × 10^−^^5^**	1.4 × 10^−^^4^	1.1 × 10^−^^5^	**3.0 × 10^−^^5^**	7.2 × 10^−^^5^	**0.00**	**0.25**	2.0 × 10^−^^5^	**5.6 × 10^−^^5^**	9.7 × 10^−^^5^	7.0 × 10^−^^6^	**2.1 × 10^−^^5^**	5.9 × 10^−^^5^	**0.00**	**0.33**
		ER	3.0 × 10^−^^3^	**4.6 × 10^−^^3^**	6.2 × 10^−^^3^	3.7 × 10^−^^3^	**5.2 × 10^−^^3^**	6.9 × 10^−^^3^	**0.03**	**−0.17**	3.2 × 10^−^^3^	**4.7 × 10^−^^3^**	6.3 × 10^−^^3^	3.3 × 10^−^^3^	**5.3 × 10^−^^3^**	6.8 × 10^−^^3^	0.44	−0.06
Ankle	sag	DF	1.8 × 10^−^^2^	**2.0 × 10^−^^2^**	2.2 × 10^−^^2^	1.6 × 10^−^^2^	**1.8 × 10^−^^2^**	2.1 × 10^−^^2^	**0.00**	**0.27**	1.8 × 10^−^^2^	**2.0 × 10^−^^2^**	2.2 × 10^−^^2^	1.7 × 10^−^^2^	**1.9 × 10^−^^2^**	2.1 × 10^−^^2^	**0.01**	**0.20**
		PF	4.7 × 10^−^^5^	**9.1 × 10^−^^5^**	2.3 × 10^−^^4^	6.3 × 10^−^^5^	**1.6 × 10^−^^4^**	4.7 × 10^−^^4^	**0.01**	**−0.20**	3.8 × 10^−^^5^	**9.3 × 10^−^^5^**	2.1 × 10^−^^4^	3.9 × 10^−^^5^	**1.7 × 10^−^^4^**	3.5 × 10^−^^4^	**0.03**	**−0.17**
	fron	SUP	7.0 × 10^−^^6^	**6.5 × 10^−^^5^**	2.3 × 10^−^^4^	1.1 × 10^−^^5^	**1.6 × 10^−^^4^**	3.4 × 10^−^^4^	**0.02**	**−0.18**	1.4 × 10^−^^5^	**5.7 × 10^−^^5^**	2.1 × 10^−^^4^	4.7 × 10^−^^6^	**1.1 × 10^−^^4^**	2.8 × 10^−^^4^	0.13	−0.12
		PRO	7.2 × 10^−^^3^	**9.1 × 10^−^^3^**	1.2 × 10^−^^2^	5.6 × 10^−^^3^	**7.4 × 10^−^^3^**	9.7 × 10^−^^3^	**0.00**	**0.27**	6.9 × 10^−^^3^	**9.0 × 10^−^^3^**	1.1 × 10^−^^2^	6.4 × 10^−^^3^	**8.4 × 10^−^^3^**	1.0 × 10^−^^2^	0.17	0.11

CD in bold—significant difference (*p* < 0.05); red cell—female > male (CD > 0); blue cell—female < male (CD < 0); light colour—small effect (|CD| > 0.11); medium colour—medium effect (|CD| > 0.28); dark colour—large effect (|CD| > 0.43).

**Table A5:** Gender differences in peak joint powers when running with a standard running shoe (S5, Puma Velocity 2); sag = sagittal plane; fron = frontal plane; tran = transverse plane; FLEX = flexor; EXT = extensor; ADD = adductor; ABD = abductor; IR = internal rotator; ER = external rotator; DF = dorsiflexor; PF = plantar flexor; SUP = supinator; PRO = pronator; con = concentric; ecc = eccentric; BW = body weight in N; BH = body height in m.

				Peak Joint Power (W·BW^−1^·BH^−1^)															
				Individual Pace								10 km/h							
Joint	Plane	Dir.	Mov.	Female			Male			*p*	CD	Female			Male			*p*	CD
				Q1	MED	Q3	Q1	MED	Q3			Q1	MED	Q3	Q1	MED	Q3		
Hip	sag	FLEX	con	0.0 × 10^0^	**1.7 × 10^−^^3^**	2.3 × 10^−^^2^	0.0 × 10^0^	**0.0 × 10^0^**	8.0 × 10^−^^3^	**0.03**	**0.16**	0.0 × 10^0^	**3.0 × 10^−^^4^**	1.9 × 10^−^^2^	0.0 × 10^0^	**0.0 × 10^0^**	9.5 × 10^−^^3^	0.15	0.11
			ecc	8.8 × 10^−^^2^	**1.7 × 10^−^^1^**	2.5 × 10^−^^1^	1.6 × 10^−^^1^	**2.0 × 10^−^^1^**	2.6 × 10^−^^1^	**0.02**	**−0.19**	8.3 × 10^−^^2^	**1.6 × 10^−^^1^**	2.3 × 10^−^^1^	1.1 × 10^−^^1^	**1.5 × 10^−^^1^**	1.8 × 10^−^^1^	0.28	0.09
		EXT	con	4.0 × 10^−^^2^	**7.3 × 10^−^^2^**	1.2 × 10^−^^1^	4.5 × 10^−^^2^	**8.2 × 10^−^^2^**	1.2 × 10^−^^1^	0.51	−0.05	3.4 × 10^−^^2^	**6.4 × 10^−^^2^**	1.0 × 10^−^^1^	2.6 × 10^−^^2^	**5.0 × 10^−^^2^**	6.9 × 10^−^^2^	**0.04**	**0.16**
			ecc	1.3 × 10^−^^3^	**2.6 × 10^−^^2^**	5.7 × 10^−^^2^	0.0 × 10^0^	**2.4 × 10^−^^2^**	6.6 × 10^−^^2^	0.92	−0.01	0.0 × 10^0^	**2.0 × 10^−^^2^**	4.7 × 10^−^^2^	0.0 × 10^0^	**2.6 × 10^−^^2^**	6.3 × 10^−^^2^	0.33	−0.08
	fron	ADD	con	0.0 × 10^0^	**5.1 × 10^−^^3^**	2.6 × 10^−^^2^	0.0 × 10^0^	**4.0 × 10^−^^3^**	1.9 × 10^−^^2^	0.56	0.05	0.0 × 10^0^	**2.1 × 10^−^^3^**	1.4 × 10^−^^2^	0.0 × 10^0^	**0.0 × 10^0^**	1.9 × 10^−^^2^	0.49	0.05
			ecc	0.0 × 10^0^	**0.0 × 10^0^**	0.0 × 10^0^	0.0 × 10^0^	**0.0 × 10^0^**	0.0 × 10^0^	0.75	0.02	0.0 × 10^0^	**0.0 × 10^0^**	0.0 × 10^0^	0.0 × 10^0^	**0.0 × 10^0^**	0.0 × 10^0^	0.24	−0.06
		ABD	con	1.0 × 10^−^^1^	**1.9 × 10^−^^1^**	3.0 × 10^−^^1^	1.0 × 10^−^^1^	**1.7 × 10^−^^1^**	2.7 × 10^−^^1^	0.42	0.06	1.0 × 10^−^^1^	**1.9 × 10^−^^1^**	2.7 × 10^−^^1^	7.2 × 10^−^^2^	**1.2 × 10^−^^1^**	2.2 × 10^−^^1^	**0.00**	**0.26**
			ecc	1.2 × 10^−^^1^	**2.0 × 10^−^^1^**	3.4 × 10^−^^1^	1.2 × 10^−^^1^	**2.0 × 10^−^^1^**	2.8 × 10^−^^1^	0.38	0.07	1.0 × 10^−^^1^	**1.8 × 10^−^^1^**	2.9 × 10^−^^1^	9.6 × 10^−^^2^	**1.5 × 10^−^^1^**	2.3 × 10^−^^1^	**0.03**	**0.17**
	tran	IR	con	0.0 × 10^0^	**3.5 × 10^−^^3^**	3.4 × 10^−^^2^	0.0 × 10^0^	**3.0 × 10^−^^3^**	1.7 × 10^−^^2^	0.38	0.07	0.0 × 10^0^	**0.0 × 10^0^**	2.2 × 10^−^^2^	0.0 × 10^0^	**2.2 × 10^−^^3^**	1.0 × 10^−^^2^	0.42	−0.06
			ecc	0.0 × 10^0^	**0.0 × 10^0^**	1.9 × 10^−^^3^	0.0 × 10^0^	**0.0 × 10^0^**	7.3 × 10^−^^3^	0.14	−0.10	0.0 × 10^0^	**0.0 × 10^0^**	1.6 × 10^−^^3^	0.0 × 10^0^	**3.3 × 10^−^⁵**	5.7 × 10^−^^3^	**0.01**	**−0.20**
		ER	con	5.8 × 10^−^^2^	**1.0 × 10^−^^1^**	1.8 × 10^−^^1^	4.8 × 10^−^^2^	**8.7 × 10^−^^2^**	1.7 × 10^−^^1^	0.28	0.09	5.9 × 10^−^^2^	**1.1 × 10^−^^1^**	1.7 × 10^−^^1^	3.4 × 10^−^^2^	**5.8 × 10^−^^2^**	1.1 × 10^−^^1^	**0.00**	**0.36**
			ecc	6.8 × 10^−^^2^	**1.4 × 10^−^^1^**	2.2 × 10^−^^1^	4.5 × 10^−^^2^	**7.4 × 10^−^^2^**	1.5 × 10^−^^1^	**0.00**	**0.27**	6.7 × 10^−^^2^	**1.2 × 10^−^^1^**	1.9 × 10^−^^1^	3.3 × 10^−^^2^	**6.1 × 10^−^^2^**	1.1 × 10^−^^1^	**0.00**	**0.39**
Knee	sag	EXT	con	2.5 × 10^−^^1^	**3.8 × 10^−^^1^**	4.6 × 10^−^^1^	3.2 × 10^−^^1^	**4.0 × 10^−^^1^**	5.3 × 10^−^^1^	**0.04**	**−0.16**	2.4 × 10^−^^1^	**3.4 × 10^−^^1^**	4.3 × 10^−^^1^	2.7 × 10^−^^1^	**3.4 × 10^−^^1^**	4.2 × 10^−^^1^	0.64	−0.04
			ecc	6.1 × 10^−^^1^	**7.9 × 10^−^^1^**	9.8 × 10^−^^1^	7.5 × 10^−^^1^	**9.1 × 10^−^^1^**	1.1 × 10^0^	**0.00**	**−0.30**	5.9 × 10^−^^1^	**7.4 × 10^−^^1^**	8.7 × 10^−^^1^	6.4 × 10^−^^1^	**8.0 × 10^−^^1^**	9.1 × 10^−^^1^	0.06	−0.15
		FLEX	con	0.0 × 10^0^	**5.9 × 10^−^^2^**	1.2 × 10^−^^1^	0.0 × 10^0^	**4.6 × 10^−^⁵**	7.2 × 10^−^^2^	**0.01**	**0.21**	0.0 × 10^0^	**5.0 × 10^−^^2^**	1.3 × 10^−^^1^	0.0 × 10^0^	**0.0 × 10^0^**	5.6 × 10^−^^2^	**0.00**	**0.27**
			ecc	3.9 × 10^−^^2^	**8.0 × 10^−^^2^**	1.4 × 10^−^^1^	1.6 × 10^−^^2^	**4.8 × 10^−^^2^**	7.9 × 10^−^^2^	**0.00**	**0.33**	2.8 × 10^−^^2^	**7.4 × 10^−^^2^**	1.4 × 10^−^^1^	2.6 × 10^−^^3^	**2.7 × 10^−^^2^**	5.2 × 10^−^^2^	**0.00**	**0.44**
	fron	ADD	con	0.0 × 10^0^	**6.6 × 10^−^^3^**	3.2 × 10^−^^2^	0.0 × 10^0^	**1.8 × 10^−^^3^**	2.2 × 10^−^^2^	0.07	0.14	0.0 × 10^0^	**2.0 × 10^−^^3^**	3.1 × 10^−^^2^	0.0 × 10^0^	**0.0 × 10^0^**	1.0 × 10^−^^2^	**0.04**	**0.15**
			ecc	0.0 × 10^0^	**0.0 × 10^0^**	4.2 × 10^−^^2^	0.0 × 10^0^	**1.6 × 10^−^^3^**	3.2 × 10^−^^2^	0.24	−0.09	0.0 × 10^0^	**0.0 × 10^0^**	1.1 × 10^−^^2^	0.0 × 10^0^	**2.4 × 10^−^^4^**	2.3 × 10^−^^2^	0.27	−0.08
		ABD	con	5.6 × 10^−^^2^	**1.1 × 10^−^^1^**	1.8 × 10^−^^1^	6.1 × 10^−^^2^	**1.2 × 10^−^^1^**	2.1 × 10^−^^1^	0.40	−0.07	4.9 × 10^−^^2^	**1.0 × 10^−^^1^**	1.9 × 10^−^^1^	5.7 × 10^−^^2^	**8.7 × 10^−^^2^**	1.4 × 10^−^^1^	0.21	0.10
			ecc	4.9 × 10^−^^2^	**1.1 × 10^−^^1^**	2.2 × 10^−^^1^	4.8 × 10^−^^2^	**9.3 × 10^−^^2^**	2.3 × 10^−^^1^	0.72	0.03	4.5 × 10^−^^2^	**1.1 × 10^−^^1^**	1.9 × 10^−^^1^	5.2 × 10^−^^2^	**7.8 × 10^−^^2^**	1.4 × 10^−^^1^	0.06	0.15
	tran	IR	con	9.2 × 10^−^^2^	**1.4 × 10^−^^1^**	2.0 × 10^−^^1^	1.4 × 10^−^^1^	**1.8 × 10^−^^1^**	2.3 × 10^−^^1^	**0.00**	**−0.34**	9.3 × 10^−^^2^	**1.4 × 10^−^^1^**	1.7 × 10^−^^1^	1.0 × 10^−^^1^	**1.4 × 10^−^^1^**	1.7 × 10^−^^1^	0.77	−0.02
			ecc	2.4 × 10^−^^2^	**4.1 × 10^−^^2^**	8.6 × 10^−^^2^	3.4 × 10^−^^2^	**4.9 × 10^−^^2^**	7.2 × 10^−^^2^	0.35	−0.07	2.7 × 10^−^^2^	**4.1 × 10^−^^2^**	8.1 × 10^−^^2^	2.5 × 10^−^^2^	**3.6 × 10^−^^2^**	5.5 × 10^−^^2^	0.05	0.16
		ER	con	0.0 × 10^0^	**3.1 × 10^−^^3^**	6.5 × 10^−^^3^	0.0 × 10^0^	**0.0 × 10^0^**	2.1 × 10^−^^3^	**0.00**	**0.33**	0.0 × 10^0^	**1.7 × 10^−^^3^**	6.8 × 10^−^^3^	0.0 × 10^0^	**5.7 × 10^−^⁵**	1.3 × 10^−^^3^	**0.00**	**0.25**
			ecc	0.0 × 10^0^	**1.0 × 10^−^^3^**	6.6 × 10^−^^3^	0.0 × 10^0^	**2.1 × 10^−^^3^**	1.0 × 10^−^^2^	0.49	−0.05	0.0 × 10^0^	**4.1 × 10^−^^4^**	4.9 × 10^−^^3^	0.0 × 10^0^	**4.0 × 10^−^^4^**	5.4 × 10^−^^3^	0.90	0.01
Ankle	sag	DF	con	4.6 × 10^−^^4^	**3.7 × 10^−^^3^**	1.2 × 10^−^^2^	1.4 × 10^−^^3^	**5.6 × 10^−^^3^**	2.0 × 10^−^^2^	0.06	−0.15	4.3 × 10^−^^4^	**3.3 × 10^−^^3^**	1.3 × 10^−^^2^	2.1 × 10^−^^3^	**5.4 × 10^−^^3^**	1.2 × 10^−^^2^	0.21	−0.10
			ecc	0.0 × 10^0^	**2.1 × 10^−^^3^**	6.5 × 10^−^^3^	0.0 × 10^0^	**4.1 × 10^−^^3^**	1.6 × 10^−^^2^	**0.02**	**−0.18**	0.0 × 10^0^	**1.3 × 10^−^^3^**	5.6 × 10^−^^3^	0.0 × 10^0^	**1.9 × 10^−^^3^**	9.8 × 10^−^^3^	0.18	−0.10
		PF	con	5.7 × 10^−^^1^	**6.7 × 10^−^^1^**	7.8 × 10^−^^1^	5.7 × 10^−^^1^	**7.2 × 10^−^^1^**	8.6 × 10^−^^1^	0.14	−0.12	5.5 × 10^−^^1^	**6.3 × 10^−^^1^**	7.1 × 10^−^^1^	4.8 × 10^−^^1^	**5.8 × 10^−^^1^**	6.8 × 10^−^^1^	**0.01**	**0.20**
			ecc	3.1 × 10^−^^1^	**3.8 × 10^−^^1^**	4.5 × 10^−^^1^	2.4 × 10^−^^1^	**3.4 × 10^−^^1^**	4.4 × 10^−^^1^	0.13	0.12	2.8 × 10^−^^1^	**3.5 × 10^−^^1^**	3.9 × 10^−^^1^	2.2 × 10^−^^1^	**2.5 × 10^−^^1^**	4.0 × 10^−^^1^	**0.00**	**0.26**
	fron	SUP	con	5.8 × 10^−^^2^	**1.0 × 10^−^^1^**	1.7 × 10^−^^1^	7.7 × 10^−^^2^	**1.0 × 10^−^^1^**	1.3 × 10^−^^1^	0.55	0.05	6.0 × 10^−^^2^	**9.4 × 10^−^^2^**	1.4 × 10^−^^1^	6.0 × 10^−^^2^	**9.3 × 10^−^^2^**	1.1 × 10^−^^1^	0.24	0.09
			ecc	4.1 × 10^−^^2^	**8.9 × 10^−^^2^**	1.3 × 10^−^^1^	1.9 × 10^−^^2^	**5.1 × 10^−^^2^**	1.0 × 10^−^^1^	**0.00**	**0.26**	4.4 × 10^−^^2^	**7.2 × 10^−^^2^**	1.0 × 10^−^^1^	2.6 × 10^−^^2^	**4.5 × 10^−^^2^**	8.4 × 10^−^^2^	**0.00**	**0.23**
		PRO	con	0.0 × 10^0^	**1.6 × 10^−^^2^**	6.2 × 10^−^^2^	9.5 × 10^−^^4^	**2.8 × 10^−^^2^**	5.6 × 10^−^^2^	0.52	−0.05	1.4 × 10^−^^4^	**1.9 × 10^−^^2^**	3.9 × 10^−^^2^	0.0 × 10^0^	**2.3 × 10^−^^2^**	3.4 × 10^−^^2^	0.96	0.00
			ecc	0.0 × 10^0^	**0.0 × 10^0^**	0.0 × 10^0^	0.0 × 10^0^	**0.0 × 10^0^**	3.3 × 10^−^^3^	**0.01**	**−0.15**	0.0 × 10^0^	**0.0 × 10^0^**	0.0 × 10^0^	0.0 × 10^0^	**0.0 × 10^0^**	0.0 × 10^0^	**0.04**	**−0.11**

CD in bold—significant difference (*p* < 0.05); red cell—female > male (CD > 0); blue cell—female < male (CD < 0); light colour—small effect (|CD| > 0.11); medium colour—medium effect (|CD| > 0.28); dark colour—large effect (|CD| > 0.43).

**Table A6:** Gender differences in joint work when running with a standard running shoe (S5, Puma Velocity 2); sag = sagittal plane; fron = frontal plane; tran = transverse plane; FLEX = flexor; EXT = extensor; ADD = adductor; ABD = abductor; IR = internal rotator; ER = external rotator; DF = dorsiflexor; PF = plantar flexor; SUP = supinator; PRO = pronator; con = concentric; ecc = eccentric; BW = body weight in N; BH = body height in m.

				Joint Work (J·BW^−1^·BH^−1^)															
				Individual Pace								10 km/h							
Joint	Plane	Dir.	Mov.	Female			Male			*p*	CD	Female			Male			*p*	CD
				Q1	MED	Q3	Q1	MED	Q3			Q1	MED	Q3	Q1	MED	Q3		
Hip	sag	FLEX	con	0.0 × 10^0^	**7.7 × 10^−^^6^**	2.1 × 10^−^^4^	0.0 × 10^0^	**1.6 × 10^−^^6^**	6.0 × 10^−^⁵	0.06	0.15	0.0 × 10^0^	**2.0 × 10^−^^6^**	2.3 × 10^−^^4^	0.0 × 10^0^	**4.8 × 10^−^^6^**	9.6 × 10^−^⁵	0.46	0.06
			ecc	5.3 × 10^−^^3^	**1.3 × 10^−^^2^**	1.8 × 10^−^^2^	9.7 × 10^−^^3^	**1.4 × 10^−^^2^**	1.7 × 10^−^^2^	0.32	−0.08	4.7 × 10^−^^3^	**1.1 × 10^−^^2^**	1.8 × 10^−^^2^	6.9 × 10^−^^3^	**9.8 × 10^−^^3^**	1.4 × 10^−^^2^	0.31	0.08
		EXT	con	8.9 × 10^−^^4^	**2.6 × 10^−^^3^**	6.2 × 10^−^^3^	1.0 × 10^−^^3^	**2.0 × 10^−^^3^**	3.5 × 10^−^^3^	0.31	0.08	7.2 × 10^−^^4^	**2.5 × 10^−^^3^**	5.6 × 10^−^^3^	6.6 × 10^−^^4^	**1.7 × 10^−^^3^**	3.0 × 10^−^^3^	**0.03**	**0.17**
			ecc	1.9 × 10^−^⁵	**3.9 × 10^−^^4^**	9.4 × 10^−^^4^	0.0 × 10^0^	**3.6 × 10^−^^4^**	1.1 × 10^−^^3^	0.79	0.02	0.0 × 10^0^	**2.5 × 10^−^^4^**	8.6 × 10^−^^4^	0.0 × 10^0^	**4.1 × 10^−^^4^**	1.3 × 10^−^^3^	0.19	−0.10
	fron	ADD	con	1.0 × 10^−^⁵	**1.3 × 10^−^^4^**	3.2 × 10^−^^4^	9.4 × 10^−^^6^	**1.0 × 10^−^^4^**	3.0 × 10^−^^4^	0.71	0.03	1.2 × 10^−^⁵	**7.6 × 10^−^⁵**	2.6 × 10^−^^4^	2.7 × 10^−^^6^	**3.7 × 10^−^⁵**	2.1 × 10^−^^4^	0.30	0.08
			ecc	0.0 × 10^0^	**1.2 × 10^−^⁵**	7.9 × 10^−^⁵	6.4 × 10^−^⁹	**7.2 × 10^−^^6^**	1.2 × 10^−^^4^	0.83	−0.02	0.0 × 10^0^	**2.7 × 10^−^^6^**	5.2 × 10^−^⁵	1.2 × 10^−^⁷	**8.4 × 10^−^^6^**	5.7 × 10^−^⁵	0.39	−0.07
		ABD	con	8.1 × 10^−^^3^	**1.6 × 10^−^^2^**	2.5 × 10^−^^2^	7.5 × 10^−^^3^	**1.3 × 10^−^^2^**	2.2 × 10^−^^2^	0.34	0.08	9.2 × 10^−^^3^	**1.7 × 10^−^^2^**	2.6 × 10^−^^2^	4.5 × 10^−^^3^	**1.1 × 10^−^^2^**	1.9 × 10^−^^2^	**0.00**	**0.26**
			ecc	4.3 × 10^−^^3^	**8.9 × 10^−^^3^**	1.5 × 10^−^^2^	4.8 × 10^−^^3^	**8.1 × 10^−^^3^**	1.4 × 10^−^^2^	0.68	0.03	3.9 × 10^−^^3^	**8.0 × 10^−^^3^**	1.3 × 10^−^^2^	3.7 × 10^−^^3^	**7.0 × 10^−^^3^**	1.2 × 10^−^^2^	0.34	0.08
	tran	IR	con	9.4 × 10^−^^6^	**1.6 × 10^−^^4^**	5.5 × 10^−^^4^	3.7 × 10^−^^6^	**8.0 × 10^−^⁵**	3.3 × 10^−^^4^	0.11	0.13	1.4 × 10^−^^6^	**7.2 × 10^−^⁵**	4.1 × 10^−^^4^	4.6 × 10^−^^6^	**6.2 × 10^−^⁵**	2.2 × 10^−^^4^	0.56	0.05
			ecc	0.0 × 10^0^	**3.7 × 10^−^^6^**	7.8 × 10^−^⁵	6.1 × 10^−^^6^	**8.1 × 10^−^⁵**	3.3 × 10^−^^4^	**0.00**	**−0.36**	0.0 × 10^0^	**1.5 × 10^−^⁵**	8.4 × 10^−^⁵	5.1 × 10^−^^6^	**8.2 × 10^−^⁵**	2.9 × 10^−^^4^	**0.00**	**−0.31**
		ER	con	2.2 × 10^−^^3^	**4.0 × 10^−^^3^**	7.4 × 10^−^^3^	1.7 × 10^−^^3^	**3.4 × 10^−^^3^**	5.7 × 10^−^^3^	0.13	0.12	2.5 × 10^−^^3^	**4.3 × 10^−^^3^**	7.4 × 10^−^^3^	1.1 × 10^−^^3^	**2.6 × 10^−^^3^**	4.0 × 10^−^^3^	**0.00**	**0.34**
			ecc	3.0 × 10^−^^3^	**6.1 × 10^−^^3^**	1.2 × 10^−^^2^	1.6 × 10^−^^3^	**4.3 × 10^−^^3^**	9.0 × 10^−^^3^	**0.00**	**0.24**	3.9 × 10^−^^3^	**6.3 × 10^−^^3^**	9.7 × 10^−^^3^	1.2 × 10^−^^3^	**2.8 × 10^−^^3^**	5.7 × 10^−^^3^	**0.00**	**0.37**
Knee	sag	EXT	con	1.4 × 10^−^^2^	**2.2 × 10^−^^2^**	2.8 × 10^−^^2^	1.6 × 10^−^^2^	**2.2 × 10^−^^2^**	3.2 × 10^−^^2^	0.11	−0.13	1.2 × 10^−^^2^	**2.1 × 10^−^^2^**	2.8 × 10^−^^2^	1.6 × 10^−^^2^	**2.1 × 10^−^^2^**	2.9 × 10^−^^2^	0.22	−0.10
			ecc	2.2 × 10^−^^2^	**3.1 × 10^−^^2^**	4.1 × 10^−^^2^	3.0 × 10^−^^2^	**3.7 × 10^−^^2^**	4.9 × 10^−^^2^	**0.00**	**−0.27**	2.1 × 10^−^^2^	**2.9 × 10^−^^2^**	4.1 × 10^−^^2^	2.5 × 10^−^^2^	**3.3 × 10^−^^2^**	4.4 × 10^−^^2^	**0.02**	**−0.19**
		FLEX	con	1.4 × 10^−^^4^	**4.5 × 10^−^^4^**	1.3 × 10^−^^3^	4.8 × 10^−^⁵	**1.9 × 10^−^^4^**	5.5 × 10^−^^4^	**0.00**	**0.27**	1.7 × 10^−^^4^	**5.1 × 10^−^^4^**	1.4 × 10^−^^3^	7.2 × 10^−^⁵	**1.9 × 10^−^^4^**	4.9 × 10^−^^4^	**0.00**	**0.29**
			ecc	9.3 × 10^−^^4^	**2.3 × 10^−^^3^**	5.0 × 10^−^^3^	2.4 × 10^−^^4^	**1.4 × 10^−^^3^**	2.3 × 10^−^^3^	**0.00**	**0.34**	5.9 × 10^−^^4^	**2.3 × 10^−^^3^**	5.2 × 10^−^^3^	3.3 × 10^−^⁵	**7.2 × 10^−^^4^**	1.8 × 10^−^^3^	**0.00**	**0.39**
	fron	ADD	con	3.4 × 10^−^⁵	**1.4 × 10^−^^4^**	4.8 × 10^−^^4^	1.9 × 10^−^^6^	**5.9 × 10^−^⁵**	2.2 × 10^−^^4^	**0.00**	**0.25**	3.6 × 10^−^⁵	**9.1 × 10^−^⁵**	5.3 × 10^−^^4^	2.5 × 10^−^⁷	**3.0 × 10^−^⁵**	2.3 × 10^−^^4^	**0.00**	**0.29**
			ecc	7.6 × 10^−^⁹	**4.1 × 10^−^⁵**	7.6 × 10^−^^4^	1.8 × 10^−^⁵	**6.9 × 10^−^⁵**	3.4 × 10^−^^4^	0.11	−0.13	1.0 × 10^−^^6^	**2.4 × 10^−^⁵**	1.7 × 10^−^^4^	1.2 × 10^−^⁵	**5.2 × 10^−^⁵**	4.3 × 10^−^^4^	**0.02**	**−0.18**
		ABD	con	2.3 × 10^−^^3^	**4.7 × 10^−^^3^**	8.0 × 10^−^^3^	2.4 × 10^−^^3^	**5.0 × 10^−^^3^**	9.7 × 10^−^^3^	0.59	−0.04	1.9 × 10^−^^3^	**4.5 × 10^−^^3^**	8.0 × 10^−^^3^	2.0 × 10^−^^3^	**4.0 × 10^−^^3^**	6.6 × 10^−^^3^	0.43	0.06
			ecc	2.3 × 10^−^^3^	**5.5 × 10^−^^3^**	1.0 × 10^−^^2^	2.1 × 10^−^^3^	**4.3 × 10^−^^3^**	1.0 × 10^−^^2^	0.78	0.02	2.2 × 10^−^^3^	**5.5 × 10^−^^3^**	9.3 × 10^−^^3^	1.8 × 10^−^^3^	**4.1 × 10^−^^3^**	7.6 × 10^−^^3^	0.13	0.12
	tran	IR	con	4.0 × 10^−^^3^	**6.1 × 10^−^^3^**	8.5 × 10^−^^3^	5.1 × 10^−^^3^	**7.5 × 10^−^^3^**	9.9 × 10^−^^3^	**0.02**	**−0.19**	4.8 × 10^−^^3^	**6.4 × 10^−^^3^**	8.0 × 10^−^^3^	3.9 × 10^−^^3^	**5.9 × 10^−^^3^**	8.0 × 10^−^^3^	0.39	0.07
			ecc	1.5 × 10^−^^3^	**2.5 × 10^−^^3^**	4.5 × 10^−^^3^	2.0 × 10^−^^3^	**3.1 × 10^−^^3^**	4.5 × 10^−^^3^	0.16	−0.12	1.5 × 10^−^^3^	**2.6 × 10^−^^3^**	4.6 × 10^−^^3^	1.5 × 10^−^^3^	**2.4 × 10^−^^3^**	3.3 × 10^−^^3^	0.34	0.08
		ER	con	1.4 × 10^−^⁵	**8.8 × 10^−^⁵**	1.7 × 10^−^^4^	0.0 × 10^0^	**1.4 × 10^−^^6^**	2.8 × 10^−^⁵	**0.00**	**0.55**	7.5 × 10^−^^6^	**5.5 × 10^−^⁵**	1.8 × 10^−^^4^	0.0 × 10^0^	**3.1 × 10^−^^6^**	2.6 × 10^−^⁵	**0.00**	**0.46**
			ecc	3.1 × 10^−^^6^	**2.1 × 10^−^⁵**	8.4 × 10^−^⁵	5.8 × 10^−^^6^	**3.3 × 10^−^⁵**	1.1 × 10^−^^4^	0.31	−0.08	2.0 × 10^−^^6^	**1.8 × 10^−^⁵**	7.4 × 10^−^⁵	2.0 × 10^−^^6^	**1.2 × 10^−^⁵**	5.7 × 10^−^⁵	0.48	0.06
Ankle	sag	DF	con	1.6 × 10^−^^6^	**2.6 × 10^−^⁵**	1.1 × 10^−^^4^	8.6 × 10^−^^6^	**4.6 × 10^−^⁵**	2.4 × 10^−^^4^	0.06	−0.15	2.1 × 10^−^^6^	**2.3 × 10^−^⁵**	1.2 × 10^−^^4^	1.3 × 10^−^⁵	**4.6 × 10^−^⁵**	1.3 × 10^−^^4^	0.13	−0.12
			ecc	4.3 × 10^−^^6^	**4.1 × 10^−^⁵**	9.8 × 10^−^⁵	3.7 × 10^−^^6^	**6.6 × 10^−^⁵**	2.6 × 10^−^^4^	0.12	−0.13	4.3 × 10^−^^6^	**3.3 × 10^−^⁵**	8.7 × 10^−^⁵	7.7 × 10^−^⁷	**5.7 × 10^−^⁵**	1.6 × 10^−^^4^	0.47	−0.06
		PF	con	4.3 × 10^−^^2^	**5.0 × 10^−^^2^**	5.6 × 10^−^^2^	4.2 × 10^−^^2^	**4.7 × 10^−^^2^**	5.5 × 10^−^^2^	0.24	0.09	4.1 × 10^−^^2^	**4.9 × 10^−^^2^**	5.3 × 10^−^^2^	3.9 × 10^−^^2^	**4.3 × 10^−^^2^**	5.2 × 10^−^^2^	**0.02**	**0.18**
			ecc	1.9 × 10^−^^2^	**2.2 × 10^−^^2^**	2.6 × 10^−^^2^	1.5 × 10^−^^2^	**1.9 × 10^−^^2^**	2.4 × 10^−^^2^	**0.00**	**0.25**	1.8 × 10^−^^2^	**2.2 × 10^−^^2^**	2.4 × 10^−^^2^	1.3 × 10^−^^2^	**1.6 × 10^−^^2^**	2.3 × 10^−^^2^	**0.00**	**0.28**
	fron	SUP	con	4.2 × 10^−^^3^	**6.5 × 10^−^^3^**	1.1 × 10^−^^2^	4.6 × 10^−^^3^	**7.2 × 10^−^^3^**	1.0 × 10^−^^2^	0.78	0.02	4.0 × 10^−^^3^	**6.0 × 10^−^^3^**	9.2 × 10^−^^3^	4.5 × 10^−^^3^	**7.1 × 10^−^^3^**	9.0 × 10^−^^3^	0.62	−0.04
			ecc	1.7 × 10^−^^3^	**3.5 × 10^−^^3^**	5.5 × 10^−^^3^	4.7 × 10^−^^4^	**1.5 × 10^−^^3^**	3.3 × 10^−^^3^	**0.00**	**0.35**	1.7 × 10^−^^3^	**3.1 × 10^−^^3^**	4.5 × 10^−^^3^	8.3 × 10^−^^4^	**1.8 × 10^−^^3^**	3.4 × 10^−^^3^	**0.00**	**0.26**
		PRO	con	7.7 × 10^−^^6^	**2.0 × 10^−^^4^**	1.2 × 10^−^^3^	4.7 × 10^−^^6^	**6.4 × 10^−^^4^**	1.1 × 10^−^^3^	0.45	−0.06	1.3 × 10^−^⁵	**2.8 × 10^−^^4^**	7.6 × 10^−^^4^	4.7 × 10^−^^6^	**4.0 × 10^−^^4^**	8.7 × 10^−^^4^	0.60	−0.04
			ecc	0.0 × 10^0^	**0.0 × 10^0^**	9.2 × 10^−^^6^	0.0 × 10^0^	**0.0 × 10^0^**	4.3 × 10^−^⁵	0.45	−0.05	0.0 × 10^0^	**0.0 × 10^0^**	8.6 × 10^−^^6^	0.0 × 10^0^	**0.0 × 10^0^**	1.9 × 10^−^^6^	0.19	0.09

CD in bold—significant difference (*p* < 0.05); red cell—female > male (CD > 0); blue cell—female < male (CD < 0); light colour—small effect (|CD| > 0.11); medium colour—medium effect (|CD| > 0.28); dark colour—large effect (|CD| > 0.43).

**Table A7:** Gender differences in segment angle parameters when running with a standard running shoe (S5, Puma Velocity 2); sag = sagittal plane; fron = frontal plane; tran = transverse plane; FS = at foot strike; MS = at mid-stance; TO = at toe-off; MAX = maximum; MIN = minimum; ROM = range of motion; CW = clockwise; CCW = counterclockwise.

			Segment Orientation Angle (deg)															
			Individual Pace								10 km/h							
Joint	Plane	Par.	Female			Male			*p*	CD	Female			Male			*p*	CD
			Q1	MED	Q3	Q1	MED	Q3			Q1	MED	Q3	Q1	MED	Q3		
Pelvis	sag	FS	−23.15	**−18.88**	−15.65	−19.61	**−16.12**	−10.66	**0.00**	**−0.35**	−22.59	**−18.90**	−15.57	−18.22	**−15.85**	−8.47	**0.00**	**−0.40**
		MS	−21.89	**−17.21**	−15.17	−18.10	**−14.91**	−8.81	**0.00**	**−0.36**	−21.38	**−16.81**	−14.29	−16.58	**−12.89**	−7.68	**0.00**	**−0.45**
+ posterior tilt		TO	−25.41	**−19.95**	−17.47	−21.65	**−18.54**	−14.80	**0.00**	**−0.28**	−24.82	**−20.83**	−17.65	−22.57	**−17.25**	−13.51	**0.00**	**−0.35**
− anterior tilt		MAX	−18.80	**−15.37**	−12.61	−15.97	**−12.37**	−6.67	**0.00**	**−0.37**	−18.37	**−14.81**	−11.60	−13.81	**−10.94**	−5.68	**0.00**	**−0.44**
		MIN	−25.55	**−20.94**	−17.88	−22.57	**−19.20**	−14.78	**0.00**	**−0.28**	−24.99	**−21.04**	−18.50	−22.62	**−18.21**	−13.84	**0.00**	**−0.33**
		ROM	4.51	**6.03**	7.55	4.41	**6.08**	8.64	0.58	−0.04	5.16	**6.70**	8.04	5.23	**6.69**	9.08	0.57	−0.04
	fron	FS	−4.97	**−2.93**	−0.83	−3.38	**−2.12**	−0.12	**0.02**	**−0.19**	−4.64	**−2.63**	−1.06	−3.09	**−1.60**	0.56	**0.00**	**−0.24**
		MS	−7.01	**−4.92**	−3.00	−4.79	**−2.67**	0.04	**0.00**	**−0.37**	−6.64	**−4.48**	−2.74	−5.05	**−2.49**	0.61	**0.00**	**−0.36**
+ right side tilt		TO	1.69	**3.90**	6.01	1.76	**3.30**	5.19	0.49	0.06	1.50	**3.98**	6.23	0.16	**2.38**	4.43	**0.01**	**0.21**
− left side tilt		MAX	1.99	**4.00**	6.31	2.56	**3.67**	5.34	0.83	0.02	2.10	**4.13**	6.23	1.57	**3.16**	4.76	0.05	0.15
		MIN	−9.32	**−7.17**	−4.55	−7.22	**−4.96**	−2.17	**0.00**	**−0.28**	−8.83	**−6.76**	−4.58	−7.08	**−4.36**	−1.96	**0.00**	**−0.34**
		ROM	7.94	**10.19**	13.78	6.44	**8.83**	11.36	**0.00**	**0.27**	8.07	**10.53**	13.56	6.10	**7.66**	9.90	**0.00**	**0.44**
	tran	FS	−5.48	**−3.35**	−1.79	−3.79	**−0.57**	2.45	**0.00**	**−0.35**	−5.46	**−3.33**	−1.21	−5.27	**−1.20**	1.91	**0.01**	**−0.22**
		MS	−5.42	**−3.58**	−2.18	−4.56	**−2.34**	0.37	**0.00**	**−0.29**	−5.44	**−3.65**	−1.68	−4.30	**−1.78**	1.55	**0.00**	**−0.28**
+ CCW rotation		TO	−0.64	**1.01**	3.55	−2.65	**−0.51**	1.58	**0.00**	**0.32**	−1.05	**1.31**	4.00	−2.70	**0.01**	2.66	**0.01**	**0.21**
− CW rotation		MAX	−0.43	**1.50**	4.41	0.23	**1.98**	3.95	0.41	−0.07	−0.56	**1.96**	4.37	−0.31	**2.36**	4.28	0.80	−0.02
		MIN	−8.28	**−6.50**	−4.90	−6.56	**−4.61**	−2.32	**0.00**	**−0.37**	−8.25	**−6.52**	−4.83	−6.47	**−4.54**	−2.24	**0.00**	**−0.36**
		ROM	6.09	**7.48**	10.30	4.18	**6.38**	8.38	**0.00**	**0.30**	5.83	**7.65**	10.35	4.02	**6.29**	8.36	**0.00**	**0.30**
Thigh	sag	FS	20.12	**22.69**	24.79	19.83	**24.18**	26.04	0.17	−0.11	20.41	**21.98**	23.51	18.83	**22.12**	23.96	0.40	0.07
		MS	9.66	**11.32**	13.15	8.71	**10.81**	13.43	0.44	0.06	9.89	**11.76**	13.62	8.99	**10.80**	14.25	0.31	0.08
+ posterior tilt		TO	−24.08	**−22.28**	−20.18	−23.61	**−21.18**	−19.26	**0.02**	**−0.19**	−23.20	**−21.28**	−19.61	−21.86	**−19.25**	−16.93	**0.00**	**−0.34**
− anterior tilt		MAX	24.52	**26.96**	28.17	24.17	**27.01**	29.57	0.62	−0.04	24.52	**26.15**	27.43	23.21	**25.13**	27.67	0.11	0.13
		MIN	−24.48	**−22.37**	−20.49	−24.07	**−21.55**	−19.67	0.09	−0.14	−23.33	**−21.32**	−19.70	−21.86	**−19.25**	−16.93	**0.00**	**−0.35**
		ROM	46.05	**49.58**	51.99	45.02	**48.47**	51.04	0.09	0.13	45.21	**47.71**	50.44	42.07	**44.74**	47.47	**0.00**	**0.41**
	fron	FS	2.97	**4.76**	6.99	1.78	**3.54**	5.20	**0.00**	**0.23**	3.52	**4.99**	7.51	2.33	**3.84**	5.44	**0.00**	**0.31**
		MS	5.80	**6.98**	8.86	4.48	**6.27**	7.68	**0.02**	**0.19**	6.05	**6.81**	8.85	4.85	**6.15**	7.72	**0.01**	**0.22**
+ lateral tilt		TO	2.44	**6.51**	8.89	−0.77	**2.48**	6.12	**0.00**	**0.41**	2.56	**5.96**	8.73	0.08	**2.17**	5.51	**0.00**	**0.46**
− medial tilt		MAX	8.27	**9.45**	11.65	6.01	**8.13**	9.90	**0.00**	**0.35**	8.37	**9.79**	10.80	6.02	**7.99**	9.49	**0.00**	**0.45**
		MIN	0.48	**2.07**	3.10	−2.90	**0.66**	2.34	**0.00**	**0.28**	0.73	**2.43**	3.97	−1.07	**0.81**	2.36	**0.00**	**0.35**
		ROM	6.01	**7.78**	10.02	6.04	**7.61**	9.51	0.70	0.03	5.19	**7.20**	9.52	4.98	**7.05**	8.97	0.52	0.05
	tran	FS	−13.69	**−0.85**	10.78	−13.32	**−4.15**	7.11	0.13	0.12	−12.93	**−3.21**	10.85	−13.99	**−3.90**	5.63	0.11	0.13
		MS	−4.74	**3.66**	14.97	−13.44	**−6.21**	10.69	**0.00**	**0.29**	−5.82	**2.50**	15.12	−11.37	**−5.08**	10.69	**0.01**	**0.22**
+ internal rotation		TO	−5.46	**11.37**	22.21	−15.79	**−2.00**	9.27	**0.00**	**0.32**	−7.26	**10.59**	19.90	−14.15	**−2.86**	9.55	**0.00**	**0.32**
− external rotation		MAX	3.70	**16.52**	27.90	−5.34	**8.04**	20.24	**0.00**	**0.31**	3.31	**16.61**	24.34	−5.34	**6.02**	15.12	**0.00**	**0.34**
		MIN	−16.00	**−5.54**	9.80	−17.95	**−11.00**	3.50	**0.02**	**0.19**	−15.41	**−7.70**	9.48	−17.13	**−10.09**	3.63	**0.05**	**0.16**
		ROM	12.83	**16.44**	22.51	11.13	**15.72**	18.87	**0.03**	**0.17**	12.81	**16.76**	21.20	9.71	**13.12**	18.50	**0.00**	**0.30**
Shank	sag	FS	3.82	**6.10**	7.79	4.80	**6.59**	8.38	**0.04**	**−0.16**	3.22	**4.99**	6.65	3.20	**4.84**	6.63	0.84	0.02
		MS	−33.20	**−32.23**	−31.00	−34.08	**−31.66**	−30.11	0.37	−0.07	−33.03	**−31.88**	−30.88	−32.86	**−30.87**	−28.55	**0.00**	**−0.24**
+ posterior tilt		TO	−39.76	**−37.87**	−35.45	−43.46	**−40.10**	−36.81	**0.00**	**0.38**	−39.20	**−37.13**	−35.12	−41.71	**−38.38**	−34.50	0.08	0.14
− anterior tilt		MAX	3.81	**6.09**	7.78	4.80	**6.59**	8.38	**0.04**	**−0.17**	3.22	**4.99**	6.65	3.20	**4.84**	6.63	0.84	0.02
		MIN	−39.70	**−37.87**	−35.77	−43.41	**−40.03**	−36.79	**0.00**	**0.36**	−39.19	**−37.02**	−35.20	−41.71	**−38.38**	−34.50	0.10	0.13
		ROM	41.21	**43.31**	45.49	43.84	**47.05**	49.80	**0.00**	**−0.45**	40.44	**42.10**	45.13	40.13	**44.07**	46.84	**0.05**	**−0.16**
	fron	FS	1.70	**3.33**	4.68	4.02	**5.22**	5.79	**0.00**	**−0.45**	2.23	**3.43**	4.73	4.13	**5.03**	5.67	**0.00**	**−0.52**
		MS	5.41	**8.12**	10.11	6.30	**9.04**	11.23	0.28	−0.09	5.94	**8.31**	9.96	7.10	**9.31**	11.64	0.16	−0.11
+ lateral tilt		TO	−5.27	**−1.30**	2.88	−3.03	**−0.31**	4.19	0.34	−0.08	−4.45	**−0.44**	2.69	−2.51	**0.10**	3.77	0.19	−0.10
− medial tilt		MAX	6.91	**8.76**	10.37	6.86	**9.48**	11.80	0.64	−0.04	6.97	**8.83**	10.44	7.38	**9.55**	11.95	0.27	−0.09
		MIN	−5.33	**−1.32**	1.40	−3.50	**−0.36**	3.48	**0.04**	**−0.16**	−4.56	**−0.81**	1.90	−2.51	**0.10**	3.51	**0.01**	**−0.21**
		ROM	8.33	**11.38**	13.52	7.38	**8.89**	11.70	**0.00**	**0.29**	8.61	**11.16**	13.43	7.62	**9.64**	11.14	**0.00**	**0.31**
	tran	FS	−16.54	**−7.94**	−1.05	−17.62	**−8.41**	−0.29	0.87	−0.01	−17.02	**−8.68**	−1.15	−16.42	**−9.65**	−3.11	0.76	0.02
		MS	7.38	**13.30**	23.76	0.86	**10.83**	18.33	**0.01**	**0.21**	7.30	**11.01**	23.89	2.96	**12.24**	18.47	**0.03**	**0.18**
+ internal rotation		TO	−14.45	**−7.09**	6.13	−15.70	**−9.09**	0.52	0.11	0.13	−14.55	**−8.02**	6.35	−15.76	**−10.34**	1.00	0.06	0.15
− external rotation		MAX	9.10	**16.84**	28.27	4.63	**14.23**	20.56	**0.01**	**0.20**	8.69	**15.75**	27.57	5.53	**14.21**	20.10	**0.02**	**0.19**
		MIN	−17.07	**−9.84**	−1.65	−19.25	**−12.23**	−4.71	0.20	0.10	−19.49	**−9.69**	−1.32	−18.28	**−12.77**	−5.57	0.14	0.12
		ROM	20.55	**25.46**	30.12	20.78	**23.68**	27.51	0.19	0.10	21.17	**25.23**	29.97	22.04	**24.71**	29.16	0.99	0.00
Foot	Sag	FS	14.22	**18.25**	19.99	15.32	**21.10**	22.83	**0.00**	**−0.25**	13.56	**17.71**	19.27	14.19	**18.46**	20.88	0.11	−0.13
		MS	−8.29	**−7.41**	−6.62	−7.28	**−6.12**	−5.26	**0.00**	**−0.39**	−7.80	**−7.02**	−6.07	−6.85	**−5.50**	−4.62	**0.00**	**−0.45**
+ posterior tilt		TO	−59.36	**−56.19**	−53.02	−58.85	**−56.48**	−54.86	0.49	0.06	−58.06	**−55.91**	−52.64	−57.93	**−55.43**	−51.31	0.40	−0.07
− anterior tilt		MAX	14.22	**18.25**	19.99	15.32	**21.10**	22.83	**0.00**	**−0.25**	13.56	**17.71**	19.27	14.19	**18.46**	20.88	0.11	−0.13
		MIN	−59.36	**−56.19**	−53.02	−58.85	**−56.48**	−54.86	0.49	0.06	−58.06	**−55.91**	−52.64	−57.93	**−55.43**	−51.31	0.40	−0.07
		ROM	69.34	**74.27**	77.00	71.02	**75.19**	79.62	0.07	−0.15	67.58	**71.69**	76.25	66.36	**70.88**	75.91	0.40	0.07
	Tran	FS	−11.37	**−8.16**	−5.43	−13.15	**−10.75**	−7.37	**0.00**	**0.28**	−10.98	**−8.69**	−4.40	−13.72	**−9.98**	−6.53	**0.00**	**0.22**
		MS	−10.00	**−7.20**	−4.11	−10.53	**−8.55**	−5.51	0.07	0.15	−10.32	**−7.19**	−3.95	−11.08	**−8.58**	−6.00	0.05	0.15
+ internal rotation		TO	−11.21	**−6.33**	4.91	−11.81	**−7.21**	−0.20	0.15	0.11	−12.40	**−6.42**	5.02	−10.66	**−7.21**	−1.81	0.45	0.06
− external rotation		MAX	−7.47	**−2.78**	4.91	−6.88	**−4.23**	0.89	0.24	0.09	−7.72	**−3.31**	5.02	−7.78	**−4.68**	−0.39	0.12	0.12
		MIN	−12.82	**−9.36**	−6.28	−14.84	**−11.75**	−8.81	**0.00**	**0.26**	−14.06	**−9.57**	−5.97	−14.35	**−10.90**	−8.19	0.08	0.14
		ROM	4.80	**6.95**	10.53	5.62	**8.01**	11.30	0.08	−0.14	4.77	**7.61**	11.51	4.64	**6.27**	10.10	0.24	0.09

CD in bold—significant difference (*p* < 0.05); red cell—female > male (CD > 0); blue cell—female < male (CD < 0); light colour—small effect (|CD| > 0.11); medium colour—medium effect (|CD| > 0.28); dark colour—large effect (|CD| > 0.43).

**Table A8:** Gender differences in peak segment angular velocities when running with a standard running shoe (S5, Puma Velocity 2); sag = sagittal plane; fron = frontal plane; tran = transverse plane; POST = posterior tilting; ANT = anterior tilting; RIG = right side tilting; LEF = left side tilting; CCW = counterclockwise rotating; CW = clockwise rotating; LAT = lateral tilting; MED = medial tilting; IR = internally rotating; ER = externally rotating.

			Peak Segment Angular Velocity (Deg/s)															
Seg.	Plane	Dir.	Individual Pace								10 km/h							
			Female			Male			*p*	CD	Female			Male			*p*	CD
			Q1	MED	Q3	Q1	MED	Q3			Q1	MED	Q3	Q1	MED	Q3		
Pelvis	sag	POST	44.7	**59.3**	73.1	44.0	**58.4**	80.7	0.70	−0.03	48.5	**65.4**	80.1	49.9	**66.5**	90.5	0.67	−0.03
		ANT	45.0	**54.0**	67.9	42.6	**56.8**	68.6	0.97	0.00	43.7	**54.6**	71.2	43.9	**53.5**	69.1	0.93	−0.01
	fron	RIG	67.3	**92.9**	113.7	53.7	**73.5**	96.3	**0.00**	**0.30**	67.6	**88.7**	111.2	50.6	**62.7**	82.4	**0.00**	**0.48**
		LEF	41.8	**68.7**	93.9	23.5	**52.2**	80.8	**0.00**	**0.26**	42.9	**64.2**	84.2	31.9	**50.3**	70.0	**0.00**	**0.27**
	tran	CCW	49.2	**65.4**	82.1	23.2	**43.0**	73.3	**0.00**	**0.31**	49.3	**62.3**	81.5	26.0	**46.3**	67.8	**0.00**	**0.32**
		CW	36.0	**54.4**	74.2	27.9	**52.4**	70.5	0.26	0.09	29.0	**52.3**	72.4	18.3	**34.4**	71.2	**0.01**	**0.20**
Thigh	sag	POST	51.7	**73.3**	90.3	36.2	**63.6**	86.8	**0.02**	**0.19**	54.2	**73.1**	90.5	50.9	**72.2**	86.3	0.30	0.08
		ANT	300.6	**318.5**	343.3	294.0	**328.1**	352.8	0.47	−0.06	280.0	**306.4**	321.2	257.7	**282.2**	293.5	**0.00**	**0.53**
	fron	LAT	45.8	**73.6**	93.9	53.2	**75.3**	97.6	0.33	−0.08	43.6	**62.4**	89.1	39.7	**58.2**	81.4	0.16	0.11
		MED	30.8	**50.8**	69.9	36.8	**55.6**	83.3	0.11	−0.13	27.6	**47.9**	68.1	35.9	**50.4**	62.8	0.57	−0.05
	tran	IR	181.0	**264.9**	334.2	155.3	**207.7**	276.8	**0.00**	**0.27**	180.8	**253.3**	348.6	123.3	**177.0**	235.6	**0.00**	**0.39**
		ER	128.9	**189.4**	264.3	112.1	**149.3**	195.5	**0.00**	**0.26**	117.7	**166.5**	234.2	83.5	**107.1**	168.6	**0.00**	**0.39**
Shank	sag	POST	0.0	**0.1**	19.4	0.0	**0.0**	0.0	**0.00**	**0.36**	0.0	**3.3**	21.4	0.0	**0.0**	4.4	**0.00**	**0.30**
		ANT	350.5	**374.4**	390.7	371.9	**390.4**	406.6	**0.00**	**−0.28**	341.1	**352.7**	367.0	323.9	**336.7**	358.8	**0.00**	**0.30**
	fron	LAT	48.8	**65.8**	95.8	34.1	**53.0**	77.5	**0.00**	**0.28**	47.1	**66.8**	94.8	38.0	**50.1**	69.5	**0.00**	**0.33**
		MED	63.0	**84.7**	110.8	64.0	**82.9**	110.8	0.75	−0.03	66.0	**81.7**	105.1	60.0	**76.2**	93.4	**0.05**	**0.16**
	tran	IR	275.3	**351.2**	460.4	283.9	**347.4**	406.3	0.38	0.07	265.9	**324.7**	459.2	262.4	**310.6**	370.3	0.11	0.13
		ER	162.1	**201.2**	270.3	146.5	**189.9**	259.9	0.17	0.11	167.8	**205.6**	249.7	150.5	**193.8**	251.1	0.24	0.09
Foot	sag	POST	0.0	**0.0**	0.0	0.0	**0.0**	0.0	0.44	−0.02	0.0	**0.0**	0.0	0.0	**0.0**	0.0	0.41	−0.02
		ANT	443.9	**486.1**	520.8	478.4	**516.0**	558.9	**0.00**	**−0.31**	431.3	**461.0**	486.7	404.8	**440.5**	463.3	**0.00**	**0.31**
	tran	IR	38.5	**53.1**	97.0	51.6	**67.4**	99.3	**0.01**	**−0.21**	36.3	**50.6**	105.9	40.3	**53.8**	77.4	0.93	−0.01
		ER	8.7	**17.0**	38.6	7.5	**18.2**	54.0	0.56	−0.05	8.9	**15.8**	38.2	4.9	**13.9**	29.3	0.05	0.15

CD in bold—significant difference (*p* < 0.05); red cell—female > male (CD > 0); blue cell—female < male (CD < 0); light colour—small effect (|CD| > 0.11); medium colour—medium effect (|CD| > 0.28); dark colour—large effect (|CD| > 0.43).

## Appendix C. Biomechanical Variables for Various Footwear Conditions

This section contains graphs of biomechanical variables vs. time during the right stance phase of running at individual pace and 10 km/h using eight footwear conditions (BF: Barefoot; S1: Vivobarefoot Primus Lite III; S2: Joe Nimble Addict; S3: Nike Free Run 5.0; S4: Puma Liberate 2; S5: Puma Velocity 2; S6: Puma Deviate Elite 2; PS: Personal shoe). Median curves and interquartile areas of the two groups of women and men are shown, as well as the course of the Cliff’s Delta, as a measure of the direction and extent of the gender difference.

## Appendix D. Gender Differences in Biomechanical Variables for Various Footwear Conditions

This section contains graphs of the effect size Cliff’s Delta regarding gender differences in biomechanical variables vs. time during the right stance phase of running at individual pace and 10 km/h using eight footwear conditions (BF: Barefoot; S1: Vivobarefoot Primus Lite III; S2: Joe Nimble Addict; S3: Nike Free Run 5.0; S4: Puma Liberate 2; S5: Puma Velocity 2; S6: Puma Deviate Elite 2; PS: Personal shoe). The course of Cliff’s Delta is a measure of the direction and extent of the gender difference while the course of the standard deviation of Cliff’s Delta indicates the variability of the gender difference with respect to the footwear condition.

**Table A9:** Effect size Cliff’s Delta (CD; shaded grey; the higher the value, the darker the colour) for gender differences in single biomechanical variables averaged over time; GRF = ground reaction force; JA = joint angle; JAV = joint angular velocity; JM = joint moment; JP = joint power; SA = segment angle; SAV = segment angular velocity; sag = sagittal plane; fron = frontal plane; tran = transverse plane; MV = mean of CD across footwear conditions (shaded pink; the higher the value, the darker the colour); SD = standard deviation between CD of all footwear conditions (shaded green; the higher the value, the darker the colour); higher values for the individual footwear conditions and for MV indicate greater gender differences; higher SD indicates larger variations in gender differences across the footwear conditions.

Var.	Joint/ Segment	Dir./Plane	Individual Pace										10 km/h									
			BF	S1	S2	S3	S4	S5	S6	PS	MV	SD	BF	S1	S2	S3	S4	S5	S6	PS	MV	SD
GRF		M/L	0.07	0.06	0.04	0.18	0.23	0.15	0.05	0.12	0.112	0.114	0.07	0.08	0.12	0.09	0.07	0.08	0.24	0.03	0.098	0.098
		A/P	0.12	0.12	0.19	0.20	0.18	0.13	0.20	0.19	0.165	0.099	0.31	0.29	0.33	0.28	0.27	0.28	0.28	0.31	0.295	0.098
		Vert	0.27	0.20	0.20	0.18	0.21	0.19	0.20	0.19	0.205	0.088	0.37	0.33	0.27	0.28	0.24	0.27	0.22	0.27	0.281	0.083
JA	Hip	sag	0.37	0.24	0.22	0.24	0.16	0.22	0.21	0.14	0.227	0.080	0.36	0.36	0.29	0.35	0.26	0.32	0.35	0.22	0.312	0.065
		fron	0.38	0.37	0.36	0.44	0.42	0.42	0.49	0.44	0.415	0.053	0.44	0.49	0.44	0.45	0.46	0.41	0.55	0.53	0.470	0.066
		tran	0.22	0.17	0.34	0.41	0.31	0.28	0.26	0.41	0.301	0.097	0.20	0.21	0.30	0.36	0.30	0.27	0.28	0.39	0.289	0.079
	Knee	sag	0.09	0.04	0.10	0.10	0.03	0.06	0.00	0.10	0.065	0.079	0.19	0.16	0.10	0.07	0.10	0.07	0.10	0.08	0.110	0.100
		fron	0.09	0.09	0.09	0.14	0.03	0.07	0.07	0.07	0.080	0.091	0.05	0.08	0.03	0.08	0.04	0.02	0.05	0.06	0.053	0.055
		tran	0.09	0.09	0.08	0.16	0.29	0.14	0.24	0.22	0.164	0.110	0.16	0.13	0.08	0.10	0.10	0.09	0.13	0.10	0.113	0.090
	Ankle	sag	0.03	0.02	0.03	0.15	0.10	0.00	0.00	0.03	0.046	0.085	0.10	0.14	0.18	0.17	0.09	0.08	0.09	0.09	0.116	0.094
		fron	0.05	0.03	0.03	0.29	0.16	0.04	0.00	0.13	0.092	0.119	0.02	0.04	0.06	0.24	0.11	0.01	0.03	0.14	0.082	0.096
JAV	Hip	sag	0.30	0.27	0.23	0.13	0.23	0.18	0.17	0.19	0.213	0.107	0.29	0.28	0.27	0.28	0.29	0.24	0.27	0.28	0.275	0.083
		fron	0.19	0.26	0.20	0.18	0.24	0.24	0.23	0.20	0.216	0.086	0.29	0.43	0.36	0.38	0.40	0.38	0.36	0.31	0.365	0.081
		tran	0.20	0.18	0.10	0.05	0.07	0.11	0.04	0.11	0.108	0.108	0.16	0.13	0.13	0.08	0.16	0.06	0.11	0.14	0.121	0.100
	Knee	sag	0.33	0.20	0.16	0.15	0.21	0.10	0.09	0.18	0.178	0.127	0.36	0.34	0.32	0.34	0.29	0.31	0.32	0.32	0.324	0.105
		fron	0.24	0.22	0.25	0.29	0.22	0.23	0.22	0.26	0.241	0.114	0.22	0.19	0.17	0.25	0.20	0.14	0.20	0.23	0.198	0.097
		tran	0.20	0.24	0.14	0.16	0.19	0.20	0.15	0.18	0.183	0.111	0.21	0.24	0.19	0.24	0.16	0.26	0.25	0.26	0.228	0.108
	Ankle	sag	0.18	0.12	0.10	0.08	0.07	0.11	0.09	0.12	0.108	0.092	0.34	0.29	0.29	0.22	0.23	0.22	0.24	0.24	0.259	0.108
		fron	0.13	0.14	0.11	0.09	0.06	0.06	0.07	0.09	0.093	0.085	0.16	0.16	0.18	0.14	0.15	0.10	0.17	0.15	0.152	0.095
JM	Hip	sag	0.10	0.11	0.08	0.09	0.08	0.09	0.05	0.07	0.084	0.094	0.12	0.12	0.06	0.05	0.07	0.09	0.03	0.09	0.078	0.084
		fron	0.06	0.06	0.04	0.11	0.20	0.12	0.04	0.09	0.091	0.098	0.13	0.10	0.11	0.08	0.08	0.10	0.19	0.09	0.110	0.082
		tran	0.28	0.26	0.21	0.12	0.13	0.13	0.19	0.15	0.184	0.094	0.34	0.33	0.28	0.27	0.27	0.30	0.22	0.33	0.291	0.085
	Knee	sag	0.24	0.18	0.27	0.34	0.21	0.20	0.21	0.34	0.248	0.113	0.23	0.17	0.21	0.24	0.16	0.18	0.16	0.21	0.194	0.095
		fron	0.04	0.06	0.02	0.06	0.16	0.02	0.02	0.03	0.051	0.081	0.07	0.06	0.03	0.04	0.02	0.02	0.08	0.03	0.043	0.058
		tran	0.18	0.08	0.09	0.21	0.26	0.17	0.08	0.19	0.157	0.137	0.19	0.17	0.17	0.10	0.18	0.13	0.21	0.14	0.160	0.110
	Ankle	sag	0.04	0.07	0.00	0.02	0.04	0.08	0.06	0.03	0.043	0.058	0.30	0.29	0.15	0.19	0.18	0.24	0.24	0.16	0.219	0.087
		fron	0.04	0.02	0.02	0.17	0.16	0.16	0.01	0.07	0.082	0.106	0.13	0.18	0.14	0.19	0.16	0.11	0.22	0.11	0.155	0.084
JP	Hip	sag	0.13	0.15	0.10	0.12	0.15	0.09	0.09	0.10	0.117	0.110	0.12	0.13	0.08	0.08	0.12	0.10	0.10	0.10	0.103	0.085
		fron	0.09	0.18	0.07	0.01	0.05	0.02	0.08	0.04	0.067	0.098	0.18	0.25	0.17	0.20	0.21	0.23	0.11	0.23	0.199	0.109
		tran	0.18	0.17	0.12	0.07	0.06	0.09	0.05	0.11	0.106	0.117	0.14	0.13	0.09	0.06	0.11	0.05	0.07	0.13	0.098	0.090
	Knee	sag	0.24	0.15	0.23	0.30	0.21	0.18	0.16	0.30	0.222	0.129	0.25	0.20	0.23	0.22	0.20	0.19	0.19	0.22	0.212	0.117
		fron	0.21	0.16	0.13	0.19	0.08	0.08	0.13	0.13	0.139	0.104	0.18	0.14	0.11	0.15	0.10	0.08	0.14	0.15	0.133	0.103
		tran	0.15	0.15	0.08	0.17	0.15	0.13	0.09	0.17	0.137	0.133	0.19	0.21	0.17	0.13	0.12	0.18	0.15	0.19	0.167	0.132
	Ankle	sag	0.11	0.09	0.06	0.06	0.09	0.11	0.08	0.11	0.089	0.085	0.29	0.30	0.23	0.27	0.27	0.30	0.27	0.26	0.273	0.095
		fron	0.15	0.11	0.07	0.08	0.07	0.06	0.08	0.09	0.090	0.094	0.20	0.17	0.12	0.09	0.10	0.05	0.13	0.15	0.128	0.099
SA	Pelvis	sag	0.47	0.38	0.36	0.43	0.26	0.35	0.32	0.34	0.363	0.065	0.43	0.43	0.36	0.46	0.33	0.41	0.38	0.35	0.394	0.048
		fron	0.12	0.16	0.10	0.18	0.24	0.24	0.23	0.19	0.183	0.075	0.17	0.31	0.27	0.26	0.30	0.26	0.34	0.31	0.278	0.073
		tran	0.19	0.22	0.30	0.25	0.24	0.30	0.28	0.26	0.255	0.074	0.27	0.25	0.24	0.23	0.20	0.24	0.26	0.29	0.246	0.055
	Thigh	sag	0.14	0.05	0.12	0.03	0.06	0.01	0.02	0.02	0.056	0.079	0.20	0.22	0.16	0.16	0.14	0.13	0.19	0.13	0.165	0.076
		fron	0.31	0.31	0.20	0.23	0.30	0.22	0.32	0.23	0.265	0.090	0.38	0.35	0.31	0.31	0.33	0.29	0.28	0.27	0.315	0.067
		tran	0.28	0.14	0.29	0.38	0.28	0.25	0.27	0.34	0.280	0.078	0.20	0.20	0.26	0.35	0.30	0.24	0.27	0.36	0.273	0.069
	Shank	sag	0.03	0.07	0.24	0.10	0.07	0.08	0.12	0.12	0.104	0.103	0.09	0.06	0.24	0.04	0.03	0.05	0.10	0.10	0.089	0.111
		fron	0.07	0.08	0.13	0.09	0.12	0.11	0.15	0.16	0.115	0.045	0.12	0.08	0.12	0.10	0.13	0.14	0.16	0.13	0.123	0.028
		tran	0.19	0.04	0.14	0.27	0.16	0.13	0.03	0.26	0.152	0.098	0.16	0.14	0.17	0.26	0.18	0.14	0.12	0.25	0.178	0.061
	Foot	sag	0.29	0.21	0.06	0.13	0.16	0.22	0.18	0.12	0.171	0.128	0.28	0.24	0.15	0.11	0.17	0.15	0.14	0.12	0.169	0.119
		tran	0.24	0.23	0.30	0.04	0.01	0.08	0.02	0.29	0.152	0.145	0.28	0.31	0.38	0.00	0.20	0.08	0.15	0.23	0.203	0.134
SAV	Pelvis	sag	0.10	0.17	0.13	0.06	0.08	0.12	0.13	0.13	0.117	0.063	0.08	0.12	0.12	0.08	0.11	0.10	0.14	0.13	0.111	0.041
		fron	0.21	0.26	0.21	0.17	0.22	0.24	0.22	0.12	0.206	0.086	0.28	0.36	0.33	0.30	0.38	0.33	0.32	0.30	0.326	0.074
		tran	0.26	0.24	0.33	0.28	0.28	0.33	0.32	0.30	0.291	0.070	0.32	0.34	0.33	0.36	0.28	0.34	0.40	0.33	0.337	0.072
	Thigh	sag	0.31	0.26	0.20	0.17	0.25	0.16	0.17	0.18	0.212	0.102	0.31	0.35	0.28	0.36	0.29	0.29	0.29	0.27	0.307	0.093
		fron	0.21	0.10	0.19	0.23	0.20	0.16	0.15	0.25	0.188	0.105	0.21	0.15	0.18	0.21	0.21	0.13	0.18	0.24	0.190	0.087
		tran	0.21	0.18	0.08	0.05	0.09	0.11	0.06	0.13	0.114	0.102	0.19	0.13	0.13	0.10	0.16	0.10	0.11	0.16	0.136	0.095
	Shank	sag	0.23	0.28	0.29	0.38	0.36	0.34	0.39	0.31	0.320	0.113	0.33	0.37	0.29	0.30	0.25	0.30	0.30	0.32	0.306	0.091
		fron	0.06	0.05	0.03	0.14	0.12	0.09	0.07	0.16	0.091	0.107	0.11	0.14	0.10	0.13	0.13	0.12	0.11	0.15	0.124	0.095
		tran	0.12	0.08	0.05	0.04	0.05	0.04	0.03	0.05	0.057	0.072	0.16	0.17	0.16	0.12	0.16	0.09	0.14	0.14	0.143	0.097
	Foot	sag	0.22	0.19	0.21	0.28	0.25	0.22	0.32	0.21	0.237	0.098	0.24	0.19	0.20	0.23	0.18	0.18	0.21	0.26	0.213	0.112
		tran	0.20	0.15	0.16	0.20	0.22	0.14	0.20	0.16	0.179	0.149	0.21	0.12	0.09	0.13	0.13	0.07	0.07	0.08	0.113	0.127

## Appendix E. Gender Differences in Biomechanical Parameters for Various Footwear Conditions

This section contains tables of the effect size Cliff’s Delta regarding gender differences in biomechanical parameters during the right stance phase of running at individual pace and 10 km/h using eight footwear conditions (BF: Barefoot; S1: Vivobarefoot Primus Lite III; S2: Joe Nimble Addict; S3: Nike Free Run 5.0; S4: Puma Liberate 2; S5: Puma Velocity 2; S6: Puma Deviate Elite 2; PS: Personal shoe). Cliff’s Delta is given as a measure of the direction and extent of the gender difference for each footwear condition and regarding all conditions as a mean value (MV) while the standard deviation of Cliff’s Delta indicates the variability of the gender difference regarding the footwear condition.

**Table A10:** Effect size Cliff’s Delta (CD) for gender differences in joint angle parameters; sag = sagittal plane; fron = frontal plane; tran = transverse plane; FS = at foot strike; MS = at mid-stance; TO = at toe-off; MAX = maximum; MIN = minimum; ROM = range of motion; MV = mean of CD across footwear conditions; SD = standard deviation between CD of all footwear conditions.

Joint	Plane	Par.	Individual Pace										10 km/h									
			BF	S1	S2	S3	S4	S5	S6	PS	MV	SD	BF	S1	S2	S3	S4	S5	S6	PS	MV	SD
Hip	sag	FS	**0.42**	**0.27**	**0.25**	**0.26**	**0.20**	**0.21**	**0.24**	**0.20**	0.26	0.07	**0.48**	**0.37**	**0.29**	**0.40**	**0.31**	**0.37**	**0.35**	**0.30**	0.36	0.06
		MS	**0.36**	**0.41**	**0.20**	**0.25**	0.16	**0.21**	**0.16**	**0.19**	0.24	0.09	**0.26**	**0.31**	**0.23**	**0.39**	**0.22**	**0.30**	**0.24**	**0.24**	0.27	0.06
+ flexion		TO	**0.28**	**0.18**	**0.18**	**0.24**	**0.16**	0.12	**0.25**	0.12	0.19	0.06	**0.18**	**0.16**	**0.20**	0.11	0.09	0.10	**0.17**	0.03	0.13	0.06
− extension		MAX	**0.28**	**0.22**	**0.24**	**0.29**	0.14	**0.21**	**0.21**	**0.21**	0.22	0.05	**0.37**	**0.32**	**0.28**	**0.41**	**0.32**	**0.35**	**0.36**	**0.28**	0.34	0.05
		MIN	**0.28**	**0.18**	**0.19**	**0.23**	0.14	0.13	**0.24**	0.13	0.19	0.05	**0.18**	**0.17**	**0.19**	0.11	0.08	0.09	**0.19**	0.03	0.13	0.06
		ROM	0.10	0.08	0.12	0.13	0.06	0.15	0.06	0.12	0.10	0.04	**0.31**	**0.28**	**0.22**	**0.42**	**0.41**	**0.43**	**0.38**	**0.37**	0.35	0.08
	fron	FS	**0.43**	**0.45**	**0.42**	**0.42**	**0.45**	**0.46**	**0.50**	**0.44**	0.45	0.03	**0.45**	**0.55**	**0.41**	**0.41**	**0.43**	**0.47**	**0.52**	**0.54**	0.47	0.06
		MS	**0.36**	**0.39**	**0.38**	**0.47**	**0.47**	**0.49**	**0.54**	**0.44**	0.44	0.06	**0.45**	**0.54**	**0.51**	**0.51**	**0.55**	**0.44**	**0.61**	**0.61**	0.53	0.06
+ adduction		TO	**0.19**	0.09	0.12	**0.25**	**0.22**	0.14	**0.30**	**0.28**	0.20	0.08	**0.17**	0.11	0.08	0.12	0.07	0.02	**0.20**	**0.29**	0.13	0.09
− abduction		MAX	**0.53**	**0.52**	**0.48**	**0.52**	**0.50**	**0.52**	**0.59**	**0.53**	0.52	0.03	**0.55**	**0.62**	**0.59**	**0.56**	**0.60**	**0.57**	**0.67**	**0.64**	0.60	0.04
		MIN	**0.26**	**0.16**	**0.21**	**0.32**	**0.26**	**0.23**	**0.36**	**0.37**	0.27	0.07	**0.29**	**0.19**	**0.16**	**0.19**	0.13	0.13	**0.30**	**0.34**	0.22	0.08
		ROM	**0.37**	**0.44**	**0.35**	**0.33**	**0.38**	**0.38**	**0.42**	**0.35**	0.38	0.04	**0.48**	**0.60**	**0.52**	**0.49**	**0.55**	**0.52**	**0.59**	**0.49**	0.53	0.05
	tran	FS	0.08	0.09	**0.19**	**0.32**	0.12	0.12	0.13	**0.25**	0.16	0.08	0.11	0.15	**0.18**	**0.23**	**0.18**	0.14	**0.20**	**0.31**	0.19	0.06
		MS	**0.46**	**0.37**	**0.40**	**0.45**	**0.33**	**0.31**	**0.31**	**0.41**	0.38	0.06	**0.36**	**0.27**	**0.31**	**0.37**	**0.29**	**0.25**	**0.29**	**0.33**	0.31	0.04
+ internal rot.		TO	**0.28**	**0.21**	**0.30**	**0.35**	**0.33**	**0.30**	**0.21**	**0.42**	0.30	0.07	**0.28**	**0.25**	**0.32**	**0.38**	**0.36**	**0.33**	**0.29**	**0.47**	0.33	0.07
− external rot.		MAX	**0.34**	**0.27**	**0.38**	**0.49**	**0.43**	**0.35**	**0.30**	**0.50**	0.38	0.08	**0.34**	**0.31**	**0.41**	**0.45**	**0.45**	**0.37**	**0.37**	**0.51**	0.40	0.07
		MIN	**0.16**	0.11	**0.28**	**0.32**	**0.21**	**0.19**	**0.17**	**0.35**	0.23	0.08	0.13	**0.16**	**0.19**	**0.30**	**0.20**	**0.19**	**0.23**	**0.32**	0.21	0.07
		ROM	**0.23**	**0.23**	0.10	**0.20**	**0.37**	**0.19**	**0.24**	**0.16**	0.21	0.08	**0.30**	**0.24**	**0.30**	**0.18**	**0.47**	**0.28**	**0.33**	**0.29**	0.30	0.08
Knee	sag	FS	−0.03	−0.06	**0.20**	−0.02	−0.09	0.03	−0.08	0.07	0.07	0.10	−0.13	−0.04	**0.22**	0.02	−0.06	−0.03	0.07	0.08	0.08	0.11
		MS	−0.01	**0.16**	**0.18**	0.06	−0.05	0.05	−0.03	0.05	0.07	0.08	−0.12	−0.13	0.10	−0.06	−0.08	−0.08	−0.11	−0.03	0.09	0.08
+ extension		TO	**0.25**	**0.31**	**0.27**	**0.32**	**0.22**	**0.32**	0.12	**0.37**	0.27	0.08	**0.23**	**0.36**	**0.29**	**0.30**	**0.30**	**0.30**	**0.19**	**0.24**	0.28	0.05
− flexion		MAX	**0.19**	**0.29**	**0.31**	**0.30**	**0.16**	**0.30**	0.14	**0.31**	0.25	0.07	**0.16**	**0.21**	**0.33**	**0.20**	**0.19**	**0.23**	**0.20**	**0.18**	0.21	0.05
		MIN	−0.09	−0.06	0.06	−0.01	−0.10	−0.03	−0.12	0.02	0.06	0.06	**−0.22**	**−0.22**	0.02	−0.13	−0.14	−0.12	**−0.20**	−0.06	0.14	0.08
		ROM	**0.24**	**0.30**	**0.23**	**0.31**	**0.24**	**0.33**	**0.29**	**0.31**	0.28	0.04	**0.34**	**0.42**	**0.33**	**0.39**	**0.27**	**0.41**	**0.43**	**0.27**	0.36	0.06
	fron	FS	**−0.26**	**−0.36**	**−0.33**	**−0.24**	**−0.50**	**−0.32**	**−0.44**	**−0.40**	0.36	0.09	**−0.30**	**−0.32**	**−0.33**	**−0.29**	**−0.39**	**−0.38**	**−0.36**	**−0.34**	0.34	0.03
		MS	**0.25**	0.15	**0.20**	**0.25**	0.07	0.13	**0.17**	0.14	0.17	0.06	0.13	0.10	0.07	**0.18**	0.09	0.07	0.11	0.10	0.11	0.03
+ adduction		TO	−0.10	−0.13	−0.06	−0.02	−0.06	−0.04	**−0.16**	−0.03	0.07	0.05	−0.09	−0.14	−0.02	−0.03	0.01	0.01	−0.15	0.00	0.06	0.07
− abduction		MAX	0.10	0.04	0.12	**0.23**	0.09	0.10	0.07	**0.18**	0.12	0.06	0.04	0.07	0.11	**0.18**	0.10	0.09	0.08	**0.17**	0.11	0.05
		MIN	−0.08	−0.12	0.07	0.14	−0.10	0.02	−0.03	0.02	0.07	0.09	**−0.16**	−0.10	−0.10	0.06	−0.11	−0.07	−0.11	−0.03	0.09	0.07
		ROM	**0.16**	**0.18**	0.09	**0.17**	**0.36**	0.11	0.14	**0.22**	0.18	0.08	**0.24**	**0.18**	**0.25**	**0.17**	**0.43**	**0.26**	**0.31**	**0.36**	0.27	0.09
	tran	MS	−0.07	**−0.26**	−0.06	−0.07	−0.14	−0.03	−0.04	−0.06	0.09	0.08	0.09	0.08	**0.20**	0.06	0.04	0.14	**0.29**	0.10	0.12	0.08
		TO	**−0.27**	**−0.36**	**−0.36**	**−0.32**	**−0.50**	**−0.48**	**−0.44**	**−0.41**	0.39	0.08	**−0.27**	**−0.33**	**−0.29**	**−0.42**	**−0.49**	**−0.49**	**−0.37**	**−0.44**	0.39	0.09
+ internal rot.		MAX	0.11	0.06	0.01	−0.13	**−0.22**	**−0.17**	−0.16	**−0.19**	0.13	0.13	**0.20**	**0.19**	**0.22**	0.02	0.07	0.03	**0.21**	0.03	0.12	0.09
− external rot.		MIN	−0.13	**−0.22**	**−0.24**	**−0.29**	**−0.45**	**−0.39**	**−0.41**	**−0.33**	0.31	0.11	−0.13	**−0.21**	−0.13	**−0.34**	**−0.37**	**−0.38**	**−0.30**	**−0.33**	0.27	0.10
		ROM	**0.23**	**0.22**	0.13	0.01	0.02	0.13	0.10	0.05	0.11	0.08	**0.33**	**0.39**	**0.34**	**0.28**	**0.33**	**0.35**	**0.40**	**0.25**	0.33	0.05
Ankle	sag	FS	**−0.40**	**−0.29**	**−0.21**	−0.04	−0.06	−0.08	−0.12	0.01	0.15	0.14	**−0.46**	**−0.46**	**−0.26**	0.01	−0.12	−0.07	**−0.19**	−0.01	0.20	0.19
		MS	−0.05	**−0.28**	−0.09	0.13	**0.19**	0.10	0.09	0.00	0.12	0.15	0.01	−0.08	−0.07	**0.16**	0.08	0.10	0.04	0.05	0.07	0.08
+ dorsiflexion		TO	**−0.18**	−0.10	−0.09	0.11	−0.02	−0.12	0.02	−0.04	0.08	0.09	−0.13	−0.12	−0.03	0.00	−0.02	−0.10	−0.01	−0.09	0.06	0.05
− plantarflexion		MAX	0.01	−0.05	−0.10	**0.17**	**0.18**	0.09	0.07	0.00	0.08	0.10	−0.03	−0.07	−0.10	**0.20**	0.08	0.12	0.00	0.03	0.08	0.10
		MIN	**−0.18**	−0.07	−0.09	0.11	−0.02	−0.12	0.02	−0.04	0.08	0.09	−0.13	−0.11	−0.03	0.01	−0.02	−0.10	−0.01	−0.09	0.06	0.05
		ROM	**0.16**	0.11	0.04	0.08	0.14	**0.22**	0.08	0.03	0.11	0.06	0.08	0.09	0.00	0.14	0.12	**0.20**	0.06	0.06	0.10	0.06
	fron	FS	0.10	0.11	**0.18**	−0.12	0.06	0.11	0.12	0.01	0.10	0.09	0.09	0.10	**0.16**	−0.06	0.08	0.06	0.12	−0.04	0.09	0.08
		MS	−0.13	−0.05	−0.11	**−0.33**	**−0.23**	**−0.18**	−0.15	**−0.17**	0.17	0.08	−0.04	−0.06	−0.08	**−0.27**	**−0.22**	−0.14	−0.12	−0.15	0.14	0.08
+ supination		TO	−0.04	0.04	−0.04	**−0.28**	**−0.16**	−0.09	−0.02	**−0.23**	0.11	0.11	−0.02	0.03	−0.05	**−0.19**	−0.09	−0.07	−0.01	**−0.20**	0.08	0.08
− pronation		MAX	0.00	0.01	0.10	**−0.17**	−0.05	0.02	0.07	−0.09	0.06	0.09	0.02	0.02	0.08	−0.11	0.02	0.04	0.06	−0.10	0.06	0.07
		MIN	**−0.20**	−0.12	−0.12	**−0.31**	**−0.25**	−0.15	−0.13	**−0.21**	0.19	0.07	−0.14	**−0.18**	−0.14	**−0.29**	**−0.20**	−0.13	−0.10	**−0.19**	0.17	0.06
		ROM	0.15	0.09	**0.22**	0.08	**0.17**	0.11	**0.16**	−0.02	0.12	0.07	0.15	0.13	**0.21**	0.12	**0.19**	0.10	**0.19**	−0.01	0.14	0.07

CD in bold—significant difference (*p* < 0.05); red cell—female > male (CD > 0); blue cell—female < male (CD < 0); light colour—small effect (|CD| > 0.11); medium colour—medium effect (|CD| > 0.28); dark colour—large effect (|CD| > 0.43); pink cell—mean CD (the higher the value, the darker the colour); green cell—standard deviation (the higher the value, the darker the colour).

**Table A11:** Effect size Cliff’s Delta (CD) for gender differences in peak joint angular velocities; sag = sagittal plane; fron = frontal plane; tran = transverse plane; FLEX = flexing; EXT = extending; ADD = adducting; ABD = abducting; IR = internally rotating; ER = externally rotating; DF = dorsiflexing; PF = plantarflexing; SUP = supinating; PRO = pronating; MV = mean of CD across footwear conditions; SD = standard deviation between CD of all footwear conditions.

Joint	Plane	Dir.	Individual Pace										10 km/h									
			BF	S1	S2	S3	S4	S5	S6	PS	MV	SD	BF	S1	S2	S3	S4	S5	S6	PS	MV	SD
Hip	sag	FLEX	**−0.15**	−0.08	**0.24**	**0.24**	0.07	**0.17**	**0.20**	**0.20**	0.17	0.15	**−0.26**	−0.04	**0.21**	0.14	**0.17**	0.08	**0.19**	0.10	0.15	0.16
		EXT	−0.13	−0.10	**−0.17**	−0.14	**−0.17**	−0.14	**−0.22**	−0.11	0.15	0.04	**0.22**	**0.39**	**0.17**	**0.41**	**0.29**	**0.37**	**0.34**	**0.38**	0.32	0.09
	fron	ADD	**0.27**	**0.34**	**0.27**	**0.33**	**0.36**	**0.35**	**0.41**	**0.46**	0.35	0.06	**0.24**	**0.51**	**0.44**	**0.50**	**0.52**	**0.43**	**0.52**	**0.50**	0.46	0.10
		ABD	**0.25**	**0.30**	**0.27**	**0.24**	**0.35**	**0.35**	**0.25**	0.15	0.27	0.07	**0.45**	**0.57**	**0.48**	**0.48**	**0.46**	**0.48**	**0.44**	**0.44**	0.48	0.04
	tran	IR	**0.42**	**0.41**	**0.17**	**0.30**	**0.49**	**0.30**	**0.26**	**0.27**	0.33	0.10	**0.40**	**0.34**	**0.46**	**0.36**	**0.45**	**0.41**	**0.40**	**0.43**	0.41	0.04
		ER	**0.32**	**0.30**	**0.19**	0.13	**0.30**	**0.27**	**0.22**	**0.22**	0.24	0.06	**0.35**	**0.25**	**0.45**	**0.18**	**0.38**	**0.36**	**0.37**	**0.33**	0.33	0.08
Knee	sag	EXT	**0.33**	**0.33**	0.10	**0.24**	**0.20**	**0.25**	**0.17**	**0.27**	0.24	0.08	**0.44**	**0.59**	**0.38**	**0.58**	**0.45**	**0.56**	**0.48**	**0.46**	0.49	0.08
		FLEX	**−0.30**	**−0.33**	−0.08	−0.12	−0.14	−0.06	−0.10	−0.03	0.15	0.11	**−0.20**	0.10	**0.26**	**0.24**	**0.25**	**0.23**	**0.43**	**0.25**	0.25	0.18
	fron	ADD	**0.38**	**0.37**	**0.19**	**0.35**	**0.48**	**0.21**	**0.27**	**0.31**	0.32	0.10	**0.36**	**0.38**	**0.44**	**0.38**	**0.45**	**0.39**	**0.40**	**0.49**	0.41	0.05
		ABD	**0.17**	**0.17**	0.14	0.11	**0.24**	**0.16**	0.03	0.13	0.14	0.06	**0.20**	0.12	**0.31**	**0.19**	**0.32**	**0.29**	**0.22**	**0.27**	0.24	0.07
	tran	IR	0.02	−0.05	−0.05	**−0.18**	**−0.25**	**−0.26**	**−0.21**	**−0.22**	0.16	0.11	**0.24**	**0.16**	**0.20**	0.00	**0.20**	0.04	0.12	0.08	0.13	0.09
		ER	**0.18**	**0.17**	0.05	0.07	0.08	0.11	**0.19**	0.10	0.12	0.05	**0.40**	**0.47**	**0.39**	**0.46**	**0.39**	**0.46**	**0.53**	**0.39**	0.44	0.05
Ankle	sag	DF	**0.40**	**0.18**	0.08	−0.01	0.03	0.13	0.03	**−0.17**	0.13	0.16	**0.48**	**0.36**	**0.31**	0.15	**0.25**	**0.28**	**0.22**	0.07	0.27	0.13
		PF	0.10	−0.07	−0.12	−0.16	−0.04	0.00	−0.10	−0.15	0.09	0.09	**0.35**	**0.27**	**0.19**	**0.30**	**0.24**	**0.32**	**0.29**	**0.24**	0.28	0.05
	fron	SUP	**0.17**	**0.18**	0.05	−0.04	0.09	0.08	**0.16**	0.00	0.10	0.08	**0.18**	0.13	0.04	0.05	0.09	0.14	0.15	−0.07	0.11	0.08
		PRO	0.14	**0.16**	0.14	0.07	0.13	0.07	0.11	0.00	0.10	0.05	**0.25**	**0.28**	**0.21**	**0.19**	**0.30**	**0.17**	**0.20**	0.02	0.20	0.09

CD in bold—significant difference (*p* < 0.05); red cell—female > male (CD > 0); blue cell—female < male (CD < 0); light colour—small effect (|CD| > 0.11); medium colour—medium effect (|CD| > 0.28); dark colour—large effect (|CD| > 0.43); pink cell—mean CD (the higher the value, the darker the colour); green cell—standard deviation (the higher the value, the darker the colour).

**Table A12:** Effect size Cliff’s Delta (CD) for gender differences in peak external joint moments; sag = sagittal plane; fron = frontal plane; tran = transverse plane; FLEX = tending to flex; EXT = tending to extend; ADD = tending to adduct; ABD = tending to abduct; IR = tending to internally rotate; ER = tending to externally rotate; DF = tending to dorsiflex; PF = tending to plantarflex; SUP = tending to supinate; PRO = tending to pronate; MV = mean of CD across footwear conditions; SD = standard deviation between CD of all footwear conditions.

Joint	Plane	Dir.	Individual Pace										10 km/h									
			BF	S1	S2	S3	S4	S5	S6	PS	MV	SD	BF	S1	S2	S3	S4	S5	S6	PS	MV	SD
Hip	sag	FLEX	−0.14	−0.04	**−0.19**	**−0.39**	**−0.25**	**−0.23**	**−0.32**	**−0.40**	0.24	0.13	−0.11	0.13	−0.12	**−0.19**	−0.07	−0.11	−0.09	**−0.26**	0.14	0.11
		EXT	0.01	**0.20**	**−0.18**	**−0.16**	−0.04	0.01	−0.14	−0.09	0.10	0.12	0.09	0.13	0.12	−0.04	−0.01	0.12	0.09	0.08	0.08	0.06
	fron	ADD	−0.05	−0.05	**−0.20**	**−0.30**	**−0.43**	**−0.25**	**−0.17**	**−0.27**	0.21	0.13	0.00	0.09	−0.11	**−0.19**	−0.12	−0.11	**−0.27**	−0.05	0.12	0.11
		ABD	0.06	0.03	**−0.16**	**−0.19**	−0.06	−0.03	**−0.16**	−0.12	0.10	0.09	**0.15**	**0.21**	0.01	−0.13	0.06	−0.01	0.09	0.00	0.08	0.10
	tran	IR	0.10	0.05	0.05	−0.06	**−0.21**	−0.04	−0.02	−0.05	0.07	0.09	**0.21**	**0.25**	**0.19**	**0.17**	0.12	**0.16**	0.05	**0.21**	0.17	0.06
		ER	−0.13	0.04	**−0.22**	**−0.29**	**−0.22**	−0.05	−0.13	**−0.20**	0.16	0.11	−0.08	0.09	−0.10	**−0.20**	−0.08	−0.12	0.09	**−0.18**	0.12	0.11
Knee	sag	EXT	0.15	**0.26**	**0.26**	**0.28**	0.14	**0.36**	0.04	**0.19**	0.21	0.10	**0.26**	**0.24**	**0.33**	**0.43**	**0.31**	**0.39**	**0.21**	**0.27**	0.30	0.08
		FLEX	**−0.42**	**−0.30**	**−0.55**	**−0.51**	**−0.41**	**−0.37**	**−0.44**	**−0.53**	0.44	0.09	**−0.28**	**−0.24**	**−0.31**	**−0.33**	**−0.22**	**−0.23**	**−0.22**	**−0.25**	0.26	0.04
	fron	ADD	−0.11	**−0.21**	−0.15	−0.07	**−0.31**	−0.09	**−0.17**	−0.16	0.16	0.08	−0.11	−0.10	−0.02	−0.01	−0.08	−0.04	**−0.16**	0.05	0.07	0.07
		ABD	−0.01	0.05	−0.14	**−0.21**	0.00	−0.01	−0.11	**−0.16**	0.09	0.09	0.14	0.13	0.04	**−0.19**	0.08	−0.03	**0.16**	−0.05	0.10	0.12
	tran	IR	0.09	0.08	0.12	0.04	**0.22**	0.11	**0.19**	0.11	0.12	0.06	**0.22**	0.14	**0.32**	0.10	**0.24**	**0.16**	**0.43**	**0.19**	0.23	0.11
		ER	−0.12	−0.04	**−0.21**	**−0.30**	**−0.47**	**−0.31**	−0.12	**−0.29**	0.23	0.14	−0.13	−0.04	**−0.20**	−0.15	−0.13	−0.10	**−0.25**	−0.04	0.13	0.07
Ankle	sag	DF	0.02	0.07	−0.03	0.11	0.11	0.13	0.03	−0.04	0.07	0.07	**0.24**	**0.22**	0.15	**0.24**	**0.17**	**0.25**	**0.26**	0.11	0.20	0.06
		PF	**−0.26**	−0.11	−0.13	**−0.21**	**−0.23**	**−0.19**	**−0.27**	−0.05	0.18	0.08	**−0.26**	−0.10	−0.05	−0.06	−0.15	−0.11	**−0.23**	0.08	0.13	0.11
	fron	SUP	**−0.29**	−0.13	−0.15	**−0.25**	**−0.30**	**−0.17**	**−0.22**	**−0.19**	0.21	0.06	**−0.24**	−0.11	−0.14	−0.10	**−0.17**	−0.09	**−0.21**	−0.04	0.14	0.07
		PRO	0.00	0.04	−0.06	**0.22**	**0.25**	**0.23**	−0.03	0.12	0.12	0.13	0.12	**0.18**	**0.18**	**0.23**	**0.20**	**0.19**	**0.30**	0.06	0.18	0.07

CD in bold—significant difference (*p* < 0.05); red cell—female > male (CD > 0); blue cell—female < male (CD < 0); light colour—small effect (|CD| > 0.11); medium colour—medium effect (|CD| > 0.28); dark colour—large effect (|CD| > 0.43); pink cell—mean CD (the higher the value, the darker the colour); green cell—standard deviation (the higher the value, the darker the colour).

**Table A13:** Effect size Cliff’s Delta (CD) for gender differences in external joint angular impulses; sag = sagittal plane; fron = frontal plane; tran = transverse plane; FLEX = tending to flex; EXT = tending to extend; ADD = tending to adduct; ABD = tending to abduct; IR = tending to internally rotate; ER = tending to externally rotate; DF = tending to dorsiflex; PF = tending to plantarflex; SUP = tending to supinate; PRO = tending to pronate; MV = mean of CD across footwear conditions; SD = standard deviation between CD of all footwear conditions.

Joint	Plane	Dir.	Individual Pace										10 km/h									
			BF	S1	S2	S3	S4	S5	S6	PS	MV	SD	BF	S1	S2	S3	S4	S5	S6	PS	MV	SD
Hip	sag	FLEX	0.14	0.12	0.05	0.04	0.03	0.16	−0.07	0.02	0.08	0.07	0.05	**0.17**	0.15	0.12	0.07	0.15	0.06	0.00	0.10	0.06
		EXT	0.04	0.04	−0.08	−0.09	−0.05	−0.05	−0.05	−0.10	0.06	0.05	0.12	−0.05	0.02	−0.02	−0.04	0.00	−0.02	0.01	0.03	0.05
	fron	ADD	−0.02	0.07	0.01	−0.11	**−0.27**	−0.14	0.04	−0.11	0.10	0.11	−0.04	−0.02	−0.12	−0.09	−0.08	−0.06	**−0.22**	0.02	0.08	0.07
		ABD	−0.07	−0.11	**−0.23**	−0.13	**−0.16**	−0.10	−0.08	**−0.17**	0.13	0.05	0.04	0.01	−0.13	−0.13	−0.06	−0.10	0.04	−0.05	0.07	0.07
	tran	IR	**0.25**	**0.25**	**0.23**	0.14	0.00	0.12	**0.21**	0.15	0.17	0.09	**0.29**	**0.27**	**0.21**	**0.27**	**0.23**	**0.28**	0.08	**0.33**	0.24	0.07
		ER	**−0.23**	−0.05	**−0.28**	**−0.26**	**−0.28**	−0.14	**−0.20**	**−0.27**	0.21	0.08	**−0.25**	**−0.17**	**−0.31**	**−0.26**	**−0.32**	**−0.22**	−0.14	**−0.29**	0.25	0.07
Knee	sag	EXT	0.12	**0.18**	**0.26**	**0.30**	**0.16**	**0.32**	0.07	**0.26**	0.21	0.09	**0.21**	**0.20**	**0.30**	**0.33**	**0.26**	**0.33**	**0.17**	**0.27**	0.26	0.06
		FLEX	**−0.28**	**−0.27**	**−0.39**	**−0.43**	**−0.28**	**−0.25**	**−0.29**	**−0.41**	0.32	0.07	**−0.28**	**−0.28**	**−0.35**	**−0.32**	**−0.27**	**−0.25**	**−0.22**	**−0.32**	0.29	0.04
	fron	ADD	−0.01	−0.01	0.06	0.00	**−0.25**	−0.07	0.02	−0.02	0.06	0.09	−0.08	−0.02	−0.06	−0.01	−0.11	−0.06	**−0.17**	0.04	0.07	0.06
		ABD	−0.09	−0.11	**−0.19**	−0.12	−0.02	0.01	−0.11	−0.15	0.10	0.06	0.06	−0.06	−0.04	**−0.23**	0.01	−0.07	0.04	−0.08	0.07	0.09
	tran	IR	**0.22**	**0.17**	**0.21**	**0.16**	**0.36**	**0.25**	**0.25**	**0.24**	0.23	0.06	**0.33**	**0.24**	**0.37**	**0.22**	**0.37**	**0.33**	**0.47**	**0.32**	0.33	0.08
		ER	−0.04	0.07	−0.05	**−0.17**	**−0.35**	**−0.17**	0.03	**−0.19**	0.13	0.14	−0.06	−0.02	**−0.18**	−0.10	−0.09	−0.06	**−0.23**	−0.02	0.09	0.07
Ankle	sag	DF	**0.24**	**0.17**	0.12	**0.23**	**0.20**	**0.27**	**0.21**	0.07	0.19	0.07	**0.23**	0.14	0.04	0.13	0.08	**0.20**	0.15	0.02	0.12	0.07
		PF	**−0.29**	**−0.16**	−0.15	**−0.21**	**−0.24**	**−0.20**	**−0.24**	−0.08	0.20	0.07	**−0.35**	**−0.22**	−0.08	−0.10	**−0.20**	**−0.17**	**−0.24**	0.04	0.17	0.12
	fron	SUP	**−0.30**	−0.15	−0.11	**−0.21**	**−0.26**	**−0.18**	**−0.16**	**−0.22**	0.20	0.06	**−0.29**	**−0.18**	−0.13	−0.08	**−0.20**	−0.12	**−0.21**	−0.12	0.17	0.07
		PRO	**0.17**	0.11	0.06	**0.26**	**0.28**	**0.27**	0.08	**0.20**	0.18	0.09	0.14	0.15	0.11	0.15	0.10	0.11	**0.22**	0.05	0.13	0.05

CD in bold—significant difference (*p* < 0.05); red cell—female > male (CD > 0); blue cell—female < male (CD < 0); light colour—small effect (|CD| > 0.11); medium colour—medium effect (|CD| > 0.28); dark colour—large effect (|CD| > 0.43); pink cell—mean CD (the higher the value, the darker the colour); green cell—standard deviation (the higher the value, the darker the colour).

**Table A14:** Effect size Cliff’s Delta (CD) for gender differences in peak joint powers; sag = sagittal plane; fron = frontal plane; tran = transverse plane; FLEX = flexor; EXT = extensor; ADD = adductor; ABD = abductor; IR = internal rotator; ER = external rotator; DF = dorsiflexor; PF = plantar flexor; SUP = supinator; PRO = pronator; con = concentric; ecc = eccentric; MV = mean of CD across footwear conditions; SD = standard deviation between CD of all footwear conditions.

Joint	Pl.	Dir.	Mov.	Individual Pace										10 km/h									
				BF	S1	S2	S3	S4	S5	S6	PS	MV	SD	BF	S1	S2	S3	S4	S5	S6	PS	MV	SD
Hip	sag	FLEX	con	−0.08	−0.07	**0.21**	0.14	0.03	**0.16**	**0.15**	0.05	0.11	0.11	−0.13	−0.03	**0.16**	0.07	0.06	0.11	**0.18**	0.04	0.10	0.10
			ecc	−0.11	−0.05	**−0.23**	**−0.28**	**−0.23**	**−0.19**	**−0.23**	**−0.23**	0.19	0.08	**0.16**	0.10	0.05	−0.01	−0.02	0.09	−0.02	0.04	0.06	0.06
		EXT	con	−0.02	**0.20**	−0.11	−0.11	−0.06	−0.05	−0.16	−0.09	0.10	0.11	**0.29**	**0.27**	0.11	0.12	0.11	**0.16**	0.04	0.02	0.14	0.10
			ecc	**−0.19**	**−0.19**	0.08	0.02	−0.14	−0.01	0.05	−0.01	0.09	0.11	**−0.33**	−0.12	0.03	−0.06	−0.08	−0.08	0.02	−0.05	0.10	0.11
	fron	ADD	con	0.03	0.11	−0.11	**−0.17**	−0.07	0.05	−0.04	−0.07	0.08	0.09	**0.17**	**0.24**	0.05	−0.03	**0.16**	0.05	**0.20**	0.05	0.12	0.10
			ecc	0.02	−0.05	−0.05	−0.06	0.01	0.02	**−0.15**	−0.05	0.05	0.06	−0.05	0.04	−0.01	−0.03	−0.08	−0.06	−0.04	0.06	0.05	0.05
		ABD	con	**0.17**	**0.24**	0.15	−0.02	0.04	0.06	0.12	0.02	0.10	0.09	**0.30**	**0.35**	**0.27**	**0.23**	**0.27**	**0.26**	**0.16**	**0.33**	0.27	0.06
			ecc	0.02	0.11	0.02	−0.04	0.01	0.07	0.13	0.10	0.06	0.06	0.03	**0.29**	0.15	**0.18**	**0.19**	**0.17**	**0.21**	**0.25**	0.18	0.08
	tran	IR	con	0.01	**0.18**	−0.01	**−0.26**	−0.07	0.07	−0.11	−0.11	0.10	0.13	0.12	**0.23**	0.10	−0.05	0.06	−0.06	**0.15**	0.00	0.10	0.10
			ecc	**−0.26**	−0.14	**−0.39**	−0.09	**−0.19**	−0.10	**−0.18**	**−0.23**	0.20	0.10	**−0.27**	**−0.20**	**−0.38**	**−0.25**	**−0.23**	**−0.20**	**−0.17**	**−0.29**	0.25	0.07
		ER	con	**0.29**	**0.35**	**0.21**	0.13	0.12	0.09	**0.22**	**0.16**	0.20	0.09	**0.44**	**0.26**	**0.36**	**0.22**	**0.40**	**0.36**	**0.22**	**0.47**	0.34	0.10
			ecc	**0.34**	**0.32**	**0.29**	**0.18**	**0.22**	**0.27**	**0.25**	**0.21**	0.26	0.06	**0.33**	**0.38**	**0.46**	**0.33**	**0.34**	**0.39**	**0.26**	**0.42**	0.36	0.06
Knee	sag	EXT	con	**−0.32**	**−0.20**	**−0.41**	**−0.37**	**−0.33**	**−0.16**	**−0.25**	**−0.40**	0.31	0.09	**−0.21**	−0.07	**−0.28**	−0.15	**−0.22**	−0.04	−0.09	**−0.26**	0.17	0.09
			ecc	**−0.53**	**−0.40**	**−0.47**	**−0.51**	**−0.36**	**−0.30**	**−0.36**	**−0.41**	0.42	0.08	**−0.26**	−0.04	**−0.16**	−0.12	0.02	−0.15	0.04	−0.11	0.11	0.10
		FLEX	con	0.15	**0.26**	**0.35**	**0.25**	**0.24**	**0.21**	**0.26**	**0.23**	0.24	0.06	**0.18**	**0.21**	**0.35**	**0.38**	**0.37**	**0.27**	**0.24**	**0.20**	0.28	0.08
			ecc	0.09	**0.16**	**0.19**	**0.25**	**0.24**	**0.33**	0.11	**0.26**	0.20	0.08	**0.23**	**0.30**	**0.33**	**0.39**	**0.36**	**0.44**	**0.30**	**0.33**	0.34	0.06
	fron	ADD	con	**0.29**	**0.32**	0.06	0.00	**0.32**	0.14	**0.17**	−0.01	0.16	0.14	**0.32**	**0.31**	**0.33**	0.04	**0.32**	**0.15**	**0.40**	**0.25**	0.27	0.12
			ecc	**−0.15**	−0.11	**−0.20**	**−0.20**	**−0.18**	−0.09	**−0.23**	**−0.17**	0.17	0.05	−0.06	−0.07	−0.11	**−0.23**	−0.12	−0.08	−0.02	**−0.20**	0.11	0.07
		ABD	con	0.04	0.03	−0.01	−0.10	**−0.17**	−0.07	−0.07	−0.03	0.07	0.07	0.07	0.06	0.11	−0.03	**0.16**	0.10	−0.11	0.15	0.10	0.09
			ecc	**0.19**	0.09	0.12	0.06	0.03	0.03	0.07	0.14	0.09	0.06	0.13	**0.18**	**0.16**	**0.16**	0.08	0.15	0.05	**0.24**	0.15	0.06
	tran	IR	con	**−0.32**	**−0.17**	**−0.32**	**−0.41**	**−0.53**	**−0.34**	**−0.20**	**−0.39**	0.33	0.12	**−0.17**	−0.02	−0.05	−0.07	−0.06	−0.02	−0.07	−0.02	0.06	0.05
			ecc	**0.19**	**0.19**	0.10	**−0.22**	−0.14	−0.07	**0.16**	−0.10	0.15	0.16	**0.22**	**0.26**	**0.23**	0.07	**0.22**	0.16	0.12	**0.27**	0.19	0.07
		ER	con	−0.09	0.06	**0.21**	**0.34**	**0.35**	**0.33**	**0.19**	**0.35**	0.24	0.16	0.04	0.13	**0.26**	**0.28**	**0.36**	**0.25**	**0.30**	**0.29**	0.24	0.10
			ecc	0.11	−0.04	−0.04	**−0.21**	−0.04	−0.05	−0.01	−0.11	0.08	0.09	**0.20**	0.08	**0.17**	−0.13	0.11	0.01	**0.30**	0.02	0.13	0.13
Ankle	sag	DF	con	**−0.21**	−0.12	−0.14	**−0.20**	**−0.24**	−0.15	**−0.30**	−0.14	0.19	0.06	**−0.22**	−0.10	−0.06	−0.09	**−0.18**	−0.10	**−0.22**	0.02	0.12	0.08
			ecc	**−0.33**	**−0.18**	**−0.16**	**−0.22**	**−0.18**	**−0.18**	**−0.19**	−0.10	0.19	0.07	**−0.37**	**−0.18**	−0.06	−0.10	−0.12	−0.10	**−0.15**	−0.02	0.14	0.11
		PF	con	−0.11	−0.12	**−0.25**	−0.09	−0.05	−0.12	**−0.19**	**−0.24**	0.14	0.07	0.14	**0.19**	0.00	**0.16**	0.12	**0.20**	0.13	0.05	0.12	0.07
			ecc	**0.22**	0.14	0.00	0.08	0.12	0.12	0.07	−0.04	0.10	0.08	**0.43**	**0.35**	**0.28**	**0.26**	**0.28**	**0.26**	**0.30**	**0.19**	0.29	0.07
	fron	SUP	con	0.10	0.10	−0.06	−0.09	0.08	0.05	−0.03	−0.03	0.07	0.08	0.15	**0.23**	**0.22**	0.09	**0.20**	0.09	**0.19**	−0.04	0.15	0.09
			ecc	**0.46**	**0.35**	**0.20**	**0.19**	**0.35**	**0.26**	**0.28**	0.12	0.28	0.11	**0.49**	**0.41**	**0.40**	**0.23**	**0.35**	**0.23**	**0.43**	**0.19**	0.34	0.11
		PRO	con	**−0.25**	−0.09	−0.02	−0.14	**−0.22**	−0.05	−0.11	−0.10	0.12	0.08	**−0.22**	−0.06	−0.02	0.03	−0.08	0.00	**−0.16**	−0.01	0.07	0.09
			ecc	−0.07	−0.08	**−0.15**	**−0.22**	**−0.10**	**−0.15**	**−0.10**	**−0.16**	0.13	0.05	−0.07	**−0.11**	−0.04	**−0.14**	−0.06	**−0.11**	−0.08	**−0.13**	0.09	0.04

CD in bold—significant difference (*p* < 0.05); red cell—female > male (CD > 0); blue cell—female < male (CD < 0); light colour—small effect (|CD| > 0.11); medium colour—medium effect (|CD| > 0.28); dark colour—large effect (|CD| > 0.43); pink cell—mean CD (the higher the value, the darker the colour); green cell—standard deviation (the higher the value, the darker the colour).

**Table A15:** Effect size Cliff’s Delta (CD) for gender differences in joint work; sag = sagittal plane; fron = frontal plane; tran = transverse plane; FLEX = flexor; EXT = extensor; ADD = adductor; ABD = abductor; IR = internal rotator; ER = external rotator; DF = dorsiflexor; PF = plantar flexor; SUP = supinator; PRO = pronator; con = concentric; ecc = eccentric; MV = mean of CD across footwear conditions; SD = standard deviation between CD of all footwear conditions.

Joint	Pl.	Dir.	Mov.	Individual Pace										10 km/h									
				BF	S1	S2	S3	S4	S5	S6	PS	MV	SD	BF	S1	S2	S3	S4	S5	S6	PS	MV	SD
Hip	sag	FLEX	con	−0.06	−0.04	**0.20**	**0.17**	0.05	0.15	**0.20**	0.04	0.11	0.11	**−0.14**	−0.05	0.12	0.05	0.05	0.06	0.12	−0.01	0.07	0.09
			ecc	0.01	−0.01	−0.11	−0.13	−0.08	−0.08	−0.06	−0.11	0.07	0.05	**0.18**	0.01	0.06	0.09	0.01	0.08	0.03	0.07	0.07	0.06
		EXT	con	0.15	**0.19**	0.00	0.03	0.01	0.08	−0.07	0.01	0.07	0.08	**0.25**	**0.21**	**0.16**	0.14	0.10	**0.17**	0.04	0.03	0.14	0.08
			ecc	**−0.16**	**−0.19**	0.08	0.06	−0.15	0.02	0.03	0.01	0.09	0.11	**−0.32**	−0.14	−0.01	−0.07	−0.11	−0.10	0.00	−0.09	0.11	0.10
	fron	ADD	con	0.05	0.11	−0.05	−0.04	−0.02	0.03	0.08	0.02	0.05	0.06	**0.28**	**0.26**	0.03	0.09	**0.18**	0.08	**0.31**	0.09	0.16	0.11
			ecc	−0.03	−0.15	**−0.17**	−0.07	−0.08	−0.02	−0.14	−0.12	0.10	0.06	−0.03	0.05	−0.06	−0.04	0.14	−0.07	−0.09	−0.02	0.06	0.08
		ABD	con	**0.24**	**0.30**	**0.20**	0.06	0.06	0.08	**0.19**	0.12	0.15	0.09	**0.29**	**0.36**	**0.21**	**0.23**	**0.24**	**0.26**	**0.16**	**0.33**	0.26	0.07
			ecc	−0.05	0.08	0.07	−0.01	−0.04	0.03	0.14	0.04	0.06	0.06	−0.06	0.11	0.08	0.09	0.14	0.08	0.08	0.15	0.10	0.06
	tran	IR	con	0.10	**0.21**	0.10	−0.08	−0.02	0.13	0.02	−0.02	0.09	0.10	**0.21**	**0.23**	0.04	0.04	0.11	0.05	**0.26**	0.13	0.13	0.09
			ecc	**−0.30**	**−0.27**	**−0.41**	**−0.23**	**−0.33**	**−0.36**	**−0.25**	**−0.38**	0.32	0.06	**−0.39**	**−0.30**	**−0.34**	**−0.34**	**−0.36**	**−0.31**	**−0.25**	**−0.48**	0.35	0.07
		ER	con	**0.38**	**0.39**	**0.24**	**0.19**	**0.17**	0.12	**0.29**	**0.26**	0.26	0.10	**0.47**	**0.33**	**0.37**	**0.27**	**0.39**	**0.34**	**0.28**	**0.50**	0.37	0.08
			ecc	**0.35**	**0.31**	**0.24**	**0.20**	**0.20**	**0.24**	**0.21**	**0.19**	0.24	0.06	**0.29**	**0.31**	**0.39**	**0.29**	**0.30**	**0.37**	**0.18**	**0.41**	0.32	0.07
Knee	sag	EXT	con	**−0.16**	−0.11	**−0.29**	**−0.27**	**−0.17**	−0.13	**−0.16**	**−0.31**	0.20	0.08	−0.15	−0.07	**−0.26**	−0.15	**−0.19**	−0.10	−0.09	**−0.28**	0.16	0.08
			ecc	**−0.39**	**−0.34**	**−0.45**	**−0.53**	**−0.37**	**−0.27**	**−0.39**	**−0.45**	0.40	0.08	**−0.27**	−0.14	**−0.23**	**−0.25**	**−0.16**	**−0.19**	−0.10	**−0.25**	0.20	0.06
		FLEX	con	**0.24**	**0.27**	**0.38**	**0.25**	**0.16**	**0.27**	**0.24**	**0.20**	0.25	0.06	**0.25**	**0.27**	**0.34**	**0.30**	**0.27**	**0.29**	**0.21**	0.14	0.26	0.06
			ecc	0.09	0.13	**0.20**	**0.29**	**0.25**	**0.34**	0.09	**0.32**	0.21	0.10	**0.21**	**0.23**	**0.30**	**0.34**	**0.35**	**0.39**	**0.22**	**0.34**	0.30	0.07
	fron	ADD	con	**0.35**	**0.31**	0.12	**0.22**	**0.44**	**0.25**	**0.29**	**0.17**	0.27	0.10	**0.32**	**0.24**	**0.32**	**0.22**	**0.44**	**0.29**	**0.50**	**0.36**	0.34	0.10
			ecc	**−0.21**	**−0.33**	**−0.22**	**−0.18**	**−0.21**	−0.13	**−0.20**	**−0.25**	0.22	0.06	−0.09	**−0.20**	−0.08	**−0.24**	−0.15	**−0.18**	−0.12	**−0.19**	0.16	0.06
		ABD	con	**0.18**	**0.16**	0.10	0.04	−0.10	−0.04	0.02	0.03	0.08	0.10	0.13	**0.16**	0.06	0.04	0.11	0.06	−0.04	0.09	0.09	0.06
			ecc	0.03	−0.04	0.08	−0.02	−0.03	0.02	0.03	0.11	0.04	0.05	−0.02	0.06	0.07	0.06	0.02	0.12	−0.07	**0.20**	0.08	0.08
	tran	IR	con	**−0.28**	0.05	−0.10	−0.09	**−0.36**	**−0.19**	−0.01	**−0.18**	0.16	0.14	**−0.16**	0.04	−0.05	0.02	−0.05	0.07	−0.06	0.08	0.07	0.08
			ecc	**0.20**	**0.16**	0.07	**−0.22**	**−0.19**	−0.12	0.12	**−0.18**	0.16	0.17	**0.19**	0.15	0.12	−0.03	**0.17**	0.08	0.03	0.11	0.11	0.07
		ER	con	0.06	**0.27**	**0.33**	**0.43**	**0.49**	**0.55**	**0.34**	**0.46**	0.37	0.15	**0.21**	**0.23**	**0.34**	**0.35**	**0.50**	**0.46**	**0.39**	**0.34**	0.36	0.10
			ecc	0.11	−0.08	−0.06	**−0.21**	−0.08	−0.08	−0.04	−0.10	0.09	0.09	**0.21**	0.13	**0.25**	−0.09	**0.16**	0.06	**0.28**	0.14	0.17	0.12
Ankle	sag	DF	con	**−0.26**	−0.14	**−0.17**	**−0.20**	**−0.27**	−0.15	**−0.27**	−0.14	0.20	0.06	**−0.29**	**−0.17**	−0.10	−0.08	**−0.24**	−0.12	**−0.22**	−0.01	0.15	0.09
			ecc	**−0.28**	−0.13	−0.07	**−0.17**	−0.12	−0.13	**−0.17**	−0.06	0.14	0.07	**−0.32**	**−0.20**	0.03	−0.04	−0.06	−0.06	−0.04	0.04	0.10	0.12
		PF	con	0.01	−0.01	−0.07	0.08	0.09	0.09	−0.03	−0.09	0.06	0.07	0.11	0.11	−0.05	0.13	0.11	**0.18**	0.07	0.03	0.10	0.07
			ecc	**0.32**	**0.21**	0.13	**0.19**	**0.26**	**0.25**	**0.21**	0.01	0.20	0.09	**0.40**	**0.34**	**0.24**	**0.22**	**0.32**	**0.28**	**0.34**	0.15	0.29	0.08
	fron	SUP	con	0.03	0.00	−0.04	−0.10	0.00	0.02	−0.07	−0.10	0.04	0.05	−0.02	0.07	0.01	−0.04	0.02	−0.04	0.02	**−0.24**	0.06	0.09
			ecc	**0.54**	**0.40**	**0.30**	**0.30**	**0.47**	**0.35**	**0.29**	**0.28**	0.36	0.10	**0.54**	**0.42**	**0.45**	**0.27**	**0.35**	**0.26**	**0.39**	**0.28**	0.37	0.10
		PRO	con	**−0.34**	**−0.16**	0.02	−0.14	**−0.21**	−0.06	−0.09	−0.10	0.14	0.11	**−0.31**	**−0.21**	−0.03	0.01	−0.15	−0.04	**−0.21**	−0.05	0.13	0.11
			ecc	0.01	0.11	−0.03	−0.08	0.03	−0.05	0.06	0.00	0.05	0.06	0.06	0.02	0.05	0.10	0.09	0.09	**0.19**	0.03	0.08	0.06

CD in bold—significant difference (*p* < 0.05); red cell—female > male (CD > 0); blue cell—female < male (CD < 0); light colour—small effect (|CD| > 0.11); medium colour—medium effect (|CD| > 0.28); dark colour—large effect (|CD| > 0.43); pink cell—mean CD (the higher the value, the darker the colour); green cell—standard deviation (the higher the value, the darker the colour).

**Table A16:** Effect size Cliff’s Delta (CD) for gender differences in segment angle parameters; sag = sagittal plane; fron = frontal plane; tran = transverse plane; FS = at foot strike; MS = at mid-stance; TO = at toe-off; MAX = maximum; MIN = minimum; ROM = range of motion; MV = mean of CD across footwear conditions; SD = standard deviation between CD of all footwear conditions.

Seg.	Plane	Par.	Individual Pace										10 km/h									
			BF	S1	S2	S3	S4	S5	S6	PS	MV	SD	BF	S1	S2	S3	S4	S5	S6	PS	MV	SD
Pelvis	sag	FS	**−0.49**	**−0.39**	**−0.40**	**−0.44**	**−0.30**	**−0.35**	**−0.36**	**−0.37**	0.39	0.06	**−0.44**	**−0.38**	**−0.35**	**−0.43**	**−0.32**	**−0.40**	**−0.38**	**−0.33**	0.38	0.04
		MS	**−0.45**	**−0.40**	**−0.37**	**−0.42**	**−0.26**	**−0.36**	**−0.30**	**−0.33**	0.36	0.06	**−0.44**	**−0.45**	**−0.40**	**−0.51**	**−0.38**	**−0.45**	**−0.41**	**−0.39**	0.43	0.04
+ posterior tilt		TO	**−0.41**	**−0.29**	**−0.25**	**−0.35**	**−0.21**	**−0.28**	**−0.28**	**−0.27**	0.29	0.06	**−0.37**	**−0.37**	**−0.33**	**−0.36**	**−0.27**	**−0.35**	**−0.32**	**−0.30**	0.33	0.03
− anterior tilt		MAX	**−0.52**	**−0.41**	**−0.38**	**−0.47**	**−0.28**	**−0.37**	**−0.33**	**−0.34**	0.39	0.08	**−0.46**	**−0.48**	**−0.39**	**−0.48**	**−0.33**	**−0.44**	**−0.39**	**−0.37**	0.42	0.06
		MIN	**−0.36**	**−0.32**	**−0.29**	**−0.37**	**−0.19**	**−0.28**	**−0.28**	**−0.29**	0.30	0.06	**−0.37**	**−0.33**	**−0.31**	**−0.38**	**−0.26**	**−0.33**	**−0.33**	**−0.28**	0.33	0.04
		ROM	−0.14	−0.07	−0.10	−0.06	0.00	−0.04	−0.01	0.03	0.06	0.06	**−0.24**	−0.15	−0.08	−0.11	−0.08	−0.04	−0.10	**−0.16**	0.12	0.06
	fron	FS	**−0.19**	−0.10	−0.14	−0.10	**−0.22**	**−0.19**	−0.14	−0.03	0.14	0.06	**−0.24**	**−0.30**	**−0.19**	**−0.22**	**−0.26**	**−0.24**	**−0.24**	**−0.19**	0.24	0.04
		MS	**−0.19**	**−0.26**	**−0.25**	**−0.32**	**−0.36**	**−0.37**	**−0.35**	**−0.26**	0.29	0.06	**−0.26**	**−0.41**	**−0.39**	**−0.38**	**−0.45**	**−0.36**	**−0.46**	**−0.45**	0.39	0.07
+ right side tilt		TO	0.16	0.15	0.15	−0.04	−0.03	0.06	−0.06	−0.09	0.09	0.11	**0.16**	**0.17**	**0.20**	0.12	0.12	**0.21**	0.03	−0.04	0.13	0.09
− left side tilt		MAX	0.10	0.08	0.13	−0.08	−0.07	0.02	−0.11	−0.14	0.09	0.10	0.10	0.09	0.13	0.08	0.05	0.15	−0.04	−0.05	0.09	0.07
		MIN	**−0.24**	**−0.28**	−0.15	**−0.26**	**−0.28**	**−0.28**	**−0.28**	**−0.21**	0.25	0.05	**−0.24**	**−0.42**	**−0.34**	**−0.34**	**−0.40**	**−0.34**	**−0.41**	**−0.39**	0.36	0.06
		ROM	**0.29**	**0.30**	**0.17**	**0.17**	**0.21**	**0.27**	**0.21**	0.12	0.22	0.06	**0.31**	**0.49**	**0.38**	**0.35**	**0.43**	**0.44**	**0.40**	**0.40**	0.40	0.06
	tran	FS	**−0.19**	**−0.22**	**−0.27**	**−0.24**	**−0.26**	**−0.35**	**−0.27**	**−0.24**	0.26	0.05	**−0.25**	**−0.27**	−0.13	**−0.19**	**−0.19**	**−0.22**	**−0.20**	**−0.35**	0.22	0.07
		MS	**−0.16**	**−0.28**	**−0.39**	**−0.35**	**−0.35**	**−0.29**	**−0.27**	**−0.30**	0.30	0.07	**−0.31**	**−0.38**	**−0.34**	**−0.30**	**−0.25**	**−0.28**	**−0.31**	**−0.37**	0.32	0.04
+ CCW rot.		TO	**0.43**	**0.28**	**0.28**	**0.21**	**0.21**	**0.32**	**0.41**	**0.25**	0.30	0.08	**0.37**	**0.18**	**0.29**	**0.23**	**0.24**	**0.21**	**0.32**	**0.18**	0.25	0.07
− CW rot.		MAX	0.02	−0.10	−0.15	**−0.16**	−0.12	−0.07	−0.08	**−0.21**	0.11	0.07	−0.11	−0.15	0.01	−0.04	−0.06	−0.02	0.02	−0.08	0.06	0.06
		MIN	−0.07	**−0.16**	**−0.33**	**−0.26**	**−0.31**	**−0.37**	**−0.21**	**−0.29**	0.25	0.10	**−0.22**	**−0.20**	**−0.25**	**−0.27**	**−0.26**	**−0.36**	**−0.30**	**−0.45**	0.29	0.08
		ROM	0.10	0.06	**0.16**	0.12	**0.17**	**0.30**	0.12	0.09	0.14	0.07	0.07	0.03	**0.26**	**0.23**	**0.16**	**0.30**	**0.30**	**0.31**	0.21	0.11
Thigh	sag	FS	0.05	0.00	**−0.17**	−0.13	−0.09	−0.11	−0.06	−0.11	0.09	0.07	**0.27**	**0.22**	0.04	**0.17**	0.13	0.07	0.15	**0.16**	0.15	0.08
		MS	**0.23**	**0.28**	0.12	0.09	**0.16**	0.06	0.09	0.10	0.14	0.08	0.07	0.08	0.02	0.12	0.05	0.08	0.07	0.13	0.08	0.04
+ posterior tilt		TO	−0.05	−0.11	0.03	**−0.19**	−0.02	**−0.19**	−0.01	−0.15	0.09	0.09	−0.08	**−0.24**	−0.14	**−0.31**	**−0.28**	**−0.34**	**−0.18**	**−0.23**	0.22	0.09
− anterior tilt		MAX	−0.06	−0.12	−0.11	−0.15	−0.09	−0.04	0.00	−0.07	0.08	0.05	0.08	0.15	0.04	0.07	0.09	0.13	**0.24**	0.08	0.11	0.06
		MIN	−0.01	−0.10	0.05	−0.12	−0.01	−0.14	0.00	−0.12	0.07	0.07	−0.09	**−0.24**	−0.11	**−0.26**	**−0.28**	**−0.35**	**−0.20**	**−0.19**	0.21	0.09
		ROM	−0.01	0.01	0.00	0.00	0.01	0.13	0.02	0.11	0.04	0.06	0.13	**0.33**	**0.19**	**0.35**	**0.31**	**0.41**	**0.36**	**0.27**	0.29	0.09
	fron	FS	**0.23**	**0.34**	**0.20**	**0.16**	**0.32**	**0.23**	**0.39**	**0.23**	0.26	0.08	**0.27**	**0.32**	**0.24**	**0.25**	**0.27**	**0.31**	**0.28**	**0.21**	0.27	0.04
		MS	**0.25**	0.11	0.12	**0.20**	**0.30**	**0.19**	**0.23**	0.13	0.19	0.07	**0.30**	**0.25**	**0.28**	**0.24**	**0.32**	**0.22**	**0.26**	**0.28**	0.27	0.03
+ lateral tilt		TO	**0.47**	**0.36**	**0.45**	**0.55**	**0.50**	**0.41**	**0.44**	**0.55**	0.47	0.07	**0.53**	**0.43**	**0.52**	**0.57**	**0.51**	**0.46**	**0.50**	**0.59**	0.51	0.05
− medial tilt		MAX	**0.41**	**0.49**	**0.42**	**0.41**	**0.47**	**0.35**	**0.40**	**0.43**	0.42	0.04	**0.51**	**0.53**	**0.48**	**0.51**	**0.58**	**0.45**	**0.45**	**0.45**	0.49	0.05
		MIN	**0.42**	**0.32**	**0.30**	**0.34**	**0.32**	**0.28**	**0.38**	**0.32**	0.34	0.05	**0.42**	**0.34**	**0.33**	**0.34**	**0.26**	**0.35**	**0.32**	**0.35**	0.34	0.04
		ROM	0.07	**0.21**	0.06	0.05	0.14	0.03	0.02	0.10	0.08	0.06	0.11	**0.26**	**0.19**	0.12	**0.29**	0.05	0.15	**0.17**	0.17	0.08
	tran	FS	0.10	0.08	0.13	**0.28**	0.10	0.12	0.14	**0.20**	0.14	0.07	0.11	0.14	**0.16**	**0.25**	**0.17**	0.13	**0.19**	**0.26**	0.17	0.05
		MS	**0.42**	**0.29**	**0.32**	**0.37**	**0.27**	**0.29**	**0.31**	**0.33**	0.32	0.05	**0.29**	**0.27**	**0.25**	**0.35**	**0.27**	**0.22**	**0.26**	**0.31**	0.28	0.04
+ internal rot.		TO	**0.35**	**0.20**	**0.33**	**0.38**	**0.34**	**0.32**	**0.30**	**0.42**	0.33	0.06	**0.31**	**0.24**	**0.34**	**0.39**	**0.37**	**0.32**	**0.30**	**0.48**	0.34	0.07
− external rot.		MAX	**0.34**	**0.23**	**0.34**	**0.43**	**0.35**	**0.31**	**0.32**	**0.39**	0.34	0.06	**0.34**	**0.29**	**0.35**	**0.42**	**0.42**	**0.34**	**0.33**	**0.44**	0.37	0.05
		MIN	**0.18**	0.11	**0.28**	**0.38**	**0.20**	**0.19**	**0.22**	**0.32**	0.23	0.09	0.12	0.15	**0.18**	**0.33**	**0.23**	**0.16**	**0.21**	**0.32**	0.21	0.08
		ROM	**0.36**	**0.23**	**0.16**	**0.17**	**0.31**	**0.17**	**0.16**	**0.16**	0.21	0.08	**0.42**	**0.30**	**0.38**	**0.22**	**0.43**	**0.30**	**0.33**	**0.36**	0.34	0.07
Shank	sag	FS	−0.03	−0.15	0.04	**−0.24**	**−0.28**	**−0.16**	**−0.32**	−0.08	0.16	0.12	0.07	**0.16**	**0.30**	0.12	−0.02	0.02	**0.18**	**0.20**	0.13	0.10
		MS	−0.01	**0.26**	**0.24**	0.03	−0.05	−0.07	−0.04	0.15	0.11	0.13	−0.12	−0.02	0.10	−0.12	−0.13	**−0.24**	**−0.17**	−0.12	0.13	0.10
+ posterior tilt		TO	**0.32**	**0.46**	**0.42**	**0.37**	**0.32**	**0.38**	**0.28**	**0.42**	0.37	0.06	**0.24**	**0.34**	**0.31**	**0.16**	**0.22**	0.14	0.12	0.09	0.20	0.09
− anterior tilt		MAX	−0.01	−0.15	0.04	**−0.22**	**−0.28**	**−0.17**	**−0.32**	−0.08	0.16	0.13	0.07	**0.16**	**0.30**	0.12	−0.02	0.02	**0.18**	**0.21**	0.13	0.11
		MIN	**0.31**	**0.45**	**0.42**	**0.36**	**0.30**	**0.36**	**0.26**	**0.42**	0.36	0.07	**0.22**	**0.35**	**0.32**	**0.18**	**0.22**	0.13	0.12	0.09	0.20	0.09
		ROM	**−0.31**	**−0.51**	**−0.38**	**−0.48**	**−0.48**	**−0.45**	**−0.47**	**−0.45**	0.44	0.07	**−0.17**	**−0.20**	−0.12	−0.12	**−0.25**	**−0.16**	0.00	0.02	0.13	0.09
	fron	FS	**−0.35**	**−0.39**	**−0.43**	**−0.55**	**−0.54**	**−0.45**	**−0.52**	**−0.57**	0.48	0.08	**−0.44**	**−0.41**	**−0.45**	**−0.54**	**−0.53**	**−0.52**	**−0.54**	**−0.55**	0.50	0.06
		MS	−0.04	**−0.23**	−0.14	0.05	−0.09	−0.09	−0.09	−0.13	0.11	0.08	−0.01	−0.06	−0.07	0.00	−0.10	−0.11	−0.11	−0.07	0.07	0.04
+ lateral tilt		TO	0.02	−0.13	−0.08	0.03	−0.05	−0.08	**−0.19**	0.03	0.07	0.08	−0.04	−0.11	−0.02	−0.02	−0.06	−0.10	**−0.19**	0.04	0.07	0.07
− medial tilt		MAX	−0.02	−0.06	−0.07	0.11	−0.06	−0.04	−0.07	−0.10	0.07	0.06	−0.09	−0.04	−0.05	0.04	−0.07	−0.09	−0.11	−0.05	0.07	0.04
		MIN	0.00	**−0.19**	−0.15	−0.03	**−0.17**	**−0.16**	**−0.21**	−0.07	0.12	0.08	−0.06	**−0.21**	−0.09	−0.09	**−0.16**	**−0.21**	**−0.22**	−0.03	0.14	0.08
		ROM	0.12	**0.23**	**0.17**	**0.22**	**0.25**	**0.29**	**0.35**	0.13	0.22	0.08	0.05	**0.28**	**0.18**	**0.25**	**0.27**	**0.31**	**0.32**	0.11	0.22	0.10
	tran	FS	−0.08	−0.01	−0.04	**0.17**	0.05	−0.01	−0.01	0.14	0.07	0.09	−0.03	0.02	0.01	0.14	0.04	0.02	0.00	0.13	0.05	0.06
		MS	**0.19**	0.01	**0.16**	**0.28**	**0.19**	**0.21**	**0.18**	**0.26**	0.19	0.08	0.15	0.14	**0.20**	**0.26**	**0.23**	**0.18**	**0.18**	**0.24**	0.20	0.04
+ internal rot.		TO	**0.24**	0.10	**0.17**	**0.27**	**0.20**	0.13	0.13	**0.29**	0.19	0.07	**0.23**	0.11	**0.24**	**0.26**	**0.20**	0.15	**0.16**	**0.29**	0.20	0.06
− external rot.		MAX	**0.24**	0.14	**0.18**	**0.30**	**0.22**	**0.20**	**0.17**	**0.30**	0.22	0.06	**0.19**	**0.20**	**0.22**	**0.27**	**0.24**	**0.19**	**0.16**	**0.25**	0.21	0.04
		MIN	**0.17**	0.08	0.09	**0.23**	0.15	0.10	0.10	**0.27**	0.15	0.07	**0.18**	0.09	0.13	**0.22**	0.15	0.12	0.12	**0.26**	0.16	0.06
		ROM	0.15	0.11	**0.18**	0.09	0.09	0.10	0.12	0.03	0.11	0.04	0.03	**0.18**	0.09	0.06	0.13	0.00	0.12	−0.03	0.08	0.07
Foot	sag	FS	**−0.44**	**−0.28**	−0.15	**−0.20**	**−0.24**	**−0.25**	**−0.23**	−0.13	0.24	0.10	**−0.44**	**−0.26**	0.00	0.01	−0.15	−0.13	−0.04	0.03	0.13	0.16
		MS	**−0.41**	−0.06	−0.01	−0.05	**−0.16**	**−0.39**	−0.14	**−0.23**	0.18	0.15	**−0.49**	**−0.32**	**−0.34**	**−0.32**	**−0.35**	**−0.45**	**−0.36**	**−0.39**	0.38	0.06
+ posterior tilt		TO	−0.05	0.13	**0.20**	**0.22**	0.13	0.06	**0.16**	**0.17**	0.14	0.09	−0.01	0.03	0.15	0.07	0.12	−0.07	0.12	0.03	0.07	0.07
− anterior tilt		MAX	**−0.45**	**−0.29**	−0.15	**−0.20**	**−0.23**	**−0.25**	**−0.23**	−0.13	0.24	0.10	**−0.44**	**−0.30**	0.00	0.01	−0.15	−0.13	−0.04	0.03	0.14	0.17
		MIN	−0.05	0.13	**0.21**	**0.22**	0.13	0.06	**0.16**	**0.17**	0.14	0.09	−0.01	0.03	0.15	0.07	0.12	−0.07	0.12	0.03	0.07	0.07
		ROM	**−0.39**	**−0.30**	**−0.18**	**−0.23**	**−0.21**	−0.15	**−0.18**	−0.06	0.21	0.10	**−0.39**	**−0.27**	0.02	0.10	−0.09	0.07	0.06	**0.18**	0.15	0.20
	tran	FS	**0.34**	**0.37**	**0.34**	0.10	**0.24**	**0.28**	**0.24**	**0.32**	0.28	0.09	**0.43**	**0.46**	**0.40**	**0.17**	**0.35**	**0.22**	**0.25**	**0.27**	0.32	0.11
		MS	**0.20**	**0.22**	**0.29**	−0.10	0.00	0.15	0.10	**0.29**	0.17	0.14	**0.28**	**0.36**	**0.39**	0.01	**0.20**	0.15	**0.20**	**0.26**	0.23	0.12
+ internal rot.		TO	**0.39**	**0.31**	**0.33**	−0.06	0.06	0.11	0.09	**0.20**	0.19	0.15	**0.35**	**0.25**	**0.36**	0.03	**0.17**	0.06	0.12	0.13	0.18	0.12
− external rot.		MAX	**0.39**	**0.26**	**0.32**	−0.10	0.01	0.09	0.01	**0.22**	0.17	0.17	**0.35**	**0.28**	**0.39**	0.02	**0.16**	0.12	0.12	**0.20**	0.21	0.13
		MIN	**0.35**	**0.43**	**0.33**	−0.01	**0.22**	**0.26**	**0.19**	**0.32**	0.27	0.14	**0.40**	**0.42**	**0.38**	0.08	**0.32**	0.14	**0.20**	**0.22**	0.27	0.13
		ROM	**0.17**	−0.10	0.05	−0.06	**−0.22**	−0.14	**−0.16**	−0.10	0.12	0.12	0.10	0.02	0.14	−0.04	−0.04	0.09	0.05	0.06	0.07	0.07

CD in bold—significant difference (*p* < 0.05); red cell—female > male (CD > 0); blue cell—female < male (CD < 0); light colour—small effect (|CD| > 0.11); medium colour—medium effect (|CD| > 0.28); dark colour—large effect (|CD| > 0.43); pink cell—mean CD (the higher the value, the darker the colour); green cell—standard deviation (the higher the value, the darker the colour).

**Table A17:** Effect size Cliff’s Delta (CD) for gender differences in peak segment angular velocities; sag = sagittal plane; fron = frontal plane; tran = transverse plane; POST = posterior tilting; ANT = anterior tilting; RIG = right side tilting; LEF = left side tilting; CCW = counterclockwise rotating; CW = clockwise rotating; LAT = lateral tilting; MED = medial tilting; IR = internally rotating; ER = externally rotating; MV = mean of CD across footwear conditions; SD = standard deviation between CD of all footwear conditions.

Seg.	Plane	Par.	Individual Pace										10 km/h									
			BF	S1	S2	S3	S4	S5	S6	PS	MV	SD	BF	S1	S2	S3	S4	S5	S6	PS	MV	SD
Pelvis	sag	POST	0.01	−0.03	−0.03	−0.09	0.08	−0.03	−0.04	0.06	0.05	0.06	0.03	−0.02	0.02	−0.03	0.04	−0.03	0.01	0.02	0.03	0.03
		ANT	0.02	0.06	−0.07	−0.06	−0.02	0.00	−0.05	0.11	0.05	0.06	0.02	0.02	0.11	0.01	−0.02	−0.01	**−0.16**	−0.01	0.04	0.07
	fron	RIG	**0.29**	**0.29**	**0.22**	**0.16**	**0.29**	**0.30**	**0.18**	0.13	0.23	0.07	**0.40**	**0.59**	**0.51**	**0.46**	**0.47**	**0.48**	**0.39**	**0.44**	0.47	0.06
		LEF	0.12	**0.16**	0.10	**0.21**	**0.28**	**0.26**	**0.30**	**0.26**	0.21	0.08	0.09	**0.27**	**0.28**	**0.32**	**0.36**	**0.27**	**0.33**	**0.39**	0.29	0.09
	tran	CCW	**0.38**	**0.18**	**0.18**	0.12	**0.23**	**0.31**	**0.25**	**0.17**	0.23	0.08	**0.41**	**0.19**	**0.33**	**0.22**	**0.33**	**0.32**	**0.37**	**0.41**	0.32	0.08
		CW	−0.03	0.00	−0.05	0.00	0.09	0.09	−0.02	0.03	0.04	0.05	−0.01	0.01	0.15	0.06	**0.17**	**0.20**	0.12	**0.21**	0.12	0.08
Thigh	sag	POST	**−0.26**	**−0.27**	0.13	0.09	0.15	**0.19**	0.10	**0.16**	0.17	0.19	**−0.36**	**−0.16**	0.11	−0.03	0.02	0.08	0.13	0.00	0.11	0.16
		ANT	**−0.16**	−0.08	**−0.20**	−0.08	−0.11	−0.06	**−0.20**	−0.04	0.12	0.06	**0.27**	**0.51**	**0.33**	**0.57**	**0.43**	**0.53**	**0.48**	**0.53**	0.46	0.11
	fron	LAT	**0.19**	**0.16**	0.11	0.05	0.07	−0.08	−0.05	0.05	0.09	0.09	**0.35**	**0.25**	**0.26**	0.10	**0.36**	0.11	**0.16**	**0.21**	0.23	0.10
		MED	**−0.27**	0.02	**−0.19**	**−0.20**	−0.11	−0.13	−0.13	**−0.21**	0.16	0.09	−0.07	0.01	−0.08	**−0.17**	−0.07	−0.05	−0.01	**−0.25**	0.09	0.09
	tran	IR	**0.44**	**0.40**	0.15	**0.29**	**0.44**	**0.27**	**0.31**	**0.30**	0.32	0.10	**0.51**	**0.36**	**0.44**	**0.39**	**0.46**	**0.39**	**0.43**	**0.42**	0.43	0.05
		ER	**0.28**	**0.34**	0.12	0.08	**0.23**	**0.26**	**0.18**	**0.20**	0.21	0.08	**0.32**	**0.32**	**0.47**	**0.17**	**0.35**	**0.39**	**0.40**	**0.30**	0.34	0.09
Shank	sag	POST	**0.23**	**0.28**	**0.28**	**0.21**	**0.31**	**0.36**	**0.23**	**0.31**	0.28	0.05	**0.20**	**0.22**	0.09	**0.19**	**0.17**	**0.30**	0.09	0.08	0.17	0.08
		ANT	**−0.16**	**−0.28**	**−0.37**	**−0.32**	**−0.32**	**−0.28**	**−0.41**	**−0.25**	0.30	0.07	**0.18**	**0.36**	**0.32**	**0.31**	**0.27**	**0.30**	**0.41**	**0.39**	0.32	0.07
	fron	LAT	**0.20**	**0.16**	**0.22**	**0.38**	**0.27**	**0.28**	**0.26**	**0.30**	0.26	0.07	**0.22**	**0.25**	**0.22**	**0.36**	**0.28**	**0.33**	**0.28**	**0.30**	0.28	0.05
		MED	−0.01	−0.05	−0.02	−0.12	−0.11	−0.03	0.08	**−0.25**	0.08	0.10	0.09	0.06	0.07	0.06	−0.01	**0.16**	**0.22**	−0.10	0.10	0.10
	tran	IR	**0.26**	0.15	0.15	0.10	0.08	0.07	0.10	0.05	0.12	0.07	**0.33**	**0.27**	0.15	**0.22**	**0.23**	0.13	**0.20**	0.07	0.20	0.08
		ER	−0.05	0.07	−0.01	−0.02	0.03	0.11	0.05	0.02	0.04	0.05	−0.03	0.07	0.02	0.08	0.04	0.09	0.03	−0.04	0.05	0.05
Foot	sag	POST	**0.37**	0.07	−0.06	−0.05	−0.01	−0.02	−0.08	−0.02	0.08	0.15	**0.32**	0.10	−0.03	0.00	0.00	−0.02	−0.06	0.00	0.07	0.12
		ANT	**−0.20**	**−0.34**	**−0.39**	**−0.46**	**−0.33**	**−0.31**	**−0.35**	**−0.35**	0.34	0.07	**0.23**	**0.23**	**0.21**	**0.17**	**0.17**	**0.31**	**0.17**	**0.18**	0.21	0.05
	tran	IR	0.15	−0.09	−0.03	**−0.21**	**−0.38**	**−0.21**	**−0.19**	−0.12	0.17	0.16	**0.17**	−0.08	0.02	−0.09	−0.13	−0.01	0.03	0.03	0.07	0.09
		ER	−0.11	−0.06	−0.15	0.14	0.11	−0.05	−0.15	−0.07	0.10	0.11	**0.16**	**0.16**	0.10	0.15	0.14	0.15	0.01	0.07	0.12	0.05

CD in bold—significant difference (*p* < 0.05); red cell—female > male (CD > 0); blue cell—female < male (CD < 0); light colour—small effect (|CD| > 0.11); medium colour—medium effect (|CD| > 0.28); dark colour—large effect (|CD| > 0.43); pink cell—mean CD (the higher the value, the darker the colour); green cell—standard deviation (the higher the value, the darker the colour).
